# Thermal and (Thermo-Reversible) Photochemical Cycloisomerization of 1*H*-2-Benzo[*c*]oxocins: From Synthetic Applications to the Development of a New T-Type Molecular Photoswitch

**DOI:** 10.1021/jacs.2c11310

**Published:** 2022-12-22

**Authors:** Minghui Zhou, Simon Mathew, Bas de Bruin

**Affiliations:** Homogeneous, Supramolecular and Bio-Inspired Catalysis (HomKat) Group, van ’t Hoff Institute for Molecular Sciences (HIMS), University of Amsterdam, Science Park 904, 1098 XH Amsterdam, The Netherlands

## Abstract

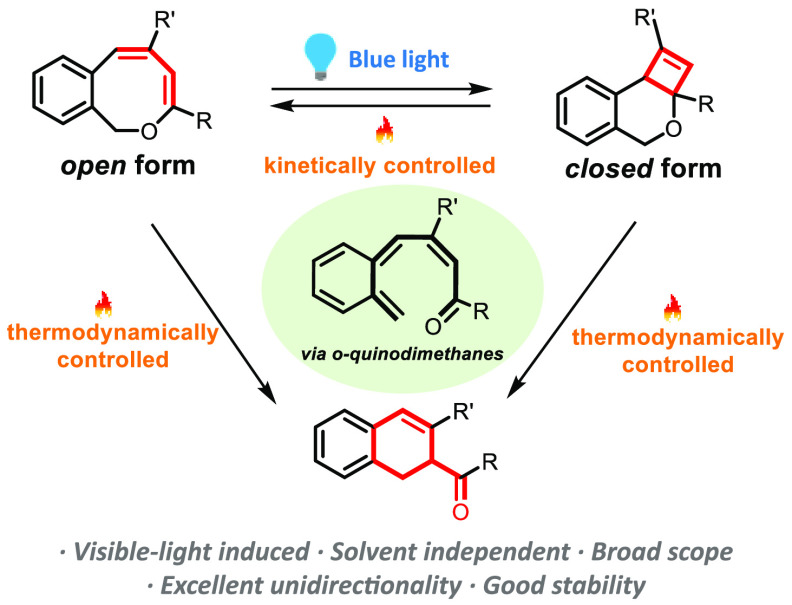

A novel T-type molecular photoswitch based on the reversible cyclization of 1*H*-2-benzo[*c*]oxocins to dihydro-4*H*-cyclobuta[*c*]isochromenes has been developed. The switching mechanism involves a light-triggered ring-contraction of 8-membered 1*H*-2-benzo[*c*]oxocins to 4,6-fused *O*-heterocyclic dihydro-4*H*-cyclobuta[*c*]isochromene ring systems, with reversion back to the 1*H*-2-benzo[*c*]oxocin state accessible through heating. Both processes are unidirectional and proceed with good efficiency, with switching properties—including reversibility and half-life time—easily adjusted via structural functionalization. Our new molecular-switching platform exhibits independence from solvent polarity, originating from its neutral-charge switching mechanism, a property highly sought-after for biological applications. The photoinduced ring-contraction involves a [2+2] conjugated-diene cyclization that obeys the Woodward–Hoffmann rules. In contrast, the reverse process initiates via a thermal ring-opening (*T* > 60 °C) to produce the original 8-membered 1*H*-2-benzo[*c*]oxocins, which is thermally forbidden according to the Woodward–Hoffmann rules. The thermal ring-opening is likely to proceed via an *ortho*-quinodimethane (*o*-QDM) intermediate, and the corresponding switching mechanisms are supported by experimental observations and density functional theory calculations. Other transformations of 1*H*-2-benzo[*c*]oxocins were found upon altering reaction conditions: prolonged heating of the 1*H*-2-benzo[*c*]oxocins at a significantly elevated temperature (72 h at 120 °C), with the resulting dihydronaphthalenes formed via the *o*-QDM intermediate. These reactions also proceed with good chemoselectivities, providing new synthetic protocols for motifs found in several bioactive molecules, but are otherwise difficult to access.

## Introduction

**Figure 1:**
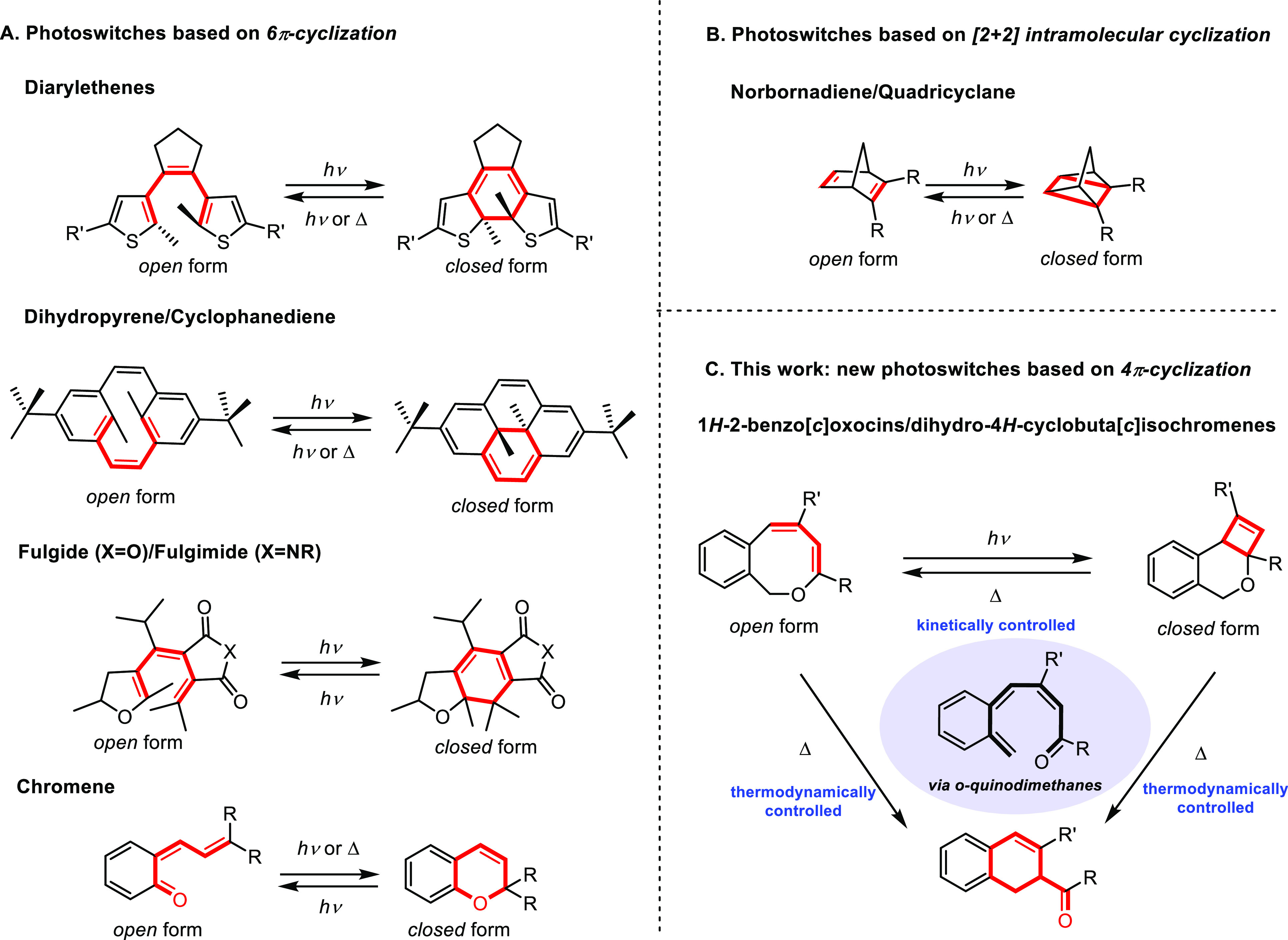
Design of molecular photoswitches by photochemical cyclization reactions. (A) Photoswitches based on 6π-cyclization. (B) Photoswitches based on [2+2] intramolecular cyclization. (C) Present work on 1*H*-2-benzo[*c*]oxocin/dihydro-4*H*-cyclobuta[*c*]isochromene photoswitches and transformations to dihydronaphthalenes under certain conditions.

Photoresponsive functional systems have been studied across a broad range of research disciplines, including nanomachinery,^1^ optical data storage,^2^ photopharmacology,^3^ smart materials,^4^ and solar energy storage.^5^ Such molecules can switch between thermally stable and metastable isomers, typically accompanied with changes in color and/or structure. Transformations from a thermally stable isomer to a metastable structure are typically driven by light, while the reverse process can be driven either photochemically (P-type) or thermally (T-type). A large collection of photoswitchable skeletons have been developed, with their transformations roughly separable into two modes of the photoisomerization process: (1) photochemical double-bond *E*/*Z*-isomerization reactions (i.e., stilbenes,^6^ azobenzenes,^7^ indigos,^8^ and iminothioindoxyls^9^) and (2) photochemical cyclization reactions (i.e., spiropyrans,^10^ diarylethenes,^11^ and norbornadienes^5^). While photoswitching based on double-bond isomerization is frequently employed, light-triggered cycloisomerization reactions that prompt the formation/cleavage of chemical bonds are less frequently encountered as molecular-switching modes of action. For the latter systems, switching via cycloisomerization typically relies on a 6π-cyclization (Figure 1A) or an intramolecular [2+2] cyclization of an unconjugated alkene (Figure 1B).^12^

Efforts have been made to design and synthesize photoresponsive molecules with improved switching properties for specific purposes toward energy storage or switchable material modification. These applications demand features including the complete conversion between isomers, high thermal stability of the metastable state, large geometrical changes upon isomerization, and visible-light sensitivity.^12^ Some of these properties are easier to achieve through photoinduced pericyclic reactions than double-bond isomerization, yet there are only a few reports featuring photoswitches based on the former principle. Most photoswitchable systems feature switching isomers that are similar in size and volume, making the observation of conformational flexibility not obvious and therefore hard to characterize. This lowers the efficiency of isomerization-mediated power transmission in specific applications, such as molecular machines.^1,12^ Switching systems that employ photocyclization possess favorable properties upon comparison to switches based on double-bond isomerization—especially higher thermal stabilities and larger enthalpy differences between isomers^12^—making them excellent candidates for emerging applications such as solar energy storage, molecular logic gates, and smart materials. Therefore, there is a clear impetus to design novel photoresponsive systems that photocyclize with larger geometrical changes, experience less fatigue, and are easily functionalized, all while maintaining a good switching efficiency alongside thermal stability.

Several natural products contain 8-membered ring structures, with many of those being active pharmaceuticals.^13^ Other related medium-sized ring structures also find applications including fragrances^14^ or as ligands in catalysts.^15^ However, the search for alternate applications of 8-membered ring structures (especially photoswitches) has been hindered by synthetic challenges. Based on new insights into metalloradical catalysis, we recently developed a facile method to prepare different kinds of 8-membered rings in high yields.^16^ Herein, we explore the photochemical and thermal reactivity of 1*H*-2-benzo[*c*]oxocins (8-membered *O*-heterocycles), which lead to the development of a novel T-type-photoswitchable system (i.e., the photoinduced metastable isomer can convert back to the stable isomer through thermal relaxation) based on reversible chemical conversion between 1*H*-2-benzo[*c*]oxocins and dihydro-4*H*-cyclobuta[*c*]isochromenes (Figure 1C). These reactions are based on reversible cyclizations, proceed with excellent efficiency, and are accompanied by large geometrical changes. The photoisomerization process exhibits excellent unidirectionality and can be performed in air using nondamaging visible light, and the process is independent of the solvent used. Furthermore, the metastable state demonstrates excellent thermal stability, and the molecular skeleton is easy to functionalize. The combination of these properties is ideal to develop a new smart-material platform in follow-up studies.

Alongside the switching behavior of dihydro-4*H*-cyclobuta[*c*]isochromenes and 1*H*-2-benzo[*c*]oxocins, these compounds are important substructures found in several bioactive molecules/enzymes that are difficult to access synthetically.^17,18^ The same holds for dihydronaphthalenes, which are formed upon prolonged heating of these compounds. While some reports of thermally promoted and UV-irradiation-induced [2+2] cycloaddition reactions are known to yield cyclobutaisochromenes, most of these transformations have a low efficiency due to unwanted side-product formation and/or isomerization.^19^ As for the synthesis of dihydronaphthalenes, most reported strategies focus on the dearomatization of naphthalenes in low/moderate yields due to the relative instability of dihydronaphthalenes compared to the naphthalene starting material. As a result, these transformations have a limited scope, being largely restricted to the formation of dihydronaphthalenes bearing electron-withdrawing functionalities.^20^ In this paper, we disclose efficient synthetic protocols to construct both dihydro-4*H*-cyclobuta[*c*]isochromenes and dihydronaphthalenes, starting from 1*H*-2-benzo[*c*]oxocins. Specifically, we report the visible-light-induced intramolecular [2+2] cyclization of 1*H*-2-benzo[*c*]oxocins to dihydro-4*H*-cyclobuta[*c*]isochromenes and the thermal ring-contraction of 1*H*-2-benzo[*c*]oxocins to produce dihydronaphthalenes (Figure 1C). In the spirit of green chemistry, all transformations reported in this paper are based on simple protocols that involve only light or heat and proceed to the desired products in near quantitative yields with excellent chemoselectivity, under mild conditions, while exhibiting a high atom economy.

## Results and Discussion

### Light-Induced Intramolecular [2+2] Cyclization of 1*H*-2-Benzo[*c*]oxocins to Dihydro-4*H*-cyclobuta[*c*]-isochromenes

During our previous study,^16a^ we observed that some of the prepared 1*H*-2-benzo[*c*]oxocins appeared to be unstable, with some of them slowly converting to other products. We initially assigned this instability to their intrinsic thermal instability at room temperature, but later, we discovered that these conversions are actually triggered upon exposure to sunlight. Therefore, we sought to explore this reactivity in more detail. Since 1*H*-2-benzo[*c*]oxocins have a conjugated-diene structure, we anticipated that a light-induced ring-contraction was occurring. Initial irradiation of a 1*H*-2-benzo[*c*]oxocin **1a** solution (DCM, 365 nm UV light, aerobic conditions) revealed the formation of two new products: dihydro-4*H*-cyclobuta[*c*]isochromene (**1b**, generated by direct light-induced intramolecular [2+2] cyclization) and trace amounts of dihydro-1*H*-epidioxybenzo[*c*]oxocine (**1c**, from a [4+2] cycloaddition reaction with singlet oxygen (^1^O_2_)). Several reactions were performed to obtain more information about these transformations, differing conditions, varying solvents and light sources, and the inclusion of additives (Table 1). According to these experimental results, the formation of both products requires the input of light. Furthermore, UV light is not needed as full conversion can be achieved by using white light (i.e., no conversion for entries 1, 5, 8, and 11 without light, with full conversion observed with white light).

**Table 1:**
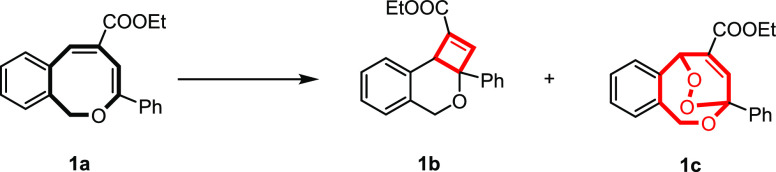
Control Experiments of the Photoinduced Transformations of 1*H*-2-Benzo[*c*]oxocin **1a**

entry^a^	additive	solvent	light source	conversion (%)^b^	**1a**	**1b**	**1c**
1		DCM			100	0	0
2		DCM	white light	fully converted	0	88	12
3^c^		DCM	white light	fully converted	0	100	0
4^c^		DMSO	white light	fully converted	0	100	0
5	[Co(TPP)] (0.1 equiv)	DCM			100	0	0
6	[Co(TPP)] (0.1 equiv)	DCM	white light	25	75	25	0
7	[Co(TPP)] (0.1 equiv)	DCM	UV light (365 nm)	fully converted	0	100	0
8	TPP (0.1 equiv)	DCM			100	0	0
9	TPP (0.1 equiv)	DCM	white light	fully converted	0	0	100
10^c^	TPP (0.1 equiv)	DCM	white light	50	50	25	25
11	CoCl_2_ (1.0 equiv)	DCM			100	0	0
12	CoCl_2_ (1.0 equiv)	DCM	white light	fully converted	0	90	10
13^d^		DCM	white light	fully converted	0	98	2

a Reaction conditions: substrate **1a** (5 mg) and an additive were mixed in DCM (1 mL) and stirred at room temperature for 15 h; reactions were performed in 10 mL vials located 10 cm from the light source.

b Conversion and the ratio of compounds were determined by integration of the ^1^H NMR signals in the presence of dimethyl sulfone as an internal standard.

c The reaction performed under a protective N_2_ atmosphere.

d No stirring.

It is clear that the transformation from **1a** to dihydro-4*H*-cyclobuta[*c*]isochromene **1b** is a noncatalyzed photoisomerization reaction (Table 1, entries 3 and 4). In the presence of air, transformation of **1a** to either dihydro-4*H*-cyclobuta[*c*]isochromene **1b** and dihydro-1*H*-epidioxybenzo[*c*]oxocine **1c** is competitive, but in the absence of a photosensitizer, conversion to dihydro-4*H*-cyclobuta[*c*]isochromene **1b** is predominant (Table 1, entry 2). Reducing the area of the air–solvent interface further inhibits the [4+2] cycloaddition with singlet oxygen leading to dihydro-1*H*-epidioxybenzo[*c*]oxocine **1c** formation, instead affording the dihydro-4*H*-cyclobuta[*c*]isochromene **1b** in a near quantitative yield (Table 1, entry 13). Obviously, the exclusion of oxygen by performing photoisomerization under a protective N_2_ atmosphere leads to the fully selective formation of **1b** (Table 1, entries 3 and 4). In the presence of both air and *meso*-tetraphenylporphyrin (TPP) as a photosensitizer (for in situ photochemical ^1^O_2_ formation),^21^ the [4+2] cycloaddition reaction prevails, leading to selective formation of dihydro-1*H*-3,6-epidioxybenzo[*c*]oxocine **1c** (Table 1, entry 9). CoCl_2_ has almost no influence on the product ratio of **1b** and **1c** for reactions performed under air (Table 1, entries 2 and 12) but in the presence of [Co(TPP)] (i.e., the catalyst used to prepare **1a**) the formation of **1c** is fully suppressed (Table 1, entries 6 and 7). Presumably, the paramagnetic [Co(TPP)] complex catalyzes the relaxation of ^1^O_2_ to ^3^O_2_. However, the intense visible absorption of [Co(TPP)] also causes it to function as an internal light filter in solution, shielding the efficient irradiation of **1a** by the white light source, requiring UV light with a higher power density for full conversion of **1a** to **1b** (Table 1, entries 6 and 7).

Kinetic studies of the ring-contraction from **1a** to **1b** under a protective N_2_ atmosphere revealed that this process is a zero-order reaction, typical for a photocyclization reaction (see the SI for details).^24^ The light-induced isomerization of **1a** to **1b** follows the expected Woodward–Hoffmann rules and proceeds in a disrotatory manner. Different solvents do not have an obvious influence on the yields (Table 1, entries 3 and 4).

**Figure 2:**
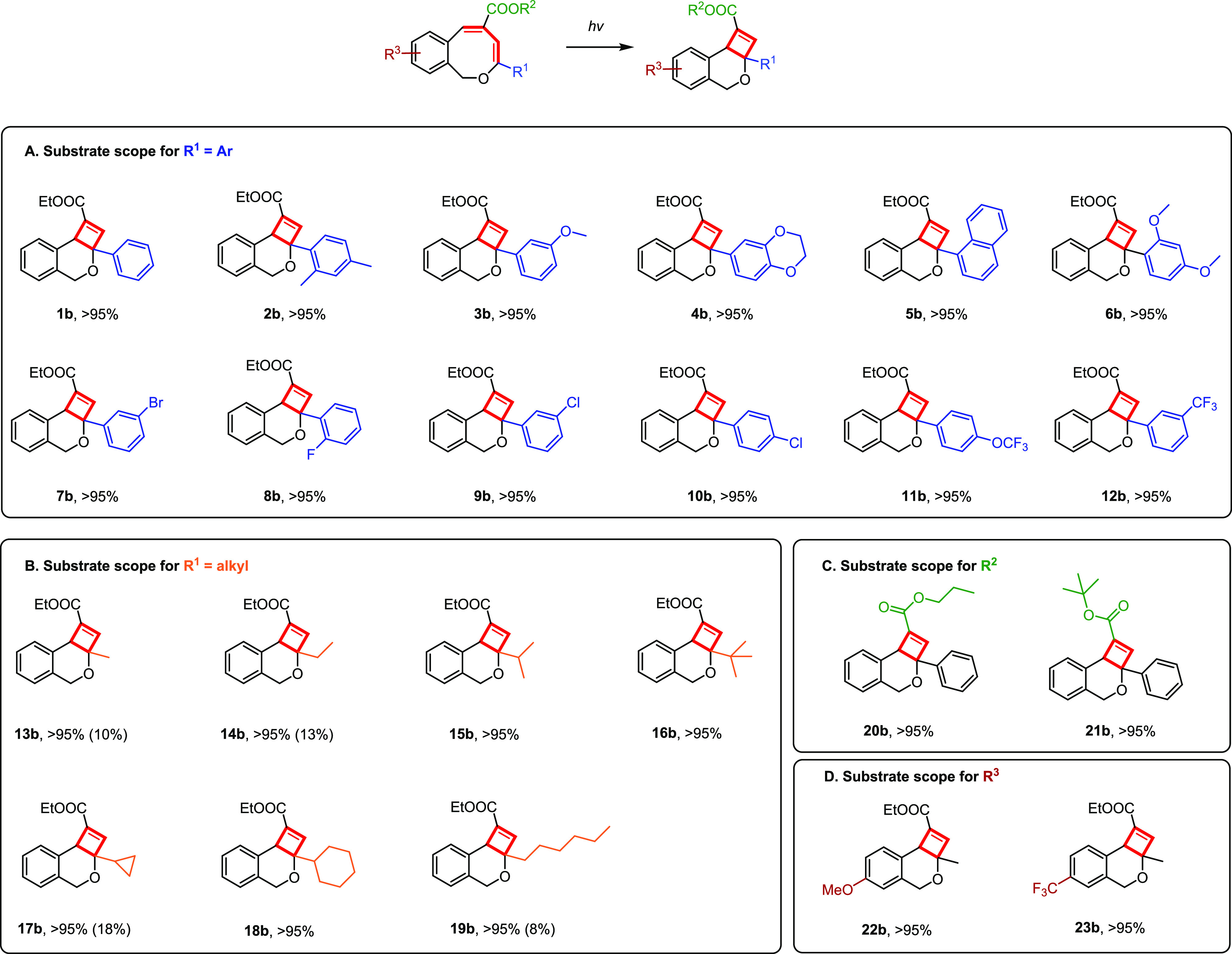
(A–D) Substrate scope of isomerization of 1*H*-2-benzo[*c*]oxocins to dihydro-4*H*-cyclobuta[*c*]isochromenes. Standard reaction conditions: 1*H*-2-benzo[*c*]oxocins (5 mg) dissolved in CD_2_Cl_2_ (0.55 mL); reactions performed in NMR tubes located 10 cm from the light source at room temperature and irradiated for 7 h. Isolated yields. For **13b**–**19b**, **22b**, and **23b**, UV light (365 nm) was used instead of white light. The yields of **14b**, **17b**, and **19b** irradiated with white light are shown between parentheses.

Next, we explored the scope of the photochemical ring-contraction from 1*H*-2-benzo[*c*]oxocins to dihydro-4*H*-cyclobuta[*c*]isochromenes, to elucidate the influence exerted by substituents on other 1*H*-2-benzo[*c*]oxocin analogues (Figure 2). To our delight, all 1*H*-2-benzo[*c*]oxocins with aromatic groups at the enol ether **R_1_** position converted to dihydro-4*H*-cyclobuta[*c*]-isochromenes with great efficiency using visible light (Figure 2A, **1b**–**12b**; Figure 2C, **20b**–**21b**). While modification of **1a** to interrogate the effect of various substituents and positions demonstrates no noticeable influence on the reactivity, 8-membered rings with aliphatic substituents at the enol ether **R_1_** position (Figure 2B, **13b**–**19b**; Figure 2D, **22b**–**23b**) experience low isomerization efficiency when using white light. For these compounds, it was necessary to change the light source to UV light (365 nm) in order to achieve full conversion (Figure 2B,D).

**Figure 3:**
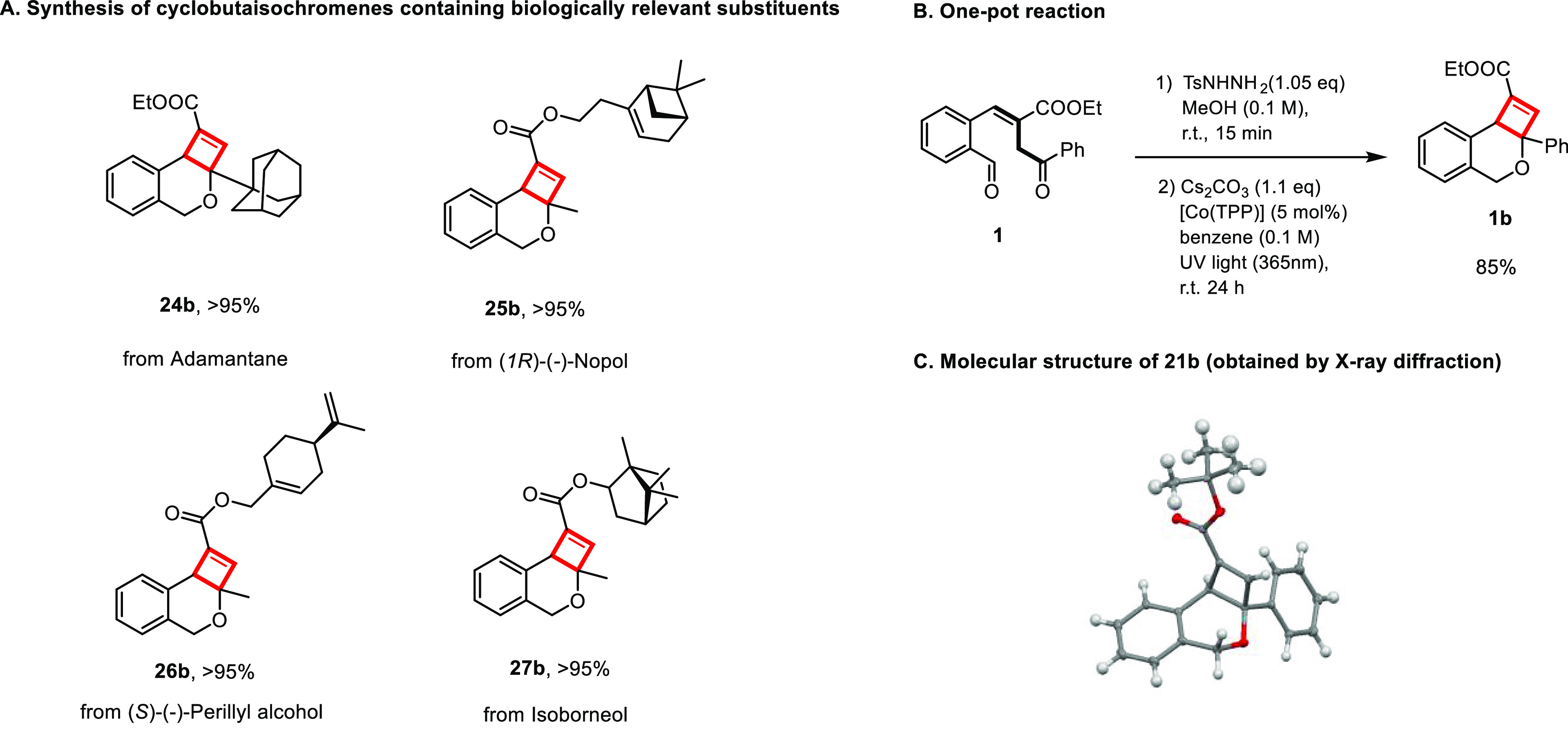
Synthetic practicality and applications of the photochemical conversion of 1*H*-2-benzo[*c*]oxocins to dihydro-4*H*-cyclobuta[*c*]isochromenes. (A) Modification of pharmaceutical derivatives and natural products. (B) One-pot reaction to produce **1b** from the aldehyde precursor **1**. (C) Molecular structure of **21b** (ORTEP diagram with 50% probability ellipsoids), as determined by single-crystal X-ray diffraction.

1*H*-2-Benzo[*c*]oxocins functionalized with biologically relevant substituents could also be isomerized to the desired products in high yields (**24b**–**27b**, Figure 3A). As the control experiments in Table 1 demonstrate that [Co(TPP)] does not interfere with the ring-closing process, a one-pot synthesis of dihydro-4*H*-cyclobuta[*c*]isochromenes was also found to proceed in excellent yield (Figure 3B). Irradiation of aldehyde substrate **1** with UV light yielded 1*H*-2-benzo[*c*]oxocin **1a** in situ, which is immediately photoisomerized to the desired dihydro-4*H*-cyclobuta[*c*]-isochromene **1b** utilizing a one-pot approach.

The structure of dihydro-4*H*-cyclobuta[*c*]isochromene **21b** was confirmed by single-crystal X-ray diffraction (Figure 3C). The X-ray structure clearly confirms that a racemic mixture of the *cis*-product is formed (only one enantiomer within the crystal is shown in Figure 3C, see the SI for details), i.e., featuring the proton and the phenyl group at the quaternary carbon atoms within the 4-membered ring in a mutual *cis*-configuration.

All of the above examples demonstrate that the visible-light-induced conversion of 1*H*-2-benzo[*c*]oxocins to dihydro-4*H*-cyclobuta[*c*]isochromenes is a powerful methodology to construct strained, fused 4,6-ring structures, which widely exist in many bioactive structures.^17^ Moreover, the 1*H*-2-benzo[*c*]oxocins can also be efficiently transformed to dihydro-1*H*-epidioxybenzo[*c*]oxocines under irradiation, in high yields via a [4+2] cycloaddition with photochemically generated ^1^O_2_ using *meso*-tetraphenylporphyrin (TPP) as a photosensitizer (Table 1). Thus, the efficient conversion of dihydro-1*H*-epidioxybenzo[*c*]oxocines also provides efficient synthetic protocols to construct medium-sized rings with strained endoperoxide substituents, which are found in many natural pharmaceuticals with great bioactivity.^22^

### Thermal Ring-Opening of Dihydro-4*H*-cyclobuta[*c*]-isochromenes to 1*H*-2-Benzo[*c*]oxocins

**Figure 4:**
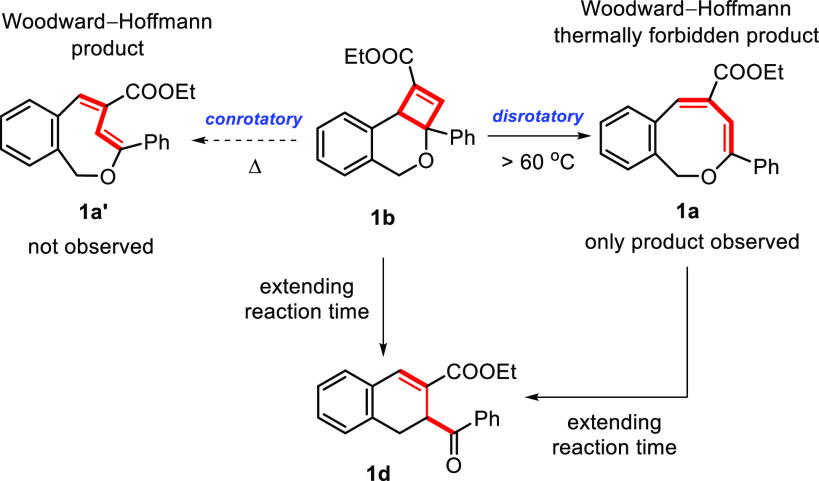
Thermal ring-opening of dihydro-4*H*-cyclobuta[*c*]isochromene **1b**.

**Figure 5:**
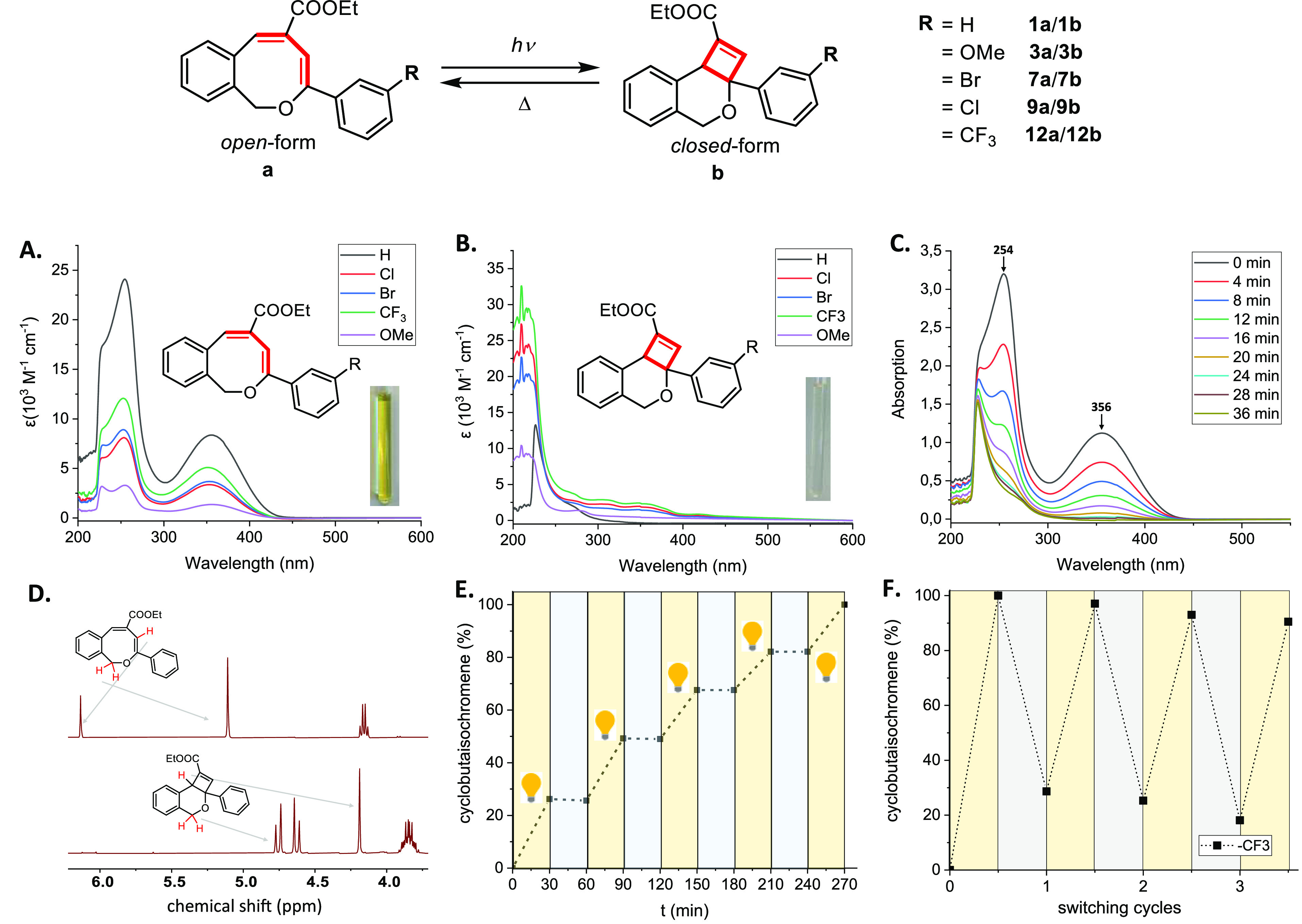
Photoswitching and thermal isomerization behavior. (A) UV/Vis spectra of 1*H*-2-benzo[*c*]oxocins **1a**, **3a**, **7a**, **9a**, and **12a** recorded in DCM. (B) UV/Vis spectra of dihydro-4*H*-cyclobuta[*c*]isochromenes **1b**, **3b**, **7b**, **9b**, and **12b** recorded in DCM. (C) UV/Vis spectra of **1a** recorded upon irradiation with blue LED light in DCM (4.8 × 10^–4^ M), followed in time. (D) ^1^H NMR spectra of **1a** and **1b** recorded in toluene-*d*_8_. (E) Switching of **1a**/**1b** between 30 min periods of dark (in gray) and light (in yellow) with white light in DCM. (F) Switching cycles of **12a**/**12b** in toluene-*d*_8_, consisting of ring-closure (in yellow) and ring-opening (in gray).

The cycloisomerizations to dihydro-4*H*-cyclobuta[*c*]-isochromenes provide a promising, new photoswitchable system that utilizes visible light for 1*H*-2-benzo[*c*]oxocins featuring aryl substituents at the enol ether **R_1_** position. Intrigued by the above results, we continued to investigate the reverse process entailing the ring-opening of the strained, fused 4,6-membered dihydro-4*H*-cyclobuta[*c*]isochromene ring compounds. Initial efforts targeting the photochemical ring-opening of **1b** quickly proved impossible as the intramolecular [2+2] cyclization breaks the conjugated structure of the 1*H*-2-benzo[*c*]oxocins. The resulting dihydro-4*H*-cyclobuta[*c*]isochromenes exhibit absorption in the UV-C region (i.e., colorless, as shown in Figure 5B) that also promote the ring-closure of **1a** to **1b**. However, we noticed that the dihydro-4*H*-cyclobuta[*c*]isochromene **1b** could thermally reverse back to the original 1*H*-2-benzo[*c*]oxocin **1a** in high yield upon mild (>60 °C) heating (Figure 4, see the SI for more details). This result was quite surprising to us, as—according to the Woodward–Hoffmann rules—thermal ring-opening should proceed in a conrotatory manner to produce the twisted 8-membered ring isomer **1a′** as the expected product (Figure 4). However, interrogation of the thermal ring-opening of **1b** in the dark revealed that the formation of the twisted structure **1a′** was not observed at all. We surmise that the twisted 8-membered ring **1a′** is strained (uphill by +9.9 kcal mol^–1^ with respect to **1b** according to DFT, vide infra), hampering its direct formation via thermal ring-opening from **1b**. At the same time, direct thermal ring-opening of **1b** to the nontwisted starting material **1a** violates the Woodward–Hoffmann rules, congruent with our inability to find a transition state between **1b** and **1a** with DFT calculations, as such attempts always led to the hypothetical twisted product **1a′**. In a few rare cases, formation of products violating Woodward–Hoffmann rules has been reported: the ring-opening of some cyclic systems with high ring strain sometimes can lead to thermally forbidden products, but harsh reaction conditions are required to overcome steric barriers.^23a^ Reactions rebelling against Woodward–Hoffmann rules can also be carried out by using a mechanical force to bias reaction pathways.^23b^

We also noticed that the reversible ring-opening process of **1b** to **1a** is sensitive to temperature during the optimization of reaction conditions. As expected, higher reaction temperatures accelerate the process, but applying longer reaction times at high temperatures leads to coformation of another product (Figure 4, dihydronapthalene **1d**, vide infra). For this reason, we employed heating at 110 °C for 10 h as the standard reaction conditions to ensure high switching efficiency for further investigations.

To gain further insight into the photophysical properties and reversibility of the switching process, five different 1*H*-2-benzo[*c*]oxocins with different phenyl-ring substituents were subjected to irradiation (Figure 5 and Table 2, **1a**/**1b** with R = H; **3a**/**3b** with R = OMe; **7a**/**7b** with R = Br; **9a**/**9b** with R = Cl; **12a**/**12b** with R = CF_3_). Importantly, photochemical isomerization of these compounds can be triggered with visible light, conducive to the development of molecular photoswitches with high structural integrity. The UV/Vis spectra of this series of 1*H*-2-benzo[*c*]oxocins were recorded in DCM at room temperature (Figure 5A), revealing absorption maxima (λ_max_) in the UV-A region at 350–360 nm with tailing into visible wavelengths. TD-DFT calculations suggest that this transition (λ = 394 nm) is essentially a singlet-to-singlet HOMO → LUMO π → π* transition. Specifically, the transition is from the carbon–carbon π-bonding donor orbital (HOMO) of the enol ether (−OC(Ph)=CH−) to the carbon–carbon π*-antibonding acceptor orbital (LUMO) of the acrylate (−C(COOR)=CH−), with both the HOMO and the LUMO slightly delocalized into the adjacent aryl groups, see the SI for details. While it is clear that this excited state leads to C–C bond formation via a 4π-cyclization upon irradiation with UV-A light, these photocyclizations can be also initiated with white light, fully consistent with the light yellow color of the substrates originating from the observed tailing of this absorption band into the visible region. The absorption onset (λ_onset_) of the 1*H*-2-benzo[*c*]oxocins in Figure 5 and Table 2 ranges from 442 to 427 nm (i.e., visible violet light), making the photoinduced isomerization accessible with blue LED light. Altering aryl substituents does not perturb the absorption maximum (λ_max_) or the absorption onset (λ_onset_), instead exerting a pronounced influence on molar absorptivity (ε at λ_max_), with the unsubstituted 8-membered ring **1a** exhibiting ε = 8357 L mol^–1^ cm^–1^ (Table 2). Changing the substituents to halogen or electron-withdrawing groups slightly decrease the ε, while **3a** (featuring the electron-donating −OMe substituent) has the lowest ε among these 8-membered rings. The quantum yield of the ring-closure process (Φ_RC_) also exhibits a dependence on the nature of the substituent, with the 1*H*-2-benzo[*c*]oxocin **12a** (featuring an electron-withdrawing −CF_3_ substituent) exhibiting Φ_RC_ = 14%, strongly contrasting **3a** Φ_RC_ = 44%. As such, a higher Φ_RC_ compensates for the lower ε of these compounds (and vice versa, Table 2), revealing why conversions within the same timescale are similar for all these compounds. Additionally, the switching behavior between 1*H*-2-benzo[*c*]oxocins and dihydro-4*H*-cyclobuta[*c*]isochromenes can be followed by UV/Vis or NMR spectroscopy (Figure 5C,D). Due to their high stabilities, the isomer ratio can be controlled simply by on–off irradiation (Figure 5E). Overall, these photophysical measurements demonstrate that the ring-closure isomerization reactions of the 1*H*-2-benzo[*c*]oxocins with aromatic substituents at the enol ether moiety are very efficient, producing the corresponding dihydro-4*H*-cyclobuta[*c*]isochromenes in good yields, with a high Φ_RC_ and without any observable side-product formation.

**Table 2:** Photophysical Data for Compounds with **1a**/**1b**, **3a**/**3b**, **7a**/**7b**, **9a**/**9b**, and **12a**/**12b**

	ring-closure						ring-opening	
–R	Φ_RC_^a^	ε (λ_max_)	λ_max_ (O)	λ_onset_ (O)^b^	isomer yield (O–C)^d^		*t*_1/2_ (25 °C)^c^	isomer yield (C–O)^d^
–OMe	0.44	1360	357	431	>99%		67 y	67%
–H	0.29	8357	355	442	>99%		60 y	62%
–Cl	0.20	3364	353	427	>99%		44 y	68%
–Br	0.43	3680	354	431	>99%		67 y	67%
–CF_3_	0.14	5085	352	432	>99%		46 y	70%

a Photoisomerization quantum yield of ring-closure (λ = 365 nm).

b ε at λ_onset_ is 1% of ε at λ_max_.

c Based on first-order rate constants at 298 K calculated using the Eyring equation.

d The conversion and the ratio of compounds were determined by integration of the ^1^H NMR signals in the presence of dimethyl sulfone as an internal standard.

We also explored the kinetics of the thermal ring-opening of dihydro-4*H*-cyclobuta[*c*]isochromenes to recover 1*H*-2-benzo[*c*]oxocins (Figure 5 and Table 2). The kinetic experiments clearly show that the ring-opening process is a first-order reaction, and the reaction rate is essentially solvent-independent (*k*_(toluene)_ = 1.26 × 10^–3^ s^–1^ and *k*_(DMSO)_ = 1.46 × 10^–3^ s^–1^, both measured at 110 °C in a solution of 1.8 × 10^–2^ M, see the SI for details). The isomer yield going from the closed to open form ranges from 62 to 70% in these cases, with no obvious substituent influence (Table 2). Since the dihydro-4*H*-cyclobuta[*c*]isochromenes are stable at room temperature and no obvious reversible ring-opening products could be observed under ambient conditions, the half-life time (*t*_1/2_) of the ring-closed isomers could be extrapolated from kinetic measurements across the temperature range 90–130 °C using the Eyring equation (SI for details). For the dihydro-4*H*-cyclobuta[*c*]isochromenes mentioned in Figure 5 and Table 2, the *t*_1/2_ ranges from 44 to 67 years at room temperature (25 °C), demonstrating the considerable stability of the metastable isomers during application as photoswitchable materials.

The investigation of switching cycles and fatigue resistance was also performed for this novel switching system. All the switching molecules mentioned in Table 2 and Figure 5 demonstrate good performance in switching cycles, with several rounds of conversions between dihydro-4*H*-cyclobuta[*c*]isochromenes and 1*H*-2-benzo[*c*]oxocins easily achieved by switching between light and heat (switching cycles of **12a**/**12b** are shown in Figure 5F, and more details are shown in the SI). The fatigue resistance of the series reveals a substituent dependence, with compound **12b** (containing a −CF_3_ electron-withdrawing group) exhibiting the best fatigue resistance among these five groups of photoswitches, with only ∼2% dihydronaphthalene formation per switching cycle, formed during the thermal ring-opening process at 110 °C.

To obtain further mechanistic information on the ring-opening process, we set the reaction temperature at 120 °C and prolonged the heating time to 72 h for the thermal conversion of dihydro-4*H*-cyclobuta[*c*]isochromene **1b** (Figure 4). To our surprise, almost full conversion to dihydronaphthalene **1d** was observed, demonstrating that formation of **1d** from **1a** upon prolonged heating is the predominant fatigue pathway of this photoswitching system. This observation is also consistent with our previous work showing that dihydronaphthalenes are the thermodynamically controlled products of the cobalt-catalyzed ring-closure reaction.^16a^

**Figure 6:**
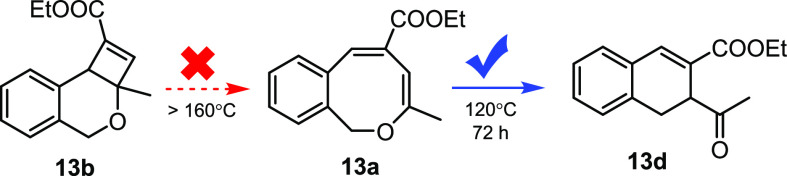
Dihydro-4*H*-cyclobuta[*c*]isochromene **13b** does not thermally relax to 1*H*-2-benzo[*c*]oxocin **13a**, but **13a** contracts to dihydronaphthalene **13d** upon heating to 120 °C.

We attempted to find conditions that facilitate the reversible T-type photoswitching between alkyl-substituted 1*H*-2-benzo[*c*]oxocin **13a** and dihydro-4*H*-cyclobuta[*c*]isochromene **13b** (Figure 6). However, much to our surprise, dihydro-4*H*-cyclobuta[*c*]isochromene **13b**—featuring an aliphatic substituted at the 4-membered ring instead of an aromatic substituent—did not convert back to **13a** at 110 °C, with higher temperatures affording slow decomposition to unknown products. Remarkably, **13a** could convert smoothly to **13d** at 120 °C in 72 h, demonstrating that the formation pathway of **13a** from **13b** is inaccessible thermally. These experimental results suggest that phenyl substituents at the 4-membered ring fragment of dihydro-4*H*-cyclobuta[*c*]isochromenes serve to lower the energy barrier of the ring-opening process when compared to aliphatic substituents.

### Mechanistic Investigations of the Thermal Ring-Opening Process

Previously, we reported DFT studies that support that the pathway to dihydronaphthalene formation shares the same *ortho*-quinodimethane (*o*-QDM) intermediate as accessible pathways leading to the [Co(TPP)]-catalyzed 1*H*-2-benzo[*c*]oxocin formation, with the latter being the kinetically controlled product.^16a^ Specifically, the chemoselectivity for 1*H*-2-benzo[*c*]oxocin formation over (the more thermodynamically stable) dihydronaphthalene was demonstrated to be determined by energy barrier differences of the cyclization process from the *o*-QDM intermediates. Coupling these DFT studies with our observations of dihydronaphthalene (**1d**) formation from dihydro-4*H*-cyclobuta[*c*]isochromene (**1b**) as the predominant origin of switching fatigue during cycling, we anticipated that formation of product **1a** upon heating **1b** at lower temperatures might also proceed via an *o*-QDM intermediate, again with formation of **1a** over **1d** being a kinetically controlled process.

We performed additional DFT studies to shed more light on this matter by finding answers to the following questions: (1) Why does thermal ring-opening of dihydro-4*H*-cyclobuta[*c*]isochromenes lead to thermally forbidden products according to Woodward–Hoffmann rules? (2) Why is the thermal ring-opening of dihydro-4*H*-cyclobuta[*c*]isochromenes to 1*H*-2-benzo[*c*]oxocins associated with dihydronaphthalene formation? (3) Why is thermal switching possible with an aryl substituent at the 4-membered ring but not with alkyl substituents at this position?

**Scheme 1:**
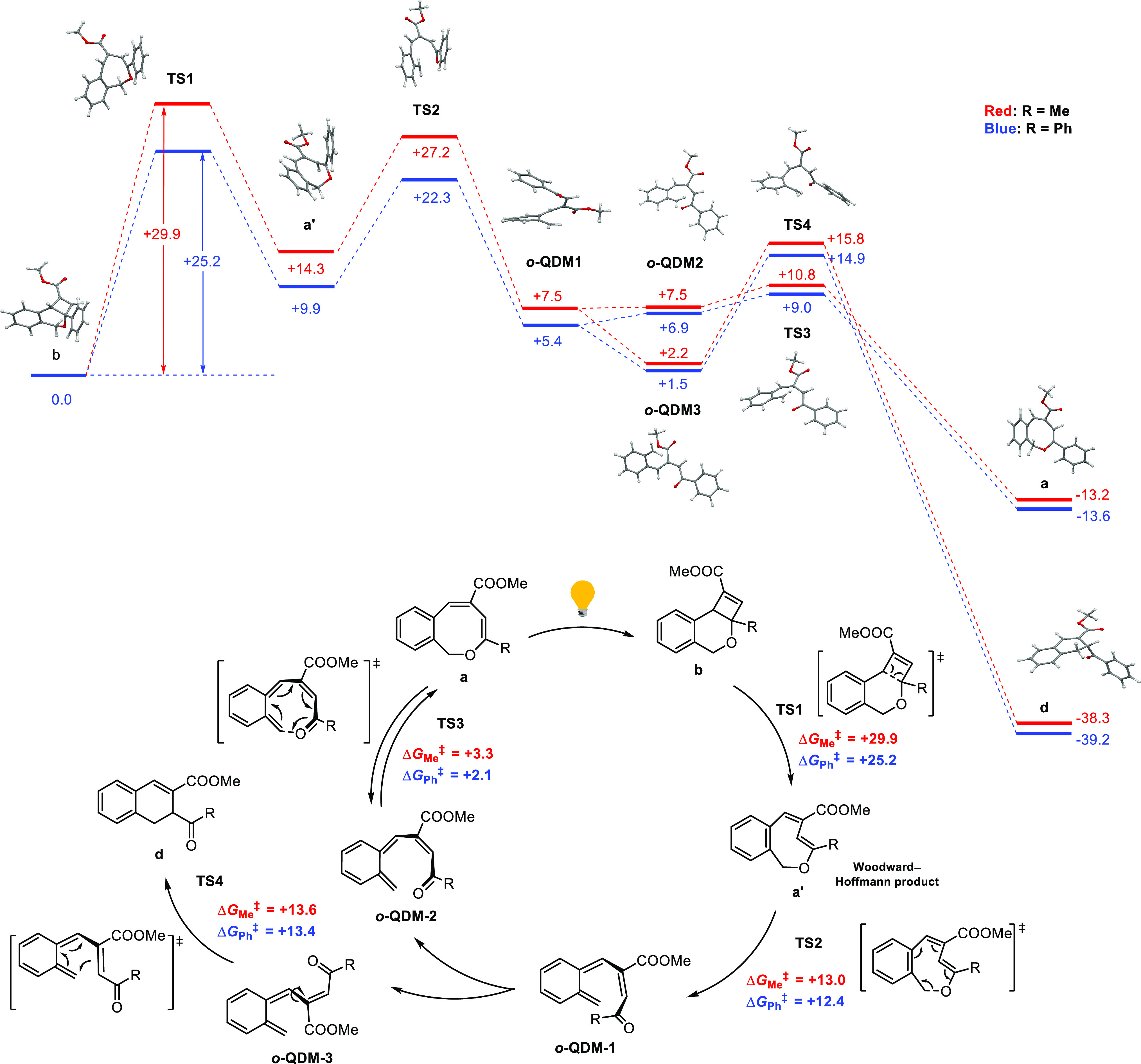
Proposed Mechanism for the Thermal Ring-Opening of Dihydro-4*H*-cyclobuta[*c*]isochromenes (**b**) to 1*H*-2-Benzo[*c*]oxocins (**a**) and Dihydronaphthalenes (**d**), Based on DFT Calculations (b3-lyp, def2-TZVP, m4 grid, and disp3) All Gibbs free energies (Δ*G*°_383K_ in kcal mol^–1^), including **TS1**–**TS4**, are reported relative to the energy of intermediate **b**. The molecular structures belong to the ring-opening process with R = Ph. To reduce computational time, a COOMe group was used instead of COOEt.

As the ring-closure process of conjugated-diene [2+2] intramolecular cyclization induced by light has been well-studied,^24^ we herein only focus on the thermal ring-opening process. We explored pathways using two different types of substituents at the enol ether position (red line for R = Me and blue line for R = Ph). The calculations were performed at the b3-lyp/def2-TZVP level of theory, using Grimme’s D3 dispersion corrections (“zero” damping), taking the experimental observations into consideration. The computed mechanisms are shown in Scheme 1. As expected, direct thermal ring-opening of the 4-membered ring of the dihydro-4*H*-cyclobuta[*c*]isochromene **b** via **TS1** proceeds in a conrotatory manner, following the Woodward–Hoffman rules to produce the twisted 1*H*-2-benzo[*c*]oxocin **a′**. This process is endergonic for both compounds (R = Me: +14.3 kcal mol^–1^; R = H: +9.9 kcal mol^–1^) producing strained, thermodynamically unstable intermediates, featuring a high energy barrier in both cases (DFT R = Me: Δ*G*^‡^_383K_ = +29.9 kcal mol^–1^; DFT R = Ph: Δ*G*^‡^_383K_ = +25.2 kcal mol^–1^; the latter value is close to the experimental free energy barrier for R = Ph, as determined by Eyring analysis: Δ*G*^‡^_383K_ = +30.5 kcal mol^–1^, see SI Table S5). However, the barrier is significantly lower for R = Ph than for R = Me, in good agreement with the experimental observations. The strained twisted intermediates **a′** are thermally unstable and easily ring-open to produce the *o*-QDM intermediate ***o*-QDM-1**, which is an exergonic step with a relatively low energy barrier (R = Me: +13.0 kcal mol^–1^; R = Ph: +12.4 kcal mol^–1^). The molecular structures show that the twisted 8-membered ring **a′** has a helical conformation similar to that of ***o*-QDM-1**, which further explains the facile ring-opening process. A slight rotation around the single bond to convert ***o*-QDM-1** to ***o*-QDM-2** is then followed by a (nearly) barrierless 8π cyclization to produce the 1*H*-2-benzo[*c*]oxocins **a** via **TS3**, thus completing the thermal switching process. This process is again exergonic, featuring a very low energy barrier (R = Me: +3.3 kcal mol^–1^; R = Ph +2.1 kcal mol^–1^). Trace amounts of ***o*-QDM-1** could also convert to ***o*-QDM-3** to undergo 6π-cyclization producing the thermodynamically more stable dihydronaphthalene products **d** via **TS4**, which is also an exergonic process with a low energy barrier (R = Me: +13.6 kcal mol^–1^; R = Ph +13.4 kcal mol^–1^). This explains the occurrence of some fatigue upon thermal switching at high temperatures. However, the energy barrier for conversion of ***o*-QDM-3** to dihydronaphthalene **d** (+13.4 kcal mol^–1^) is substantially higher than the total highest barrier leading to the desired 1*H*-2-benzo[*c*]oxocins **a** (+7.5 kcal mol^–1^) from this same intermediate (at 110 °C), thus explaining the predominant formation of products **a** over products **d** for kinetic reasons (Scheme 1). Meanwhile, the different outcome upon heating dihydro-4*H*-cyclobuta[*c*]-isochromenes with alkyl or aryl groups can be readily explained by the much higher energy barrier needed for thermal ring-opening of the isochromenes with an alkyl group (e.g., R = Me in Scheme 1). For dihydro-4*H*-cyclobuta[*c*]isochromenes with aliphatic substituents, the barrier from **b** to intermediate **a′** is too high to be overcome simply by heating (Scheme 1, 4.7 kcal mol^–1^ higher for R = Me than for R = Ph), which inhibits the formation of *o*-QDM intermediates needed in the follow-up steps. The difference in the energy barrier of ring-opening for dihydro-4*H*-cyclobuta[*c*]isochromenes with alkyl or aryl groups can be explained by the strength of the breaking bond. Compared with the optimized geometries of **b** with methyl and phenyl groups, the length of the breaking C–C bond (via **TS1** to release **a′**) is different: The bond length is slightly longer when R = Ph (R = Me: 1.592 Å; R = Ph: 1.606 Å), suggesting a weaker bond in the aryl analogue. The higher energy barrier for the ring-opening of dihydro-4*H*-cyclobuta[*c*]isochromenes with an alkyl group is likely to be additionally influenced by electronic effects. Aromatic substituents (e.g., R = Ph) at the enol ether position are in electronic conjugation with the π system of the forming conjugated-diene moiety, allowing electronic delocalization in **TS1** providing stabilization of the transition state leading to a lower energy barrier compared with dihydro-4*H*-cyclobuta[*c*]isochromenes substituted with an alkyl group.

**Scheme 2:**
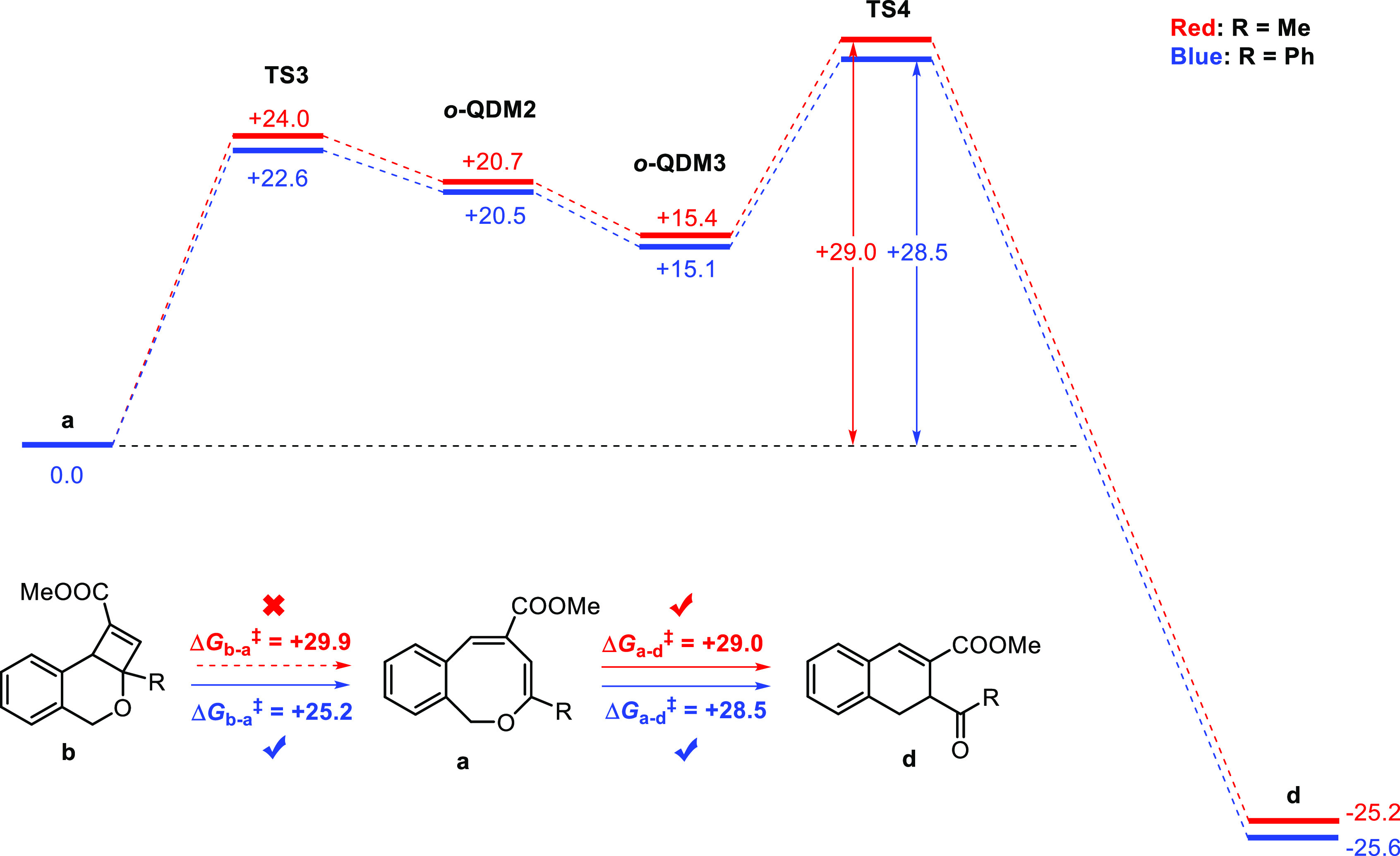
Proposed Mechanism for the Thermal Ring-Contraction of 1*H*-2-Benzo[*c*]oxocins (**a**) to Dihydronaphthalenes (**d**), Based on DFT Calculations (b3-lyp, def2-TZVP, m4 grid, and disp3) All Gibbs free energies (Δ*G*°_383K_ in kcal mol^–1^), including **TS3** and **TS4**, are reported relative to the energy of intermediate **a**. To reduce computational time, a COOMe group was used instead of COOEt.

Apart from a direct pathway involving the thermal ring-opening of dihydro-4*H*-cyclobuta[*c*]isochromenes **b** to dihydronaphthalenes **d** via ***o*-QDM-3**, these species can of course also form by thermal ring-contraction of 1*H*-2-benzo[*c*]oxocins **a**, also proceeding (according to DFT) via the *ortho*-quinodimethide intermediate ***o*-QDM-3** (Scheme 2). One clear observation for the aryl-substituted 1*H*-2-benzo[*c*]oxocins (e.g., R = Ph in Scheme 1) is that the total overall highest-energy barrier from **a** to **d** via **TS4** is +28.5 kcal mol^–1^, which is higher than the total highest barrier for thermal switching of the **b** to **a** pathway (i.e., the barrier to generate **TS1** in Scheme 1; +25.2 kcal mol^–1^ for R = Ph). The DFT results are therefore in agreement with the experimental thermal switching results and explain how the thermally forbidden product **a** can be regenerated—in favor of dihydronapthalene formation—upon mild heating, while applying harsher reaction conditions (i.e., higher temperatures with longer times) leads to formation of the thermodynamically favored dihydronapthalene product **d**.

For aliphatic-substituted 1*H*-2-benzo[*c*]oxocins (e.g., R = Me in Scheme 1), the total energy barrier for conversion of **a** to **d** is similarly high (Scheme 2, +29.0 kcal mol^–1^ for R = Me and +28.5 kcal mol^–1^ for R = Ph). However, thermal ring-opening of **b** to **a′** for R = Me has an even higher DFT-computed barrier (+29.9 kcal mol^–1^, see Scheme 1). While these relative DFT barriers are in qualitative agreement with the experimental results, the **TS4** barrier for R = Me might be underestimated, as the experimental barrier for the ring-opening of **13b** to **13a′** is too high to be overcome by heating at temperatures low enough to prevent unselective decomposition, thus inhibiting the ring-opening switching process of 1*H*-2-benzo[*c*]oxocins with aliphatic substituents.

### Dihydronaphthalene Synthesis

The formation of dihydronaphthalenes containing a ketone functionality connected to the aliphatic part of the partially saturated 6-membered ring from thermal ring-contraction of 1*H*-2-benzo[*c*]oxocins reveals an efficient and convenient approach to access these structures, as they are a key structural motif in many bioactive molecules but are hard to synthesize by existing organic methods.^18^ Therefore, we decided to also explore the generality of thermal ring-contraction from 1*H*-2-benzo[*c*]oxocins to dihydronapthalene performed at higher temperatures (Table 3). In addition to the transformation from **13a** to **13d** (vide supra), all types of 8-membered 1*H*-2-benzo[*c*]oxocins functionalized with different substituents at the enol ether moiety (including alkyls, bulky groups, and aromatic rings) readily convert to dihydronaphthalenes upon prolonged heating to 120 °C. As such, the transformation of 1*H*-2-benzo[*c*]oxocins to dihydronaphthalenes provides a valuable new strategy to construct these skeletons, and this reaction is indeed quite general.

**Table 3:**
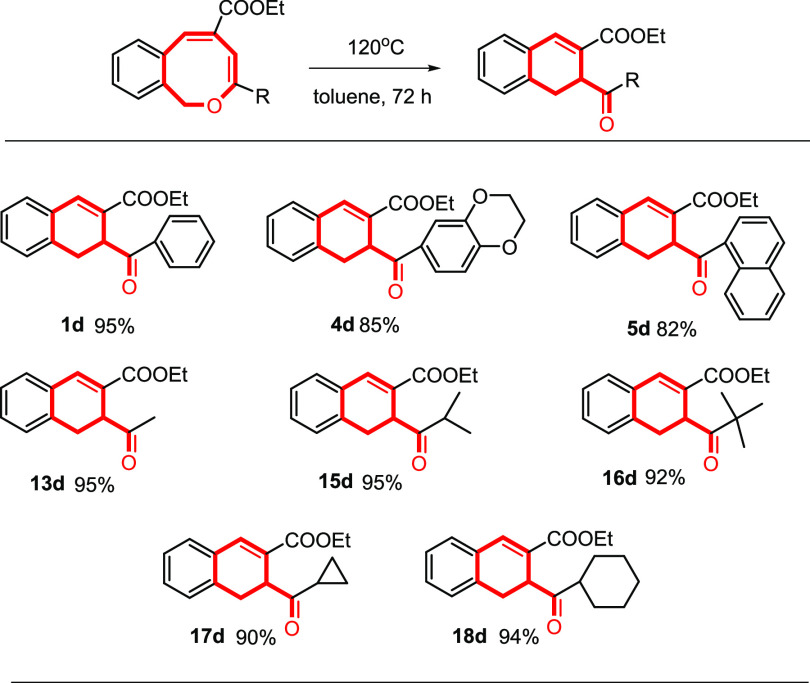
Scope of Dihydronapthalene Formation from 1*H*-2-Benzo[*c*]oxocins^a^

a Reaction conditions: 1*H*-2-benzo[*c*]oxocins (0.05 mmol) dissolved in 1.0 mL of anhydrous toluene in a pressure tube and heating the solution to 120 °C for 3 days. Isolated yields.

## Summary and Conclusions

We developed a novel and powerful T-type photoswitch based on the reversible cyclization between 1*H*-2-benzo[*c*]oxocins and dihydro-4*H*-cyclobuta[*c*]isochromenes. Ring-closure is triggered by light and ring-opening by heat, providing a convenient approach to realize unidirectional switching. The photothermal switch is efficient in both directions, exhibiting outstanding conformational flexibility and high thermal stability in both isomeric states, coupled with sizable quantum yields for the photoreactions. The photoswitching behavior is independent of the solvent polarity and easy to adjust by variation of structural substituents. Visible-light photoactivation and facile functionalization of these new photoswitches are promising features for a broad range of applications, ranging from energy storage to smart materials. The proposed pathways of the thermal conversion are supported by DFT calculations and confirmed with experimental observations. While the light-induced ring-closure adheres to the Woodward–Hoffmann rules, the ring-opening reversion unexpectedly produces thermally forbidden products violating Woodward–Hoffmann rules. This can be explained by ring-opening to an *o*-QDM intermediate that preferentially ring-closes to the desired 1*H*-2-benzo[*c*]oxocin for kinetic reasons. Next to the formation of dihydro-4*H*-cyclobuta[*c*]isochromenes, we also disclosed other transformations of 1*H*-2-benzo[*c*]oxocins: In the presence of air and tetraphenylporphyrin (TPP) as a photosensitizer, photochemical activation of 1*H*-2-benzo[*c*]oxocins leads to formation of dihydro-1*H*-epidioxybenzo[*c*]oxocines via [4+2] cycloaddition of singlet oxygen to the diene moiety of the 1*H*-2-benzo[*c*]oxocin substrates. Heating the 1*H*-2-benzo[*c*]-oxocins at elevated temperatures with a longer reaction time results in formation of dihydronaphthalenes via *o*-QDM intermediates. These reactions also proceed with good chemoselectivities, thus providing new synthetic protocols for substructures that are found in several bioactive molecules but are difficult to prepare otherwise.

## Supporting Information Available

Experimental details; synthesis procedures; relevant NMR, UV/Vis, HRMS, and XRD data; DFT study (PDF)
X-ray structure of **21b** (ZIP)
