# Supplementary material for: Thermal and (Thermo-Reversible) Photochemical Cycloisomerization of 1H-2-Benzo[c]oxocins: From Synthetic Applications to the Development of a New T-Type Molecular Photoswitch
Source: J Am Chem Soc. 2022 Dec 22;145(1):645–57. doi: 10.1021/jacs.2c11310 (PMC9837851; doi:10.1021/jacs.2c11310)

# Supporting Information

Belonging to the paper:

## Thermal and (thermo-reversible) photochemical transformations of 1*H*-2-benzo[c]oxocins; From synthetic applications to development of a new T-type molecular photoswitch

*Minghui Zhou, Simon Mathew and Bas de Bruin**

Homogeneous, Supramolecular and Bio-Inspired Catalysis (HomKat) group, van ’t Hoff Institute for Molecular Sciences (HIMS), University of Amsterdam, Science Park 904, 1098 XH Amsterdam (The Netherlands)

E-mail: B.deBruin@uva.nl

**Contents**

1. General information S2

2. Synthesis of 1*H*-2-benzo[*c*]oxocins S3

3. Photo-isomerization of 1*H*-2-benzo[*c*]oxocins S4

 3.1. General methods of photo-isomerization of 1*H*-2-benzo[*c*]oxocins S4

 3.2. Characterization of dihydro-4*H*-cyclobuta[*c*]isochromenes S7

4. Single crystal X-ray diffraction studies S13

5. Determination of physical and photophysical properties S15

 5.1. Determination of isomers by UV/vis spectra S15

 5.2. Determination of the quantum yields S18

 5.3. Optimization of reaction of thermal ring-opening reaction S20

 5.4. Kinetic studies S21

 5.5. Determination of the half-life times S23

 5.6. Solvent-dependence studies S29

 5.7. Switching cycles S30

6. Synthesis of dihydronaphalenes S32

 6.1. General procedures of the synthesis of dihydronaphalenes S32

 6.2. Characterization of dihydronaphalenes S29

7. Computational details (DFT) S34

 7.1. Computational studies for the thermal ring-opening reaction S34

 7.2. TD–DFT calculations S37

 7.3. Illustration of the ring-opening step S39

 7.4. DFT energy tables S40

 7.5. Optimized geometries S44

8. References in the supporting information S64

9. NMR spectra S65

 9.1. 2D-NMR characterization S65

 9.2. NMR spectra of dihydro-4*H*-cyclobuta[*c*]isochromenes S71

 9.3. NMR spectra of dihydronaphalenes S99

## 1. General Information

Substrate syntheses were carried out open to air and in solvents as received from the supplier, unless otherwise specified. Cobalt catalytic reactions were performed in a nitrogen-filled glove box. Anhydrous solvent (benzene) used for catalysis was degassed. All chemicals were purchased from commercial suppliers (Sigma-Aldrich, Fluorochem or TCI) and used without further purification. Flash column chromatography was performed manually with silica gel. Eluent mixtures are reported as v/v%. Products were visualized by UV light. NMR spectra were measured on a Bruker DRX 500, Bruker AMX 400, Bruker DRX 300 or on a Varian Mercury 300 spectrometer at room temperature. NMR chemical shifts are reported in ppm and are referenced internally to the residual solvent peak of $CDCl_3$ ($^1$H NMR: δ = 7.26 ppm, $^{13}$C NMR: δ = 77.16 ppm), $CD_2Cl_2$ ($^1$H NMR: δ = 5.32 ppm, $^{13}$C NMR: δ = 53.84 ppm), toluene-$d_8$ ($^1$H NMR: δ = 2.08, 6.97, 7.01, 7.09 ppm, $^{13}$C NMR: δ = 137.48, 128.87, 127.96, 125.13, 20.43 ppm). Individual peaks are reported as: multiplicity (s = singlet, d = doublet, t = triplet, q = quartet, m = multiplet), integration, coupling constant (*J*) in Hz. NMR tubes were purchased from Sigma-Aldrich (WG-5MM-ECONOMY-7, OD: 5mm, length: 7'', thin wall: 0.43 mm). High resolution mass spectra (HRMS) were recorded on a JEOL AccuTOF GCv4G, JMS-T100GCV mass spectrometer equipped with a field desorption (FD)/field ionization (FI) probe, fitted with an FI emitter (Carbotec, Germany), 10 µm tungsten wire, flashing a current of 40 mA on each spectrum for 30 ms. HRMS spectra in FI mode were recorded as GC-FI spectra, with GC analysis was performed on a Thermo Scientific Trace GC Ultra equipped with an Agilent 19091S-433 column (30.0 m × 0.25 mm × 0.25 µm). Temperature program: initial temperature 50°C, heat to 315°C with 15°C min$^{-1}$, hold for 5 min. Inlet temperature 230°C, split ratio of 15:1, 1.0 mL min$^{-1}$ helium flow, GC interface at 250°C. HRMS spectra in FI mode were recorded with an FD emitter (Carbotec, Germany), 13 µm tungsten wire, current rate 51.2 mA min$^{-1}$ over 1.2 min. UV/Vis spectra were recorded on a Hewlett Packard 8453 or a double beam Shimadzu UV-2600 spectrometer in a 1.0 cm Teflon screw-cap quartz cuvette using the solvent as a background.

## 2. Synthesis of 1*H*-2-benzo[*c*]oxocins

All the procedures and characterizations of 1*H*-2-benzo[*c*]oxocins in this paper are described in previous report. [1]

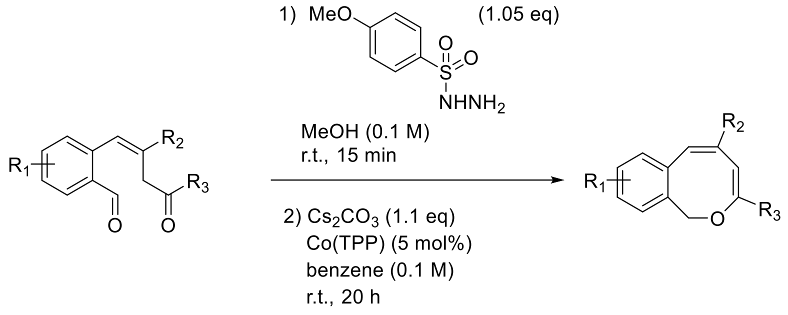


General procedures of the synthesis of 1*H*-2-benzo[*c*]oxocins: aldehyde (0.1 mmol, 1 eq.) and 4-methoxybenzenesulfonohydrazide (0.105 mmol, 1.05 eq.) were dissolved in methanol (0.1 M) in a 4 mL vial. The mixture was stirred for 15 min at room temperature, and then the solvent was removed under reduced pressure. The vial (with mixture inside) was transferred to a nitrogen-filled glovebox, and [Co(TPP)] (0.005 mmol, 0.05 eq.), $Cs_2CO_3$ (0.11 mmol, 1.1 eq.) and benzene (0.1 M) were added subsequently. The mixture was stirred at room temperature for 20 h. The product was directly purified by column chromatography.

## 3. Photo-isomerization of 1*H*-2-benzo[*c*]oxocins

### 3.1. General methods of photo-isomerization of 1*H*-2-benzo[*c*]oxocins

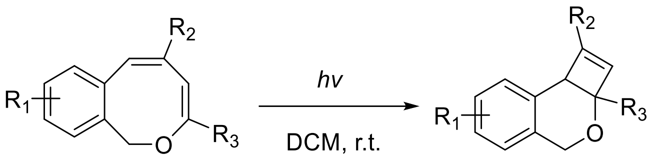


The 1*H*-2-benzo[*c*]oxocins were dissolved in $CD_2Cl_2$ ith NMR tubes, irradiated the NMR tubes by light directly until the isomerization completed.

(1) White light: fluorescence light, 24 W, Calex, 6500K, 180mA, 240V~50/60Hz.

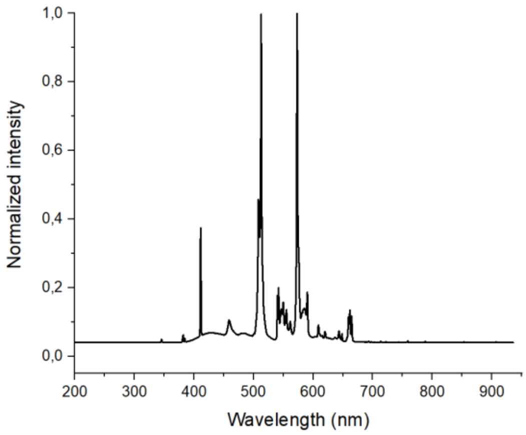


Figure S1. Spectra of fluorescence white light

(2) UV light: LED light, 4W, Philips, TUV, G4, T5, UV-C, with 365nm filter.

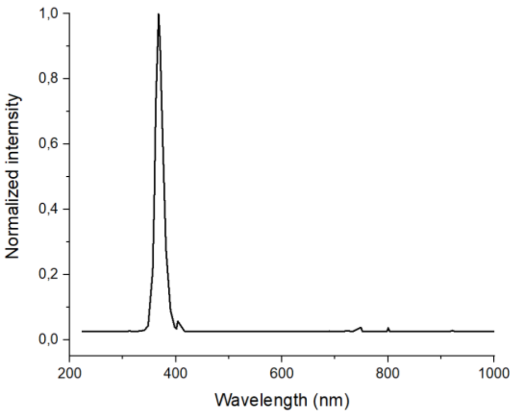


Figure S2. Spectra of UV light

(3) Blue light: LED, Innotas Elektronik, 470nm.

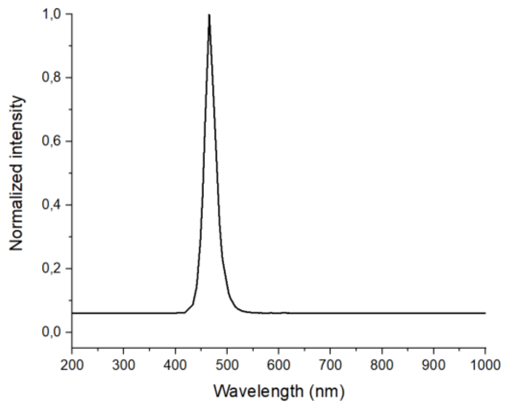


Figure S3. Spectra of blue LED light

**Set-up of the reactions of photoisomerization**

For light source as white light and UV light, reactions were set at a distance of 15 cm away from the light source as follows.

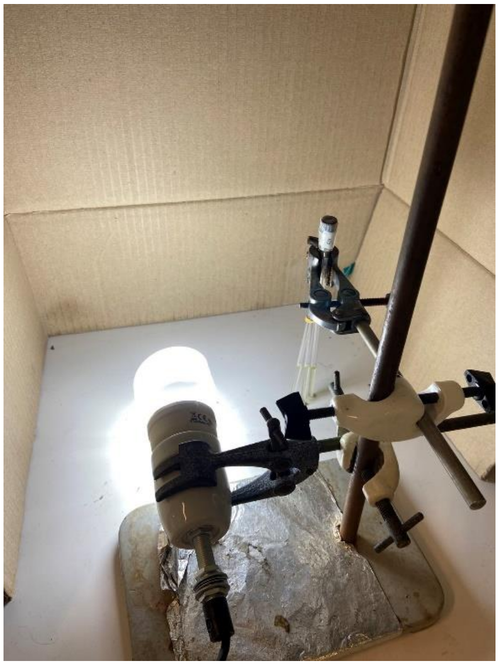

Figure S4. Set-up of photo-isomerization with white/UV light

For light source as blue light, reactions were set at a distance of 5 cm away from the light source as follows.

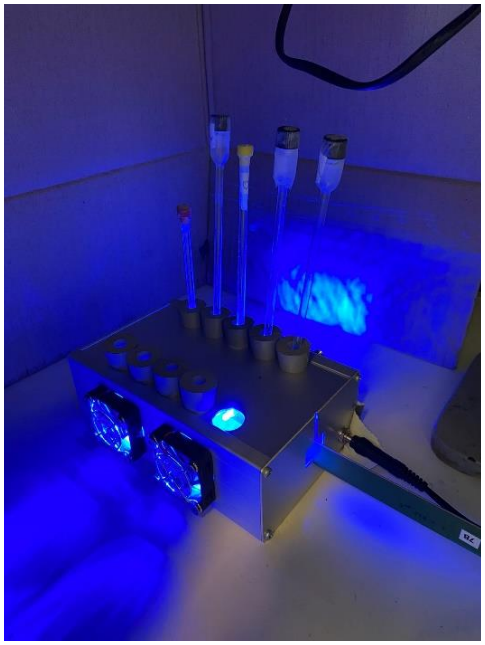

Figure S5. Set-up of photo-isomerization with blue light

### 3.2. Characterization of the dihydro-4*H*-cyclobuta[*c*]isochromenes

**Ethyl 2a-phenyl-2a,8b-dihydro-4*H*-cyclobuta[*c*]isochromene-1-carboxylate (1b)**

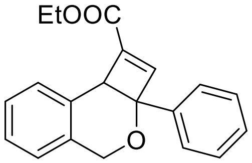


The reaction was irradiated with LED white light for 7 h, > 99% yield. $^1$H NMR (500 MHz, $CDCl_3$) δ 7.56 – 7.50 (m, 2H), 7.41 – 7.25 (m, 6H), 7.20 (d, *J* = 7.3 Hz, 1H), 7.15 (s, 1H), 4.99 – 4.82 (m, 2H), 4.25 (s, 1H), 4.19 (qd, *J* = 7.2, 1.6 Hz, 2H), 1.29 (t, *J* = 7.2 Hz, 3H). $^{13}$C NMR (125 MHz, $CDCl_3$) δ 161.50, 145.28, 140.52, 139.16, 134.56, 133.67, 130.01, 128.38, 127.95, 127.67, 126.65, 125.87, 124.96, 80.41, 64.55, 60.77, 52.86, 14.07. HRMS (FD, *m/z*): calculated for $C_{20}H_{18}O_3$: 306.1256, found: 306.1252.

**Ethyl 3-phenyl-3,6-dihydro-1H-3,6-epidioxybenzo[*c*]oxocine-5-carboxylate (1c)**

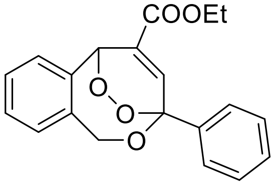


No further purification. Colorless oil, 98% yield. $^1$H NMR (500 MHz, $CDCl_3$) δ 7.67 (d, *J* = 7.5 Hz, 2H), 7.57 (s, 1H), 7.52 – 7.37 (m, 5H), 7.32 (d, *J* = 7.5 Hz, 1H), 7.13 (s, 1H), 6.03 (s, 1H), 4.99 (d, *J* = 14.2 Hz, 1H), 4.85 (d, *J* = 14.2 Hz, 1H), 4.30 (q, *J* = 7.2 Hz, 2H), 1.33 (t, *J* = 7.1 Hz, 3H). $^{13}$C NMR (125 MHz, $CDCl_3$) δ 163.06, 139.70, 137.48, 134.23, 133.78, 133.27, 132.71, 130.91, 129.62, 129.51, 129.19, 128.49, 126.19, 102.39, 79.25, 67.24, 61.78, 14.12. HRMS (FD, *m/z*): calculated for $C_{20}H_{18}O_5$: 338.1154, found: 338.1147.

**Ethyl 2a-(2,4-dimethylphenyl)-2a,8b-dihydro-4H-cyclobuta[*c*]isochromene-1-carboxylate (2b)**

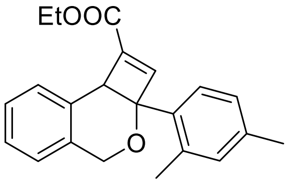


The reaction was irradiated with LED white light for 7 h, > 99% yield. $^1$H NMR (500 MHz, $CDCl_3$) δ 7.44 – 7.15 (m, 6H), 7.07 – 6.95 (m, 2H), 4.95 – 4.69 (m, 2H), 4.31 (s, 1H), 4.15 (q, *J* = 7.2 Hz, 2H), 2.32 (s, 3H), 2.13 (s, 3H), 1.26 (t, *J* = 7.2 Hz, 3H). $^{13}$C NMR (125 MHz, $CDCl_3$) δ 161.65, 144.85, 138.27, 138.17, 137.41, 135.31, 134.30, 134.24, 132.49, 129.65, 127.81, 127.51, 126.59, 125.85, 125.14, 80.77, 64.47, 60.63, 51.23, 21.00, 20.38, 14.06. HRMS (FD, *m/z*): calculated for $C_{22}H_{22}O_3$: 334.1569, found: 334.1571.

**Ethyl 2a-(3-methoxyphenyl)-2a,8b-dihydro-4*H*-cyclobuta[*c*]isochromene-1-carboxylate (3b)**

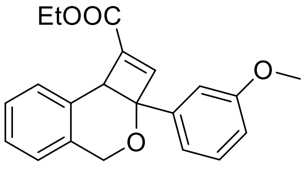


The reaction was irradiated with LED white light for 7 h, > 99% yield. $^1$H NMR (400 MHz, Toluene-$d_8$) δ 7.33 – 6.68 (m, 9H), 4.72 (d, *J* = 13.9 Hz, 1H), 4.59 (d, *J* = 13.9 Hz, 1H), 4.19 (s, 1H), 3.85 – 3.74 (m, 2H), 3.29 (s, 3H), 0.86 (t, *J* = 7.1 Hz, 3H). $^{13}$C NMR (125 MHz, Toluene-$d_8$) δ 160.67, 159.96, 145.14, 142.59, 139.56, 136.87, 134.72, 134.00, 130.16, 129.12, 126.38, 124.81, 117.99, 113.31, 111.65, 80.46, 64.19, 60.04, 54.24, 53.40, 13.60. HRMS (FI, *m/z*): calculated for $C_{21}H_{20}O_4$: 336.1362, found: 336.1361.

**Ethyl2a-(2,3-dihydrobenzo[*b*][1,4]dioxin-6-yl)-2a,8b-dihydro-4*H*-cyclobuta[*c*]isochromene-1-carboxylate (4b)**

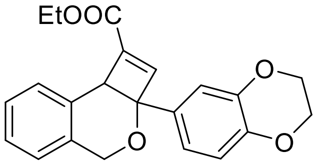


The reaction was irradiated with LED white light for 7 h, > 99% yield. $^1$H NMR (500 MHz, $CDCl_3$) δ 7.40 – 7.27 (m, 2H), 7.21 – 6.85 (m, 6H), 4.91 (dd, *J* = 14.0, 6.1 Hz, 1H), 4.82 (d, *J* = 14.0 Hz, 1H), 4.26 (s, 4H), 4.22 (d, *J* = 6.1 Hz, 1H), 4.17 (q, *J* = 7.0 Hz, 2H), 1.28 (t, *J* = 7.0 Hz, 3H). $^{13}$C NMR (125 MHz, $CDCl_3$) δ 161.52, 145.22, 143.34, 143.32, 139.02, 134.55, 133.86, 133.71, 129.99, 127.62,

126.59, 124.92, 119.07, 117.11, 115.17, 80.03, 64.54, 64.42, 64.36, 60.72, 52.80, 14.07. HRMS (FD, *m/z*): calculated for $C_{22}H_{20}O_5$: 364.1311, found: 364.1300.

**Ethyl 2a-(naphthalen-1-yl)-2a,8b-dihydro-4*H*-cyclobuta[*c*]isochromene-1-carboxylate (5b)**

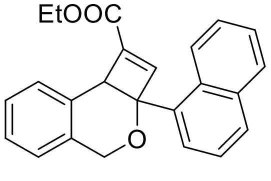


The reaction was irradiated with LED white light for 7 h, > 99% yield. $^1$H NMR (500 MHz, $CD_2Cl_2$) δ 7.84 (dd, *J* = 43.2, 8.4 Hz, 3H), 7.70 – 7.11 (m, 9H), 4.96 (dd, *J* = 77.7, 14.6 Hz, 2H), 4.44 (s, 1H), 4.14 (q, *J* = 7.0 Hz, 2H), 1.26 (t, *J* = 7.0 Hz, 3H). $^{13}$C NMR (125 MHz, $CD_2Cl_2$) δ 161.43, 144.50, 139.12, 136.63, 134.52, 134.26, 134.15, 131.19, 130.05, 129.25, 128.57, 127.88, 126.81, 125.97, 125.79, 125.74, 125.62, 125.33, 124.55, 80.97, 64.60, 60.65, 51.58, 13.82. HRMS (FD, *m/z*): calculated for $C_{24}H_{20}O_3$: 356.1412, found: 356.1428.

**Ethyl 2a-(3,5-dimethoxyphenyl)-2a,8b-dihydro-4*H*-cyclobuta[*c*]isochromene-1-carboxylate (6b)**

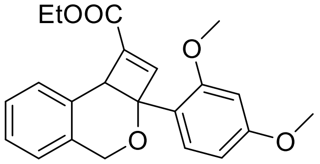


The reaction was irradiated with LED white light for 7 h, > 99% yield. $^1$H NMR (500 MHz, $CD_2Cl_2$) δ 7.46 (d, *J* = 7.9 Hz, 1H), 7.31 (ddd, *J* = 46.4, 15.9, 7.9 Hz, 4H), 7.18 (d, *J* = 7.9 Hz, 1H), 6.51 (t, *J* = 8.9 Hz, 2H), 4.77 (dd, *J* = 85.6, 14.0 Hz, 2H), 4.44 (s, 1H), 4.13 (q, *J* = 7.6 Hz, 2H), 3.84 (s, 3H), 3.74 (s, 3H), 1.26 (t, *J* = 7.5 Hz, 3H). $^{13}$C NMR (125 MHz, $CD_2Cl_2$) δ 161.79, 161.29, 159.25, 145.38, 138.23, 134.95, 129.83, 129.77, 129.70, 127.38, 126.20, 124.83, 120.77, 103.97, 99.18, 78.94, 77.54, 64.13, 60.46, 55.36, 51.12, 13.86. HRMS (FD, *m/z*): calculated for $C_{22}H_{22}O_5$: 366.1467, found: 366.1461.

**Ethyl 2a-(3-bromophenyl)-2a,8b-dihydro-4*H*-cyclobuta[*c*]isochromene-1-carboxylate (7b)**

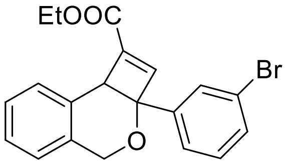


The reaction was irradiated with LED white light for 7 h, > 99% yield. $^1$H NMR (500 MHz, $CD_2Cl_2$) δ 7.71 (d, *J* = 10.2 Hz, 1H), 7.57 – 7.17 (m, 7H), 7.07 (d, *J* = 9.9 Hz, 1H), 4.98 – 4.83 (m, 2H), 4.19 (m, 3H), 1.29 (t, *J* = 7.1 Hz, 3H). $^{13}$C NMR (125 MHz, $CD_2Cl_2$) δ 161.14, 144.48, 143.20, 139.70, 134.60, 133.42, 130.83, 130.03, 129.94, 128.89, 127.62, 126.72, 124.91, 124.38, 122.34, 79.84, 64.40, 60.80, 53.06, 13.83. HRMS (FD, *m/z*): calculated for $C_{20}H_{17}BrO_3$: 384.0361, found: 384.0359.

**Ethyl 2a-(2-fluorophenyl)-2a,8b-dihydro-4*H*-cyclobuta[*c*]isochromene-1-carboxylate (8b)**

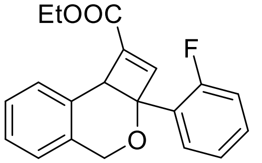


The reaction was irradiated with LED white light for 7 h, > 99% yield. $^1$H NMR (500 MHz, $CDCl_3$) δ 7.48 – 7.31 (m, 3H), 7.28 (s, 3H), 7.22 – 7.05 (m, 3H), 4.92 (d, *J* = 14.0 Hz, 1H), 4.78 (d, *J* = 14.0 Hz, 1H), 4.48 (s, 1H), 4.16 (q, *J* = 6.8 Hz, 2H), 1.29 – 1.25 (t, *J* = 6.8 Hz, 3H). $^{13}$C NMR (125 MHz, $CDCl_3$) δ 161.58, 144.26, 138.83, 134.37, 133.56, 130.37, 130.30, 129.91, 129.44, 129.41, 127.75, 126.69, 125.00, 123.95, 116.33, 116.16, 78.17, 64.44, 60.75, 51.10, 14.06. HRMS (FD, *m/z*): calculated for $C_{20}H_{17}FO_3$: 324.1162, found: 324.1166.

**Ethyl 2a-(3-chlorophenyl)-2a,8b-dihydro-4*H*-cyclobuta[*c*]isochromene-1-carboxylate (9b)**

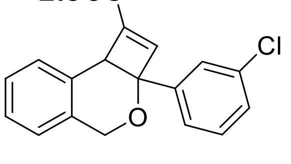


The reaction was irradiated with LED white light for 7 h, > 99% yield. $^1$H NMR (500 MHz, $CD_2Cl_2$) δ 7.55 (d, *J* = 6.7 Hz, 1H), 7.44 (t, *J* = 7.2 Hz, 1H), 7.35 (dp, *J* = 16.3, 7.0 Hz, 5H), 7.24 (t, *J* = 7.0 Hz, 1H), 7.07 (d, *J* = 6.4 Hz, 1H), 4.99 – 4.83 (m, 2H), 4.18 (dd, *J* = 14.7, 7.7 Hz, 3H), 1.31 – 1.28 (m, 3H). $^{13}$C NMR (125 MHz, $CD_2Cl_2$) δ 161.15, 144.51, 142.95, 139.69, 134.60, 134.13, 133.43, 130.02,

129.66, 127.87, 127.61, 126.72, 125.99, 124.91, 123.92, 79.90, 64.40, 60.79, 53.06, 13.83. HRMS (FD, *m/z*): calculated for $C_{20}H_{17}ClO_3$: 340.0866, found: 340.0861.

**Ethyl 2a-(4-chlorophenyl)-2a,8b-dihydro-4*H*-cyclobuta[*c*]isochromene-1-carboxylate (10b)**

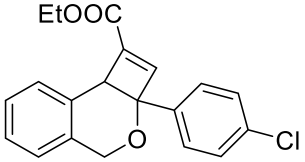


The reaction was irradiated with LED white light for 7 h, > 99% yield. $^1H$ NMR (500 MHz, $CD_2Cl_2$) δ 7.57 – 7.15 (m, 8H), 7.07 (s, 1H), 4.98 – 4.82 (m, 2H), 4.19 (s, 1H), 4.19 – 4.14 (m, 2H), 1.31 – 1.27 (m, 3H). $^{13}C$ NMR (125 MHz, $CD_2Cl_2$) δ 161.19, 144.67, 139.53, 139.45, 134.65, 133.52, 133.47, 130.01, 128.35, 127.59, 127.26, 126.68, 124.91, 79.93, 64.40, 60.77, 53.04, 13.83. HRMS (FD, *m/z*): calculated for $C_{20}H_{17}ClO_3$: 340.0866, found: 340.0871.

**Ethyl 2a-(4-(trifluoromethoxy)phenyl)-2a,8b-dihydro-4*H*-cyclobuta[*c*]isochromene-1-carboxylate (11b)**

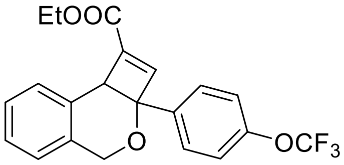


The reaction was irradiated with LED white light for 7 h, > 99% yield. $^1H$ NMR (500 MHz, $CD_2Cl_2$) δ 7.58 (d, *J* = 8.7 Hz, 2H), 7.34 (dq, *J* = 29.1, 7.2 Hz, 3H), 7.24 (dd, *J* = 12.3, 7.7 Hz, 3H), 7.08 (s, 1H), 5.00 – 4.82 (m, 2H), 4.21 (s, 1H), 4.18 (q, *J* = 7.1 Hz, 2H), 1.31 – 1.28 (t, *J* = 7.1 Hz, 3H). $^{13}C$ NMR (125 MHz, $CD_2Cl_2$) δ 161.17, 148.68, 144.60, 139.71, 139.60, 134.63, 133.45, 130.00, 127.60, 127.35, 126.70, 124.91, 120.73, 79.88, 64.42, 60.78, 53.05, 13.82. HRMS (FD, *m/z*): calculated for $C_{21}H_{17}F_3O_4$: 390.1079, found: 390.1078.

**Ethyl 2a-(3-(trifluoromethyl)phenyl)-2a,8b-dihydro-4*H*-cyclobuta[*c*]isochromene-1-carboxylate (12b)**

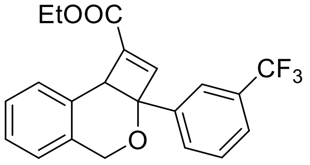


The reaction was irradiated with LED white light for 7 h, > 99% yield. $^1H$ NMR (500 MHz, $CD_2Cl_2$) δ 7.82 (s, 1H), 7.75 (d, *J* = 7.8 Hz, 1H), 7.63 (d, *J* = 7.6 Hz, 1H), 7.54 (t, *J* = 7.9 Hz, 1H), 7.41 – 7.20 (m, 4H), 7.09 (s, 1H), 5.01 – 4.86 (m, 2H), 4.22 (s, 1H), 4.19 (q, *J* = 7.2 Hz, 2H), 1.30 (t, *J* = 7.2 Hz, 3H). $^{13}C$ NMR (125 MHz, $CD_2Cl_2$) δ 161.10, 144.40, 141.95, 139.91, 134.56, 133.31, 130.03, 129.20, 128.87, 127.64, 126.77, 124.93, 124.59, 124.55, 122.55, 122.51, 79.96, 64.42, 60.83, 53.09, 13.81. HRMS (FD, *m/z*): calculated for $C_{21}H_{17}F_3O_3$: 374.1130, found: 374.1137.

**Ethyl 2a-methyl-2a,8b-dihydro-4*H*-cyclobuta[*c*]isochromene-1-carboxylate (13b)**

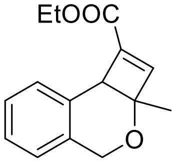


The reaction was irradiated with UV light for 4 h, > 99% yield. $^1H$ NMR (500 MHz, $CD_2Cl_2$) δ 7.50 – 7.04 (m, 4H), 6.91 (d, *J* = 16.2 Hz, 1H), 4.69 (q, *J* = 14.6 Hz, 2H), 4.15 (p, *J* = 7.9, 7.3 Hz, 2H), 3.99 (s, 1H), 1.59 (s, 3H), 1.28 (q, *J* = 8.2, 7.3 Hz, 3H). $^{13}C$ NMR (125 MHz, $CD_2Cl_2$) δ 161.69, 147.73, 137.28, 134.69, 134.18, 130.12, 127.39, 126.23, 124.74, 77.20, 64.42, 60.49, 50.97, 22.76, 13.85. HRMS (FI, *m/z*): calculated for $C_{15}H_{16}O_3$: 244.1099, found: 244.1106.

**Ethyl 2a-ethyl-2a,8b-dihydro-4*H*-cyclobuta[*c*]isochromene-1-carboxylate (14b)**

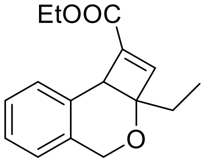


The reaction was irradiated with UV light for 4 h, > 99% yield. $^1H$ NMR (500 MHz, $CD_2Cl_2$) δ 7.46 – 7.04 (m, 3H), 6.95 (d, *J* = 12.2 Hz, 1H), 4.70 (q, *J* = 13.8 Hz, 2H), 4.13 (q, *J* = 7.0 Hz, 2H), 4.00 (s, 1H), 1.89 (q, *J* = 7.2 Hz, 2H), 1.29 – 1.25 (m, 4H), 1.05 – 1.01 (m, 3H). $^{13}C$ NMR (125 MHz, $CD_2Cl_2$) δ 161.63, 146.89, 137.96, 134.99, 134.34, 130.07, 127.34, 126.19, 124.70, 80.25, 64.31, 60.45, 49.32, 29.84, 13.84, 9.01. HRMS (FI, *m/z*): calculated for $C_{16}H_{18}O_3$: 258.1256, found: 258.1260.

**Ethyl 2a-isopropyl-2a,8b-dihydro-4*H*-cyclobuta[*c*]isochromene-1-carboxylate (15b)**

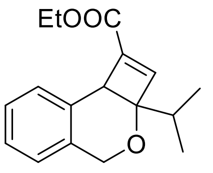

The reaction was irradiated with UV light for 4 h, > 99% yield. $^1H$ NMR (500 MHz, $CDCl_3$) δ 7.34 (dd, *J* = 36.2, 7.6 Hz, 2H), 7.22 (t, *J* = 7.6 Hz, 1H), 7.12 (d, *J* = 7.5 Hz, 1H), 6.97 (s, 1H), 4.76 – 4.66 (m, 2H), 4.15 (qt, *J* = 7.3, 3.3 Hz, 2H), 4.03 (s, 1H), 2.04 (dt, *J* = 13.7, 7.5 Hz, 1H), 1.27 (t, *J* = 7.1 Hz, 3H), 1.10 – 0.97 (m, 6H). $^{13}C$ NMR (125 MHz, $CDCl_3$) δ 161.80, 147.09, 138.23, 134.95, 134.13, 129.97, 127.47, 126.28, 124.76, 82.88, 64.30, 60.56, 48.23, 34.53, 17.81, 17.58, 14.07. HRMS (FD, *m/z*): calculated for $C_{17}H_{20}O_3$: 272.1412, found: 272.1407.

**Ethyl 2a-(tert-butyl)-2a,8b-dihydro-4*H*-cyclobuta[*c*]isochromene-1-carboxylate (16b)**

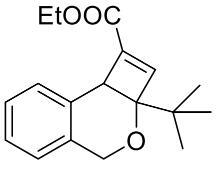

The reaction was irradiated with UV light for 4 h, > 99% yield. $^1H$ NMR (500 MHz, $CDCl_3$) δ 7.36 (d, *J* = 7.5 Hz, 1H), 7.30 (d, *J* = 3.2 Hz, 1H), 7.17 (dt, *J* = 50.4, 7.5 Hz, 2H), 6.91 (d, *J* = 7.5 Hz, 1H), 4.75 – 4.65 (m, 2H), 4.19 (d, *J* = 7.5 Hz, 1H), 4.14 (qd, *J* = 7.5, 3.2 Hz, 2H), 1.26 (t, *J* = 7.2 Hz, 3H), 1.04 (s, 9H). $^{13}C$ NMR (125 MHz, $CDCl_3$) δ 161.62, 146.33, 138.16, 135.27, 134.27, 129.93, 127.40, 126.28, 124.65, 85.62, 64.14, 60.52, 46.07, 35.30, 25.51, 14.05. HRMS (FD, *m/z*): calculated for $C_{18}H_{22}O_3$: 286.1569, found: 286.1567.

**Ethyl 2a-cyclopropyl-2a,8b-dihydro-4*H*-cyclobuta[*c*]isochromene-1-carboxylate (17b)**

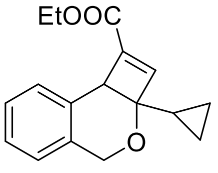

The reaction was irradiated with UV light for 4 h, > 99% yield. $^1H$ NMR (500 MHz, $CDCl_3$) δ 7.38 (d, *J* = 7.5 Hz, 1H), 7.31 (d, *J* = 7.5 Hz, 1H), 7.23 (t, *J* = 7.4 Hz, 1H), 7.12 (d, *J* = 7.5 Hz, 1H), 6.74 (d, *J* = 1.8 Hz, 1H), 4.76 – 4.67 (m, 2H), 4.17 – 4.12 (m, 2H), 3.98 (s, 1H), 1.31 (dd, *J* = 9.4, 5.1 Hz, 1H), 1.29 – 1.26 (m, 3H), 0.63 (hept, *J* = 5.0 Hz, 1H), 0.56 (dq, *J* = 9.5, 4.5 Hz, 1H), 0.48 (dt, *J* = 9.8, 4.7 Hz, 1H), 0.40 (dq, *J* = 9.6, 5.1 Hz, 1H). $^{13}C$ NMR (126 MHz, $CDCl_3$) δ 161.61, 144.64, 138.91, 134.72, 133.82, 130.09, 127.56, 126.41, 124.85, 80.51, 64.54, 60.65, 49.21, 16.01, 14.05, 1.89, 1.41. HRMS (FD, *m/z*): calculated for $C_{17}H_{18}O_3$: 270.1256, found: 270.1262.

**Ethyl 2a-cyclohexyl-2a,8b-dihydro-4*H*-cyclobuta[*c*]isochromene-1-carboxylate (18b)**

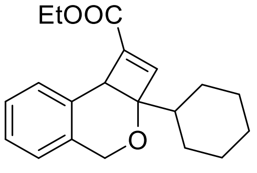

The reaction was irradiated with UV light for 4 h, > 99% yield. $^1H$ NMR (500 MHz, $CD_2Cl_2$) δ 7.45 – 7.04 (m, 4H), 6.96 (s, 1H), 4.74 – 4.62 (m, 2H), 4.13 (q, *J* = 7.2 Hz, 2H), 4.06 (s, 1H), 1.93 – 0.86 (m, 14H). $^{13}C$ NMR (125 MHz, $CD_2Cl_2$) δ 161.61, 146.90, 138.22, 135.30, 134.50, 129.95, 127.32, 126.17, 124.68, 82.28, 64.05, 60.42, 48.26, 44.57, 27.79, 27.68, 26.43, 26.29, 13.85. HRMS (FI, *m/z*): calculated for $C_{20}H_{24}O_3$: 312.1725, found: 312.1736.

**Ethyl 2a-hexyl-2a,8b-dihydro-4*H*-cyclobuta[*c*]isochromene-1-carboxylate (19b)**

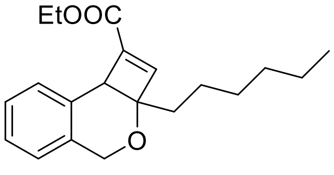

The reaction was irradiated with UV light for 4 h, > 99% yield. $^1H$ NMR (500 MHz, $CD_2Cl_2$) δ 7.43 – 7.03 (m, 4H), 6.93 (s, 1H), 4.69 (q, *J* = 14.3 Hz, 2H), 4.13 (q, *J* = 6.8 Hz, 2H), 3.99 (s, 1H), 1.84 (dd, *J* = 11.0, 5.8 Hz, 2H), 1.48 (dt, *J* = 13.9, 6.5 Hz, 2H), 1.41 – 1.19 (m, 9H), 0.96 – 0.87 (m, 3H). $^{13}C$ NMR (125 MHz, $CD_2Cl_2$) δ 161.67, 147.16, 137.74, 134.97, 134.35, 130.07, 127.34, 126.18, 124.71, 79.84, 64.28, 60.45, 49.81, 36.95, 31.75, 29.58, 25.01, 22.57, 13.85, 13.80. HRMS (FI, *m/z*): calculated for $C_{20}H_{26}O_3$: 314.1882, found: 314.1882.

**Propyl-2a-phenyl-2a,8b-dihydro-4*H*-cyclobuta[*c*]isochromene-1-carboxylate (20b)**

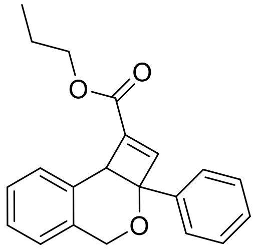


The reaction was irradiated with LED white light for 7 h, > 99% yield. $^{1}$H NMR (400 MHz, $CD_2Cl_2$) δ 7.59 – 7.19 (m, 9H), 7.14 (s, 1H), 5.00 – 4.79 (m, 2H), 4.24 (s, 1H), 4.16 – 4.00 (m, 2H), 1.68 (h, *J* = 7.2 Hz, 2H), 0.95 (t, *J* = 7.4 Hz, 3H). $^{13}$C NMR (125 MHz, $CD_2Cl_2$) δ 161.46, 145.20, 140.74, 139.20, 134.86, 133.84, 130.00, 128.26, 127.81, 127.53, 126.58, 125.80, 124.89, 80.36, 66.27, 64.37, 52.92, 21.84, 10.17. HRMS (FD, *m/z*): calculated for $C_{21}H_{20}O_3$: 320.1412, found: 320.1413.

**Tert-butyl 2a-phenyl-2a,8b-dihydro-4*H*-cyclobuta[*c*]isochromene-1-carboxylate (21b)**

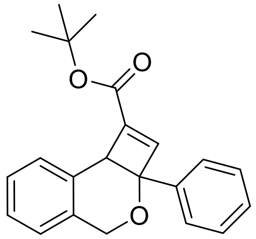


The reaction was irradiated with LED white light for 7 h, > 99% yield. $^{1}$H NMR (500 MHz, $CD_2Cl_2$) δ 7.58 – 7.32 (m, 7H), 7.29 (t, *J* = 7.3 Hz, 1H), 7.23 (d, *J* = 7.5 Hz, 1H), 7.05 (s, 1H), 4.97 – 4.81 (m, 2H), 4.19 (s, 1H), 1.47 (s, 9H). $^{13}$C NMR (125 MHz, $CD_2Cl_2$) δ 160.68, 144.09, 140.95, 140.62, 134.84, 134.01, 130.04, 128.21, 127.74, 127.36, 126.45, 125.89, 124.84, 81.33, 80.03, 64.33, 52.92, 27.75. HRMS (FD, *m/z*): calculated for $C_{22}H_{22}O_3$: 334.1569, found: 334.1565.

**Ethyl 6-methoxy-2a-methyl-2a,8b-dihydro-4*H*-cyclobuta[*c*]isochromene-1-carboxylate (22b)**

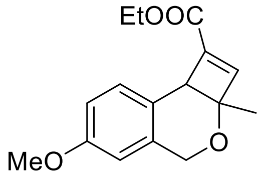


The reaction was irradiated with UV light for 4 h, > 99% yield. $^{1}$H NMR (500 MHz, $CD_2Cl_2$) δ 7.29 (d, *J* = 8.4 Hz, 1H), 6.94 – 6.82 (m, 2H), 6.70 (d, *J* = 13.9 Hz, 1H), 4.72 – 4.65 (m, 1H), 4.61 (d, *J* = 14.0 Hz, 1H), 4.13 (q, *J* = 7.2 Hz, 2H), 3.94 (s, 1H), 3.81 (s, 3H), 1.35 – 1.27 (m, 3H). $^{13}$C NMR (125 MHz, $CD_2Cl_2$) δ 161.80, 158.16, 147.37, 137.77, 135.73, 131.12, 126.12, 113.17, 110.00, 77.27, 64.51, 60.44, 55.21, 50.30, 22.75, 13.85. HRMS (FI, *m/z*): calculated for $C_{16}H_{18}O_4$: 274.1205, found: 274.1201.

**Ethyl 2a-methyl-6-(trifluoromethyl)-2a,8b-dihydro-4*H*-cyclobuta[*c*]isochromene-1-carboxylate (23b)**

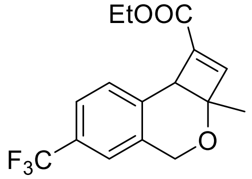


The reaction was irradiated with UV light for 4 h, > 99% yield. $^{1}$H NMR (400 MHz, $CD_2Cl_2$) δ 7.64 – 7.50 (m, 2H), 7.42 (s, 1H), 6.92 (s, 1H), 4.74 (s, 2H), 4.14 (q, *J* = 7.1 Hz, 2H), 4.06 (s, 1H), 1.61 (s, 3H), 1.27 (t, *J* = 7.2 Hz, 3H). $^{13}$C NMR (125 MHz, $CD_2Cl_2$) δ 161.41, 148.02, 138.58, 136.76, 135.32, 130.68, 124.02, 123.99, 121.73, 121.70, 77.30, 64.13, 60.66, 50.71, 22.63, 13.83. HRMS (FI, *m/z*): calculated for $C_{16}H_{15}F_3O_3$: 312.0973, found: 312.0974.

**Ethyl 2a-((3r,5r,7r)-adamantan-1-yl)-2a,8b-dihydro-4*H*-cyclobuta[*c*]isochromene-1-carboxylate (24b)**

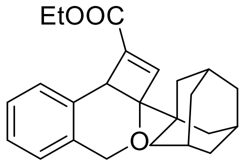


The reaction was irradiated with UV light for 4 h, > 99% yield. $^{1}$H NMR (400 MHz, $CD_2Cl_2$) δ 7.37 (d, *J* = 7.5 Hz, 1H), 7.30 (t, *J* = 7.4 Hz, 1H), 7.23 (t, *J* = 7.4 Hz, 1H), 7.13 (d, *J* = 7.5 Hz, 1H), 6.92 (s, 1H), 4.68 (q, *J* = 14.0 Hz, 2H), 4.26 (s, 1H), 4.12 (q, *J* = 7.2 Hz, 2H), 2.01 (s, 3H), 1.83 – 1.64 (m, 12H), 1.32 – 1.27 (m, 3H). $^{13}$C NMR (125 MHz, $CD_2Cl_2$) δ 161.43, 146.09, 138.20, 135.74, 134.66, 129.92, 127.27, 126.17, 124.59, 85.00, 63.70, 60.41, 44.93, 37.01, 36.86, 28.51, 13.83. HRMS (FD, *m/z*): calculated for $C_{24}H_{28}O_3$: 364.2038, found: 364.2049.

**2-((1S,5R)-6,6-dimethylbicyclo[3.1.1]hept-2-en-2-yl)ethyl-2a-methyl-2a,8b-dihydro-4*H*-cyclobuta[*c*]iso-chromene-1-carboxylate (25b)**

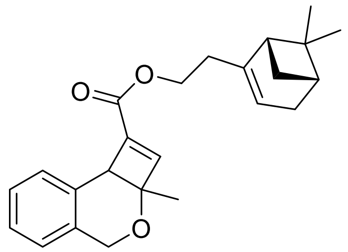

The reaction was irradiated with UV light for 4 h, > 99% yield. $^{1}$H NMR (400 MHz, $CD_2Cl_2$) δ 7.42 – 7.19 (m, 3H), 7.13 (d, *J* = 7.5 Hz, 1H), 6.88 (s, 1H), 5.34 – 5.27 (m, 1H), 4.68 (q, *J* = 14.1 Hz, 2H), 4.10 (td, *J* = 6.9, 4.6 Hz, 2H), 3.98 (s, 1H), 2.38 (dt, *J* = 8.5, 5.6 Hz, 1H), 2.31 (t, *J* = 7.3 Hz, 2H), 2.27 – 2.20 (m, 1H), 2.10 (d, *J* = 3.3 Hz, 1H), 2.08 – 2.02 (m, 1H), 1.58 (s, 3H), 1.29 (s, 3H), 1.14 (d, *J* = 8.6 Hz, 1H), 0.92 (td, *J* = 8.0, 6.6, 2.9 Hz, 1H), 0.81 (d, *J* = 16.1 Hz, 3H). $^{13}$C NMR (125 MHz, $CD_2Cl_2$) δ 161.72, 147.85, 144.12, 137.25, 134.69, 134.15, 130.14, 127.43, 126.24, 124.74, 118.89, 77.22, 64.41, 62.79, 51.00, 45.66, 40.80, 37.87, 35.73, 31.55, 31.33, 25.99, 22.74, 20.80. HRMS (EI, *m/z*): calculated for $C_{24}H_{28}O_3$: 364.2038, found: 364.2044.

**((R)-4-(prop-1-en-2-yl)cyclohex-1-en-1-yl)methyl-2a-methyl-2a,8b-dihydro-4*H*-cyclobuta[*c*]isochromene-1-carboxylate (26b)**

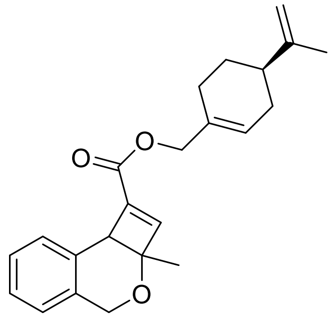

The reaction was irradiated with UV light for 4 h, > 99% yield. $^{1}$H NMR (400 MHz, $CD_2Cl_2$) δ 7.42 – 7.10 (m, 4H), 6.92 (s, 1H), 5.75 (s, 1H), 4.80 – 4.73 (m, 2H), 4.68 (dd, *J* = 18.5, 12.1 Hz, 2H), 4.53 (dd, *J* = 12.4, 7.9 Hz, 1H), 4.42 (dd, *J* = 12.2, 3.9 Hz, 1H), 4.00 (s, 1H), 2.25 – 1.82 (m, 4H), 1.78 (s, 3H), 1.59 (s, 3H), 1.57 – 1.37 (m, 1H), 1.34 (d, *J* = 6.0 Hz, 1H), 0.91 (dt, *J* = 10.6, 7.1 Hz, 1H). $^{13}$C NMR (125 MHz, $CD_2Cl_2$) δ 161.65, 149.80, 148.13, 137.18, 134.76, 134.16, 132.45, 130.16, 127.37, 126.29, 125.96, 124.79, 108.46, 77.30, 68.40, 64.44, 51.05, 40.81, 30.46, 27.34, 26.31, 22.72, 20.48. HRMS (EI, *m/z*): calculated for $C_{23}H_{26}O_3$: 350.1882, found: 350.1891.

**(1R,4R)-1,7,7-trimethylbicyclo[2.2.1]heptan-2-yl-2a-methyl-2a,8b-dihydro-4*H*-cyclobuta[*c*]iso-chromene-1-carboxylate (27b)**

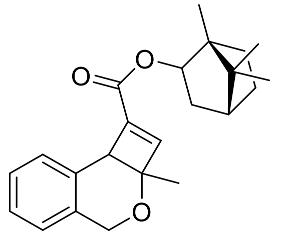

The reaction was irradiated with UV light for 4 h, > 99% yield. $^{1}$H NMR (400 MHz, $CD_2Cl_2$) δ 7.42 – 7.10 (m, 4H), 6.87 (d, *J* = 2.8 Hz, 1H), 4.77 – 4.60 (m, 3H), 3.98 (d, *J* = 3.0 Hz, 1H), 1.81 – 1.69 (m, 1H), 1.58 (s, 3H), 1.32 (m, 5H), 1.16 – 1.09 (m, 1H), 1.04 (d, *J* = 17.3 Hz, 3H), 0.88 (s, 3H), 0.82 (m, 3H). $^{13}$C NMR (125 MHz, $CD_2Cl_2$) δ 161.46, 147.80, 137.64, 134.89, 134.23, 130.30, 127.43, 126.33, 124.85, 81.50, 77.25, 64.42, 51.13, 48.71, 46.89, 45.16, 38.70, 33.78, 31.92, 26.91, 22.70, 19.88, 11.20. HRMS (EI, *m/z*): calculated for $C_{23}H_{28}O_3$: 352.2038, found: 352.2052.

## 4. Single crystal X-ray diffraction studies

### X-ray Crystal Structure Determination of compound 21b

X-ray diffraction data of compound **21b** were measured on a Bruker D8 Quest Eco diffractometer using graphite-monochromated (Triumph) Mo K$\alpha$ radiation ($\lambda$ = 0.71073 Å) and a CPAD Photon III C14 detector. The sample was cooled with $N_2$ to 100 K with a Cryostream 700 (Oxford Cryosystems). Intensity data were integrated using the SAINT software.[2] Absorption correction and scaling was executed with SADABS.[3] The structures were solved using intrinsic phasing with the program SHELXT 2018/2[4] against $F^2$ of all reflections. Refinement in space group $P2_1/c$ was performed as an inversion twin, yielding BASF=0.48710, indicative of (near perfect) merohedral twinning (i.e., cocrystallisation of both enantiomers). Least-squares refinement was performed with SHELXL-2018/3.[5] All non-hydrogen atoms were refined with anisotropic displacement parameters. The hydrogen atoms were introduced at calculated positions with a riding model. The X-ray crystallographic data for **21b** was deposited at the Cambridge Crystallographic Data Centre (CCDC), under the deposition number CCDC 2212964.

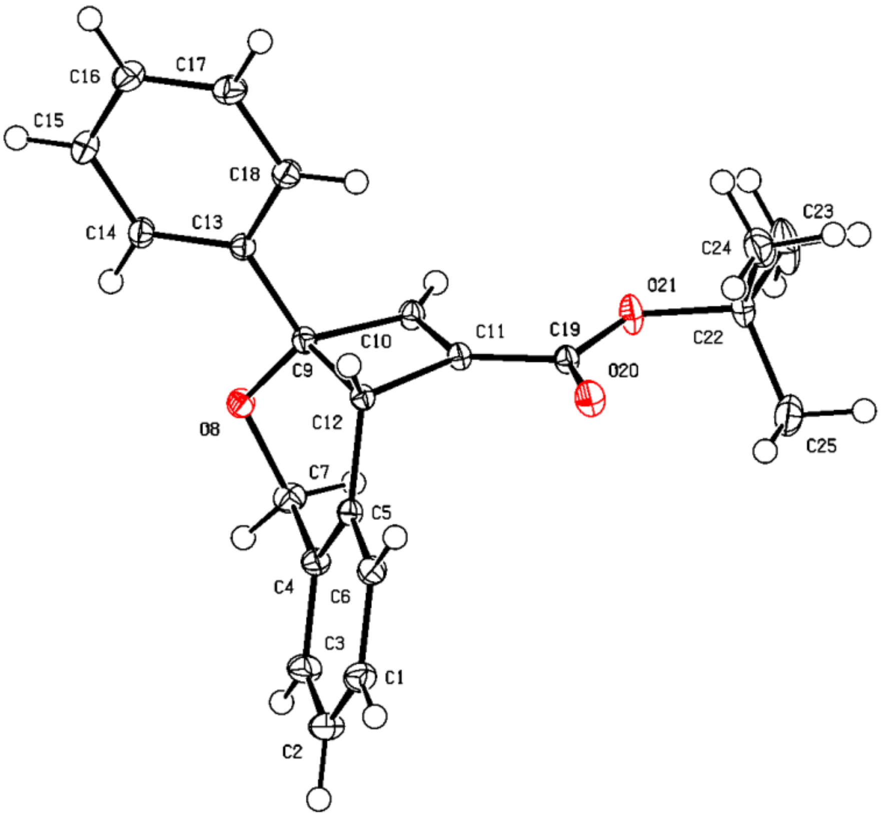


Figure S6. X-ray Crystal Structure of **21b**

Table S1. Bond distances of compounds **21b** as determined by X-ray diffraction studies.

| **Atom–Atom** | **Length [Å]** |
|---|---|
| C1–C2 | 1.3927(11) |
| C1–C6 | 1.3940(10) |
| C2–C3 | 1.3910(11) |
| C3–C4 | 1.3966(9) |
| C4–C5 | 1.3988(9) |
| C4–C7 | 1.5014(10) |
| C5–C6 | 1.3967(9) |
| C5–C12 | 1.5022(8) |
| C7–O8 | 1.4348(8) |
| O8–C9 | 1.4124(7) |
| C9–C13 | 1.5144(8) |
| C9–C10 | 1.5297(8) |
| C9–C12 | 1.5976(8) |
| C10–C11 | 1.3431(9) |
| C11–C19 | 1.4741(8) |
| C11–C12 | 1.5225(8) |
| C13–C14 | 1.3923(9) |
| C13–C18 | 1.3982(9) |
| C14–C15 | 1.3960(9) |
| C15–C16 | 1.3894(11) |
| C16–C17 | 1.3940(11) |
| C17–C18 | 1.3909(10) |
| C19–O20 | 1.2151(8) |
| C19–O21 | 1.3343(8) |
| O21–C22 | 1.4861(8) |
| C22–C23 | 1.5178(11) |
| C22–C25 | 1.5196(12) |
| C22–C24 | 1.5235(11) |

**21b**: $C_{22}H_{22}O_3$, Fw = 334.39, 0.303×0.268×0.217, monoclinic, *P* 21/*c*, (No: 14), a = 9.8817(5), b = 15.1066(8), c = 12.1195(6) Å, α = 90, β = 94.976(2), γ = 90°, V = 1802.37(16) $Å^3$, Z = 4, $D_x$ = 1.232 g/$cm^3$, μ = 0.081 $mm^{-1}$. 237584 Reflections were measured up to a resolution of $(\sin \theta/\lambda)_{max}$ = 0.52 $Å^{-1}$. 13344 Reflections were unique ($R_{int}$ = 0.0638), of which 9077 were observed [I>2σ(I)]. 229 Parameters were refined with 0 restraints. R1/wR2 [I > 2σ(I)]: 0.0548/0.1272. R1/wR2 [all refl.]: 0.0967/0.1477. S = 1.044. Residual electron density between -0.288 and 0.829 e/$Å^3$. CCDC 2212964.

## 5. Determination of physical and photophysical properties

### 5.1. UV/Vis spectra of the isomers

(1)

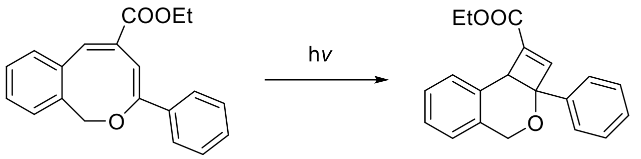


**1a** **1b** C = 6.5 × $10^{-5}$ M in $CH_2Cl_2$, 25°C

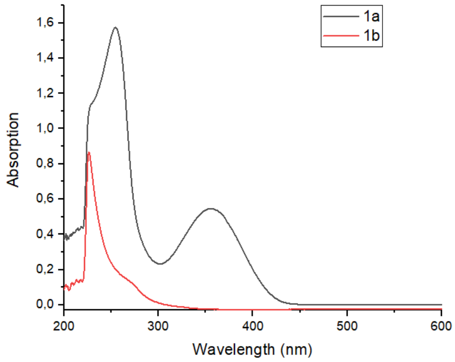


Figure S7. UV/Vis spectra of **1a**/**1b**

(2)

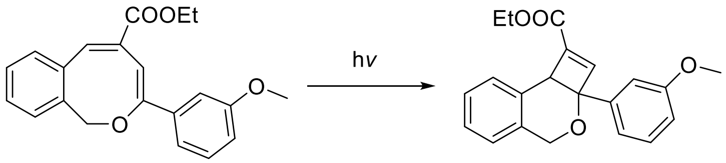


**3a** **3b** C = 4.8 × $10^{-4}$ M in $CH_2Cl_2$, 25°C

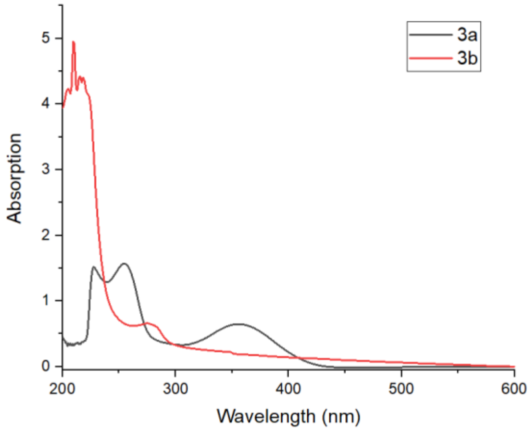


Figure S8. UV/Vis spectra of **3a**/**3b**

(3)

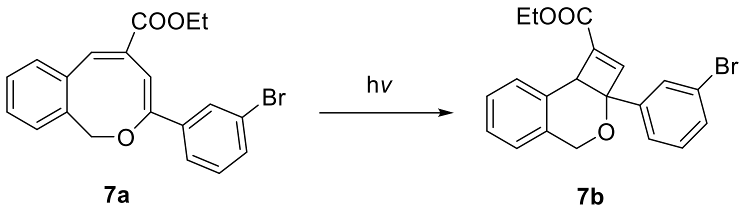


C = 2.2 × 10$^{-4}$ M in $CH_2Cl_2$, 25°C

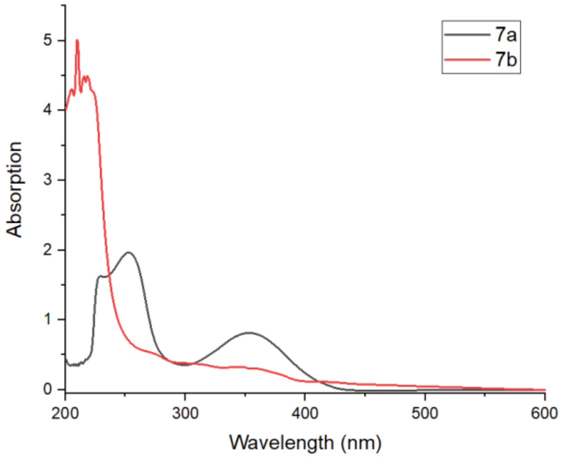


Figure S9. UV/Vis spectra of **7a**/**7b**

(4)

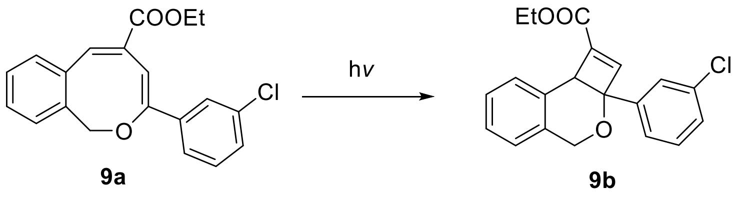


C = 1.8 × 10$^{-4}$ M in $CH_2Cl_2$, 25°C

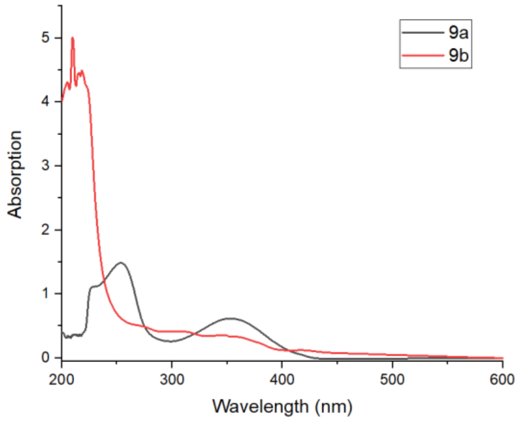


Figure S10. UV/Vis spectra of **9a**/**9b**

(5)

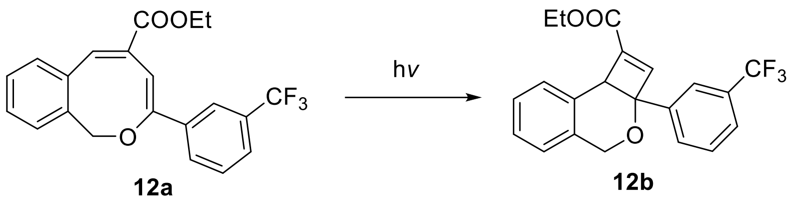


C = 1.5 × $10^{-4}$ M in $CH_2Cl_2$, 25°C

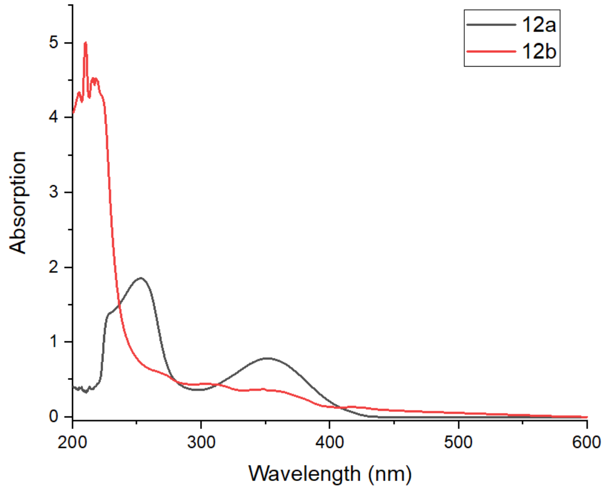


Figure S11. UV/Vis spectra of **12a**/**12b**

### 5.2. Determination of the quantum yield

The quantum yield (QY) of the conversion of 1*H*-2-benzo[*c*]oxocins to dihydro-4*H*-cyclobuta[*c*]isochromenes was determined via ferrioxalate actinometry, following a procedure reported in the literature.[6-8]

A 0.15 M solution of ferrioxalate was prepared by dissolving 2.21 g of potassium ferrioxalate hydrate in 30 mL of 0.05 M $H_2SO_4$. A buffered solution of phenanthroline was prepared by dissolving 50 mg of phenanthroline and 11.25 g of sodium acetate in 50 mL of 0.5 M $H_2SO_4$. Both solutions were stored in the dark. To determine the photon flux of the lamp, 2.0 mL of the ferrioxalate solution was placed in a cuvette and irradiated for 95 seconds with the same setup adopted for batch experiments. After irradiation, 0.35 mL of the phenanthroline solution was added to the cuvette. The solution was then allowed to rest for 0.5 h to allow the ferrous ions to completely coordinate to the phenanthroline. The absorbance of the solution was measured at 510 nm. A non-irradiated sample was also prepared and the absorbance at 510 nm measured. Conversion was calculated using the following eq. (1).

$$\text{mol } Fe^{2+} = (V*\Delta A)/(l*\varepsilon) \qquad (1)$$

Where V is the total volume (0.00235 L) of the solution after addition of phenanthroline, ΔA is the difference in absorbance at 510 nm between the irradiated and non-irradiated solutions, l is the path length (1.000 cm), and ε is the molar absorptivity at 510 nm (11100 L $mol^{-1}$ $cm^{-1}$). The photon flux can be calculated using eq. (2).

$$\text{Photon flux} = \text{mol } Fe^{2+}/(\phi*t*f) \qquad (2)$$

Where ϕ is the quantum yield for the ferrioxalate actinometer (1.216 for a 0.15 M solution at λ = 365 nm), t is the time (95 s), and f is the fraction of light absorbed at λ = 365 nm (0.9977, vide infra). The photon flux was calculated to be $2.95 \times 10^{-9}$ einstein $s^{-1}$.

$$\text{mol } Fe^{2+} = (2.35*0.001*1.6071)/(1.000*11100) = 3.40 \times 10^{-7} \text{ mol}$$

$$\text{Photon flux} = 3.40 \times 10^{-7}/(1.216*95*0.9977) = 2.95 \times 10^{-9} \text{ einstein s}^{-1}$$

The absorbance of the above ferrioxalate solution at 365 nm was measured to be 2.6345. The fraction of light absorbed (f) by this solution was calculated using eq. (3), where A is the measured absorbance at 365 nm.

$$f = 1 - 10^{-A} \qquad (3)$$

Quantum yields were calculated after 20 min of reaction (conversions were followed by $^{1}$H-NMR spectroscopy).

QY = (moles of conversion in 20 min)/(moles of photon reaching the reaction in 20 min)

Table S2. Quantum yield of **1a/1b**, **3a/3b**, **7a/7b**, **9a/9b** and **12a/12b**

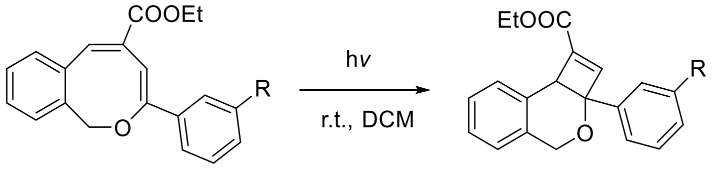


**a** **b**

| -**R** | Conversion in 20 min (mmol) | $\Phi_{RC}$ |
|---|---|---|
| -H | 0.001022734 | 0.29 |
| -OMe | 0.001568424 | 0.44 |
| -$CF_3$ | 0.000479059 | 0.14 |
| -Cl | 0.000697819 | 0.20 |
| -Br | 0.001531495 | 0.43 |

### 5.3. Optimization of reaction of thermal ring-opening reaction

Table S3. Optimization of reaction of thermal ring-opening reaction[a]

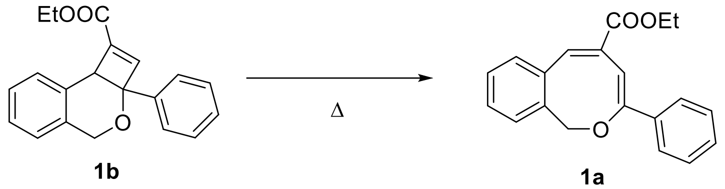


| Entry | T (°C) | Reaction time (h) | Solvent | Yield of **1a** (%)[b] |
|---|---|---|---|---|
| 1 | 60 | 48 | DMSO | 4 |
| 2 | 80 | 24 | DMSO | 10 |
| 3 | 115 | 15 | DMSO | 67 |
| 4 | 60 | 72 | toluene | 3 |
| 5 | 90 | 10 | toluene | 11 |
| 6 | 110 | 6 | toluene | 48 |
| 7 | 110 | 10 | toluene | 62 |
| 8 | 120 | 1.25 | *o*-xylene | 28 |
| 9 | 130 | 1.25 | *o*-xylene | 52 |
| 10 | 130 | 2 | *o*-xylene | 68 |

[a] General procedures: **1b** was dissolved in deuterated solvent in NMR tube with $N_2$ protection, heated the NMR tube in dark. [b] Yield was determined by integration of the $^1$H NMR signals in the presence of dimethyl sulfone as internal standard.

## 5.4. Kinetic study

(1) Ring-closure process

**1a** was dissolved in $CD_2Cl_2$ in NMR tube with $N_2$ protection, irradiated the NMR tube with white light at room temperature. Kinetics were followed by $^1$H-NMR spectroscopy.

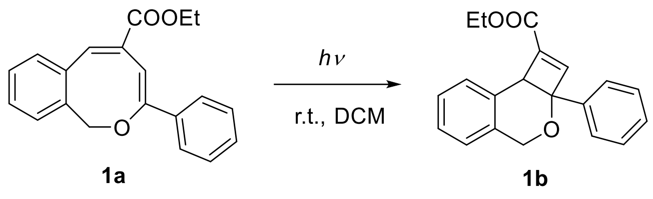


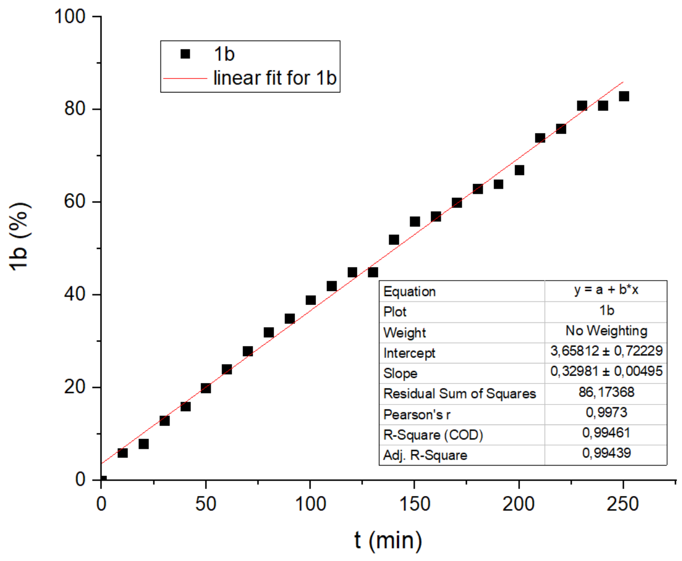


Figure S12. Kinetic study of photo-isomerization

(2) Ring-opening process

**1b** was dissolved in DMSO-$d^6$ in NMR tube with $N_2$ protection, heated the NMR tube at 115°C in dark. Kinetics were followed by $^1$H-NMR spectroscopy.

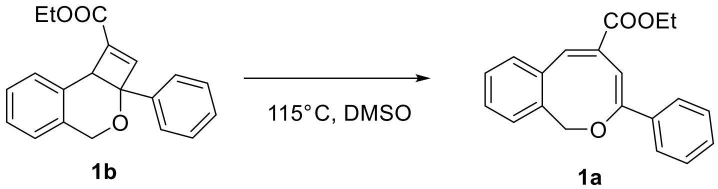


**1b** **1a**

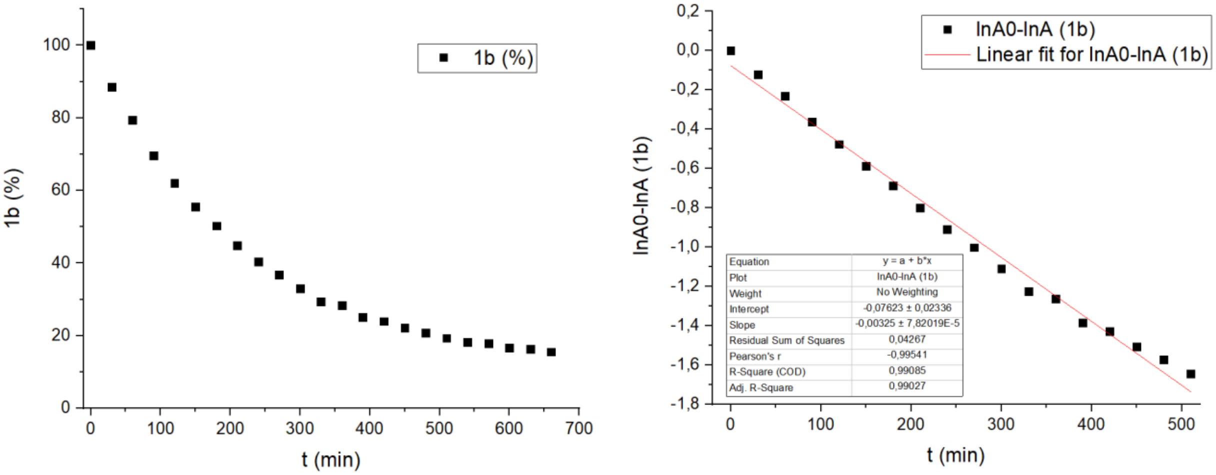


Figure S13. Kinetic study of thermal-isomerization

## 5.5. Determination of half-life times of the dihydro-4*H*-cyclobuta[*c*]isochromenes

Kinetics were followed by $^{1}H$-NMR spectroscopy, for which the kinetics at 25°C were extrapolated using an Eyring plot.

General procedures: dihydro-4*H*-cyclobuta[*c*]isochromenes were dissolved in *o*-xylene-$d^{10}$ in NMR tubes with $N_2$ protection, heated the NMR tubes at corresponding temperature in dark.

(1)

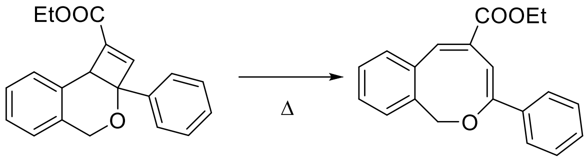


**1b** **1a**

$C = 2.7 \times 10^{-2}$ M

a. 90°C

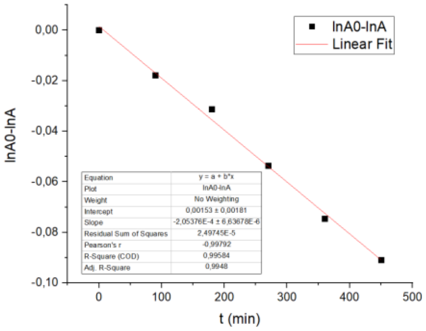


b. 100°C

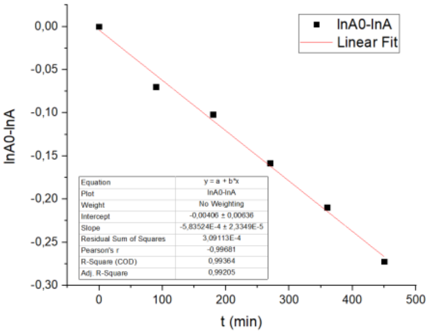


c. 110°C

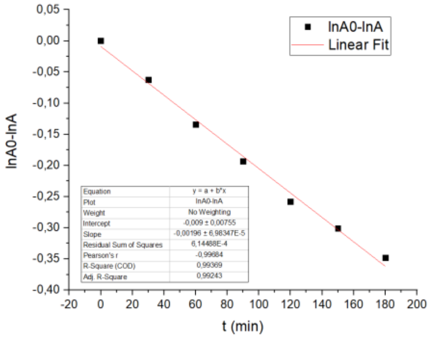


d. 120°C

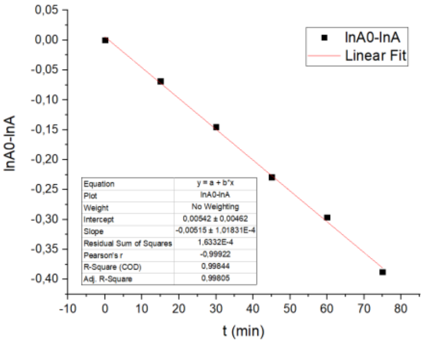


e. 130°C

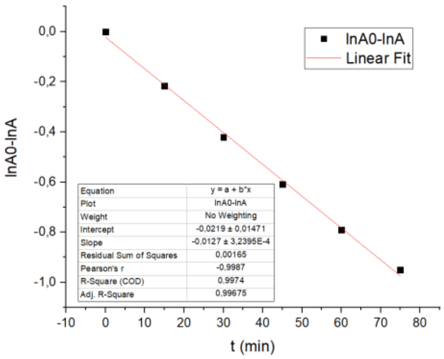


f. Eyring plot

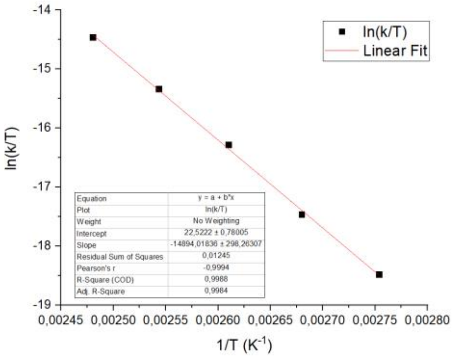


Figure S14. Thermal-isomerization of **1b** between 90–130°C

(2)

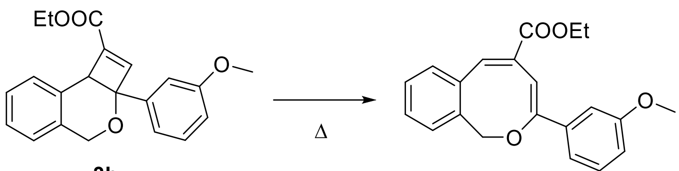


**3b** **3a**

$C = 2.0 \times 10^{-2}$ M

a. 90°C

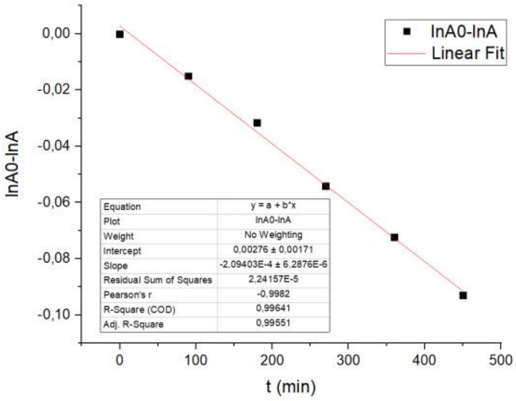


b. 100°C

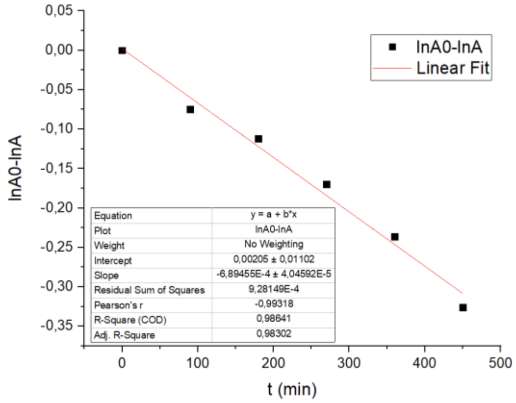


c. 110°C

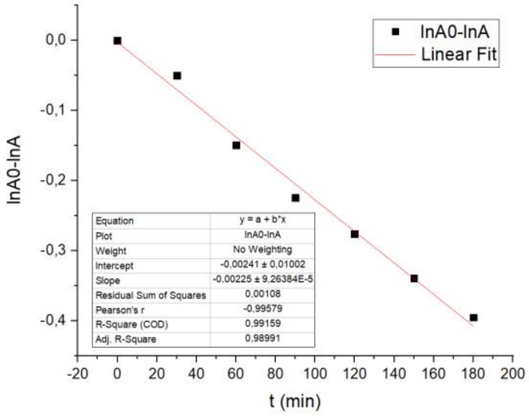


d. 120°C

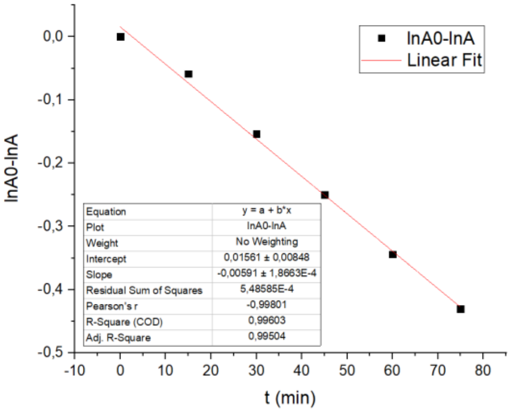


e. 130°C

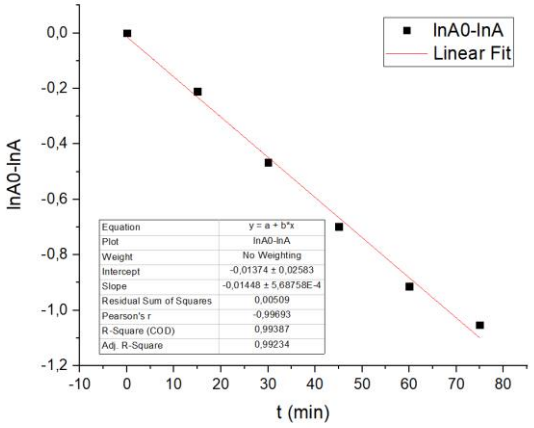


f. Eyring plot

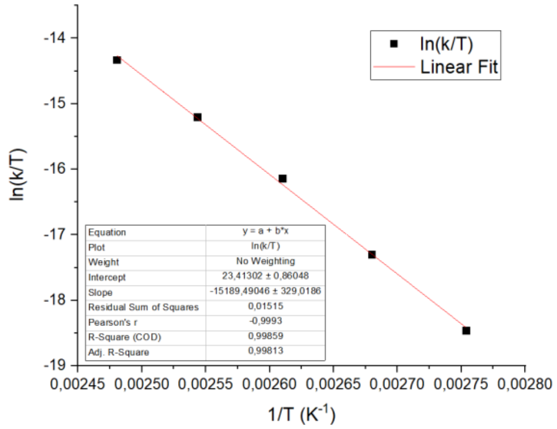


Figure S15. Thermal-isomerization of **3b** between 90–130°C

(3)

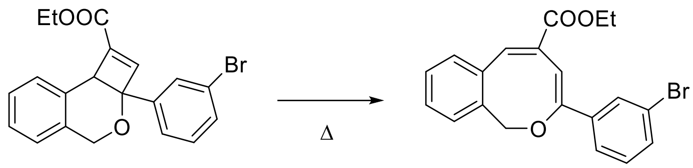


**7b** **7a**

C = 1.6 × $10^{-2}$ M

a. 90°C

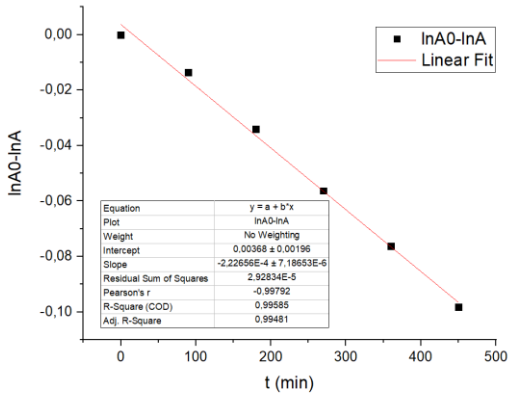


b. 100°C

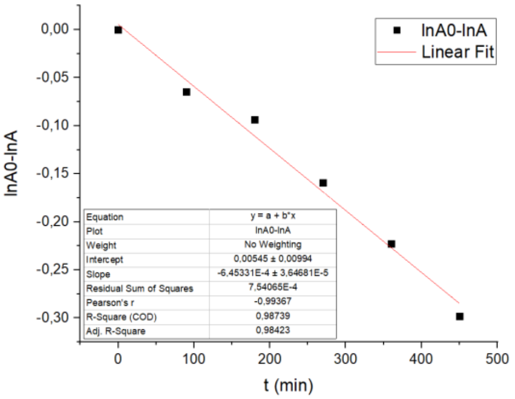


c. 110°C

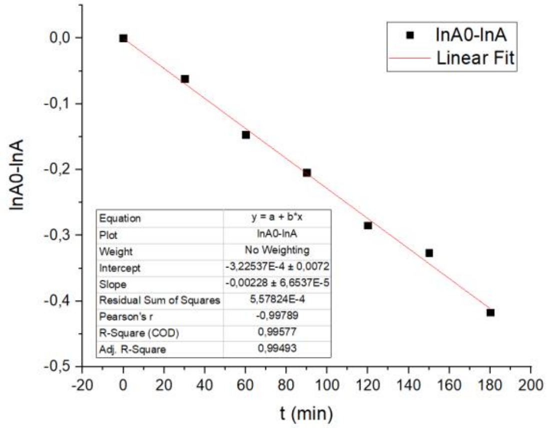


d. 120°C

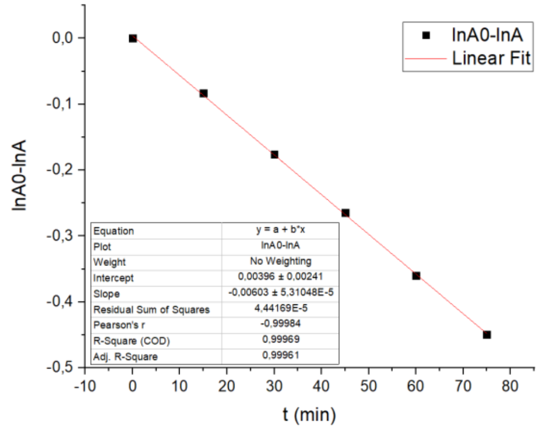


e. 130°C

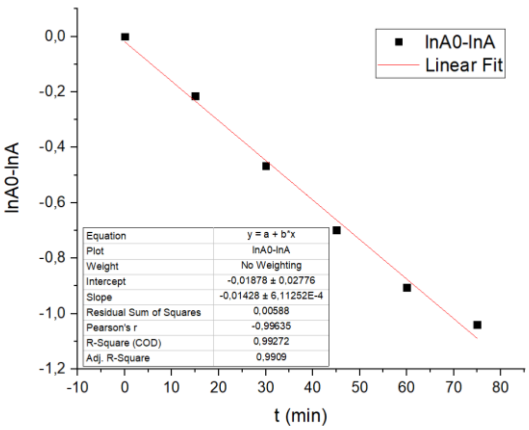


f. Eyring plot

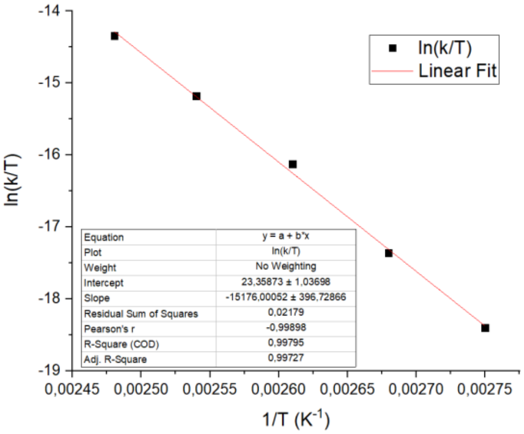


Figure S16. Thermal-isomerization of **7b** between 90–130°C

(4)

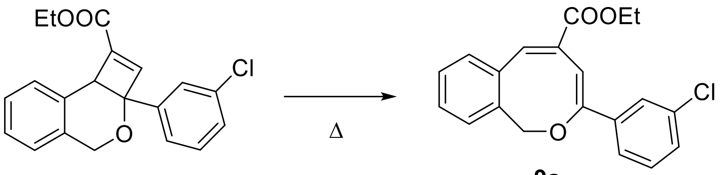


**9b** **9a**

C = 1.9 × 10$^{-2}$ M

a. 90°C

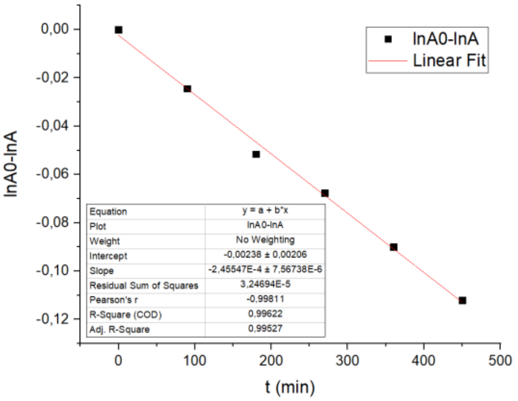


b. 100°C

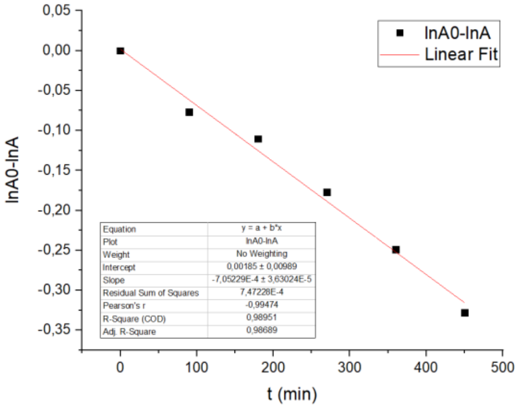


c. 110°C

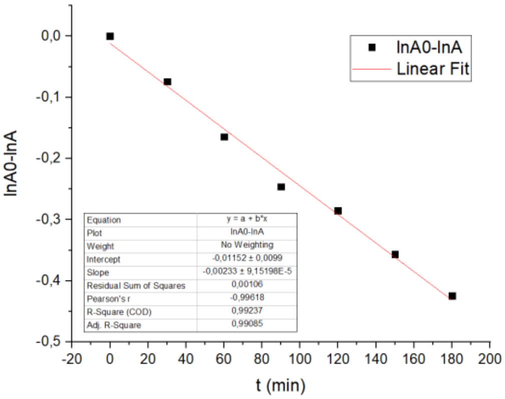


d. 120°C

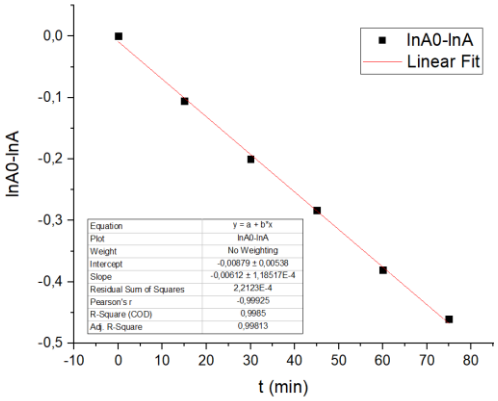


e. 130°C

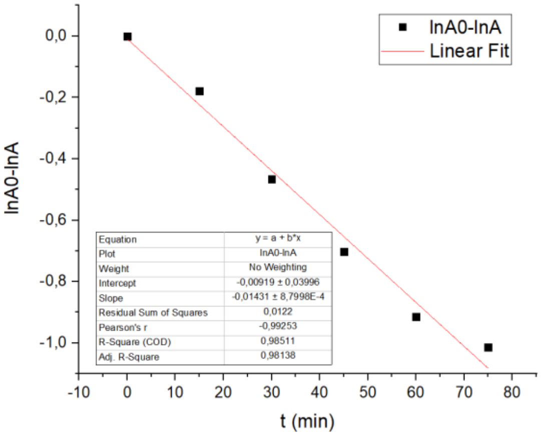


f. Eyring plot

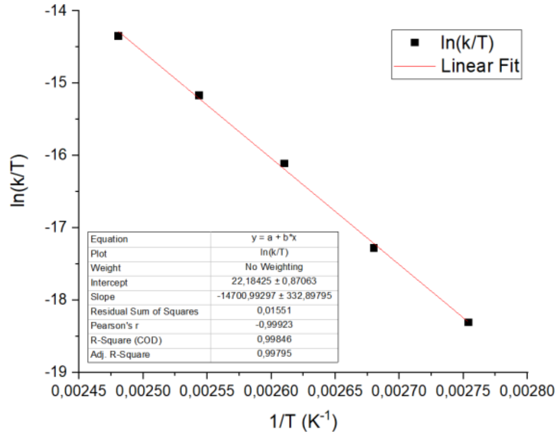


Figure S17. Thermal-isomerization of **9b** between 90–130°C

(5)

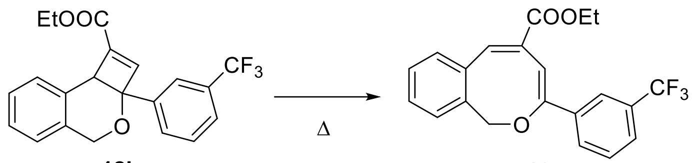


**12b** **12a**

C = 1.7 × $10^{-2}$ M

a. 90°C

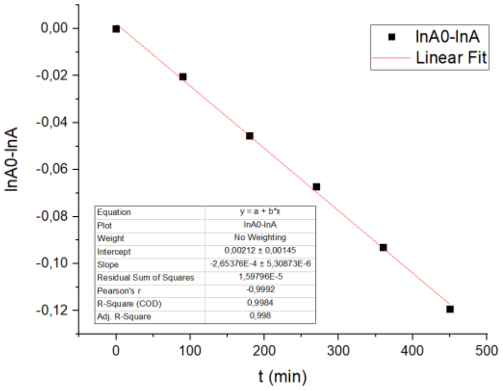


b. 100°C

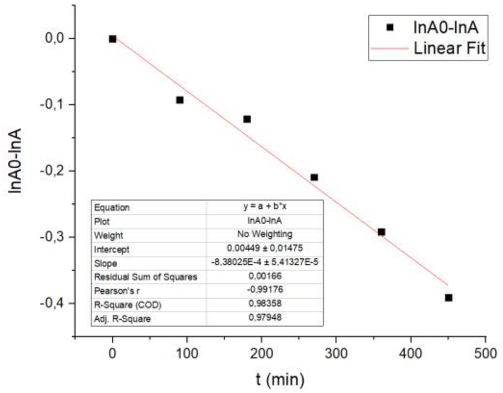


c. 110°C

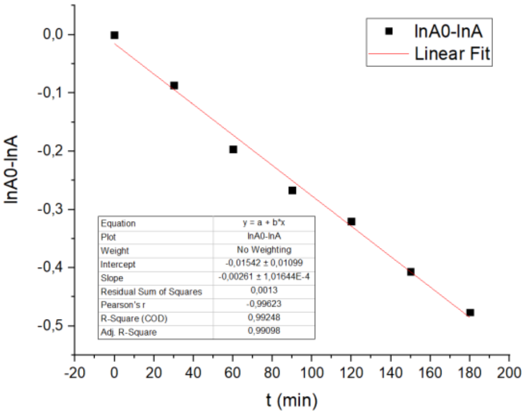


d. 120°C

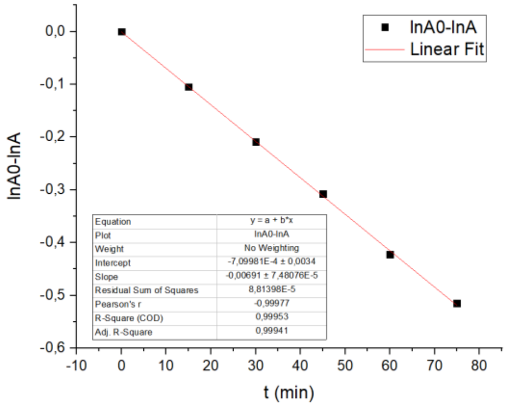


e. 130°C

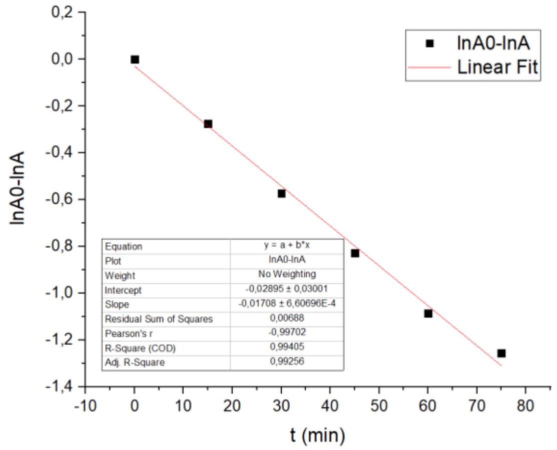


f. Eyring plot

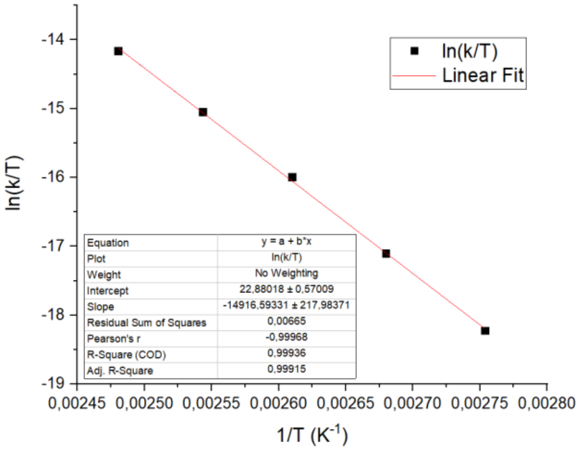


Figure S18. Thermal-isomerization of **12b** between 90–130°C

Table S4. Reaction rate constant and half-life time based on Eyring plot (thermal ring-opening of dihydro-4*H*-cyclobuta[*c*]isochromenes)

| **-R** | $k$ (at 298.15K) | $t_{1/2}$ (s) | $t_{1/2}$ (y) |
|---|---|---|---|
| -H | $3.6 \times 10^{-10}$ | $1.9 \times 10^{9}$ | 60.4 |
| -OMe | $3.3 \times 10^{-10}$ | $2.1 \times 10^{9}$ | 66.7 |
| $-CF_3$ | $4.8 \times 10^{-10}$ | $1.4 \times 10^{9}$ | 45.6 |
| -Cl | $5.0 \times 10^{-10}$ | $1.4 \times 10^{9}$ | 44.4 |
| -Br | $3.3 \times 10^{-10}$ | $2.1 \times 10^{9}$ | 67.4 |

Table S5. Eyring Activation Parameters (thermal ring-opening of dihydro-4*H*-cyclobuta[*c*]isochromenes**)**

| **-R** | $\Delta H^{\ddagger}$ [a] | $\Delta S^{\ddagger}$ [b] | $\Delta G^{\ddagger}_{298K}$ [a] | $\Delta G^{\ddagger}_{383K}$ [a] |
|---|---|---|---|---|
| -H | +29.6 | -2.5 | +30.3 | +30.5 |
| -OMe | +30.2 | -0.7 | +30.4 | +30.4 |
| $-CF_3$ | +29.6 | -1.7 | +30.1 | +30.3 |
| -Cl | +29.2 | -3.1 | +30.1 | +30.4 |
| -Br | +30.1 | -0.8 | +30.4 | +30.4 |

[a] kcal $mol^{-1}$; [b] cal $mol^{-1}$ $K^{-1}$

## 5.6. Solvent-dependence studies

Kinetics were followed by $^1$H-NMR spectroscopy.

General procedures: **1b** was dissolved in corresponding deuterated solvent in NMR tube with $N_2$ protection, heated the NMR tube at 110°C in dark.

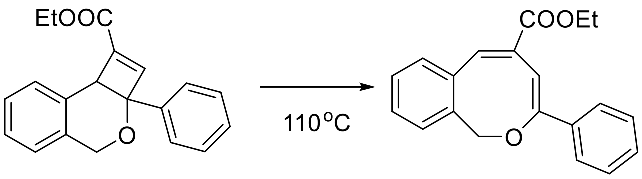


**1b** **1a**

$C = 1.8 \times 10^{-2}$ M

a. 110°C in toluene

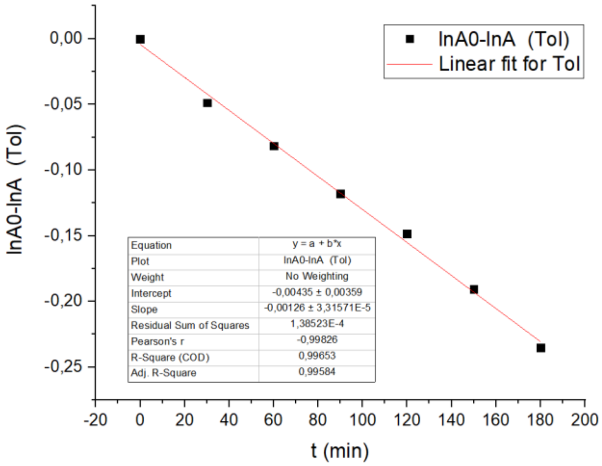


$k_{(toluene)} = 1.26 \times 10^{-3}\ s^{-1}$

b. 110°C in DMSO

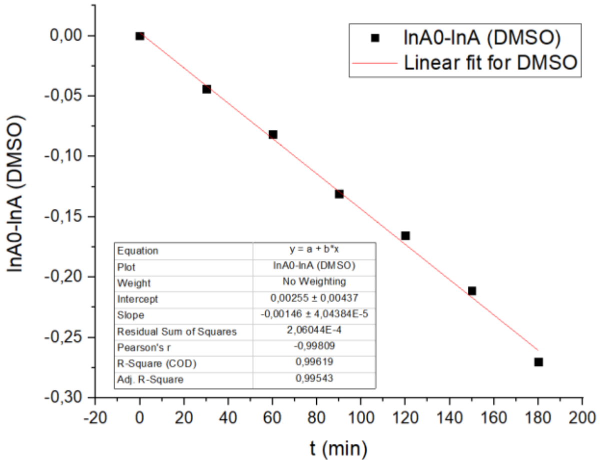


$k_{(DMSO)} = 1.46 \times 10^{-3}\ s^{-1}$

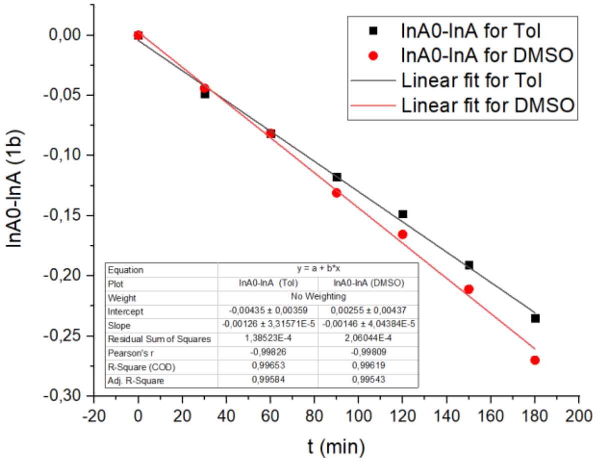


Figure S19. Thermal-isomerization of **12b** in toluene and DMSO

## 5.7. Switching cycles

Kinetics were followed by $^{1}$H-NMR spectroscopy. General procedures: Ring-closure: 1*H*-2-benzo[*c*]oxocins were dissolved in toluene-$d^8$ in NMR tubes with $N_2$ protection. The compounds were irradiated with white light for 4 h. Ring-opening: dihydro-4*H*-cyclobuta[*c*]isochromenes were dissolved in toluene-$d^8$ in NMR tubes with $N_2$ protection. The compounds were heated at 110°C for 10 h in dark.

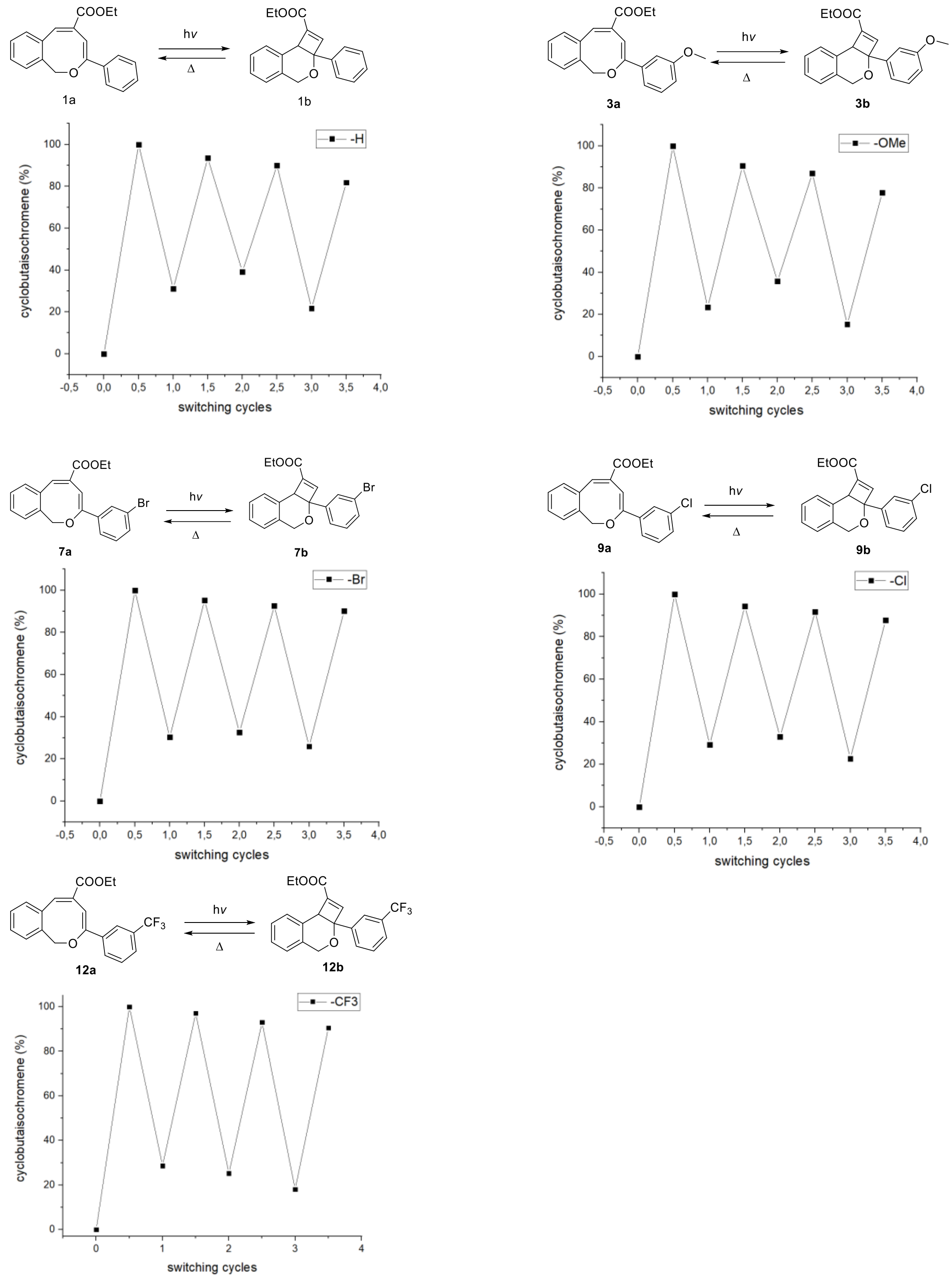


Figure S20. Switching cycles of **1a/1b**, **3a/3b**, **7a/7b**, **9a/9b** and **12a/12b**

Table S6. Fatigue of dihydro-4*H*-cyclobuta[*c*]isochromenes in each switching cycle

| **–R** | Fatigue in each switching cycle[a] |
|---|---|
| –OMe | 5.5% |
| –H | 4.5% |
| –Cl | 3% |
| –Br | 2.5% |
| $–CF_3$ | 2.3% |

[a] Average of four switching cycles.

## 6. Synthesis of dihydronaphalenes

### 6.1. General procedures of the synthesis of dihydronaphalenes

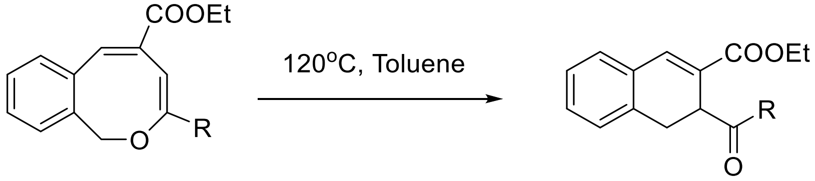


1*H*-2-benzo[*c*]oxocins (0.1 mmol) were dissolved in aqueous toluene (1.5 mL) in pressure tube with $N_2$ protection. The solution was heated to 120°C for 72 h.

### 6.2. Characterization of dihydronaphalenes

**Ethyl 3-benzoyl-3,4-dihydronaphthalene-2-carboxylate (1d)**

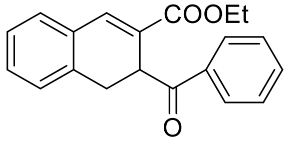


The product was purified by column chromatography (petroleum ether/ethyl acetate = 7:1) as yellow oil, 95% yield. $^{1}$H NMR (500 MHz, $CDCl_3$) δ 8.03 (d, *J* = 7.6 Hz, 2H), 7.81 (s, 1H), 7.56 (dt, *J* = 48.8, 7.9 Hz, 3H), 7.35 – 7.30 (m, 1H), 7.27 – 7.22 (m, 2H), 7.08 – 7.00 (m, 1H), 4.89 – 4.86 (m, 1H), 4.23 (q, *J* = 7.1 Hz, 2H), 3.42 – 3.29 (m, 1H), 3.17 (dd, *J* = 16.3, 5.3 Hz, 1H), 1.26 (d, *J* = 7.0 Hz, 3H). $^{13}$C NMR (125 MHz, $CDCl_3$) δ 199.34, 166.74, 138.77, 135.85, 133.71, 133.05, 131.87, 129.97, 128.95, 128.72, 128.64, 128.17, 127.78, 127.30, 60.87, 41.37, 31.91, 14.18. HRMS (FD, *m/z*): calculated for $C_{20}H_{18}O_3$: 306.1256, found: 306.1246.

**Ethyl 3-(2,3-dihydrobenzo[b][1,4]dioxine-6-carbonyl)-3,4-dihydronaphthalene-2-carboxylate (4d)**

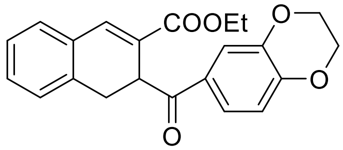


The product was purified by column chromatography (petroleum ether/ethyl acetate = 6:1) as yellow oil, 85% yield. $^{1}$H NMR (500 MHz, $CDCl_3$) δ 7.78 (s, 1H), 7.59 (dd, *J* = 6.5, 2.2 Hz, 3H), 7.23 (dd, *J* = 5.5, 3.4 Hz, 2H), 7.10 – 6.94 (m, 2H), 4.78 (dd, *J* = 8.9, 5.2 Hz, 1H), 4.36 – 4.30 (m, 4H), 4.22 (q, *J* = 7.2 Hz, 2H), 3.35 (dd, *J* = 16.3, 8.9 Hz, 1H), 3.16 (dd, *J* = 16.4, 5.3 Hz, 1H), 1.28 (td, *J* = 7.1, 3.4 Hz, 3H). $^{13}$C NMR (125 MHz, $CDCl_3$) δ 197.59, 166.75, 148.05, 143.45, 138.65, 133.82, 131.90, 129.88, 129.42, 128.89, 128.34, 127.75, 127.22, 122.78, 118.22, 117.35, 64.73, 64.13, 60.81, 41.04, 32.15, 14.19. HRMS (FD, *m/z*): calculated for $C_{22}H_{20}O_5$: 364.1311, found: 364.1307.

**Ethyl 3-(1-naphthoyl)-3,4-dihydronaphthalene-2-carboxylate (5d)**

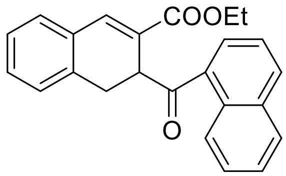


The product was purified by column chromatography (petroleum ether/ethyl acetate = 10:1) as yellow oil, 82% yield. $^{1}$H NMR (500 MHz, $CDCl_3$) δ 8.01 – 7.81 (m, 5H), 7.59 – 7.43 (m, 3H), 7.34 (d, *J* = 6.7 Hz, 1H), 7.27 (d, *J* = 10.8 Hz, 2H), 7.03 (d, *J* = 6.9 Hz, 1H), 4.77 (dt, *J* = 8.7, 3.9 Hz, 1H), 4.27 (dhept, *J* = 7.2, 3.8 Hz, 2H), 3.28 (dd, *J* = 16.5, 8.9 Hz, 1H), 3.16 (dt, *J* = 16.5, 3.9 Hz, 1H), 1.32 (t, *J* = 7.2 Hz, 3H). $^{13}$C NMR (125 MHz, $CDCl_3$) δ 203.35, 166.84, 138.77, 136.55, 133.95, 133.76, 132.08, 131.57, 130.05, 128.99, 128.27, 127.85, 127.45, 127.34, 126.44, 126.01, 125.40, 124.43, 60.94, 45.55, 31.05, 14.25. HRMS (FD, *m/z*): calculated for $C_{24}H_{20}O_3$: 356.1412, found: 356.1421.

**Ethyl 3-acetyl-3,4-dihydronaphthalene-2-carboxylate (13d)**

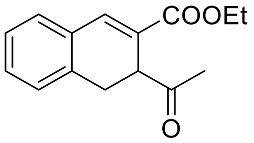

No further purification. Yellow oil, 95% yield. $^{1}H$ NMR (300 MHz, $CDCl_3$) δ 7.70 (s, 1H), 7.33 – 7.17 (m, 4H), 4.32 (q, *J* = 7.2 Hz, 2H), 3.82 (dd, *J* = 8.0, 3.5 Hz, 1H), 3.31 (dd, *J* = 16.3, 3.5 Hz, 1H), 3.15 (dd, *J* = 16.2, 8.1 Hz, 1H), 2.15 (s, 3H), 1.38 (t, *J* = 7.1 Hz, 3H). $^{13}C$ NMR (125 MHz, $CDCl_3$) δ 207.47, 166.86, 138.26, 134.92, 131.52, 130.22, 128.88, 127.94, 127.71, 127.16, 61.04, 46.13, 30.46, 28.22, 14.30. HRMS (FI, *m/z*): calculated for $C_{15}H_{16}O_3$: 244.1099, found: 244.1107.

**Ethyl 3-isobutyryl-3,4-dihydronaphthalene-2-carboxylate (15d)**

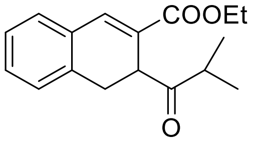

No further purification. Yellow oil, 95% yield. $^{1}H$ NMR (500 MHz, $CDCl_3$) δ 7.71 (s, 1H), 7.25 (p, *J* = 6.6 Hz, 3H), 7.15 (d, *J* = 7.0 Hz, 1H), 4.29 (tp, *J* = 7.2, 3.6 Hz, 2H), 4.03 (t, *J* = 6.3 Hz, 1H), 3.19 (d, *J* = 6.3 Hz, 2H), 2.94 (hept, *J* = 6.7 Hz, 1H), 1.36 (t, *J* = 7.0 Hz, 3H), 1.08 (d, *J* = 7.2 Hz, 6H). $^{13}C$ NMR (125 MHz, $CDCl_3$) δ 212.95, 166.82, 138.46, 134.61, 131.74, 131.37, 130.05, 128.79, 127.67, 127.15, 60.89, 44.46, 38.58, 31.03, 18.84, 18.46, 14.29. HRMS (FD, *m/z*): calculated for $C_{17}H_{20}O_3$: 272.1412, found: 272.1425.

**Ethyl 3-pivaloyl-3,4-dihydronaphthalene-2-carboxylate (16d)**

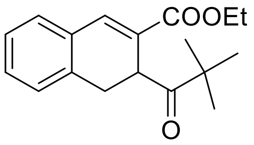

No further purification. Yellow oil, 92% yield. $^{1}H$ NMR (500 MHz, $CDCl_3$) δ 7.71 (s, 1H), 7.27 – 7.21 (m, 3H), 7.09 (d, *J* = 7.3 Hz, 1H), 4.40 (ddd, *J* = 7.6, 6.5, 1.0 Hz, 1H), 4.24 (q, *J* = 7.2 Hz, 2H), 3.21 (dd, *J* = 16.2, 7.9 Hz, 1H), 3.04 (dd, *J* = 16.2, 6.6 Hz, 1H), 1.34 (t, *J* = 7.1 Hz, 3H), 1.30 (s, 9H). $^{13}C$ NMR (125 MHz, $CDCl_3$) δ 214.16, 166.61, 138.83, 133.96, 132.06, 129.85, 129.23, 128.57, 127.52, 127.23, 60.76, 44.85, 40.99, 32.58, 27.39, 14.31. HRMS (FD, *m/z*): calculated for $C_{18}H_{22}O_3$: 286.1569, found: 286.1571.

**Ethyl 3-(cyclopropanecarbonyl)-3,4-dihydronaphthalene-2-carboxylate (17d)**

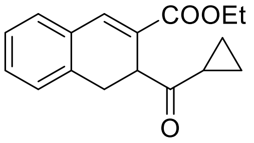

The product was purified by column chromatography (petroleum ether/ethyl acetate = 8:1) as yellow oil, 90% yield. $^{1}H$ NMR (500 MHz, $CDCl_3$) δ 7.83 (d, *J* = 2.6 Hz, 1H), 7.57 – 7.48 (m, 1H), 7.39 – 7.28 (m, 3H), 4.42 (p, *J* = 8.5, 7.8 Hz, 2H), 4.12 (d, *J* = 10.2 Hz, 1H), 3.45 (d, *J* = 16.2 Hz, 1H), 3.33 (dd, *J* = 16.3, 8.2 Hz, 1H), 2.18 (p, *J* = 7.6, 7.1 Hz, 1H), 1.48 (t, *J* = 7.2 Hz, 3H), 1.06 (tt, *J* = 4.3, 2.3 Hz, 2H), 1.04 – 0.87 (m, 2H). $^{13}C$ NMR (125 MHz, $CDCl_3$) δ 208.78, 166.91, 138.37, 134.94, 131.63, 130.11, 128.96, 128.84, 128.62, 127.79, 127.50, 127.05, 60.92, 46.29, 30.78, 18.72, 14.30, 11.05, 10.93. HRMS (FD, *m/z*): calculated for $C_{17}H_{18}O_3$: 270.1256, found: 270.1251.

**Ethyl 3-(cyclohexanecarbonyl)-3,4-dihydronaphthalene-2-carboxylate (18d)**

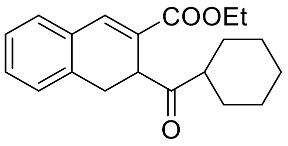

The product was purified by column chromatography (petroleum ether/ethyl acetate = 8:1) as yellow oil, 94% yield. $^{1}H$ NMR (500 MHz, $CDCl_3$) δ 7.70 (s, 1H), 7.25 (td, *J* = 14.0, 12.7, 7.0 Hz, 3H), 7.14 (d, *J* = 6.9 Hz, 1H), 4.28 (q, *J* = 7.2 Hz, 2H), 4.02 (t, *J* = 6.3 Hz, 1H), 3.18 (d, *J* = 6.3 Hz, 2H), 2.66 (td, *J* = 11.1, 3.2 Hz, 1H), 1.91 – 1.54 (m, 4H), 1.41 – 1.16 (m, 8H), 0.89 (q, *J* = 6.8 Hz, 1H).$^{13}C$ NMR (125 MHz, $CDCl_3$) δ 211.93, 166.84, 138.40, 134.59, 131.76, 130.00, 128.77, 127.96, 127.65, 127.11, 60.87, 48.88, 44.47, 30.93, 29.02, 28.56, 25.81, 25.78, 25.65, 14.31. HRMS (FD, *m/z*): calculated for $C_{20}H_{24}O_3$: 312.1725, found: 312.1730.

## 7. Computational details (DFT)

### 7.1. Computational studies for the thermal ring-opening reaction

The mechanism of the thermal ring-opening processes from dihydro-4*H*-cyclobuta[*c*]isochromenes **b** to 1*H*-2-benzo[*c*]oxocins **a** and dihydronaphthalene **d** were explored computationally using density functional theory (DFT). The results obtained are also described in the main text. All geometries were fully optimized as minima or transition states using the Turbomole program package[9] coupled to the PQS Baker optimizer[10] via the BOpt package.[11] We used unrestricted ri-DFT-D3 calculations at the B3LYP level,[12] in combination with the def2-TZVP basis set[13] and a small (m4) grid size. Grimme's dispersion corrections[14] (version 3, disp3, 'zero damping') were used to include Van der Waals interactions. All minima (no imaginary frequencies) and transition states (one imaginary frequency) were characterized by calculating the Hessian matrix. Thermochemical parameters such as the zero-point energy (ZPE), Gibbs free energy and gas-phase thermal corrections (entropy and enthalpy, 383 K, 1 bar) were obtained from these analyses. The nature of the transition states was confirmed by following the intrinsic reaction coordinate (IRC). The relative free energies ($\Delta G°_{383K}$ in kcal $mol^{-1}$) obtained from these calculations are reported in the main text. For every transition state, the imaginary eigenvalue was followed in both directions to confirm its connection to the relative reactant and product states. A separate archive file is provided, containing an Excel sheet with all free energies, enthalpies and entropies ($\Delta G°_{383K}$, $\Delta H°_{383K}$, $\Delta S°$, SCF+ZPE, SCF) and negative eigenvalues of the transition states and all optimized geometries. Optimized geometries of all stationary states and transition states are supplied in .pdb and .xyz format.

Scheme S1. Proposed mechanism for thermal ring-open from dihydro-4*H*-cyclobuta[*c*]isochromenes **b** to 1*H*-2-benzo[*c*]oxocins **a** and dihydronaphthalene **d**, based on DFT balculations (b3-lyp, def2-TZVP, m4 grid, disp3). All Gibbs free energies ($\Delta G^{o}_{383K}$ in kcal mol$^{-1}$), including **TS1–TS4**, are reported relative to the energy of intermediate **b**. The molecular structures belong to the ring-opening process with R = Ph. To reduce computational time a COOMe group was used instead of a COOEt group.

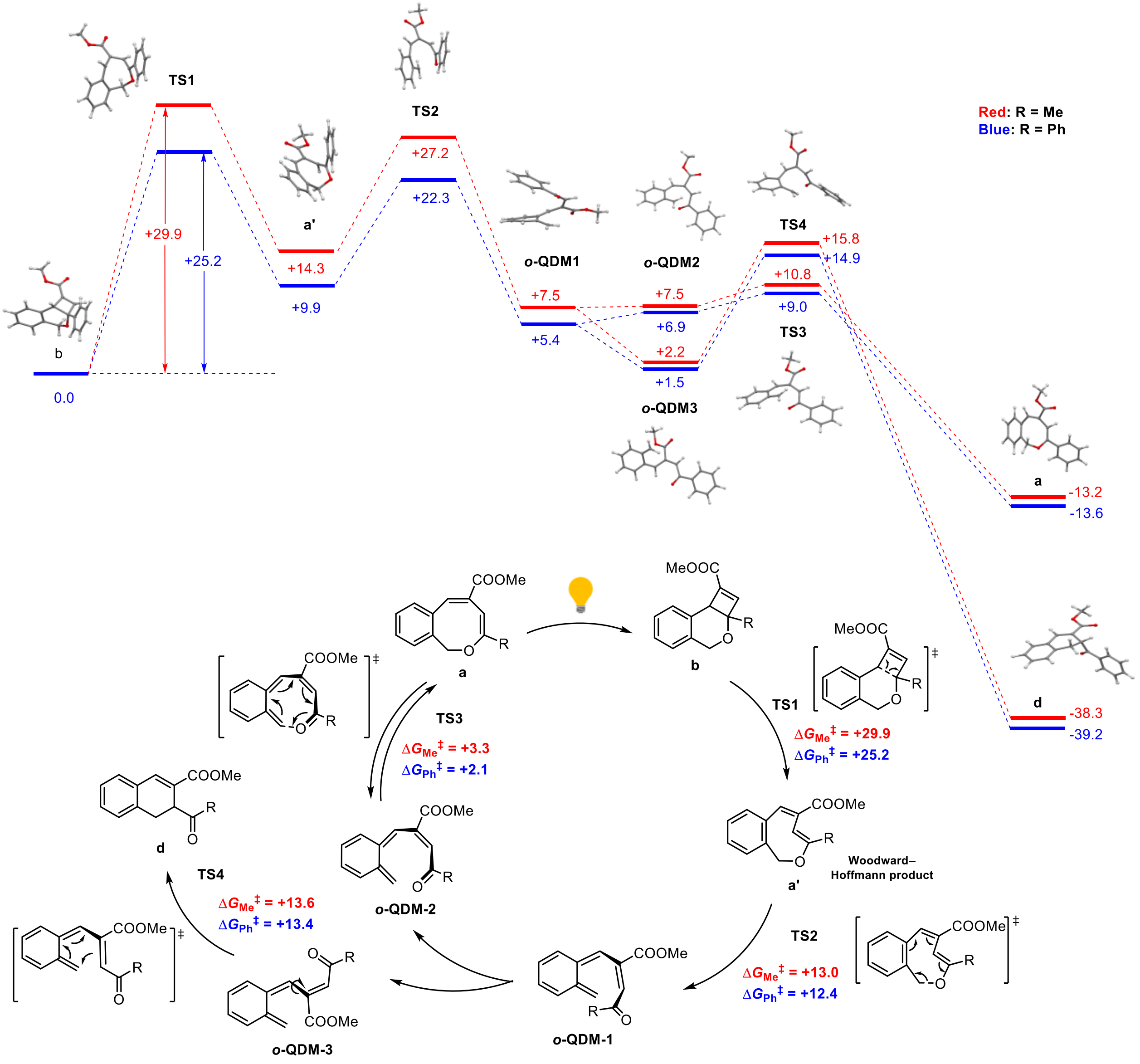

Scheme S2. Proposed mechanism for the thermal ring-contraction of 1*H*-2-benzo[*c*]oxocins **a** to dihydronaphthalenes **d**, based on DFT calculations (b3-lyp, def2-TZVP, m4 grid, disp3). All Gibbs free energies ($\Delta G^{o}_{383K}$ in kcal mol$^{-1}$), including **TS3** and **TS4**, are reported relative to the energy of intermediate **a**. To reduce computational time a COOMe group was used instead of a COOEt group.

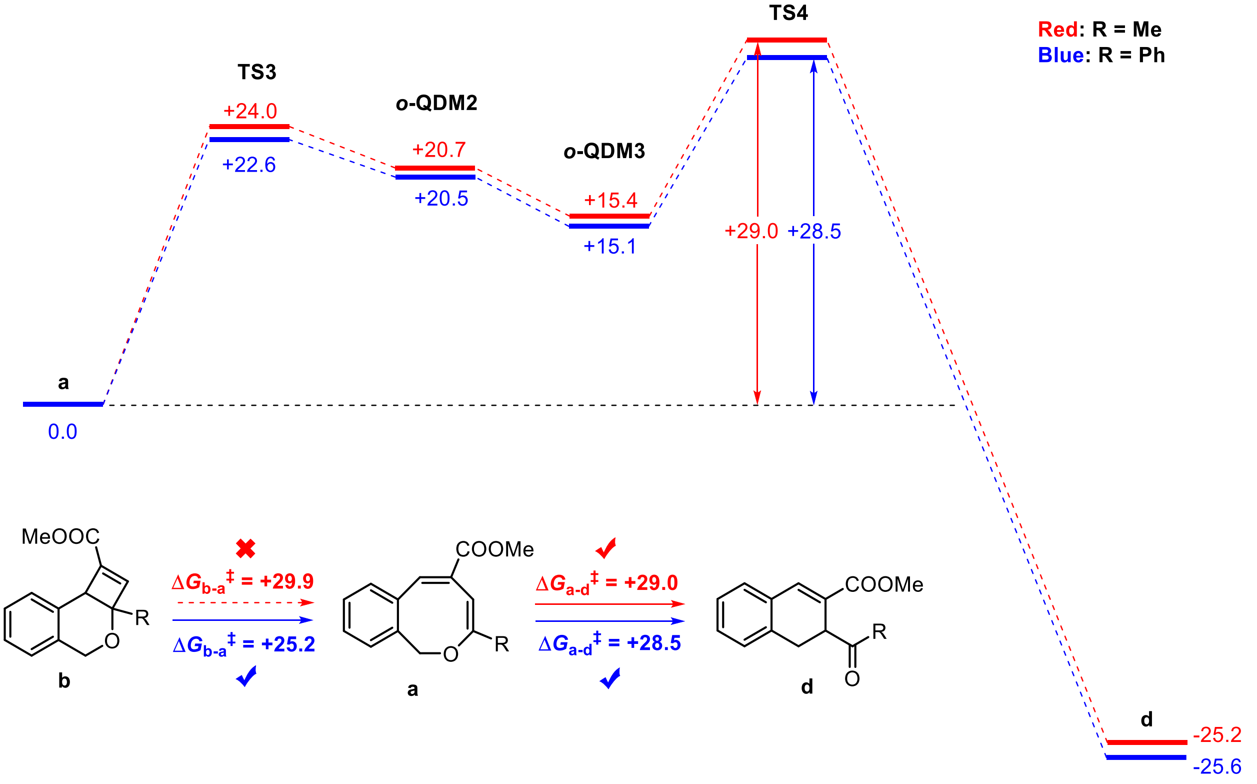

### 7.2. TD-DFT calculations

TD-DFT calculations were performed with Turbomole 7.6 (escf module, b3-lyp, def2-TZVP, m4 grid, COSMO $\varepsilon$ = 8.930, refractive index = 1.4244; 50 roots, RPA, singlet transitions), using the methyl ester analog of **1a** and **1b**.

The lowest energy transition of 1*H*-2-benzo[*c*]oxocin **1a**, calculated at 394 nm, corresponds to the HOMO (77a) $\rightarrow$ LUMO (78a) transition. This transition is essentially a singlet-to-singlet $\pi\rightarrow\pi^*$ transition, involving the enol ether -OC(Ph)=CH- carbon-carbon $\pi$-bonding donor orbital (HOMO) and the acrylate -C(COOR)=CH- carbon-carbon $\pi^*$-antibonding acceptor orbital (LUMO). See Figure S21.

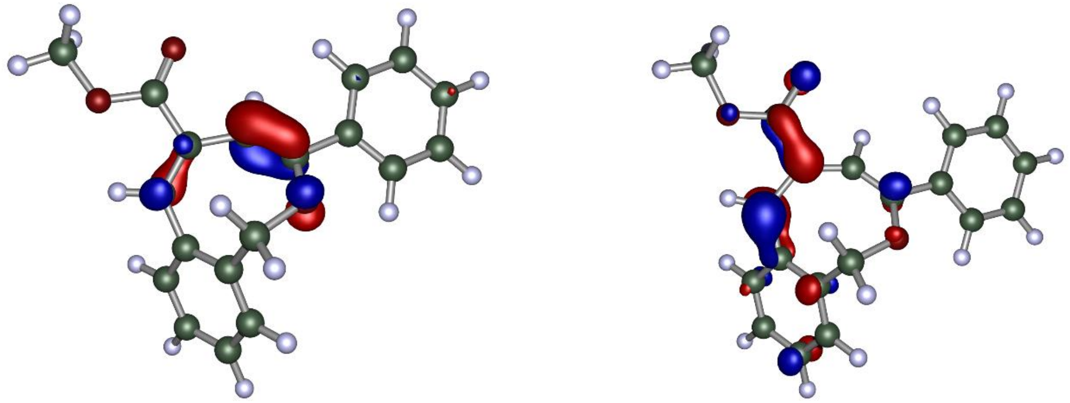

Figure S21. HOMO (enol ether C=C $\pi$-type orbital 77a; left) and LUMO (acrylate C=C $\pi^*$-type orbital 78a; right) of the methyl ester analogs of **1a**.

The computed UV/Vis spectra are shown in Figure S22. These agree qualitatively with the experimental spectra, confirming the disappearance of the tailing band at 394 nm, producing a colorless solution upon formation of the dihydro-4*H*-cyclobuta[*c*]isochromene **1b** (methyl ester analog).

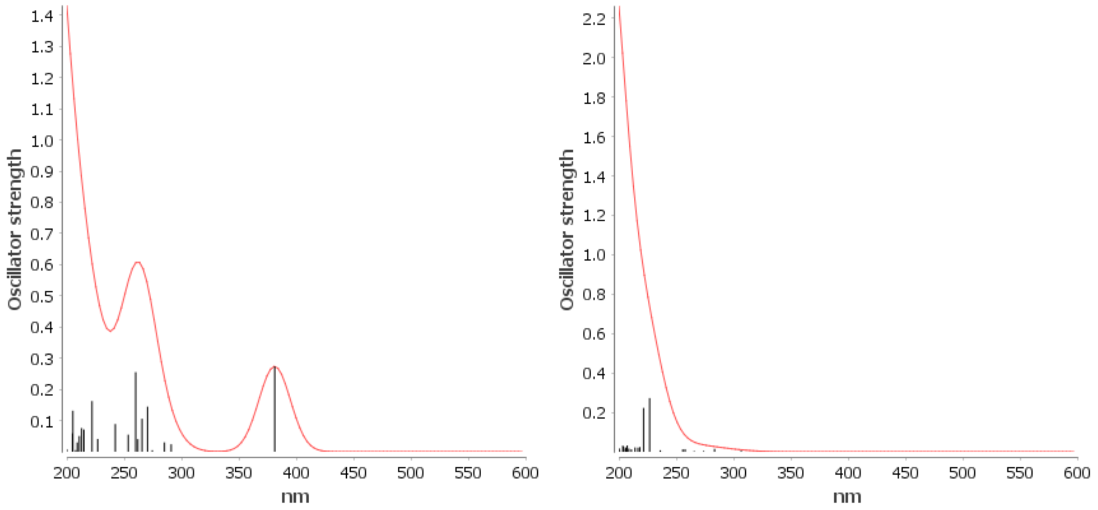


Figure S22. TD-DFT calculated UV/Vis spectra (b3-lyp, def2-TZVP) of the methyl ester analogs of 1*H*-2-benzo[c]oxocine **1a** (left) and dihydro-4*H*-cyclobuta[*c*]isochromene **1b** (right).

Additional TD-DFT calculations were performed with range-separated DFT (Turbomole 7.6, escf module, cam-b3lyp, aug-cc-pvdz, m5 grid, COSMO $\varepsilon$ = 8.930, refractive index = 1.4244; 50 roots, RPA, singlet transitions), using the methyl ester analogs of **1a** and **1b**, giving similar results (Figure S23).

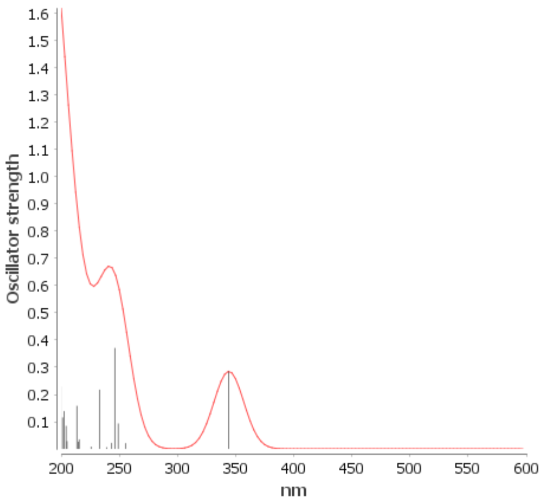


Figure S23. TD-DFT calculated UV/Vis spectra (cam-b3lyp, aug-cc-pvdz) of the methyl ester analogs of 1*H*-2-benzo[c]oxocine **1a** (left) and dihydro-4*H*-cyclobuta[*c*]isochromene **1b** (right).

TD-DFT calculations on **1a** were also performed with range-separated DFT and the larger def2-TZVPPD basis set (Turbomole 7.6, escf module, cam-b3lyp, aug-cc-pvdz, m5 grid, COSMO $\varepsilon$ = 8.930, refractive index = 1.4244; 50 roots, RPA, singlet transitions, using the methyl ester analogs of **1a** and **1b**). The results are shown in Figure S24.

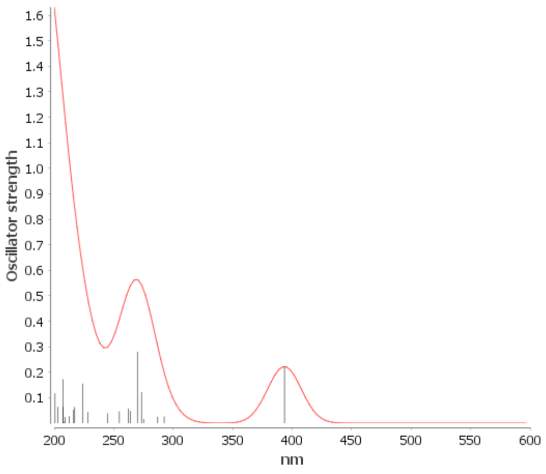


Figure S24. TD-DFT calculated UV/Vis spectra (cam-b3lyp, def2-TZVPPD) of the methyl ester analog of 1*H*-2-benzo[c]oxocine **1a** (left) and dihydro-4*H*-cyclobuta[*c*]isochromene **1b** (right).

## 7.3. Illustration of the ring-opening step

Based on DFT calculations, the twisted 8-membered ring **a’** has a helical conformation similar to that of ***o*-QDM-1**, which facilitates the ring-opening process. As shown in table S7, the transformations from **a’** to ***o*-QDM-1** followed by elongating the distance between C1 and O26 (the break of C–O bond), combined with shortening the distance between C25 and O26 (single bond becomes double bond).

Table S7. Bond length changes on going from **a’** via **TS2** to ***o*-QDM-1**.

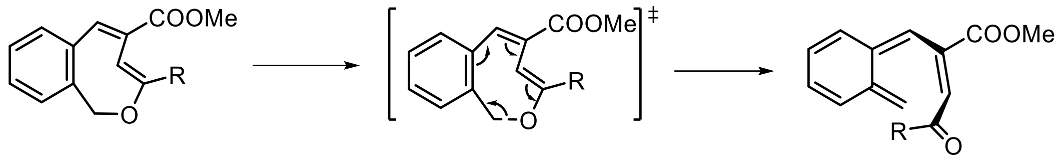


**a'** **TS2** ***o*-QDM-1**

**a’** (R = Ph)

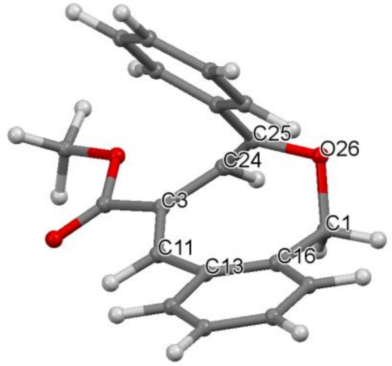


| Atom–Atom | Length [Å] |
|---|---|
| C1–O26 | 1.465 |
| O26–C25 | 1.375 |
| C25–C24 | 1.354 |
| C24–C3 | 1.455 |
| C3–C11 | 1.355 |
| C11–C13 | 1.479 |
| C13–C16 | 1.434 |
| C16–C1 | 1.522 |

**TS2** (R = Ph)

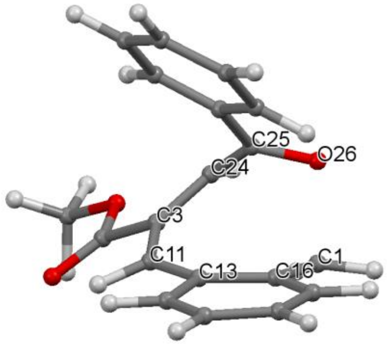


| Atom–Atom | Length [Å] |
|---|---|
| C1–O26 | 1.955 |
| O26–C25 | 1.280 |
| C25–C24 | 1.423 |
| C24–C3 | 1.392 |
| C3–C11 | 1.399 |
| C11–C13 | 1.427 |
| C13–C16 | 1.454 |
| C16–C1 | 1.411 |

***o*-QDM-1** (R = Ph)

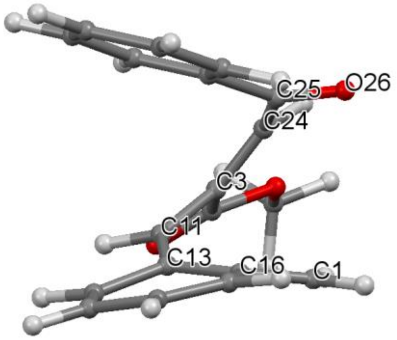


| Atom–Atom | Length [Å] |
|---|---|
| C1–O26 | 3.236 |
| O26–C25 | 1.220 |
| C25–C24 | 1.488 |
| C24–C3 | 1.349 |
| C3–C11 | 1.459 |
| C11–C13 | 1.361 |
| C13–C16 | 1.489 |
| C16–C1 | 1.346 |

## 7.4. DFT energy tables

Table S8. Free energies. enthalpies and entropies at 383 K (110°C).

**R** = Me

| ring_open_Me | **ΔG_383K** | **ΔH_383K** | **ΔS** |
|---|---|---|---|
| | **kcal mol$^{-1}$** | **kcal mol$^{-1}$** | **cal mol$^{-1}$ K$^{-1}$** |
| B3LYP. def2-TZVP. disp3. m4. 383 K | | | |
| **b_Me** | 0.0 | 0.0 | 0.0 |
| **TS1Δ_Me** | 29.9 | 29.4 | -1.1 |
| **a'_Me** | 14.3 | 14.5 | 0.7 |
| **TS2Δ_Me** | 27.2 | 27.8 | 1.4 |
| ***o*-QDM-1_Me** | 7.5 | 12.9 | 14.1 |
| **o-QDM-2_Me** | 7.5 | 13.2 | 15.0 |
| **o-QDM-3_Me** | 2.2 | 6.6 | 11.6 |
| **TS3Δ_Me** | 10.8 | 13.3 | 6.5 |
| **a_Me** | -13.2 | -12.0 | 2.9 |
| **TS4Δ_Me** | 15.8 | 18.9 | 8.0 |
| **d_Me** | -38.3 | -36.2 | 5.5 |

**R** = Ph

| ring_open_Ph | **ΔG_383K** | **ΔH_383K** | **ΔS** |
|---|---|---|---|
| | **kcal mol$^{-1}$** | **kcal mol$^{-1}$** | **cal mol$^{-1}$ K$^{-1}$** |
| B3LYP. def2-TZVP. disp3. m4. 383 K | | | |
| **b_Ph** | 0.0 | 0.0 | 0.0 |
| **TS1Δ_Ph** | 25.2 | 23.9 | -3.6 |
| **a'_Ph** | 9.9 | 8.8 | -2.9 |
| **TS2Δ_Ph** | 22.3 | 21.4 | -2.1 |
| **o-QDM-1_Ph** | 5.4 | 8.9 | 8.9 |
| **o-QDM-2_Ph** | 6.9 | 10.7 | 9.9 |
| **o-QDM-3_Ph** | 1.5 | 4.5 | 8.0 |
| **TS3Δ_Ph** | 9.0 | 10.3 | 3.4 |
| **a_Ph** | -13.6 | -13.7 | -0.2 |
| **TS4Δ_Ph** | 14.9 | 16.2 | 3.4 |
| **d_Ph** | -39.2 | -38.5 | 1.8 |

Table S9. Free energy barriers at 383 K (110°C).

**R** = Me

| ring_open_Me | G | neg. eigenv | | **ΔG_383K** | **barriers** |
|---|---|---|---|---|---|
| B3LYP. def2-TZVP. disp3. m4. 383 K | | | | **kcal mol$^{-1}$** | |
| **b_Me** | -767.22915 | | -481443.9639 | 0.0 | |
| **TS1Δ_Me** | -767.18157 | -346.45 cm$^{-1}$ | -481414.107 | 29.9 | 29.9 |
| **a'_Me** | -767.20644 | | -481429.7132 | 14.3 | |
| **TS2Δ_Me** | -767.18574 | -461.34 cm$^{-1}$ | -481416.7237 | 27.2 | 13.0 |
| **o-QDM-1_Me** | -767.21718 | | -481436.4526 | 7.5 | |
| **o-QDM-2_Me** | -767.21718 | | -481436.4526 | 7.5 | |
| **o-QDM-3_Me** | -767.22558 | | -481441.7237 | 2.2 | |
| **TS3Δ_Me** | -767.21187 | -129.25 cm$^{-1}$ | -481433.1205 | 10.8 | 3.3 |
| **a_Me** | -767.25011 | | -481457.1165 | -13.2 | |
| **TS4Δ_Me** | -767.20390 | -245.12 cm$^{-1}$ | -481428.1193 | 15.8 | 13.6 |
| **d_Me** | -767.29020 | | -481482.2734 | -38.3 | |

**R** = Ph

| ring_open_Ph | G | neg. eigenv | | **ΔG_383K** | **barriers** |
|---|---|---|---|---|---|
| B3LYP. def2-TZVP. disp3. m4. 383 K | | | | **kcal mol$^{-1}$** | |
| **b_Ph** | -958.87566 | | -601704.0654 | 0.0 | |
| **TS1Δ_Ph** | -958.83544 | -316.75 cm-1 | -601678.827 | 25.2 | 25.2 |
| **a'_Ph** | -958.85992 | | -601694.1884 | 9.9 | |
| **TS2Δ_Ph** | -958.84020 | -443.79 cm-1 | -601681.8139 | 22.3 | 12.4 |
| **o-QDM-1_Ph** | -958.86699 | | -601698.6249 | 5.4 | |
| **o-QDM-2_Ph** | -958.86466 | | -601697.1628 | 6.9 | |
| **o-QDM-3_Ph** | -958.87332 | | -601702.597 | 1.5 | |
| **TS3Δ_Ph** | -958.86131 | -101.01 cm-1 | -601695.0606 | 9.0 | 2.1 |
| **a_Ph** | -958.89738 | | -601717.6949 | -13.6 | |
| **TS4Δ_Ph** | -958.85198 | -247.24 cm-1 | -601689.206 | 14.9 | 13.4 |
| **d_Ph** | -958.93811 | | -601743.2534 | -39.2 | |

Table S10. Enthalpy barriers at 383 K (110°C).

R = Me

| | H_383K | SCF | ZPE_corr | H_corr | **ΔH_383K** |
|---|---|---|---|---|---|
| ring_open_Me | | | | | **kcal mol$^{-1}$** |
| B3LYP. def2-TZVP. disp3. m4. 383 K | | | | | |
| **b_Me** | -767.14442 | -767.41910 | 0.24986 | 0.27468 | 0.0 |
| **TS1Δ_Me** | -767.09751 | -767.36998 | 0.24785 | 0.27247 | 29.4 |
| **a'_Me** | -767.12127 | -767.39593 | 0.24949 | 0.27466 | 14.5 |
| **TS2Δ_Me** | -767.10017 | -767.37159 | 0.24607 | 0.27142 | 27.8 |
| **o-QDM-1_Me** | -767.12385 | -767.39672 | 0.24537 | 0.27287 | 12.9 |
| **o-QDM-2_Me** | -767.12332 | -767.39597 | 0.24511 | 0.27265 | 13.2 |
| **o-QDM-3_Me** | -767.13386 | -767.40669 | 0.24553 | 0.27283 | 6.6 |
| **TS3Δ_Me** | -767.12319 | -767.39512 | 0.24588 | 0.27193 | 13.3 |
| **a_Me** | -767.16358 | -767.43918 | 0.25027 | 0.27560 | -12.0 |
| **TS4Δ_Me** | -767.11426 | -767.38577 | 0.24529 | 0.27151 | 18.9 |
| **d_Me** | -767.20209 | -767.47723 | 0.24938 | 0.27514 | -36.2 |

R = Ph

| | H_383K | SCF | ZPE_corr | H_corr | **ΔH_383K** |
|---|---|---|---|---|---|
| ring_open_Ph | | | | | **kcal mol$^{-1}$** |
| B3LYP. def2-TZVP. disp3. m4. 383 K | | | | | |
| **b_Ph** | -958.77623 | -959.10907 | 0.30260 | 0.33284 | 0.0 |
| **TS1Δ_Ph** | -958.73818 | -959.06874 | 0.30056 | 0.33056 | 23.9 |
| **a'_Ph** | -958.76224 | -959.09516 | 0.30258 | 0.33292 | 8.8 |
| **TS2Δ_Ph** | -958.74205 | -959.07183 | 0.29922 | 0.32978 | 21.4 |
| **o-QDM-1_Ph** | -958.76212 | -959.09337 | 0.29876 | 0.33125 | 8.9 |
| **o-QDM-2_Ph** | -958.75916 | -959.09074 | 0.29933 | 0.33158 | 10.7 |
| **o-QDM-3_Ph** | -958.76900 | -959.10050 | 0.29925 | 0.33150 | 4.5 |
| **TS3Δ_Ph** | -958.75983 | -959.09031 | 0.29946 | 0.33048 | 10.3 |
| **a_Ph** | -958.79805 | -959.13187 | 0.30328 | 0.33382 | -13.7 |
| **TS4Δ_Ph** | -958.75048 | -959.08058 | 0.29896 | 0.33010 | 16.2 |
| **d_Ph** | -958.83756 | -959.17131 | 0.30301 | 0.33375 | -38.5 |

Table S11. SCF+ZPE barriers.

R = Me

| | SCF+ZPE | SCF | ZPE_corr | H_corr | **Δ (SCF+ZPE)** |
|---|---|---|---|---|---|
| ring_open_Me | | | | | **kcal mol$^{-1}$** |
| B3LYP. def2-TZVP. disp3. m4. 383 K | | | | | |
| **b_Me** | -767.16924 | -767.41910 | 0.24986 | 0.27468 | 0.0 |
| **TS1Δ_Me** | -767.12213 | -767.36998 | 0.24785 | 0.27247 | 29.6 |
| **a'_Me** | -767.14644 | -767.39593 | 0.24949 | 0.27466 | 14.3 |
| **TS2Δ_Me** | -767.12552 | -767.37159 | 0.24607 | 0.27142 | 27.4 |
| **o-QDM-1_Me** | -767.15135 | -767.39672 | 0.24537 | 0.27287 | 11.2 |
| **o-QDM-2_Me** | -767.15086 | -767.39597 | 0.24511 | 0.27265 | 11.5 |
| **o-QDM-3_Me** | -767.16116 | -767.40669 | 0.24553 | 0.27283 | 5.1 |
| **TS3Δ_Me** | -767.14924 | -767.39512 | 0.24588 | 0.27193 | 12.6 |
| **a_Me** | -767.18891 | -767.43918 | 0.25027 | 0.27560 | -12.3 |
| **TS4Δ_Me** | -767.14048 | -767.38577 | 0.24529 | 0.27151 | 18.0 |
| **d_Me** | -767.22785 | -767.47723 | 0.24938 | 0.27514 | -36.8 |

R = Ph

| | SCF+ZPE | SCF | ZPE_corr | H_corr | **Δ (SCF+ZPE)** |
|---|---|---|---|---|---|
| ring_open_Ph | | | | | **kcal mol$^{-1}$** |
| B3LYP. def2-TZVP. disp3. m4. 383 K | | | | | |
| **b_Ph** | -958.80647 | -959.10907 | 0.30260 | 0.33284 | 0.0 |
| **TS1Δ_Ph** | -958.76818 | -959.06874 | 0.30056 | 0.33056 | 24.0 |
| **a'_Ph** | -958.79258 | -959.09516 | 0.30258 | 0.33292 | 8.7 |
| **TS2Δ_Ph** | -958.77261 | -959.07183 | 0.29922 | 0.32978 | 21.2 |
| **o-QDM-1_Ph** | -958.79461 | -959.09337 | 0.29876 | 0.33125 | 7.4 |
| **o-QDM-2_Ph** | -958.79141 | -959.09074 | 0.29933 | 0.33158 | 9.5 |
| **o-QDM-3_Ph** | -958.80125 | -959.10050 | 0.29925 | 0.33150 | 3.3 |
| **TS3Δ_Ph** | -958.79085 | -959.09031 | 0.29946 | 0.33048 | 9.8 |
| **a_Ph** | -958.82859 | -959.13187 | 0.30328 | 0.33382 | -13.9 |
| **TS4Δ_Ph** | -958.78162 | -959.08058 | 0.29896 | 0.33010 | 15.6 |
| **d_Ph** | -958.86830 | -959.17131 | 0.30301 | 0.33375 | -38.8 |

**7.5. Optimized geometries**

**7.4.1. Calculations to structures with R = Me**

**a_Me**

31

| | | | |
|---|---|---|---|
| C | 3.584000 | -2.296000 | 0.325000 |
| H | 2.861000 | -1.699000 | -0.234000 |
| C | 4.311000 | 0.090000 | -1.521000 |
| C | 3.850000 | 1.313000 | -2.271000 |
| O | 3.946000 | 2.444000 | -1.857000 |
| O | 3.303000 | 1.032000 | -3.474000 |
| C | 2.885000 | 2.169000 | -4.245000 |
| H | 3.732000 | 2.822000 | -4.457000 |
| H | 2.128000 | 2.739000 | -3.706000 |
| H | 2.475000 | 1.762000 | -5.166000 |
| C | 4.489000 | -1.044000 | -2.234000 |
| H | 3.042000 | -3.058000 | 0.884000 |
| C | 4.991000 | -2.341000 | -1.785000 |
| C | 6.024000 | -4.844000 | -1.051000 |
| C | 5.873000 | -3.037000 | -2.627000 |
| C | 4.592000 | -2.944000 | -0.582000 |
| C | 5.116000 | -4.188000 | -0.232000 |
| C | 6.398000 | -4.265000 | -2.260000 |
| H | 6.155000 | -2.590000 | -3.573000 |
| H | 4.798000 | -4.649000 | 0.697000 |
| H | 7.092000 | -4.774000 | -2.917000 |
| H | 6.424000 | -5.806000 | -0.757000 |
| H | 4.279000 | -0.985000 | -3.295000 |
| C | 4.676000 | 0.403000 | -0.136000 |
| H | 4.997000 | 1.432000 | -0.039000 |
| C | 4.652000 | -0.250000 | 1.042000 |
| C | 5.137000 | 0.395000 | 2.306000 |
| H | 5.508000 | 1.402000 | 2.126000 |
| H | 4.327000 | 0.437000 | 3.039000 |
| H | 5.937000 | -0.207000 | 2.744000 |
| O | 4.193000 | -1.488000 | 1.343000 |

**a’_Me**

31

| | | | |
|---|---|---|---|
| C | 4.064000 | -1.072000 | 0.118000 |
| H | 4.490000 | -0.075000 | 0.011000 |
| C | 3.544000 | 0.240000 | -3.095000 |
| C | 3.457000 | 1.239000 | -4.196000 |

| | | | |
|---|---|---|---|
| O | 4.056000 | 1.194000 | -5.246000 |
| O | 2.606000 | 2.236000 | -3.875000 |
| C | 2.456000 | 3.267000 | -4.862000 |
| H | 1.751000 | 3.977000 | -4.439000 |
| H | 2.071000 | 2.851000 | -5.794000 |
| H | 3.414000 | 3.748000 | -5.063000 |
| C | 4.448000 | -0.761000 | -3.213000 |
| H | 4.271000 | -1.424000 | 1.127000 |
| C | 4.848000 | -1.862000 | -2.305000 |
| C | 5.752000 | -4.245000 | -1.046000 |
| C | 5.501000 | -2.898000 | -3.003000 |
| C | 4.676000 | -2.046000 | -0.890000 |
| C | 5.127000 | -3.238000 | -0.321000 |
| C | 5.944000 | -4.067000 | -2.405000 |
| H | 5.652000 | -2.776000 | -4.068000 |
| H | 4.987000 | -3.379000 | 0.744000 |
| H | 6.434000 | -4.826000 | -3.001000 |
| H | 6.085000 | -5.147000 | -0.548000 |
| H | 4.968000 | -0.792000 | -4.165000 |
| C | 2.830000 | 0.365000 | -1.832000 |
| C | 2.302000 | -0.736000 | -1.273000 |
| C | 1.580000 | -1.847000 | -1.953000 |
| H | 1.379000 | -1.614000 | -2.997000 |
| H | 2.140000 | -2.783000 | -1.881000 |
| H | 0.627000 | -2.005000 | -1.438000 |
| O | 2.606000 | -0.994000 | 0.045000 |
| H | 3.137000 | 1.174000 | -1.177000 |

**b_Me**

31

| | | | |
|---|---|---|---|
| C | 4.871000 | -0.480000 | -0.826000 |
| C | 4.837000 | -1.957000 | -1.087000 |
| C | 4.844000 | -4.711000 | -1.561000 |
| C | 4.353000 | -2.470000 | -2.289000 |
| C | 5.318000 | -2.837000 | -0.110000 |
| C | 5.312000 | -4.208000 | -0.354000 |
| C | 4.360000 | -3.838000 | -2.531000 |
| H | 3.979000 | -1.787000 | -3.042000 |
| H | 5.671000 | -4.888000 | 0.412000 |
| H | 3.984000 | -4.223000 | -3.471000 |
| H | 4.845000 | -5.779000 | -1.740000 |
| H | 4.047000 | 0.024000 | -1.336000 |
| C | 5.036000 | -0.069000 | 0.703000 |

| | | | |
|---|---|---|---|
| C | 4.017000 | 0.924000 | 1.226000 |
| H | 3.938000 | 1.783000 | 0.559000 |
| H | 3.043000 | 0.440000 | 1.305000 |
| H | 4.312000 | 1.272000 | 2.217000 |
| C | 6.222000 | 0.209000 | -0.999000 |
| C | 6.386000 | 0.514000 | 0.291000 |
| C | 7.062000 | 0.472000 | -2.179000 |
| O | 8.157000 | 0.980000 | -2.153000 |
| O | 6.443000 | 0.069000 | -3.309000 |
| C | 7.183000 | 0.259000 | -4.526000 |
| H | 6.546000 | -0.124000 | -5.319000 |
| H | 7.397000 | 1.317000 | -4.681000 |
| H | 8.124000 | -0.290000 | -4.488000 |
| H | 7.173000 | 1.020000 | 0.837000 |
| O | 5.059000 | -1.147000 | 1.616000 |
| C | 5.824000 | -2.272000 | 1.189000 |
| H | 5.743000 | -3.007000 | 1.990000 |
| H | 6.883000 | -1.992000 | 1.099000 |

**d_Me**

31

| | | | |
|---|---|---|---|
| H | 0.116000 | 1.945000 | 0.234000 |
| C | 0.137000 | 1.548000 | 1.243000 |
| C | 0.188000 | 0.549000 | 3.831000 |
| C | -0.121000 | 0.202000 | 1.453000 |
| C | 0.421000 | 2.395000 | 2.312000 |
| C | 0.443000 | 1.896000 | 3.609000 |
| C | -0.087000 | -0.309000 | 2.763000 |
| H | 0.625000 | 3.443000 | 2.130000 |
| H | 0.663000 | 2.551000 | 4.442000 |
| H | 0.214000 | 0.149000 | 4.838000 |
| C | -0.516000 | -0.725000 | 0.331000 |
| H | -1.609000 | -0.726000 | 0.257000 |
| H | -0.144000 | -0.347000 | -0.625000 |
| C | -0.309000 | -1.730000 | 2.978000 |
| H | -0.428000 | -2.078000 | 3.996000 |
| C | -0.315000 | -2.617000 | 1.970000 |
| C | -0.060000 | -2.183000 | 0.551000 |
| H | -0.621000 | -2.826000 | -0.128000 |
| C | -0.406000 | -4.075000 | 2.181000 |
| O | -0.225000 | -4.886000 | 1.296000 |
| O | -0.700000 | -4.427000 | 3.449000 |
| C | -0.756000 | -5.838000 | 3.706000 |

| | | | |
|---|---|---|---|
| H | -1.524000 | -6.309000 | 3.092000 |
| H | 0.205000 | -6.305000 | 3.490000 |
| H | -1.000000 | -5.933000 | 4.761000 |
| C | 1.431000 | -2.329000 | 0.189000 |
| O | 2.306000 | -2.125000 | 0.995000 |
| C | 1.720000 | -2.720000 | -1.241000 |
| H | 2.781000 | -2.612000 | -1.456000 |
| H | 1.128000 | -2.124000 | -1.940000 |

**o_QDM_1_Me**

31

| | | | |
|---|---|---|---|
| C | 4.992000 | -0.502000 | -0.465000 |
| H | 4.798000 | 0.417000 | -0.996000 |
| C | 3.156000 | 0.019000 | -3.062000 |
| C | 3.465000 | 1.231000 | -3.904000 |
| O | 4.165000 | 1.191000 | -4.887000 |
| O | 2.879000 | 2.356000 | -3.460000 |
| C | 3.109000 | 3.536000 | -4.248000 |
| H | 2.579000 | 4.335000 | -3.735000 |
| H | 2.719000 | 3.402000 | -5.256000 |
| H | 4.175000 | 3.755000 | -4.303000 |
| C | 4.032000 | -1.121000 | -3.352000 |
| H | 5.189000 | -0.421000 | 0.597000 |
| C | 4.776000 | -1.904000 | -2.529000 |
| C | 5.838000 | -4.006000 | -0.908000 |
| C | 5.397000 | -3.086000 | -3.113000 |
| C | 5.013000 | -1.704000 | -1.071000 |
| C | 5.418000 | -2.880000 | -0.305000 |
| C | 5.880000 | -4.088000 | -2.352000 |
| H | 5.390000 | -3.171000 | -4.193000 |
| H | 5.434000 | -2.789000 | 0.775000 |
| H | 6.284000 | -4.977000 | -2.818000 |
| H | 6.177000 | -4.851000 | -0.322000 |
| H | 4.121000 | -1.331000 | -4.414000 |
| C | 2.087000 | 0.049000 | -2.245000 |
| C | 1.511000 | -1.031000 | -1.399000 |
| C | 1.520000 | -2.479000 | -1.838000 |
| H | 1.676000 | -2.598000 | -2.909000 |
| H | 2.313000 | -3.017000 | -1.316000 |
| H | 0.570000 | -2.922000 | -1.541000 |
| O | 0.971000 | -0.705000 | -0.359000 |
| H | 1.574000 | 0.994000 | -2.111000 |

**o_QDM_2_Me**

31

| | | | |
|---|---|---|---|
| H | 0.876000 | 2.367000 | 0.321000 |
| C | 0.824000 | 1.837000 | 1.264000 |
| C | 0.669000 | 0.441000 | 3.704000 |
| C | 0.155000 | 0.542000 | 1.273000 |
| C | 1.294000 | 2.392000 | 2.396000 |
| C | 1.165000 | 1.695000 | 3.654000 |
| C | 0.290000 | -0.278000 | 2.500000 |
| H | 1.753000 | 3.373000 | 2.379000 |
| H | 1.499000 | 2.182000 | 4.562000 |
| H | 0.625000 | -0.092000 | 4.646000 |
| C | -0.651000 | 0.207000 | 0.250000 |
| H | -1.254000 | -0.689000 | 0.259000 |
| H | -0.729000 | 0.843000 | -0.623000 |
| C | 0.087000 | -1.620000 | 2.607000 |
| H | 0.021000 | -1.994000 | 3.624000 |
| C | -0.094000 | -2.670000 | 1.615000 |
| C | 0.672000 | -2.976000 | 0.542000 |
| C | -1.210000 | -3.662000 | 1.882000 |
| O | -1.517000 | -4.574000 | 1.153000 |
| O | -1.852000 | -3.402000 | 3.035000 |
| C | -2.930000 | -4.293000 | 3.366000 |
| H | -3.323000 | -3.936000 | 4.314000 |
| H | -2.565000 | -5.316000 | 3.459000 |
| H | -3.699000 | -4.261000 | 2.593000 |
| H | 0.381000 | -3.862000 | -0.010000 |
| C | 1.909000 | -2.313000 | 0.093000 |
| O | 2.281000 | -1.228000 | 0.499000 |
| C | 2.714000 | -3.088000 | -0.933000 |
| H | 3.627000 | -2.546000 | -1.168000 |
| H | 2.123000 | -3.224000 | -1.844000 |
| H | 2.959000 | -4.086000 | -0.561000 |

**o_QDM_3_Me**

31

| | | | |
|---|---|---|---|
| H | -2.3500565 | 1.6148296 | 2.1501938 |
| C | -1.3542418 | 1.3384629 | 2.4757664 |
| C | 1.2586038 | 0.6431653 | 3.2810324 |
| C | -0.6279620 | 0.3543847 | 1.6897961 |
| C | -0.7673200 | 1.9634662 | 3.5176454 |
| C | 0.5883861 | 1.6467623 | 3.8919714 |

| | | | |
|---|---|---|---|
| C | 0.6281327 | -0.1691142 | 2.2650405 |
| H | -1.2982080 | 2.7335995 | 4.0632326 |
| H | 1.0559249 | 2.2023953 | 4.6951288 |
| H | 2.2560585 | 0.3695699 | 3.6032596 |
| C | -1.0385442 | 0.0437892 | 0.4402840 |
| H | -0.4393685 | -0.5506187 | -0.2332867 |
| H | -1.9758501 | 0.4255748 | 0.0551908 |
| C | 1.1996536 | -1.3661099 | 1.9330081 |
| H | 2.2163619 | -1.5309946 | 2.2654754 |
| C | 0.6133440 | -2.4636062 | 1.2022919 |
| C | 1.3094135 | -3.3649807 | 0.4621933 |
| C | -0.8648608 | -2.7643360 | 1.3256687 |
| O | -1.5369871 | -3.2572390 | 0.4539452 |
| O | -1.3266717 | -2.4829190 | 2.5536804 |
| C | -2.7334257 | -2.6889623 | 2.7511779 |
| H | -2.9278584 | -2.4055810 | 3.7819959 |
| H | -2.9975376 | -3.7324419 | 2.5795274 |
| H | -3.3032873 | -2.0604522 | 2.0654895 |
| H | 0.7519096 | -4.1842145 | 0.0250042 |
| C | 2.7542615 | -3.3239132 | 0.1788547 |
| O | 3.4931009 | -2.4329796 | 0.5665321 |
| C | 3.2881288 | -4.4797445 | -0.6436007 |
| H | 4.3566198 | -4.3554306 | -0.8042063 |
| H | 2.7719033 | -4.5270656 | -1.6066233 |
| H | 3.0983769 | -5.4282962 | -0.1336695 |

**TS1_Me**

31

| | | | |
|---|---|---|---|
| C | 5.154000 | -0.725000 | -1.456000 |
| C | 4.922000 | -2.147000 | -1.159000 |
| C | 4.304000 | -4.919000 | -0.972000 |
| C | 4.148000 | -2.823000 | -2.128000 |
| C | 5.339000 | -2.919000 | -0.034000 |
| C | 5.018000 | -4.276000 | 0.027000 |
| C | 3.852000 | -4.172000 | -2.053000 |
| H | 3.789000 | -2.256000 | -2.979000 |
| H | 5.334000 | -4.842000 | 0.896000 |
| H | 3.269000 | -4.637000 | -2.838000 |
| H | 4.086000 | -5.977000 | -0.896000 |
| H | 4.544000 | -0.412000 | -2.302000 |
| C | 5.265000 | -0.170000 | 0.780000 |
| C | 4.056000 | 0.706000 | 0.885000 |
| H | 4.070000 | 1.479000 | 0.121000 |

| | | | |
|---|---|---|---|
| H | 3.139000 | 0.121000 | 0.815000 |
| H | 4.066000 | 1.190000 | 1.869000 |
| C | 6.294000 | 0.099000 | -1.189000 |
| C | 6.502000 | 0.210000 | 0.154000 |
| C | 7.125000 | 0.768000 | -2.218000 |
| O | 8.116000 | 1.418000 | -1.980000 |
| O | 6.656000 | 0.556000 | -3.467000 |
| C | 7.398000 | 1.185000 | -4.524000 |
| H | 6.881000 | 0.920000 | -5.443000 |
| H | 7.415000 | 2.267000 | -4.390000 |
| H | 8.424000 | 0.817000 | -4.540000 |
| H | 7.444000 | 0.343000 | 0.674000 |
| O | 5.252000 | -1.189000 | 1.645000 |
| C | 6.028000 | -2.337000 | 1.168000 |
| H | 6.021000 | -3.041000 | 1.996000 |
| H | 7.056000 | -2.029000 | 0.985000 |

**TS2_Me**

31

| | | | |
|---|---|---|---|
| C | 4.367000 | -0.916000 | -0.064000 |
| H | 4.347000 | 0.107000 | -0.400000 |
| C | 3.370000 | 0.162000 | -3.092000 |
| C | 3.468000 | 1.269000 | -4.102000 |
| O | 4.143000 | 1.234000 | -5.103000 |
| O | 2.698000 | 2.325000 | -3.773000 |
| C | 2.738000 | 3.436000 | -4.680000 |
| H | 2.080000 | 4.187000 | -4.251000 |
| H | 2.385000 | 3.135000 | -5.666000 |
| H | 3.754000 | 3.822000 | -4.770000 |
| C | 4.256000 | -0.904000 | -3.271000 |
| H | 4.531000 | -1.052000 | 0.996000 |
| C | 4.771000 | -1.899000 | -2.381000 |
| C | 5.847000 | -4.164000 | -0.979000 |
| C | 5.404000 | -2.996000 | -3.043000 |
| C | 4.789000 | -1.952000 | -0.926000 |
| C | 5.296000 | -3.121000 | -0.288000 |
| C | 5.913000 | -4.083000 | -2.384000 |
| H | 5.449000 | -2.968000 | -4.125000 |
| H | 5.263000 | -3.149000 | 0.795000 |
| H | 6.362000 | -4.889000 | -2.951000 |
| H | 6.241000 | -5.026000 | -0.457000 |
| H | 4.673000 | -0.969000 | -4.270000 |
| C | 2.497000 | 0.251000 | -2.009000 |

| | | | |
|---|---|---|---|
| C | 2.142000 | -0.906000 | -1.275000 |
| C | 1.551000 | -2.138000 | -1.901000 |
| H | 1.413000 | -2.036000 | -2.976000 |
| H | 2.160000 | -3.018000 | -1.682000 |
| H | 0.574000 | -2.309000 | -1.437000 |
| O | 2.407000 | -0.942000 | -0.027000 |
| H | 2.312000 | 1.214000 | -1.545000 |

**TS3_Me**

31

| | | | |
|---|---|---|---|
| H | 1.510000 | 2.530000 | 0.570000 |
| C | 1.140000 | 1.993000 | 1.435000 |
| C | 0.147000 | 0.592000 | 3.628000 |
| C | 0.588000 | 0.682000 | 1.218000 |
| C | 1.165000 | 2.566000 | 2.664000 |
| C | 0.633000 | 1.854000 | 3.777000 |
| C | 0.171000 | -0.105000 | 2.371000 |
| H | 1.567000 | 3.562000 | 2.802000 |
| H | 0.628000 | 2.319000 | 4.755000 |
| H | -0.228000 | 0.060000 | 4.494000 |
| C | 0.473000 | 0.223000 | -0.061000 |
| H | -0.100000 | -0.657000 | -0.302000 |
| H | 0.826000 | 0.820000 | -0.891000 |
| C | -0.239000 | -1.444000 | 2.425000 |
| H | -0.719000 | -1.656000 | 3.371000 |
| C | -0.241000 | -2.600000 | 1.604000 |
| C | 0.529000 | -3.056000 | 0.543000 |
| C | -1.270000 | -3.648000 | 2.014000 |
| O | -1.778000 | -4.427000 | 1.246000 |
| O | -1.565000 | -3.633000 | 3.328000 |
| C | -2.542000 | -4.597000 | 3.757000 |
| H | -2.649000 | -4.444000 | 4.827000 |
| H | -2.198000 | -5.609000 | 3.544000 |
| H | -3.490000 | -4.430000 | 3.246000 |
| H | 0.231000 | -4.019000 | 0.153000 |
| C | 1.679000 | -2.468000 | -0.065000 |
| O | 2.150000 | -1.375000 | 0.291000 |
| C | 2.304000 | -3.194000 | -1.242000 |
| H | 3.374000 | -2.991000 | -1.265000 |
| H | 1.871000 | -2.808000 | -2.171000 |
| H | 2.130000 | -4.270000 | -1.214000 |

**TS4_Me**

31

| | | | |
|---|---|---|---|
| H | -0.1167486 | 2.1805547 | 0.1647071 |
| C | 0.0762863 | 1.7478155 | 1.1392237 |
| C | 0.6389466 | 0.6396715 | 3.6408283 |
| C | 0.6310534 | 0.4246546 | 1.1901810 |
| C | -0.0643875 | 2.4975240 | 2.2673251 |
| C | 0.2826480 | 1.9503431 | 3.5335571 |
| C | 0.6976252 | -0.2165333 | 2.4921986 |
| H | -0.4087662 | 3.5217929 | 2.2014673 |
| H | 0.2129726 | 2.5677615 | 4.4203117 |
| H | 0.8039491 | 0.1977345 | 4.6162002 |
| C | 1.1260345 | -0.1385868 | 0.0322525 |
| H | 2.0084993 | -0.7411152 | 0.0705578 |
| H | 0.8843053 | 0.3147124 | -0.9225912 |
| C | 0.4485642 | -1.5781558 | 2.7035316 |
| H | 0.2666429 | -1.8449794 | 3.7370704 |
| C | 0.0029539 | -2.4993013 | 1.7360102 |
| C | 0.5109588 | -2.6661901 | 0.4582480 |
| C | -1.2088571 | -3.3265654 | 2.0600379 |
| O | -1.8679338 | -3.9229833 | 1.2417982 |
| O | -1.4965381 | -3.3387729 | 3.3778521 |
| C | -2.6275388 | -4.1372777 | 3.7602828 |
| H | -2.6987344 | -4.0431847 | 4.8408057 |
| H | -2.4757616 | -5.1783206 | 3.4741217 |
| H | -3.5342623 | -3.7675871 | 3.2812393 |
| H | -0.1423182 | -3.1560450 | -0.2555831 |
| C | 1.9582997 | -2.8518585 | 0.1996751 |
| O | 2.8333093 | -2.6016129 | 1.0081166 |
| C | 2.2871056 | -3.4524802 | -1.1530596 |
| H | 3.3658288 | -3.4921188 | -1.2860936 |
| H | 1.8294855 | -2.8619280 | -1.9511480 |
| H | 1.8733778 | -4.4619674 | -1.2241246 |

**7.4.2. Calculations to structures with R = Ph**

**a_Ph**

38

| | | | |
|---|---|---|---|
| C | 3.459000 | -2.264000 | 0.289000 |
| H | 2.790000 | -1.628000 | -0.291000 |
| C | 4.388000 | 0.053000 | -1.535000 |
| C | 4.013000 | 1.312000 | -2.272000 |

| | | | |
|---|---|---|---|
| O | 4.146000 | 2.427000 | -1.822000 |
| O | 3.500000 | 1.087000 | -3.500000 |
| C | 3.155000 | 2.260000 | -4.252000 |
| H | 4.034000 | 2.883000 | -4.417000 |
| H | 2.403000 | 2.846000 | -3.722000 |
| H | 2.760000 | 1.895000 | -5.197000 |
| C | 4.491000 | -1.088000 | -2.252000 |
| H | 2.856000 | -2.990000 | 0.833000 |
| C | 4.903000 | -2.412000 | -1.792000 |
| C | 5.776000 | -4.961000 | -1.013000 |
| C | 5.753000 | -3.168000 | -2.613000 |
| C | 4.452000 | -2.975000 | -0.588000 |
| C | 4.896000 | -4.243000 | -0.215000 |
| C | 6.200000 | -4.420000 | -2.224000 |
| H | 6.075000 | -2.750000 | -3.559000 |
| H | 4.537000 | -4.675000 | 0.712000 |
| H | 6.871000 | -4.978000 | -2.865000 |
| H | 6.116000 | -5.941000 | -0.703000 |
| H | 4.292000 | -1.015000 | -3.314000 |
| C | 4.780000 | 0.328000 | -0.151000 |
| H | 5.237000 | 1.303000 | -0.067000 |
| C | 4.664000 | -0.303000 | 1.036000 |
| O | 4.079000 | -1.492000 | 1.327000 |
| C | 5.202000 | 0.307000 | 2.279000 |
| C | 6.256000 | 1.416000 | 4.629000 |
| C | 5.707000 | -0.514000 | 3.293000 |
| C | 5.215000 | 1.693000 | 2.472000 |
| C | 5.740000 | 2.241000 | 3.635000 |
| C | 6.235000 | 0.037000 | 4.454000 |
| H | 4.793000 | 2.345000 | 1.719000 |
| H | 5.735000 | 3.315000 | 3.769000 |
| H | 6.664000 | 1.845000 | 5.536000 |
| H | 6.632000 | -0.615000 | 5.223000 |
| H | 5.690000 | -1.587000 | 3.162000 |

**a’_Ph**

38

| | | | |
|---|---|---|---|
| C | 4.045000 | -1.031000 | 0.130000 |
| H | 4.449000 | -0.029000 | -0.013000 |
| C | 3.604000 | 0.197000 | -3.090000 |
| C | 3.555000 | 1.183000 | -4.204000 |
| O | 4.241000 | 1.169000 | -5.199000 |
| O | 2.616000 | 2.125000 | -3.970000 |

| | | | |
|---|---|---|---|
| C | 2.486000 | 3.138000 | -4.978000 |
| H | 1.700000 | 3.802000 | -4.628000 |
| H | 2.214000 | 2.691000 | -5.935000 |
| H | 3.423000 | 3.682000 | -5.098000 |
| C | 4.523000 | -0.796000 | -3.160000 |
| H | 4.236000 | -1.331000 | 1.159000 |
| C | 4.881000 | -1.891000 | -2.233000 |
| C | 5.719000 | -4.254000 | -0.899000 |
| C | 5.525000 | -2.955000 | -2.896000 |
| C | 4.681000 | -2.036000 | -0.820000 |
| C | 5.104000 | -3.217000 | -0.210000 |
| C | 5.933000 | -4.115000 | -2.260000 |
| H | 5.694000 | -2.862000 | -3.961000 |
| H | 4.945000 | -3.326000 | 0.857000 |
| H | 6.415000 | -4.901000 | -2.829000 |
| H | 6.030000 | -5.148000 | -0.374000 |
| H | 5.084000 | -0.827000 | -4.089000 |
| C | 2.818000 | 0.331000 | -1.873000 |
| C | 2.279000 | -0.770000 | -1.299000 |
| O | 2.584000 | -0.994000 | 0.023000 |
| H | 3.067000 | 1.165000 | -1.225000 |
| C | 1.576000 | -1.850000 | -1.988000 |
| C | 0.259000 | -3.915000 | -3.346000 |
| C | 0.902000 | -1.599000 | -3.193000 |
| C | 1.549000 | -3.147000 | -1.460000 |
| C | 0.895000 | -4.169000 | -2.136000 |
| C | 0.264000 | -2.624000 | -3.872000 |
| H | 0.887000 | -5.169000 | -1.719000 |
| H | 2.054000 | -3.346000 | -0.527000 |
| H | -0.249000 | -4.714000 | -3.872000 |
| H | -0.247000 | -2.416000 | -4.804000 |
| H | 0.876000 | -0.588000 | -3.580000 |

**b_Ph**

38

| | | | |
|---|---|---|---|
| C | 4.881000 | -0.467000 | -0.807000 |
| C | 4.847000 | -1.949000 | -1.028000 |
| C | 4.851000 | -4.713000 | -1.427000 |
| C | 4.270000 | -2.500000 | -2.172000 |
| C | 5.417000 | -2.795000 | -0.072000 |
| C | 5.409000 | -4.172000 | -0.275000 |
| C | 4.277000 | -3.873000 | -2.377000 |
| H | 3.824000 | -1.842000 | -2.908000 |

| | | | |
|---|---|---|---|
| H | 5.838000 | -4.826000 | 0.476000 |
| H | 3.829000 | -4.289000 | -3.270000 |
| H | 4.851000 | -5.786000 | -1.577000 |
| H | 4.027000 | 0.021000 | -1.280000 |
| C | 5.111000 | -0.011000 | 0.716000 |
| C | 6.209000 | 0.239000 | -1.056000 |
| C | 6.417000 | 0.600000 | 0.212000 |
| C | 6.999000 | 0.471000 | -2.277000 |
| O | 8.078000 | 1.012000 | -2.312000 |
| O | 6.355000 | -0.002000 | -3.363000 |
| C | 7.043000 | 0.156000 | -4.615000 |
| H | 6.391000 | -0.286000 | -5.365000 |
| H | 7.213000 | 1.212000 | -4.826000 |
| H | 8.003000 | -0.359000 | -4.588000 |
| H | 7.206000 | 1.151000 | 0.708000 |
| O | 5.245000 | -1.074000 | 1.633000 |
| C | 6.012000 | -2.184000 | 1.166000 |
| H | 6.020000 | -2.899000 | 1.989000 |
| H | 7.049000 | -1.871000 | 0.983000 |
| C | 4.073000 | 0.953000 | 1.246000 |
| C | 2.095000 | 2.723000 | 2.147000 |
| C | 3.154000 | 0.555000 | 2.217000 |
| C | 3.989000 | 2.250000 | 0.734000 |
| C | 3.008000 | 3.128000 | 1.179000 |
| C | 2.173000 | 1.435000 | 2.663000 |
| H | 4.696000 | 2.579000 | -0.018000 |
| H | 2.961000 | 4.130000 | 0.771000 |
| H | 1.332000 | 3.407000 | 2.497000 |
| H | 1.469000 | 1.110000 | 3.418000 |
| H | 3.214000 | -0.445000 | 2.623000 |

**d_Ph**

38

| | | | |
|---|---|---|---|
| H | 0.153000 | 1.627000 | -0.032000 |
| C | 0.215000 | 1.308000 | 1.003000 |
| C | 0.371000 | 0.505000 | 3.655000 |
| C | -0.101000 | -0.002000 | 1.333000 |
| C | 0.610000 | 2.215000 | 1.984000 |
| C | 0.684000 | 1.814000 | 3.314000 |
| C | -0.012000 | -0.415000 | 2.675000 |
| H | 0.857000 | 3.233000 | 1.709000 |
| H | 0.989000 | 2.516000 | 4.079000 |
| H | 0.437000 | 0.181000 | 4.688000 |

| | | | |
|---|---|---|---|
| C | -0.607000 | -0.990000 | 0.313000 |
| H | -1.703000 | -0.963000 | 0.321000 |
| H | -0.292000 | -0.699000 | -0.690000 |
| C | -0.300000 | -1.800000 | 3.011000 |
| H | -0.401000 | -2.061000 | 4.057000 |
| C | -0.389000 | -2.759000 | 2.076000 |
| C | -0.171000 | -2.438000 | 0.620000 |
| H | -0.775000 | -3.121000 | 0.024000 |
| C | -0.582000 | -4.187000 | 2.401000 |
| O | -0.594000 | -5.065000 | 1.566000 |
| O | -0.741000 | -4.427000 | 3.719000 |
| C | -0.907000 | -5.805000 | 4.083000 |
| H | -1.788000 | -6.227000 | 3.599000 |
| H | -0.032000 | -6.385000 | 3.790000 |
| H | -1.025000 | -5.810000 | 5.164000 |
| C | 1.304000 | -2.652000 | 0.228000 |
| O | 2.172000 | -2.701000 | 1.072000 |
| C | 1.648000 | -2.740000 | -1.229000 |
| C | 2.423000 | -2.901000 | -3.912000 |
| C | 0.693000 | -2.908000 | -2.236000 |
| C | 2.999000 | -2.655000 | -1.587000 |
| C | 3.383000 | -2.732000 | -2.917000 |
| C | 1.078000 | -2.991000 | -3.568000 |
| H | 3.732000 | -2.530000 | -0.802000 |
| H | 4.431000 | -2.662000 | -3.181000 |
| H | 2.722000 | -2.964000 | -4.951000 |
| H | 0.329000 | -3.130000 | -4.337000 |
| H | -0.357000 | -2.988000 | -1.989000 |

**o_QDM_1_Ph**

38

| | | | |
|---|---|---|---|
| C | 4.685000 | -0.739000 | -0.385000 |
| H | 4.622000 | 0.253000 | -0.806000 |
| C | 3.263000 | 0.289000 | -3.142000 |
| C | 3.598000 | 1.493000 | -3.985000 |
| O | 4.234000 | 1.435000 | -5.010000 |
| O | 3.108000 | 2.642000 | -3.487000 |
| C | 3.361000 | 3.820000 | -4.272000 |
| H | 2.915000 | 4.641000 | -3.716000 |
| H | 2.903000 | 3.728000 | -5.257000 |
| H | 4.433000 | 3.974000 | -4.393000 |
| C | 4.103000 | -0.868000 | -3.433000 |
| H | 4.739000 | -0.811000 | 0.693000 |

| | | | |
|---|---|---|---|
| C | 4.641000 | -1.830000 | -2.634000 |
| C | 5.272000 | -4.230000 | -1.209000 |
| C | 5.216000 | -2.991000 | -3.298000 |
| C | 4.694000 | -1.849000 | -1.146000 |
| C | 4.888000 | -3.148000 | -0.509000 |
| C | 5.492000 | -4.131000 | -2.634000 |
| H | 5.339000 | -2.942000 | -4.373000 |
| H | 4.765000 | -3.197000 | 0.567000 |
| H | 5.860000 | -4.996000 | -3.172000 |
| H | 5.446000 | -5.175000 | -0.711000 |
| H | 4.332000 | -0.955000 | -4.490000 |
| C | 2.189000 | 0.353000 | -2.329000 |
| C | 1.669000 | -0.708000 | -1.425000 |
| O | 1.469000 | -0.398000 | -0.262000 |
| H | 1.698000 | 1.307000 | -2.194000 |
| C | 1.385000 | -2.083000 | -1.922000 |
| C | 0.833000 | -4.680000 | -2.768000 |
| C | 1.194000 | -2.355000 | -3.278000 |
| C | 1.287000 | -3.120000 | -0.993000 |
| C | 1.022000 | -4.414000 | -1.414000 |
| C | 0.912000 | -3.648000 | -3.699000 |
| H | 0.965000 | -5.218000 | -0.691000 |
| H | 1.436000 | -2.892000 | 0.054000 |
| H | 0.625000 | -5.690000 | -3.098000 |
| H | 0.760000 | -3.852000 | -4.751000 |
| H | 1.266000 | -1.555000 | -4.002000 |

**o_QDM_2_Ph**

38

| | | | |
|---|---|---|---|
| H | 0.873000 | 2.764000 | 0.756000 |
| C | 0.770000 | 2.114000 | 1.617000 |
| C | 0.475000 | 0.422000 | 3.818000 |
| C | 0.374000 | 0.740000 | 1.364000 |
| C | 0.983000 | 2.589000 | 2.861000 |
| C | 0.810000 | 1.721000 | 3.990000 |
| C | 0.309000 | -0.184000 | 2.513000 |
| H | 1.268000 | 3.622000 | 3.017000 |
| H | 0.958000 | 2.113000 | 4.989000 |
| H | 0.364000 | -0.220000 | 4.684000 |
| C | 0.057000 | 0.394000 | 0.096000 |
| H | -0.298000 | -0.587000 | -0.170000 |
| H | 0.118000 | 1.125000 | -0.700000 |
| C | 0.045000 | -1.539000 | 2.527000 |

| | | | |
|---|---|---|---|
| H | -0.184000 | -1.898000 | 3.523000 |
| C | -0.095000 | -2.597000 | 1.562000 |
| C | 0.638000 | -2.932000 | 0.458000 |
| C | -1.169000 | -3.631000 | 1.878000 |
| O | -1.459000 | -4.560000 | 1.163000 |
| O | -1.796000 | -3.396000 | 3.045000 |
| C | -2.822000 | -4.337000 | 3.402000 |
| H | -3.211000 | -3.994000 | 4.357000 |
| H | -2.406000 | -5.340000 | 3.492000 |
| H | -3.608000 | -4.347000 | 2.646000 |
| H | 0.307000 | -3.826000 | -0.048000 |
| C | 1.835000 | -2.276000 | -0.045000 |
| O | 2.230000 | -1.209000 | 0.419000 |
| C | 2.581000 | -2.917000 | -1.180000 |
| C | 4.065000 | -3.990000 | -3.299000 |
| C | 3.532000 | -2.141000 | -1.852000 |
| C | 2.391000 | -4.243000 | -1.585000 |
| C | 3.132000 | -4.776000 | -2.633000 |
| C | 4.263000 | -2.669000 | -2.905000 |
| H | 1.680000 | -4.879000 | -1.075000 |
| H | 2.979000 | -5.806000 | -2.929000 |
| H | 4.637000 | -4.404000 | -4.120000 |
| H | 4.989000 | -2.052000 | -3.420000 |
| H | 3.682000 | -1.120000 | -1.527000 |

**o_QDM_3_Ph**

38

| | | | |
|---|---|---|---|
| H | -2.6668584 | 1.4863474 | 2.8180818 |
| C | -1.6251274 | 1.2766164 | 3.0300440 |
| C | 1.1011107 | 0.7636311 | 3.5415159 |
| C | -0.8777449 | 0.4884784 | 2.0639273 |
| C | -1.0147211 | 1.8161992 | 4.1057342 |
| C | 0.3915766 | 1.5984735 | 4.3350490 |
| C | 0.4685325 | 0.0328597 | 2.4671397 |
| H | -1.5689411 | 2.4456463 | 4.7908049 |
| H | 0.8697219 | 2.0874415 | 5.1745004 |
| H | 2.1438497 | 0.5572849 | 3.7504071 |
| C | -1.3644750 | 0.3011488 | 0.8170053 |
| H | -0.7720680 | -0.1318728 | 0.0249069 |
| H | -2.3646466 | 0.6264814 | 0.5592911 |
| C | 1.1248837 | -1.0387752 | 1.9267356 |
| H | 2.1770376 | -1.1376418 | 2.1581528 |
| C | 0.5860582 | -2.0841961 | 1.0910719 |

| | | | |
|---|---|---|---|
| C | 1.2836571 | -2.7906744 | 0.1627296 |
| C | -0.8347562 | -2.5661328 | 1.2936887 |
| O | -1.5329475 | -3.0216918 | 0.4215226 |
| O | -1.2051420 | -2.4936778 | 2.5811727 |
| C | -2.5575051 | -2.8839414 | 2.8622395 |
| H | -2.6814528 | -2.7549193 | 3.9340232 |
| H | -2.7263586 | -3.9216101 | 2.5745301 |
| H | -3.2524609 | -2.2455135 | 2.3150367 |
| H | 0.7529537 | -3.5960528 | -0.3223114 |
| C | 2.6807244 | -2.5539970 | -0.2300310 |
| O | 3.3865930 | -1.7166373 | 0.3214782 |
| C | 3.2517985 | -3.3943903 | -1.3358082 |
| C | 4.4416234 | -4.8807826 | -3.3861233 |
| C | 4.6446068 | -3.4478498 | -1.4619875 |
| C | 2.4642872 | -4.0910737 | -2.2585432 |
| C | 3.0565760 | -4.8232513 | -3.2808185 |
| C | 5.2350809 | -4.1908307 | -2.4722682 |
| H | 1.3850239 | -4.0488063 | -2.2042037 |
| H | 2.4347657 | -5.3483680 | -3.9950328 |
| H | 4.9019436 | -5.4580793 | -4.1785009 |
| H | 6.3143560 | -4.2319050 | -2.5519361 |
| H | 5.2464444 | -2.8949374 | -0.7532242 |

**TS1_Ph**

38

| | | | |
|---|---|---|---|
| C | 5.116000 | -0.614000 | -1.293000 |
| C | 4.878000 | -2.017000 | -0.981000 |
| C | 4.175000 | -4.754000 | -0.653000 |
| C | 3.913000 | -2.657000 | -1.794000 |
| C | 5.448000 | -2.804000 | 0.062000 |
| C | 5.081000 | -4.143000 | 0.201000 |
| C | 3.577000 | -3.989000 | -1.650000 |
| H | 3.437000 | -2.075000 | -2.574000 |
| H | 5.517000 | -4.718000 | 1.010000 |
| H | 2.845000 | -4.432000 | -2.315000 |
| H | 3.926000 | -5.800000 | -0.529000 |
| H | 4.403000 | -0.254000 | -2.032000 |
| C | 5.503000 | -0.030000 | 0.912000 |
| C | 6.316000 | 0.182000 | -1.174000 |
| C | 6.655000 | 0.369000 | 0.121000 |
| C | 7.064000 | 0.765000 | -2.317000 |
| O | 8.083000 | 1.406000 | -2.212000 |
| O | 6.474000 | 0.487000 | -3.498000 |

| | | | |
|---|---|---|---|
| C | 7.121000 | 1.029000 | -4.660000 |
| H | 6.511000 | 0.723000 | -5.505000 |
| H | 7.174000 | 2.117000 | -4.597000 |
| H | 8.133000 | 0.633000 | -4.752000 |
| H | 7.633000 | 0.601000 | 0.528000 |
| O | 5.635000 | -1.085000 | 1.727000 |
| C | 6.338000 | -2.219000 | 1.113000 |
| H | 6.498000 | -2.919000 | 1.929000 |
| H | 7.306000 | -1.884000 | 0.744000 |
| C | 4.324000 | 0.799000 | 1.157000 |
| C | 2.053000 | 2.368000 | 1.645000 |
| C | 3.202000 | 0.252000 | 1.798000 |
| C | 4.298000 | 2.154000 | 0.790000 |
| C | 3.169000 | 2.923000 | 1.022000 |
| C | 2.080000 | 1.032000 | 2.036000 |
| H | 5.170000 | 2.595000 | 0.325000 |
| H | 3.164000 | 3.966000 | 0.731000 |
| H | 1.176000 | 2.974000 | 1.834000 |
| H | 1.219000 | 0.594000 | 2.526000 |
| H | 3.217000 | -0.788000 | 2.091000 |

**TS2_Ph**

38

| | | | |
|---|---|---|---|
| C | 4.310000 | -0.851000 | -0.095000 |
| H | 4.349000 | 0.161000 | -0.464000 |
| C | 3.429000 | 0.230000 | -3.113000 |
| C | 3.614000 | 1.348000 | -4.099000 |
| O | 4.357000 | 1.317000 | -5.051000 |
| O | 2.837000 | 2.411000 | -3.811000 |
| C | 2.962000 | 3.533000 | -4.698000 |
| H | 2.284000 | 4.289000 | -4.311000 |
| H | 2.683000 | 3.249000 | -5.713000 |
| H | 3.988000 | 3.903000 | -4.705000 |
| C | 4.290000 | -0.862000 | -3.268000 |
| H | 4.441000 | -0.961000 | 0.973000 |
| C | 4.709000 | -1.892000 | -2.373000 |
| C | 5.582000 | -4.211000 | -0.927000 |
| C | 5.267000 | -3.041000 | -3.014000 |
| C | 4.696000 | -1.928000 | -0.920000 |
| C | 5.105000 | -3.120000 | -0.257000 |
| C | 5.674000 | -4.155000 | -2.333000 |
| H | 5.328000 | -3.032000 | -4.095000 |
| H | 5.052000 | -3.133000 | 0.826000 |

| | | | |
|---|---|---|---|
| H | 6.063000 | -5.005000 | -2.882000 |
| H | 5.900000 | -5.095000 | -0.390000 |
| H | 4.742000 | -0.933000 | -4.251000 |
| C | 2.493000 | 0.339000 | -2.088000 |
| C | 2.087000 | -0.809000 | -1.352000 |
| O | 2.355000 | -0.839000 | -0.101000 |
| H | 2.291000 | 1.308000 | -1.648000 |
| C | 1.513000 | -2.007000 | -1.989000 |
| C | 0.449000 | -4.305000 | -3.173000 |
| C | 0.854000 | -1.931000 | -3.223000 |
| C | 1.605000 | -3.246000 | -1.345000 |
| C | 1.080000 | -4.387000 | -1.936000 |
| C | 0.335000 | -3.072000 | -3.813000 |
| H | 1.167000 | -5.342000 | -1.434000 |
| H | 2.105000 | -3.301000 | -0.388000 |
| H | 0.038000 | -5.195000 | -3.633000 |
| H | -0.170000 | -3.003000 | -4.769000 |
| H | 0.751000 | -0.969000 | -3.709000 |

**TS3_Ph**

38

| | | | |
|---|---|---|---|
| H | 1.440000 | 2.688000 | 0.799000 |
| C | 1.079000 | 2.099000 | 1.634000 |
| C | 0.111000 | 0.566000 | 3.750000 |
| C | 0.577000 | 0.780000 | 1.349000 |
| C | 1.071000 | 2.615000 | 2.888000 |
| C | 0.552000 | 1.835000 | 3.962000 |
| C | 0.172000 | -0.073000 | 2.461000 |
| H | 1.436000 | 3.617000 | 3.076000 |
| H | 0.521000 | 2.255000 | 4.960000 |
| H | -0.256000 | -0.017000 | 4.585000 |
| C | 0.501000 | 0.377000 | 0.049000 |
| H | -0.022000 | -0.517000 | -0.244000 |
| H | 0.846000 | 1.024000 | -0.746000 |
| C | -0.200000 | -1.421000 | 2.450000 |
| H | -0.682000 | -1.691000 | 3.381000 |
| C | -0.176000 | -2.535000 | 1.570000 |
| C | 0.627000 | -2.927000 | 0.511000 |
| C | -1.208000 | -3.612000 | 1.892000 |
| O | -1.627000 | -4.407000 | 1.087000 |
| O | -1.619000 | -3.603000 | 3.174000 |
| C | -2.597000 | -4.597000 | 3.521000 |
| H | -2.806000 | -4.444000 | 4.577000 |

| | | | |
|---|---|---|---|
| H | -2.201000 | -5.598000 | 3.348000 |
| H | -3.501000 | -4.467000 | 2.926000 |
| H | 0.328000 | -3.860000 | 0.059000 |
| C | 1.799000 | -2.295000 | -0.010000 |
| O | 2.213000 | -1.213000 | 0.445000 |
| C | 2.519000 | -2.921000 | -1.169000 |
| C | 3.950000 | -3.963000 | -3.345000 |
| C | 3.467000 | -2.142000 | -1.843000 |
| C | 2.307000 | -4.235000 | -1.601000 |
| C | 3.020000 | -4.752000 | -2.677000 |
| C | 4.171000 | -2.654000 | -2.923000 |
| H | 1.598000 | -4.875000 | -1.094000 |
| H | 2.848000 | -5.773000 | -2.992000 |
| H | 4.502000 | -4.365000 | -4.185000 |
| H | 4.896000 | -2.034000 | -3.436000 |
| H | 3.640000 | -1.132000 | -1.498000 |

**TS4_Ph**

38

| | | | |
|---|---|---|---|
| H | -0.2913980 | 2.1356958 | 0.1088260 |
| C | -0.1060414 | 1.7497166 | 1.1042651 |
| C | 0.4371210 | 0.7676649 | 3.6625975 |
| C | 0.5611565 | 0.4851478 | 1.2229026 |
| C | -0.3654758 | 2.5148814 | 2.2012097 |
| C | -0.0304981 | 2.0373002 | 3.4979773 |
| C | 0.6273865 | -0.1096746 | 2.5454178 |
| H | -0.7965503 | 3.5014555 | 2.0874878 |
| H | -0.1971086 | 2.6700170 | 4.3606993 |
| H | 0.5947386 | 0.3696903 | 4.6579831 |
| C | 1.1553983 | -0.0700047 | 0.1077862 |
| H | 2.0901917 | -0.5806968 | 0.2040767 |
| H | 0.9186842 | 0.3220705 | -0.8748312 |
| C | 0.4962769 | -1.4836522 | 2.7874349 |
| H | 0.2993085 | -1.7383439 | 3.8212245 |
| C | 0.1721814 | -2.4684746 | 1.8363289 |
| C | 0.7150521 | -2.6210454 | 0.5702484 |
| C | -0.9630932 | -3.3996566 | 2.1587032 |
| O | -1.5779318 | -4.0364706 | 1.3366917 |
| O | -1.2350677 | -3.4559886 | 3.4784602 |
| C | -2.2935107 | -4.3500820 | 3.8576791 |
| H | -2.3606644 | -4.2796048 | 4.9402767 |
| H | -2.0603276 | -5.3704264 | 3.5525056 |
| H | -3.2323114 | -4.0477899 | 3.3932834 |

| | | | |
|---|---|---|---|
| H | 0.1136455 | -3.1894631 | -0.1277596 |
| C | 2.1761211 | -2.6725247 | 0.3406186 |
| O | 2.9916636 | -2.4475119 | 1.2225920 |
| C | 2.6427834 | -3.0822292 | -1.0268638 |
| C | 3.6400712 | -3.8092696 | -3.5344421 |
| C | 3.9625874 | -3.5228397 | -1.1654745 |
| C | 1.8316363 | -3.0033496 | -2.1623753 |
| C | 2.3300790 | -3.3586380 | -3.4098376 |
| C | 4.4554262 | -3.8916138 | -2.4082561 |
| H | 0.8140376 | -2.6449451 | -2.0821349 |
| H | 1.6951514 | -3.2852761 | -4.2839944 |
| H | 4.0253775 | -4.0934626 | -4.5059341 |
| H | 5.4762662 | -4.2407892 | -2.5022534 |
| H | 4.5856370 | -3.5668162 | -0.2821195 |

## 8. References in the supporting information


[1] M. Zhou, L. A. Wolzak, Z. Li, F. J. de Zwart, S. Mathew, B. de Bruin. *J. Am. Chem. Soc.* **2021**, *143*, 20501–20512.

[2] Bruker, *SAINT* V8.40B, Bruker *AXS* Inc., Madison, Wisconsin, USA, 2001.

[3] *SADABS*-2016/2 - Bruker *AXS* area detector scaling and absorption correction: L. Krause, R. Herbst-Irmer, G.M. Sheldrick, D. Stalke, J. Appl. Cryst. **2015**, *48*, 3-10.

[4] G. M. Sheldrick. Acta Cryst. A., **2015**, *71*, 3-8.

[5] G. M. Sheldrick. Acta Cryst. C., **2015**, *71*, 3-8.

[6] M. A. Cismesia, T. P. Yoon. *Chem. Sci*. **2015**, 6, 5426.

[7] C. G. Hatchard, C. A. Parker. *Proc. Roy. Soc. (London)* **1956**, *A235*, 518–536.

[8] T. Wan, L. Capaldo, G. Laudadio, A. V. Nyuchev, J. A. Rincon, P. Garcia-Losada, C. Mateos, M. O. Frederick, M. Nuno, T. Noel. *Angew. Chem. Int. Ed*. **2021**, 60, 17893 –17897.

[9] TURBOMOLE Version 7.5.1 (TURBOMOLE Gmbh, Karlsruhe, Germany).

[10] (a) PQS version 2.4, 2001, Parallel Quantum Solutions, Fayetteville, Arkansas, USA (the Baker optimizer is available separately from PQS upon request); (b) J. Baker, *J. Comput. Chem*. **1986**, *7*, 385–395.

[11] P. H. M. Budzelaar, *J. Comput. Chem.* **2007**, *28*, 2226–2236.

[12] (a) A. D. Becke, *J. Chem. Phys.* **1993**, *98*, 5648–5652. (b) C. Lee, W. Yang, R. G. Parr. *Phys. Rev. B* **1988**, *37*, 785–789.

[13] (a) F. Weigend, R. Ahlrichs. *Phys. Chem. Chem. Phys.* **2005**, *7*, 3297–3305. (b) F. Weigend, M. Häser, H. Patzelt, R. Ahlrichs. *Chem. Phys. Lett.* **1998**, *294*, 143–152.

[14] S. Grimme, J. Antony, S. Ehrlich, H. Krieg. *J. Chem. Phys.* **2010**, *132*, 154104.

## 9. NMR spectra

### 9.1. 2D-NMR characterization

9.1.1 Combined 1D- and 2D-NMR characterization of product **1b**

Ethyl 2a-phenyl-2a,8b-dihydro-4*H*-cyclobuta[c]isochromene-1-carboxylate (**1b**)

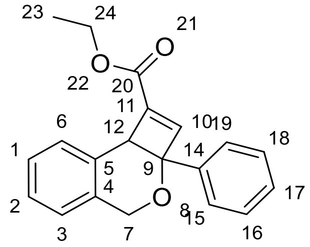


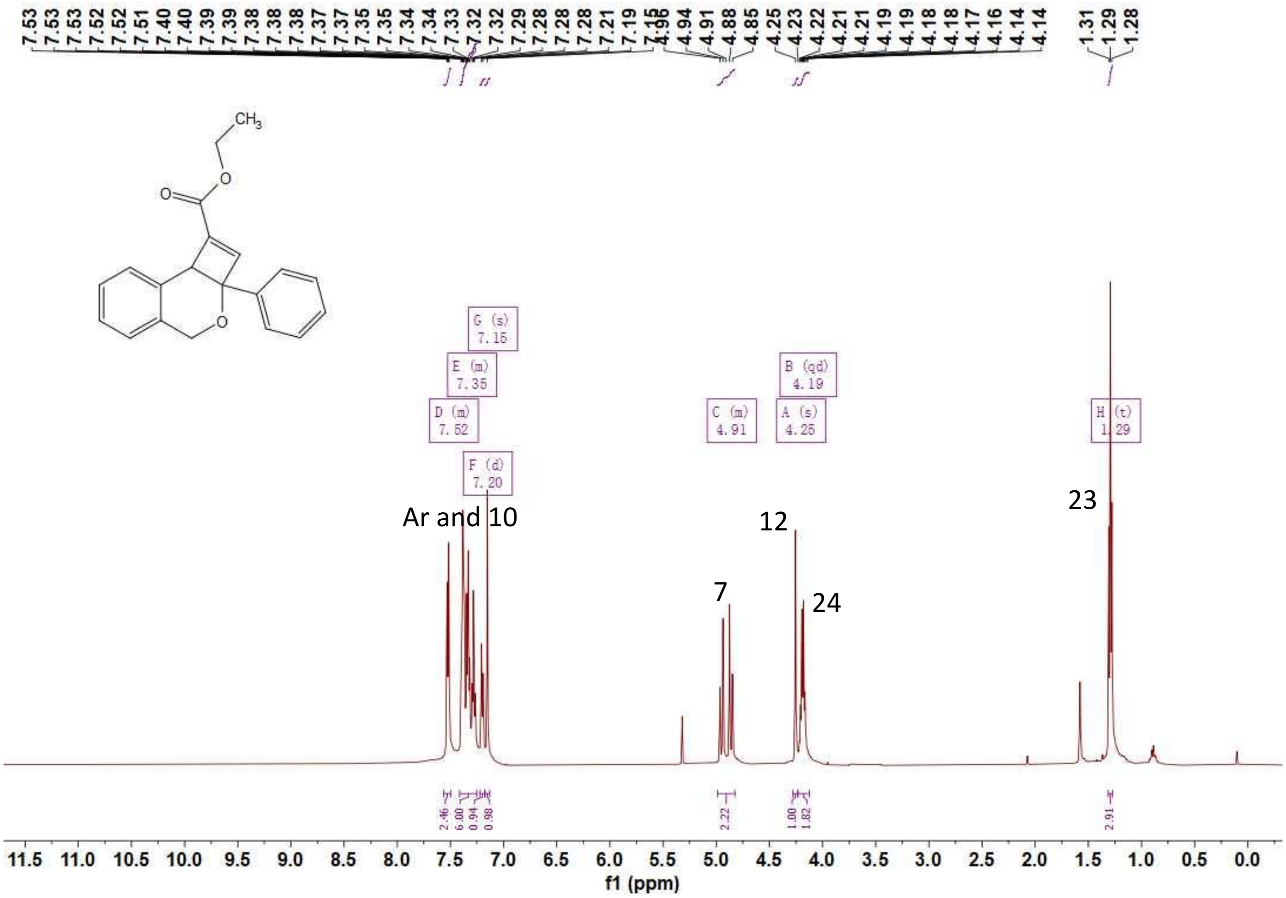


Figure S25. $^1$H-NMR of **1b** (500 MHz, $CDCl_3$)

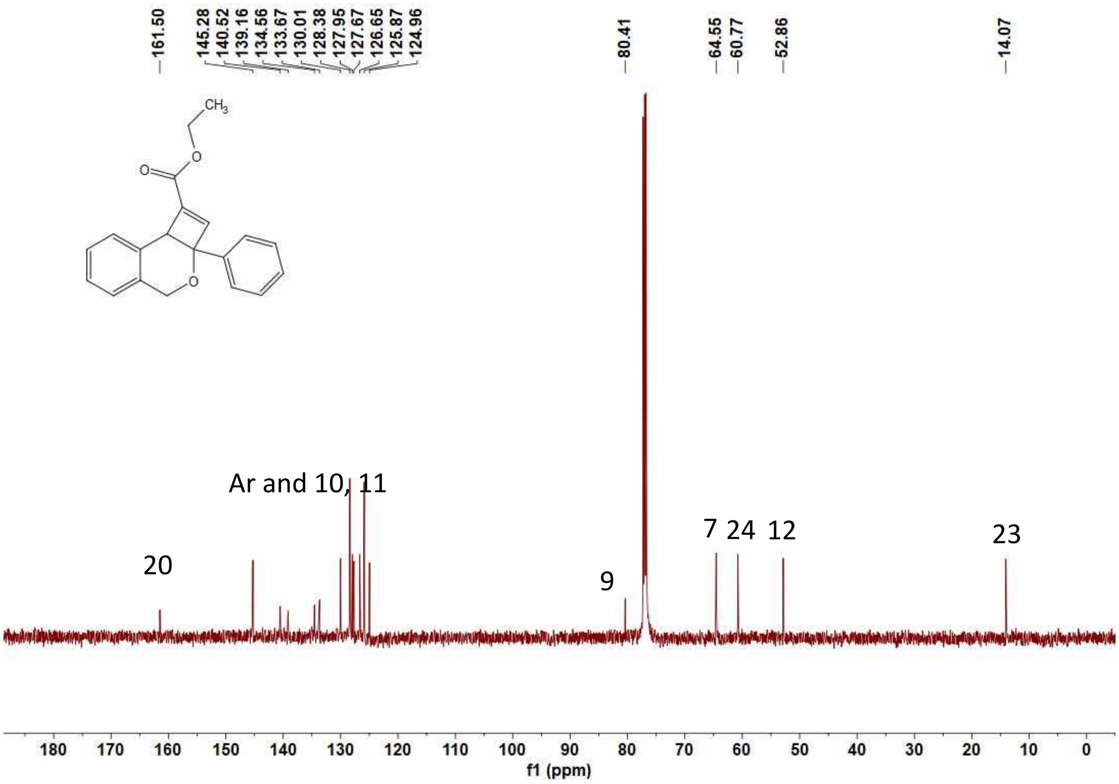


Figure S26. $^{13}$C-NMR of **1b** (125 MHz, $CDCl_3$)

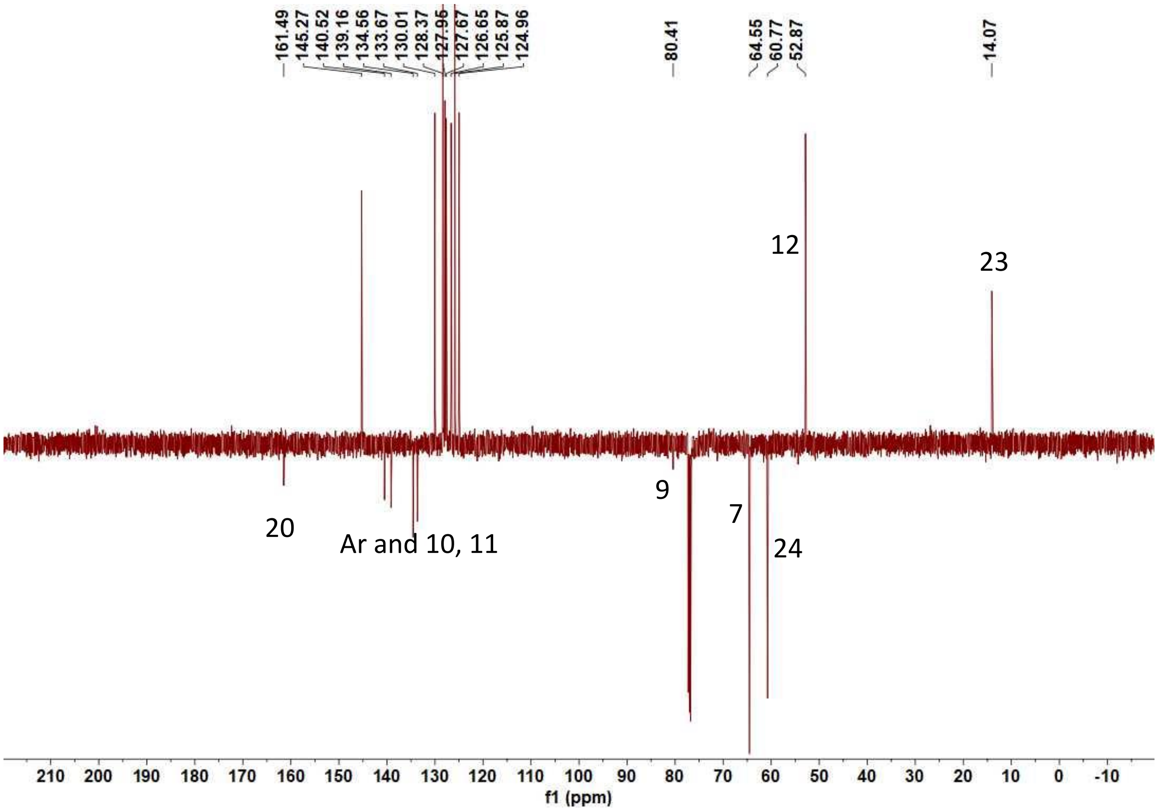


Figure S27. $^{13}$C-NMR-APT of **1b** (125 MHz, $CDCl_3$)

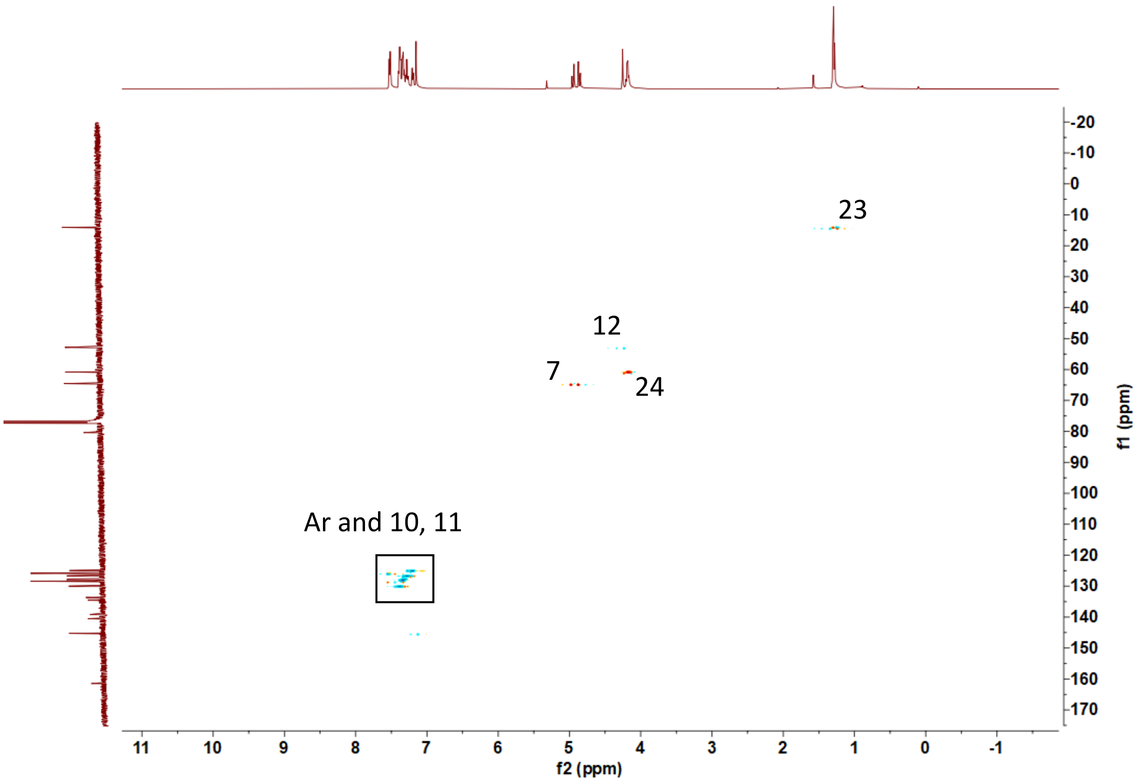


Figure S28. $^{1}H$-$^{13}C$ HSQC-NMR of **1b** ($CDCl_3$)

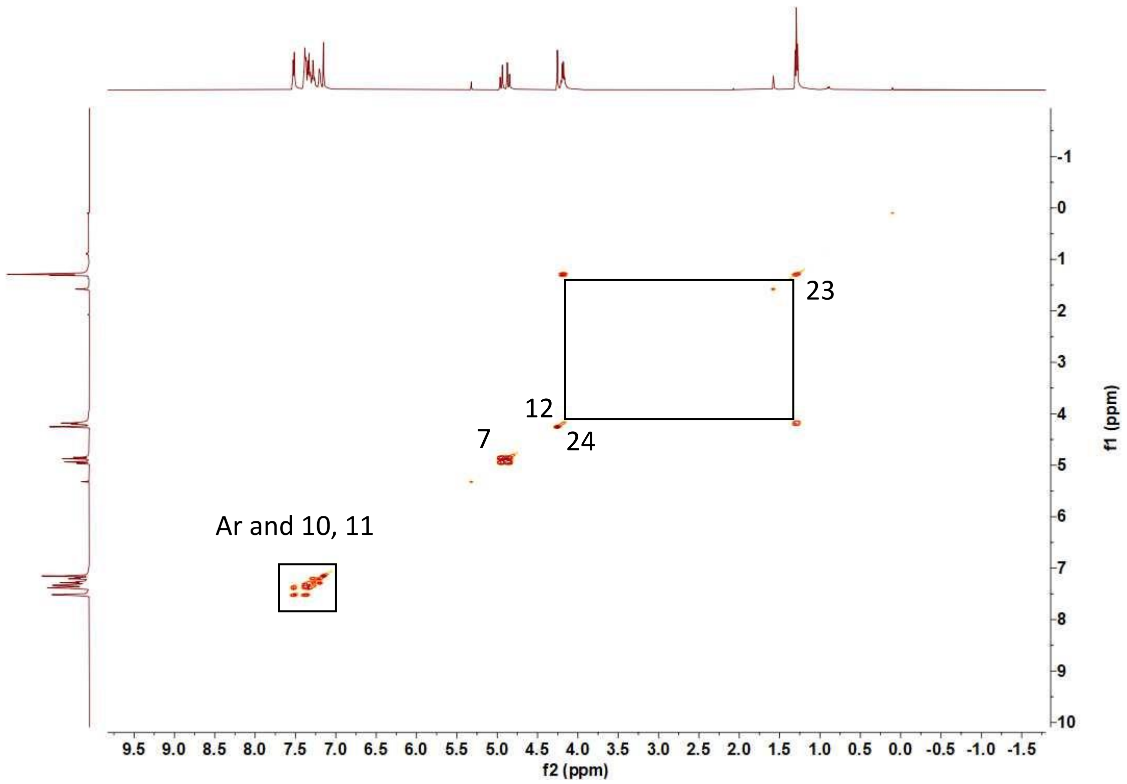


Figure S29. $^{1}H$-$^{1}H$ COSY-NMR of **1b**($CDCl_3$)

9.1.2 Combined 1D- and 2D-NMR characterization of product **13b**

Ethyl 2a-methyl-2a,8b-dihydro-4*H*-cyclobuta[c]isochromene-1-carboxylate (**13b**)

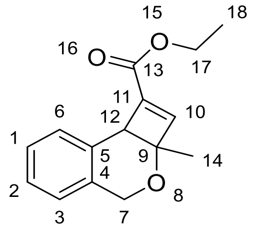


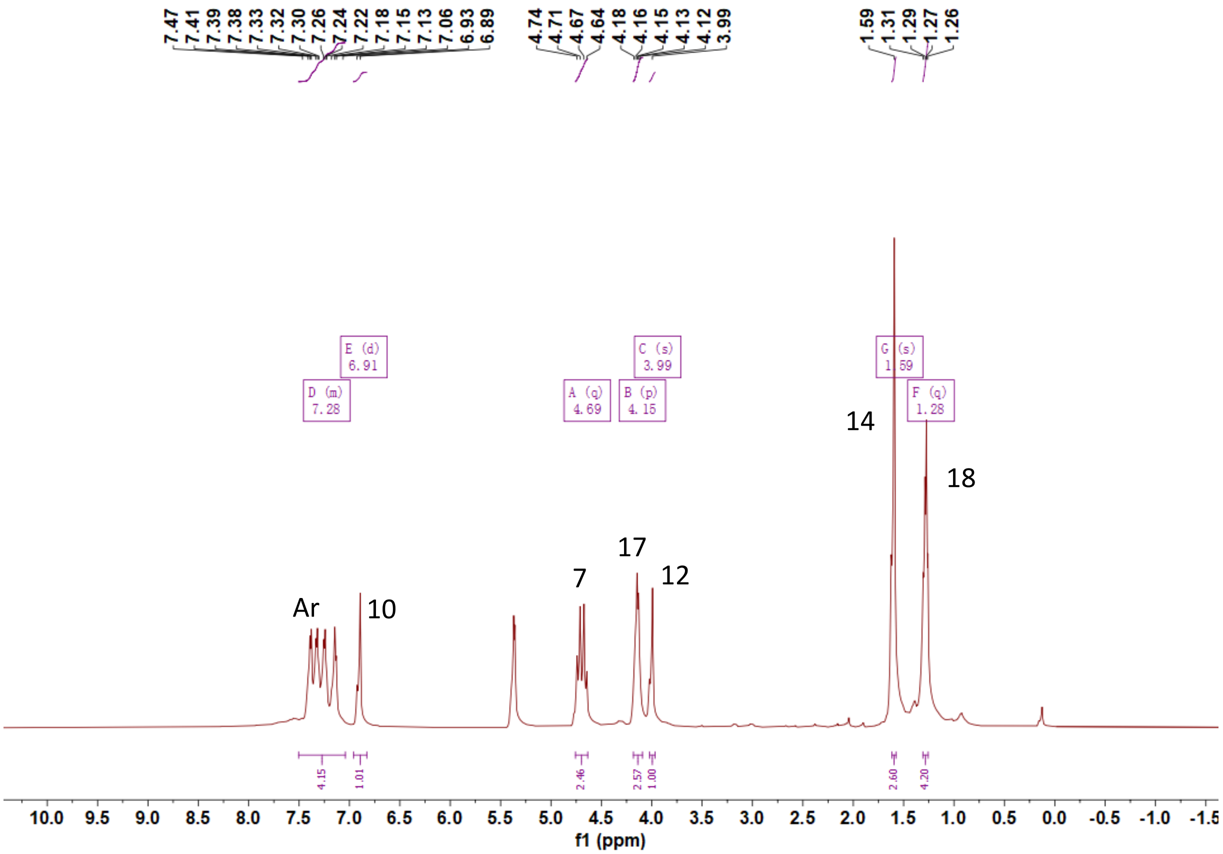


Figure S30. $^1$H-NMR of **13b** (500 MHz, $CD_2Cl_2$)

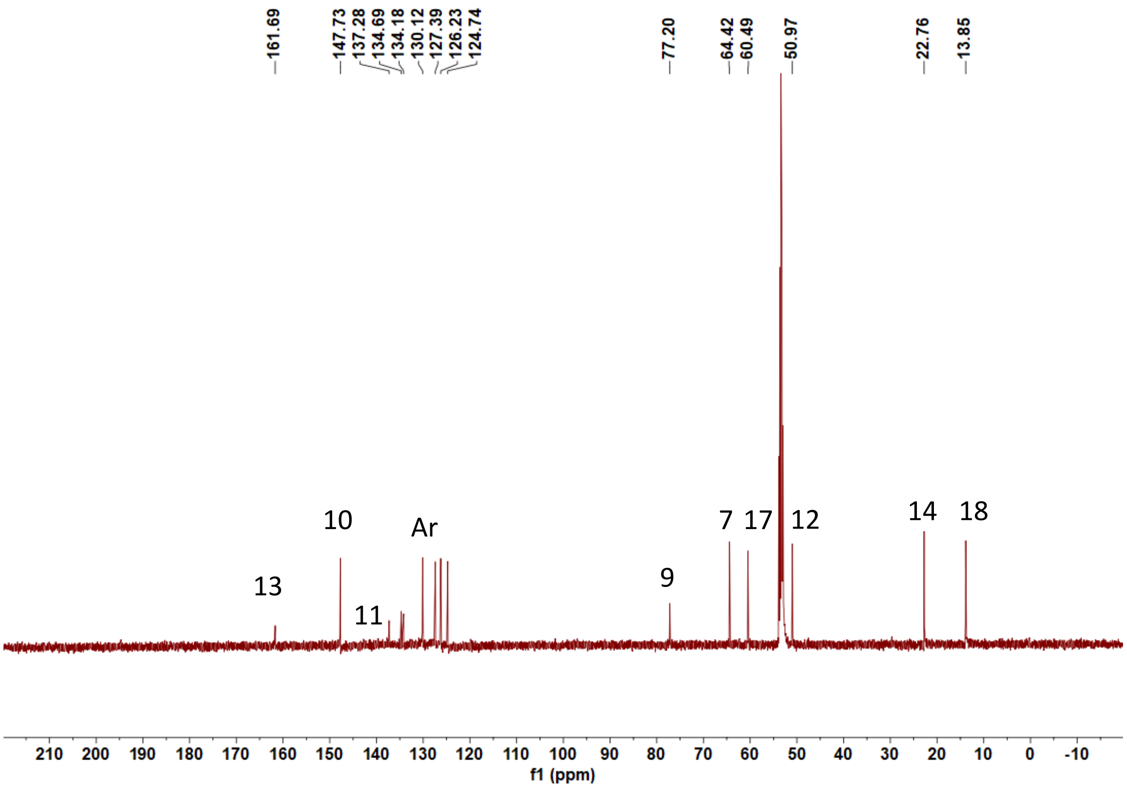


Figure S31. $^{13}$C-NMR of **13b** (125 MHz, $CD_2Cl_2$)

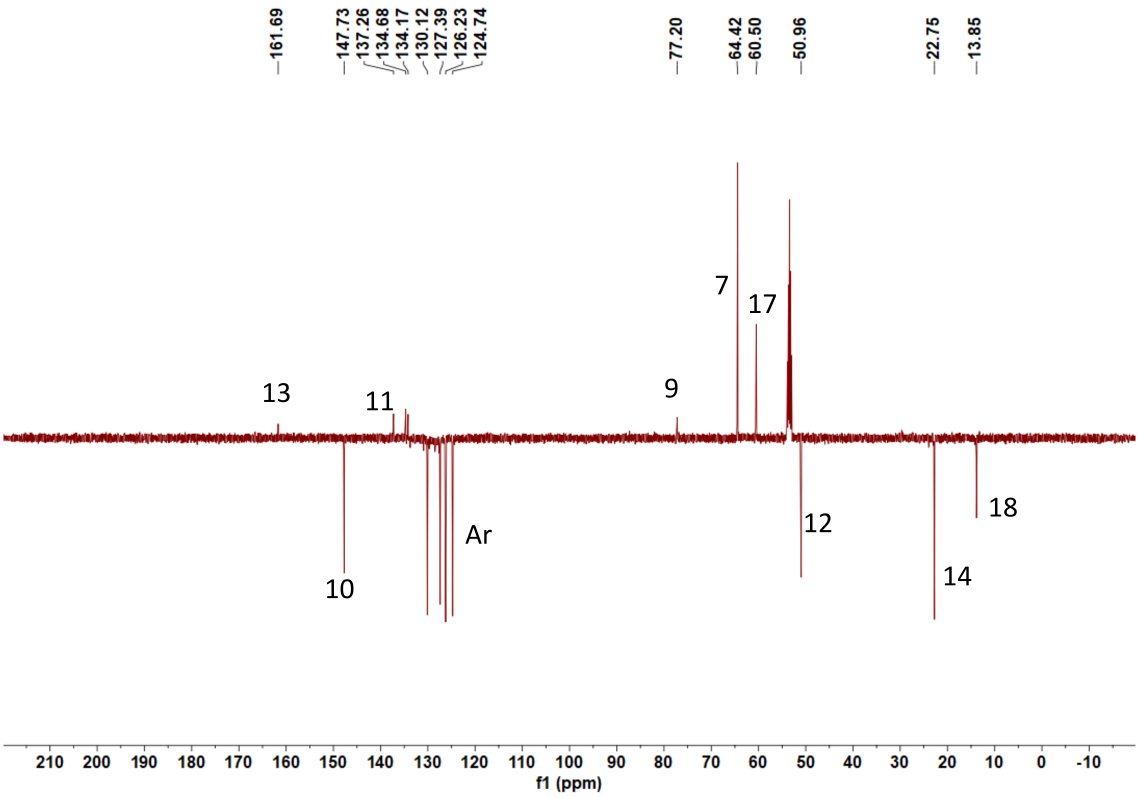


Figure S32. $^{13}$C-NMR-APT of **13b** (125 MHz, $CD_2Cl_2$)

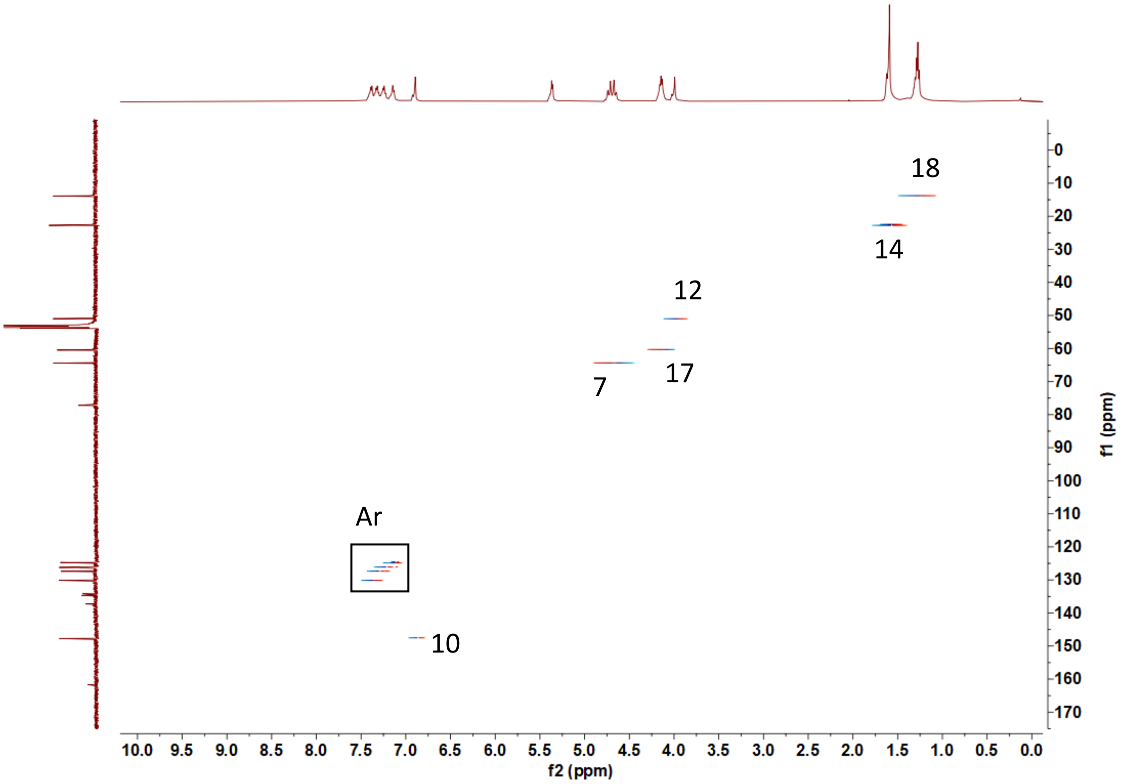


Figure S33. $^{1}H$-$^{13}C$ HSQC-NMR of **13b** ($CD_2Cl_2$)

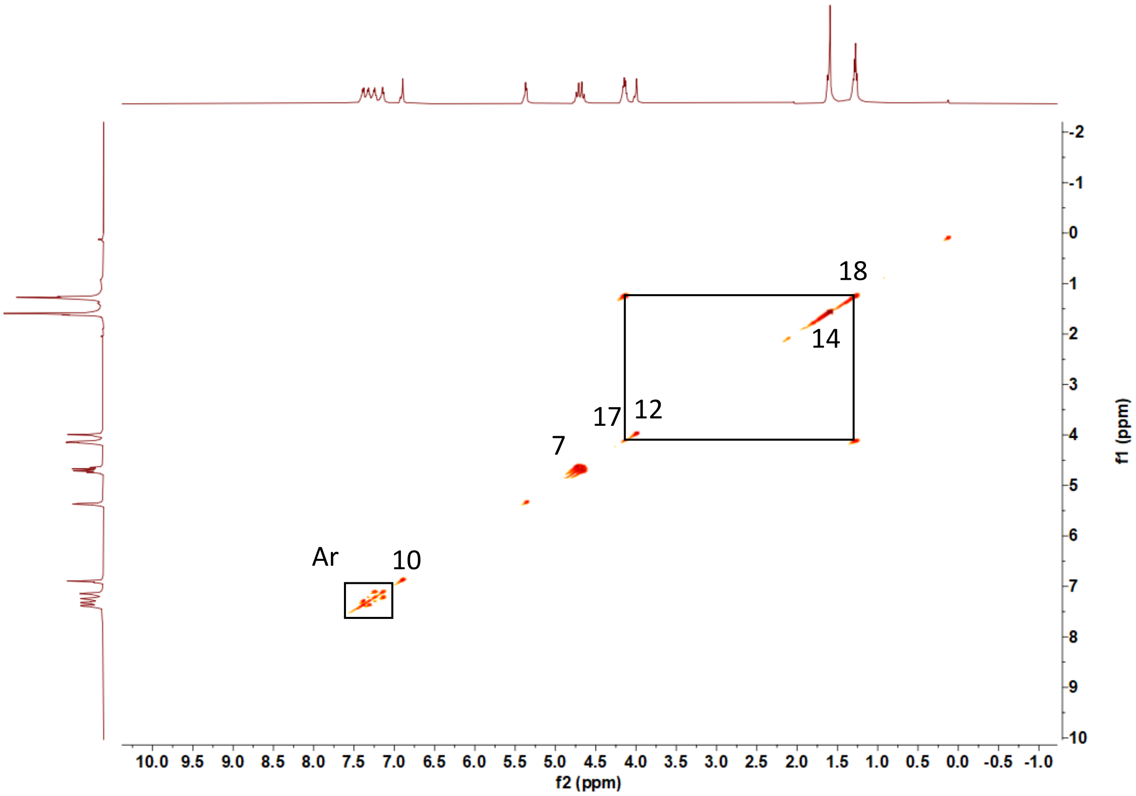


Figure S34. $^{1}H$-$^{1}H$ COSY-NMR of **13b** ($CD_2Cl_2$)

## 9.2. NMR spectra of dihydro-4*H*-cyclobuta[*c*]isochromenes

### Ethyl 2a-phenyl-2a,8b-dihydro-4*H*-cyclobuta[c]isochromene-1-carboxylate (1b)

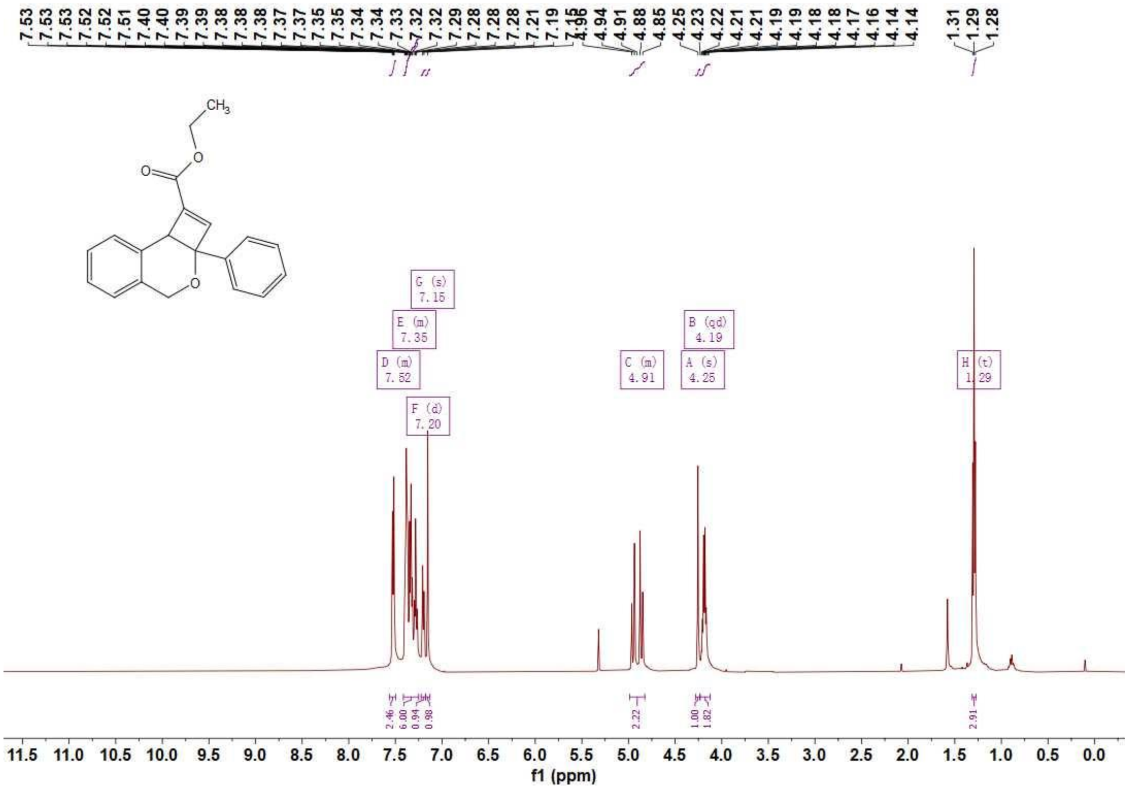


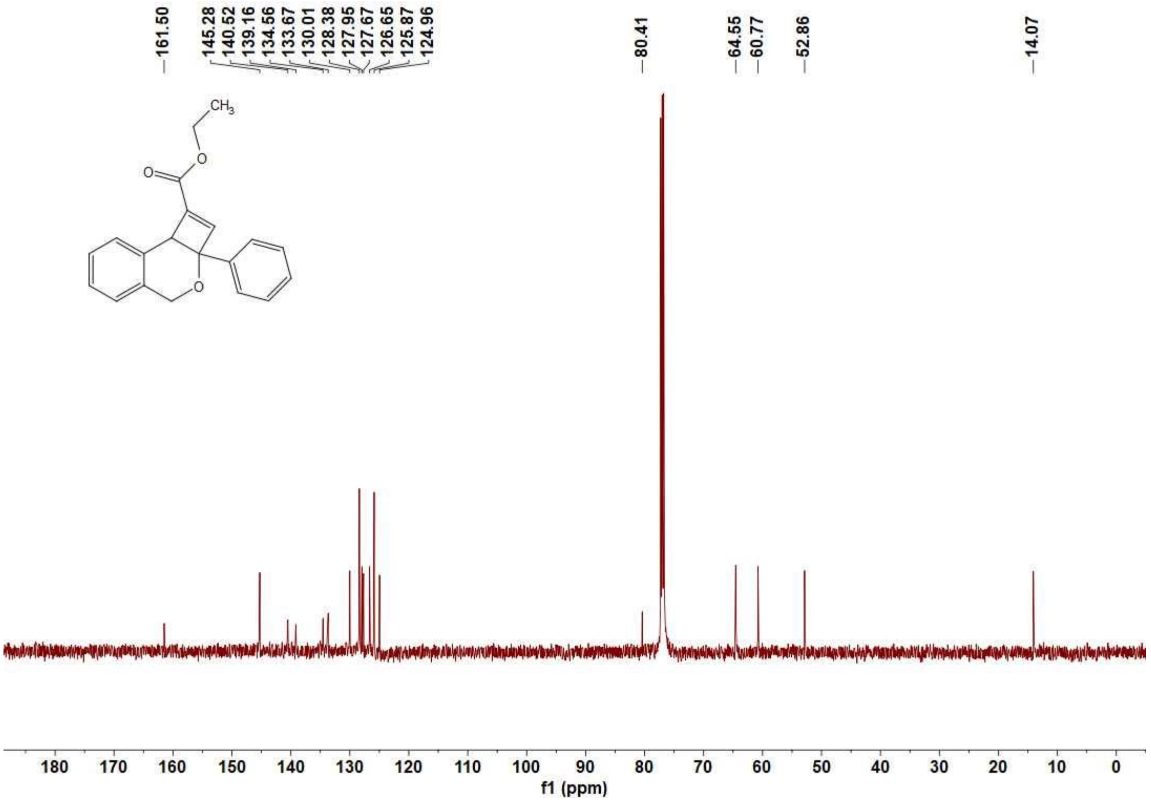

**Ethyl 3-phenyl-3,6-dihydro-1*H*-3,6-epidioxybenzo[*c*]oxocine-5-carboxylate (1c)**

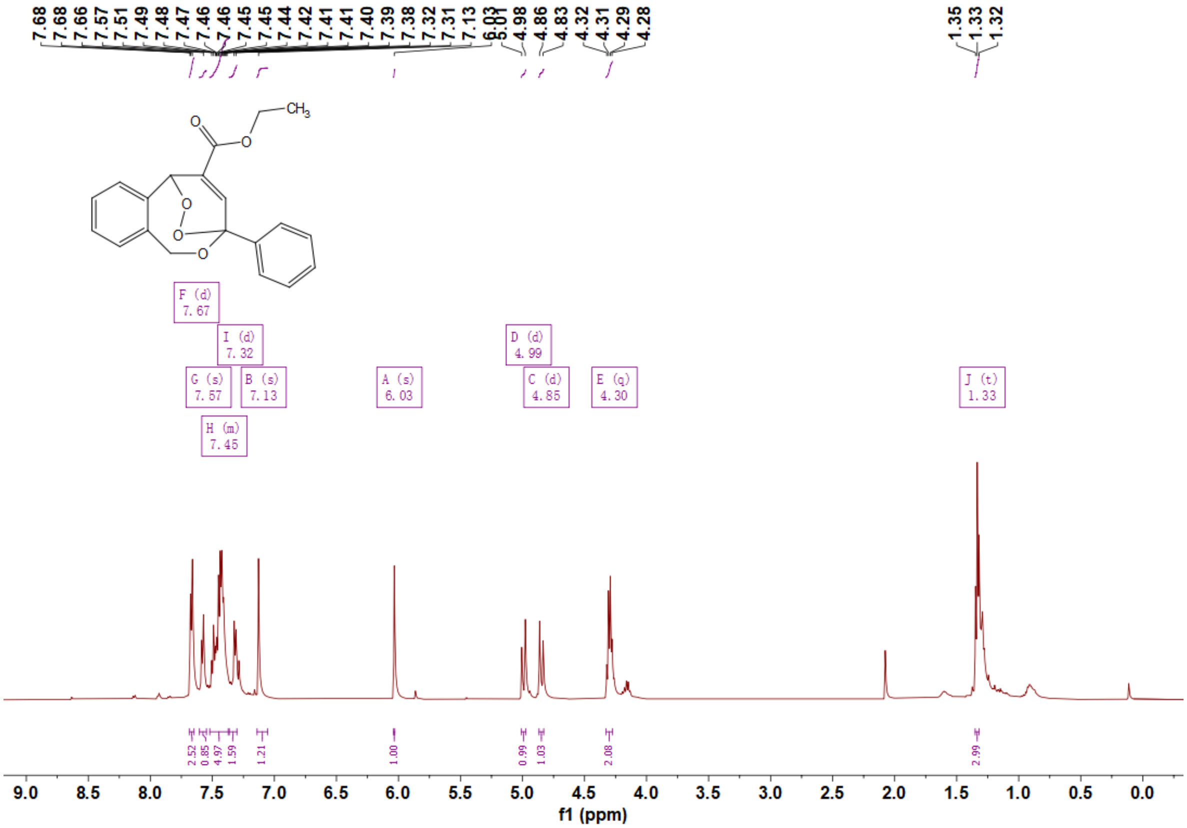


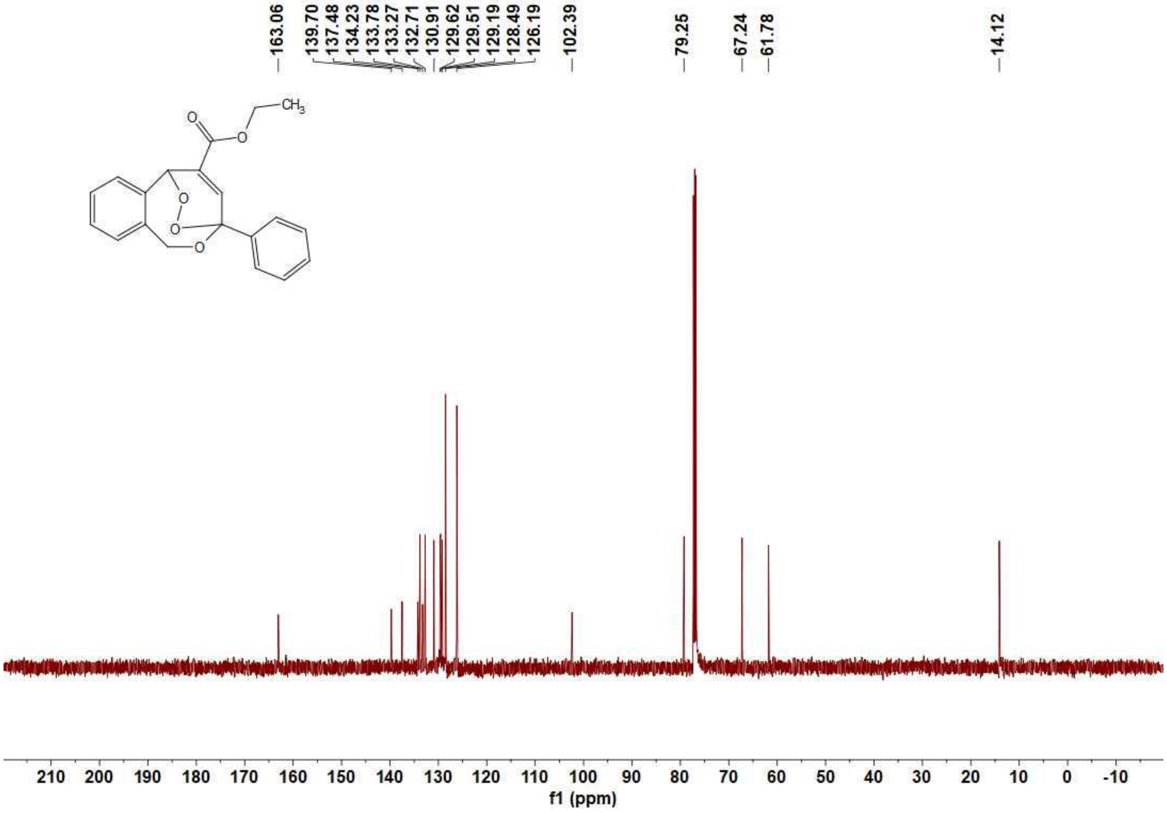

**Ethyl 2a-(2,4-dimethylphenyl)-2a,8b-dihydro-4*H*-cyclobuta[*c*]isochromene-1-carboxylate (2b)**

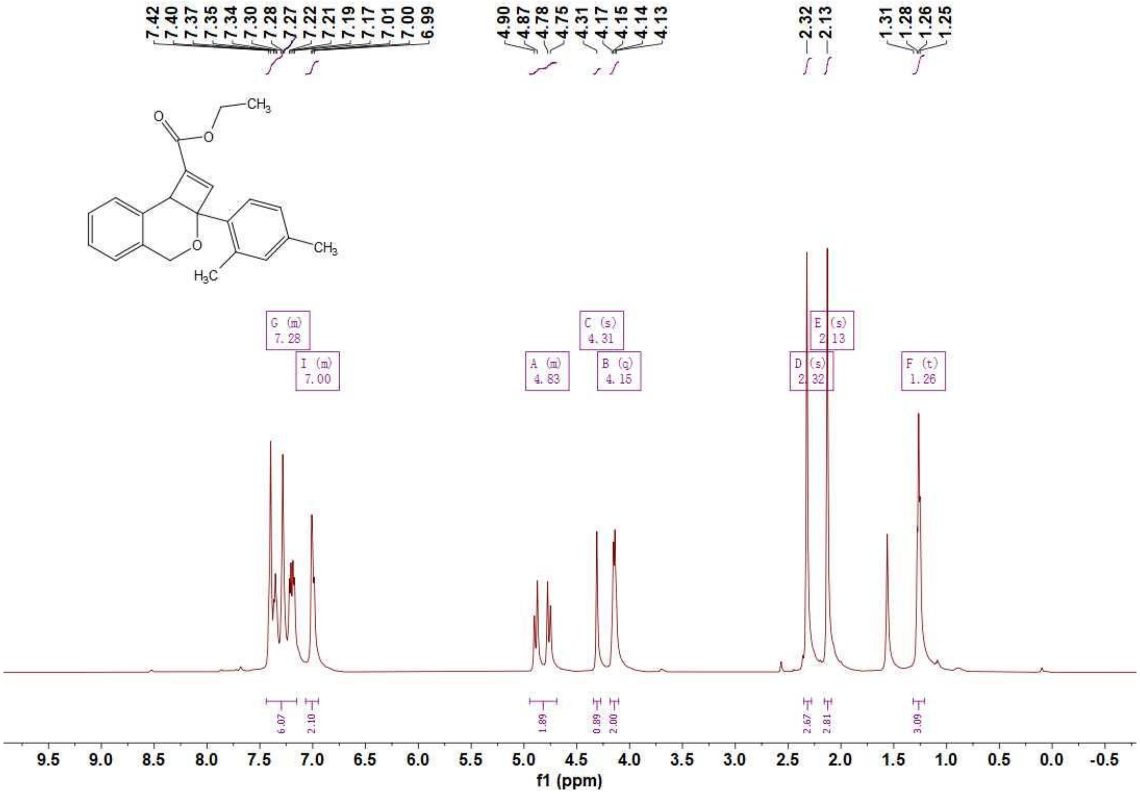


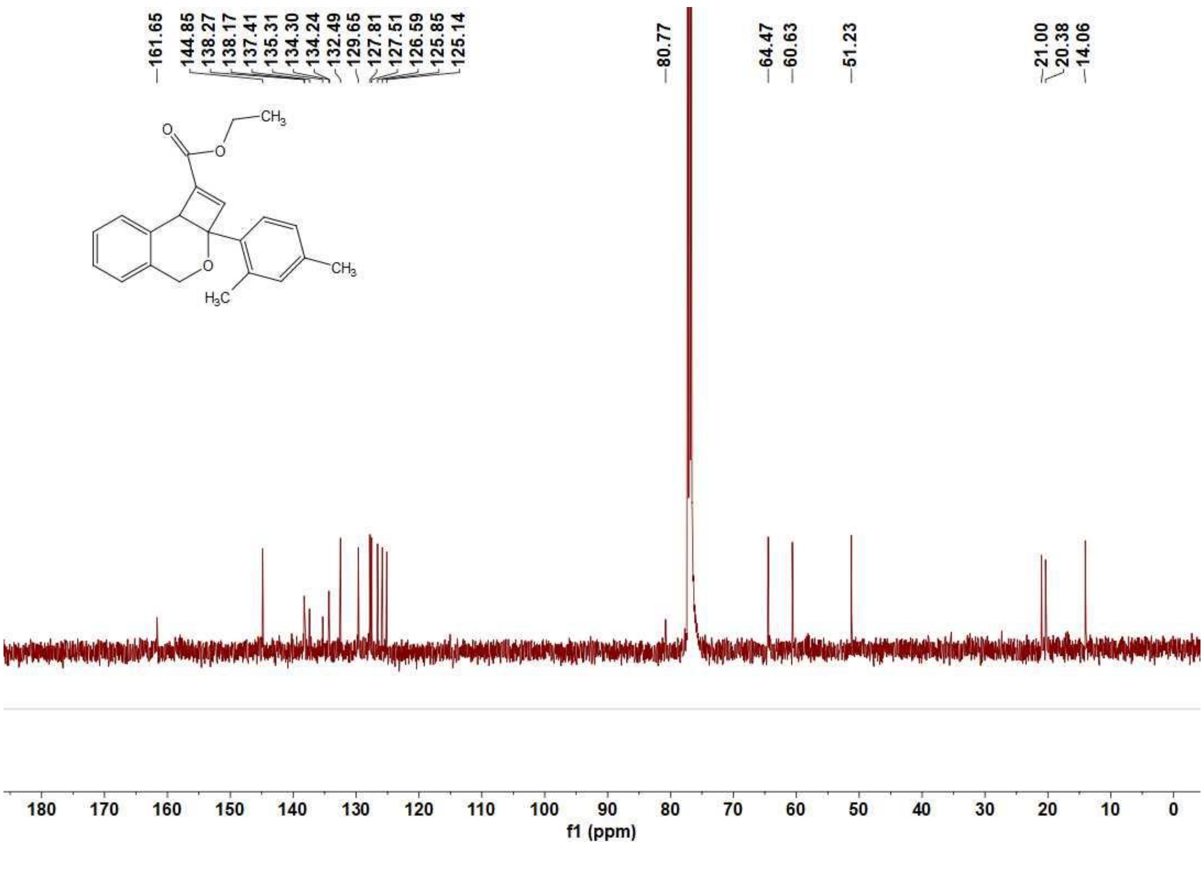

**Ethyl 2a-(3-methoxyphenyl)-2a,8b-dihydro-4*H*-cyclobuta[*c*]isochromene-1-carboxylate (3b)**

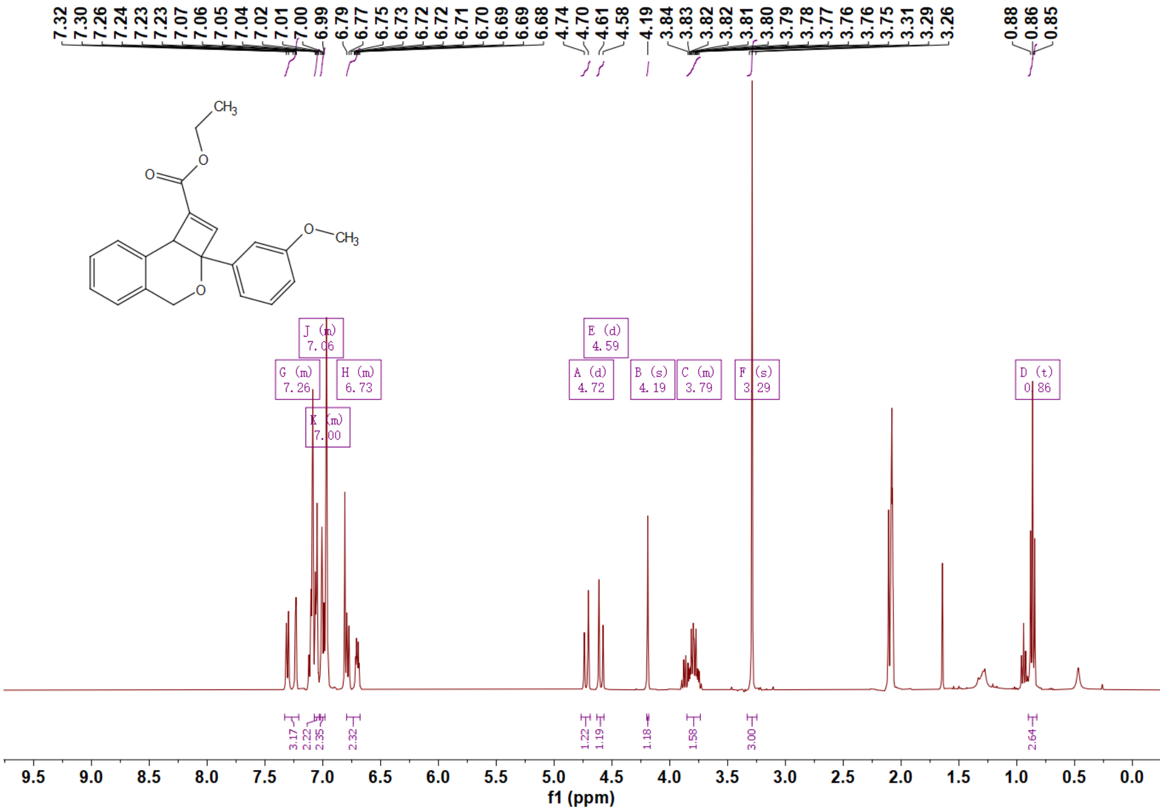

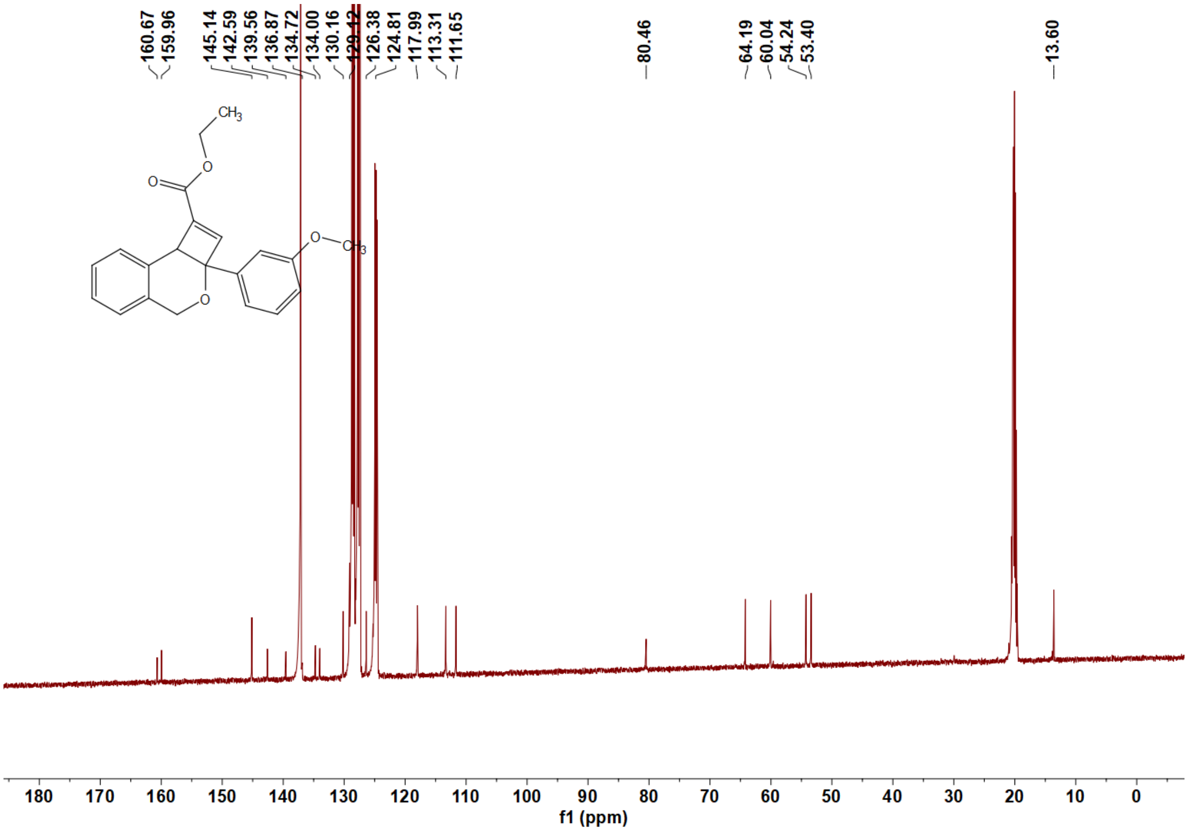

**Ethyl 2a-(2,3-dihydrobenzo[*b*][1,4]dioxin-6-yl)-2a,8b-dihydro-4*H*-cyclobuta[*c*]isochromene-1-carboxylate (4b)**

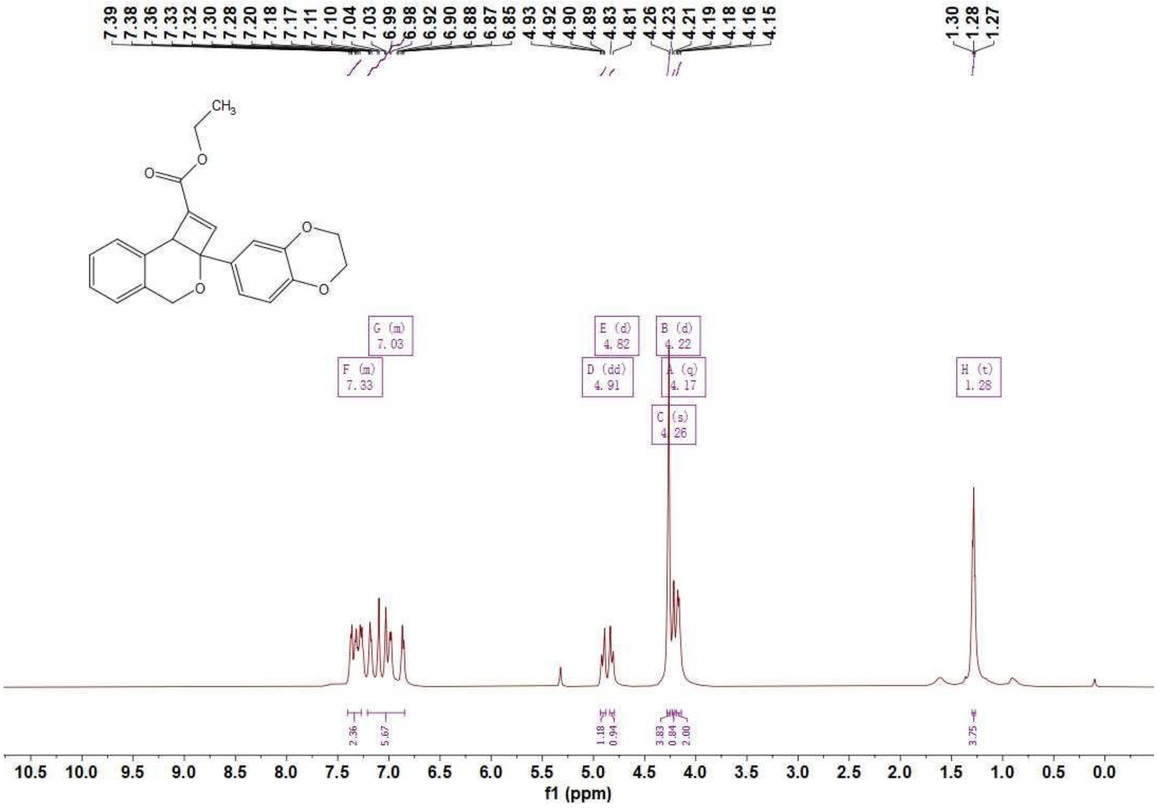


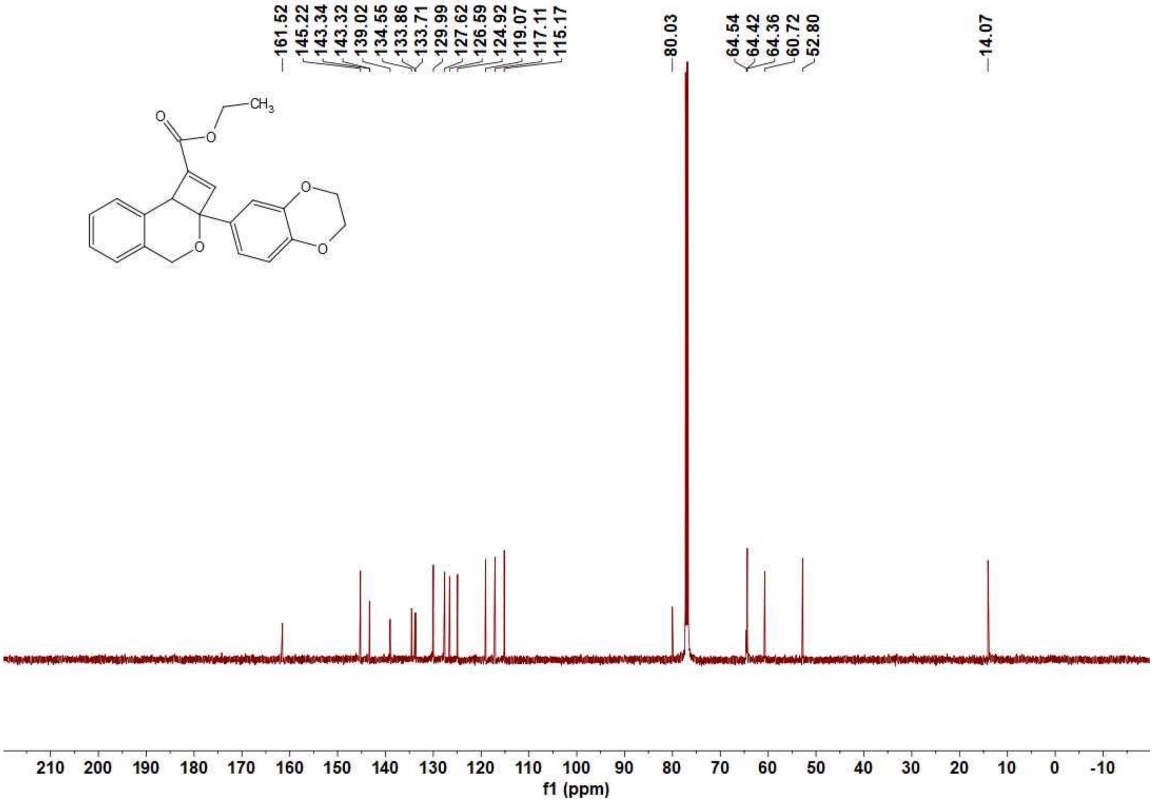

**Ethyl 2a-(naphthalen-1-yl)-2a,8b-dihydro-4*H*-cyclobuta[*c*]isochromene-1-carboxylate (5b)**

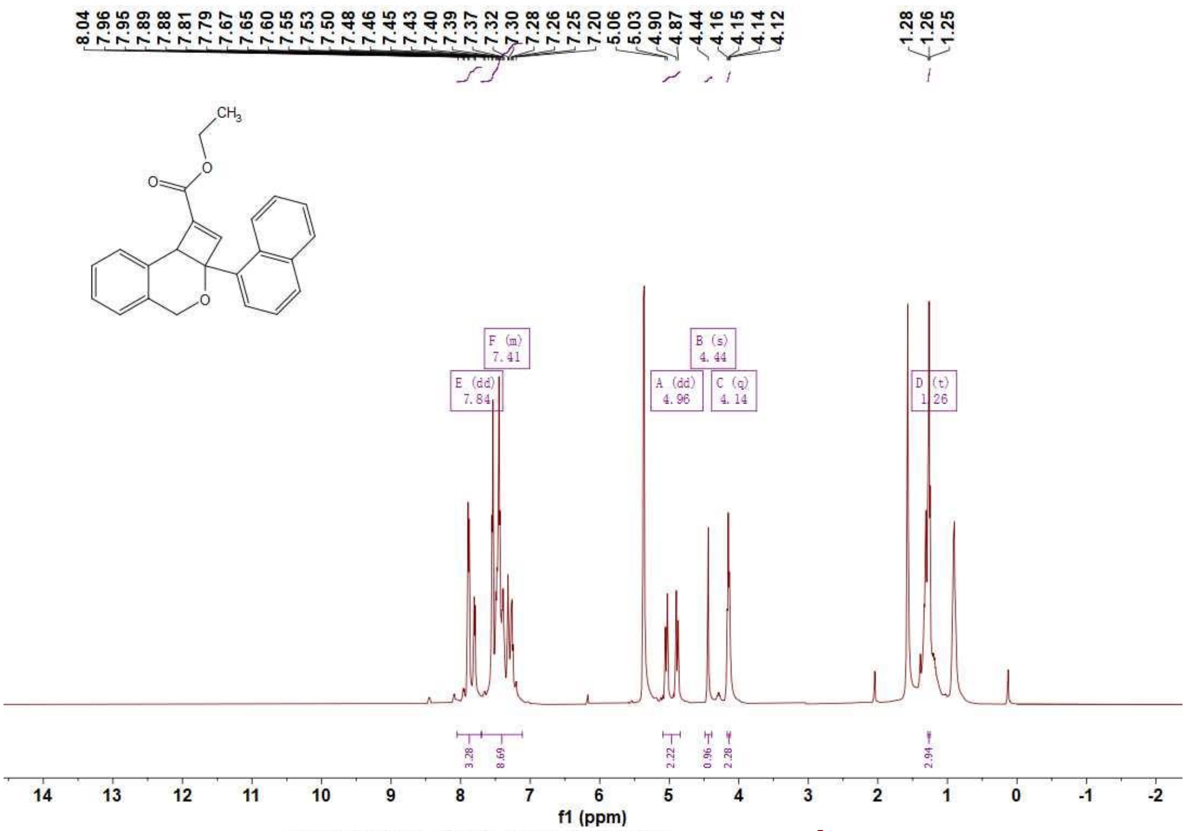


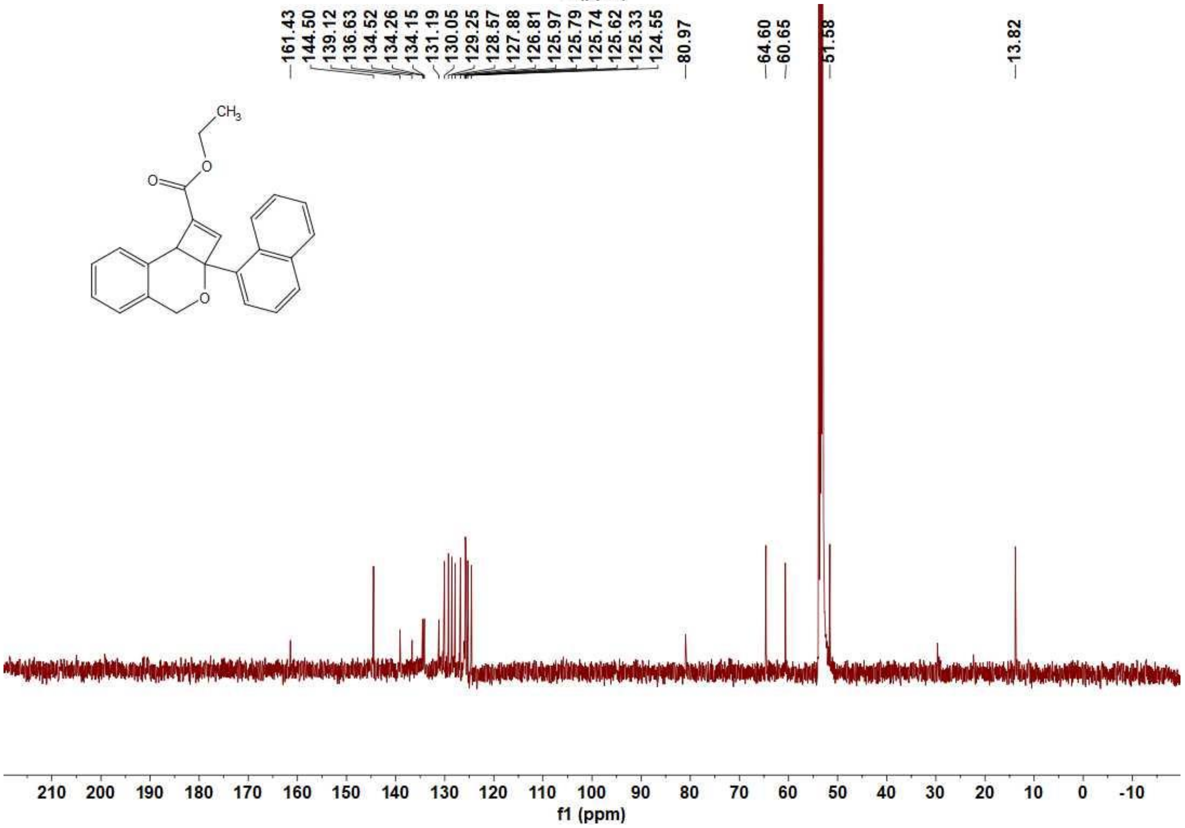

**Ethyl 2a-(3,5-dimethoxyphenyl)-2a,8b-dihydro-4*H*-cyclobuta[*c*]isochromene-1-carboxylate (6b)**

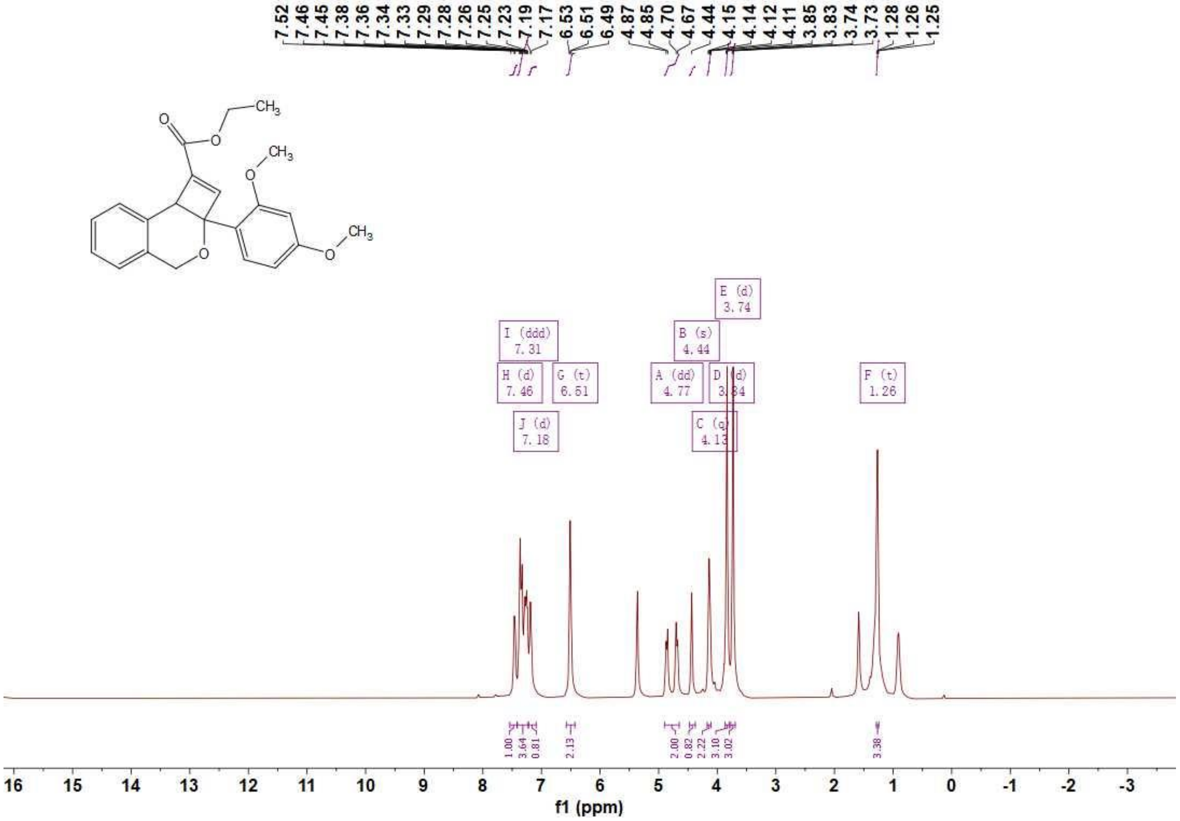


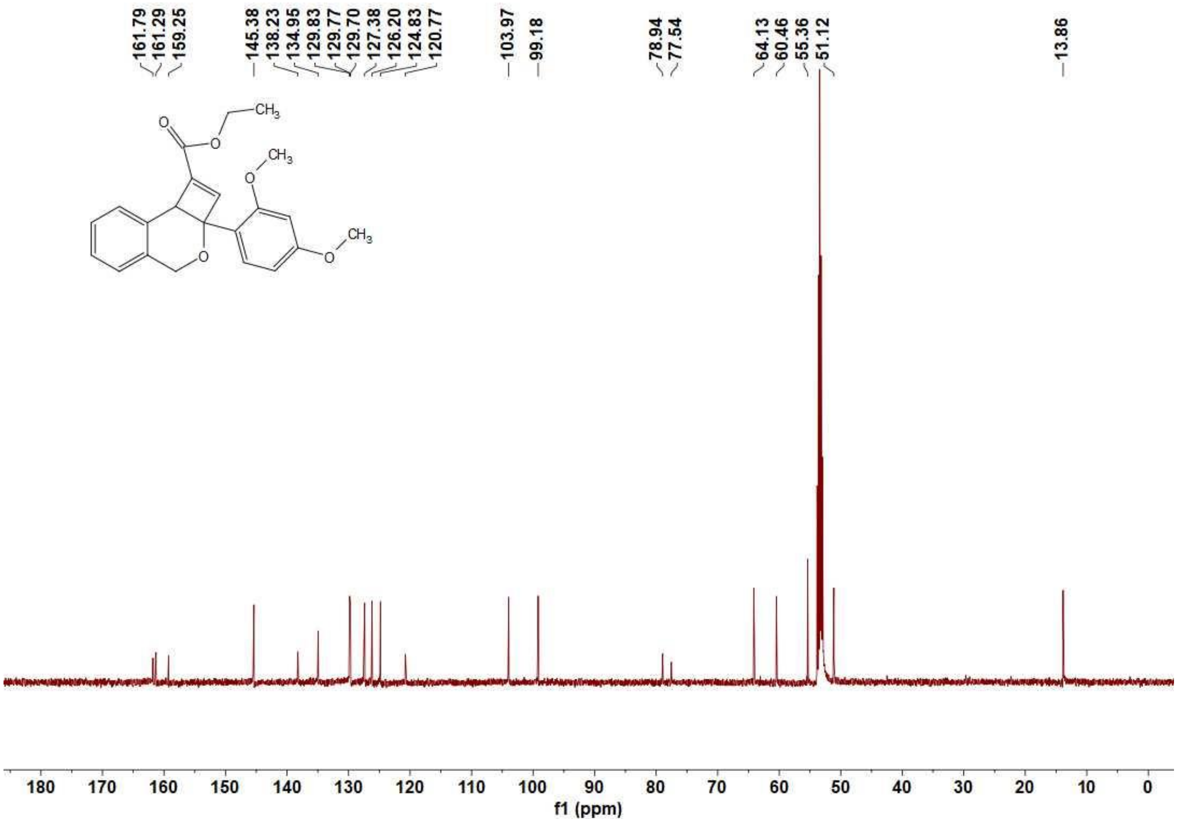

**Ethyl 2a-(3-bromophenyl)-2a,8b-dihydro-4*H*-cyclobuta[*c*]isochromene-1-carboxylate (7b)**

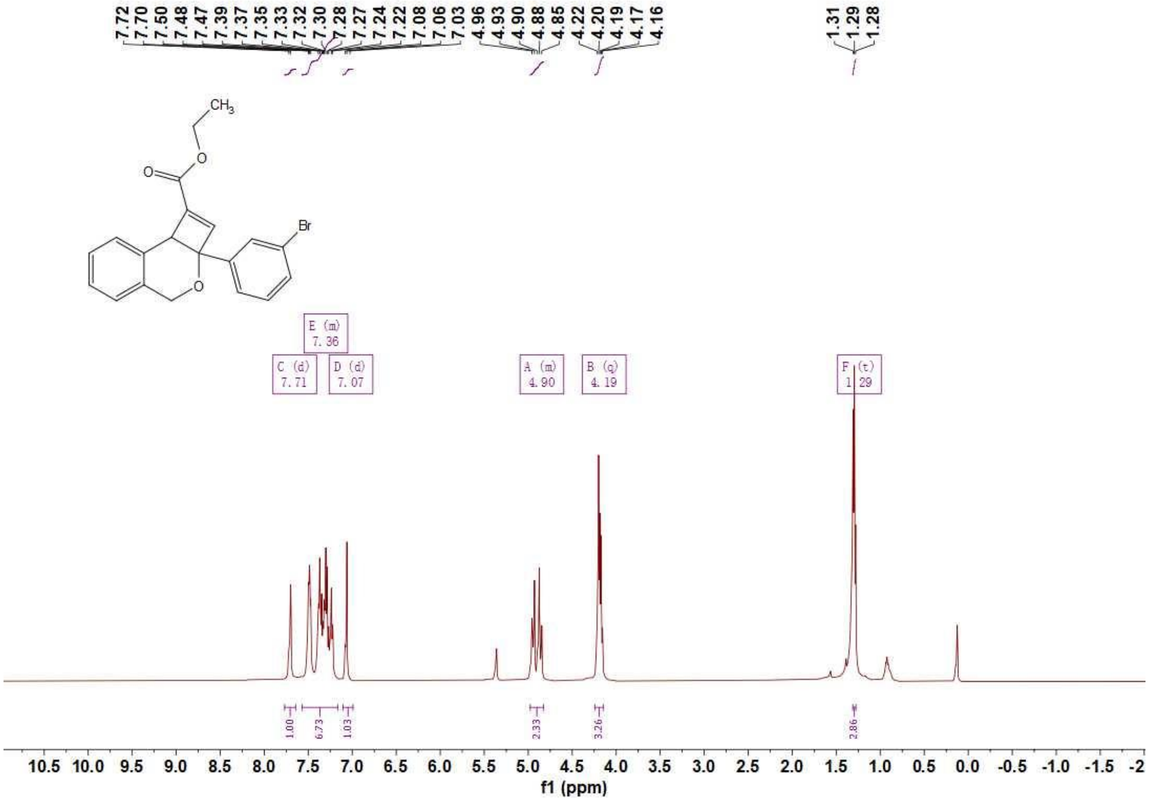

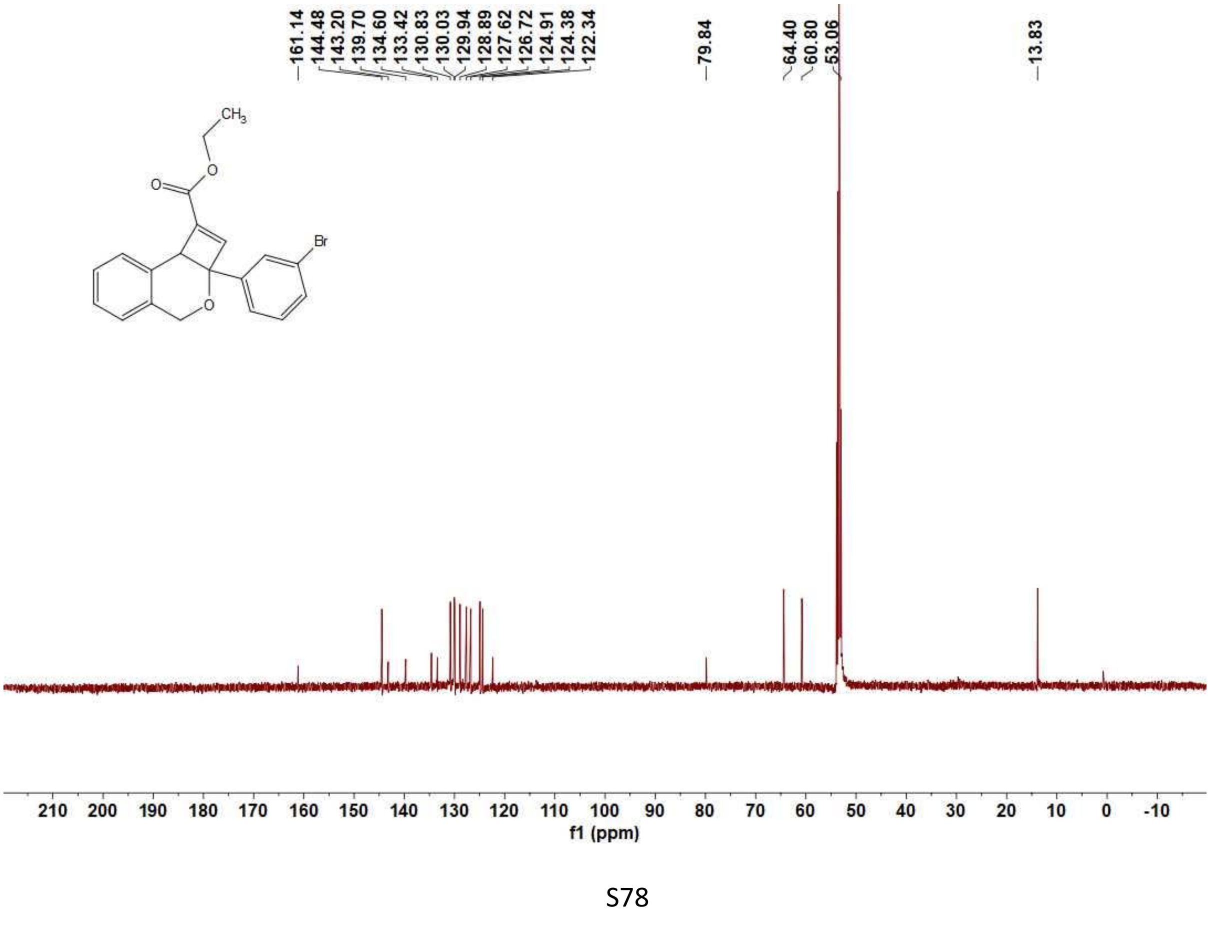

**Ethyl 2a-(2-fluorophenyl)-2a,8b-dihydro-4*H*-cyclobuta[*c*]isochromene-1-carboxylate (8b)**

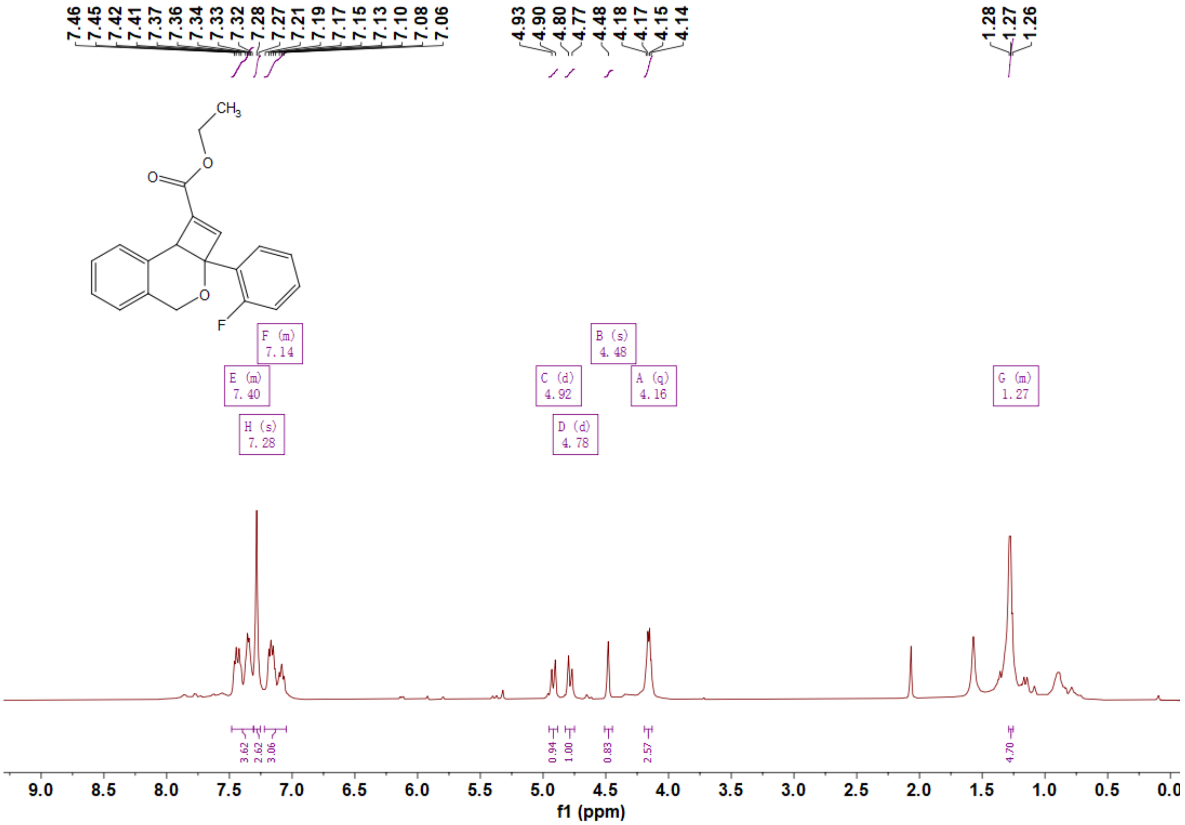


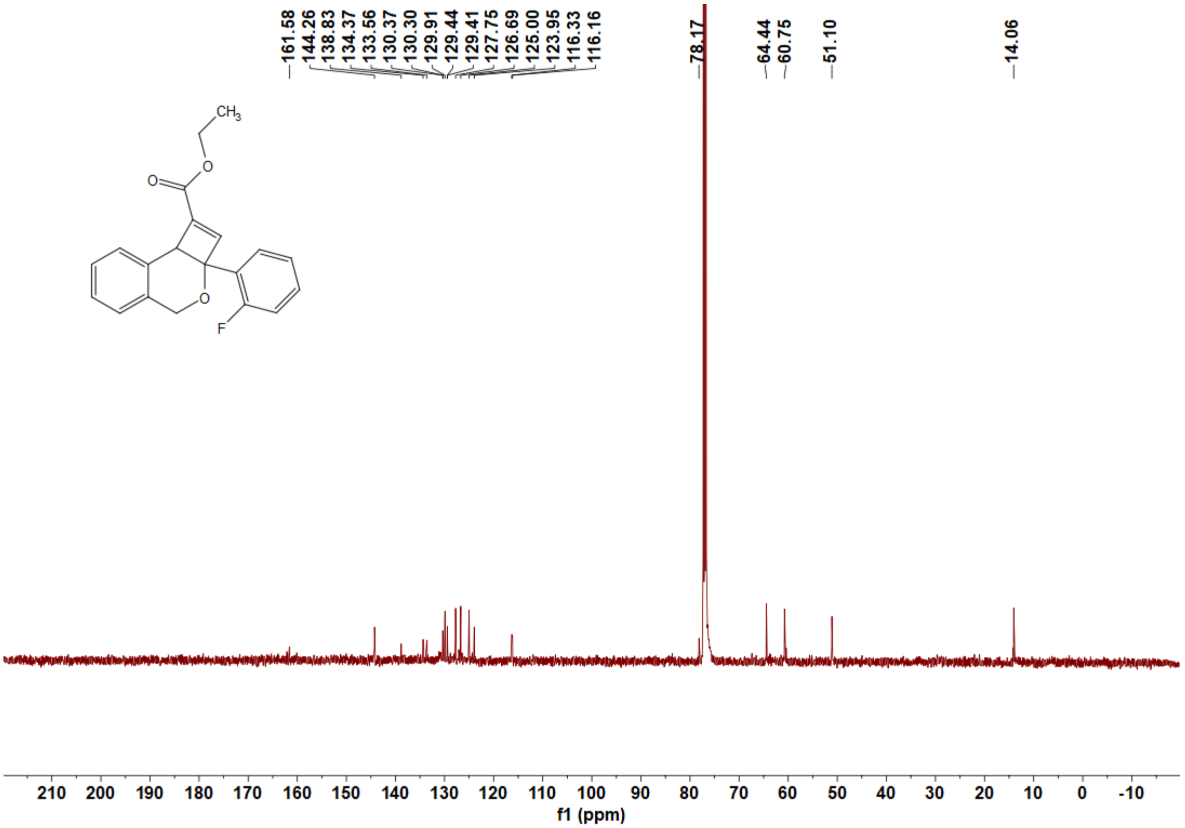

**Ethyl 2a-(3-chlorophenyl)-2a,8b-dihydro-4*H*-cyclobuta[*c*]isochromene-1-carboxylate (9b)**

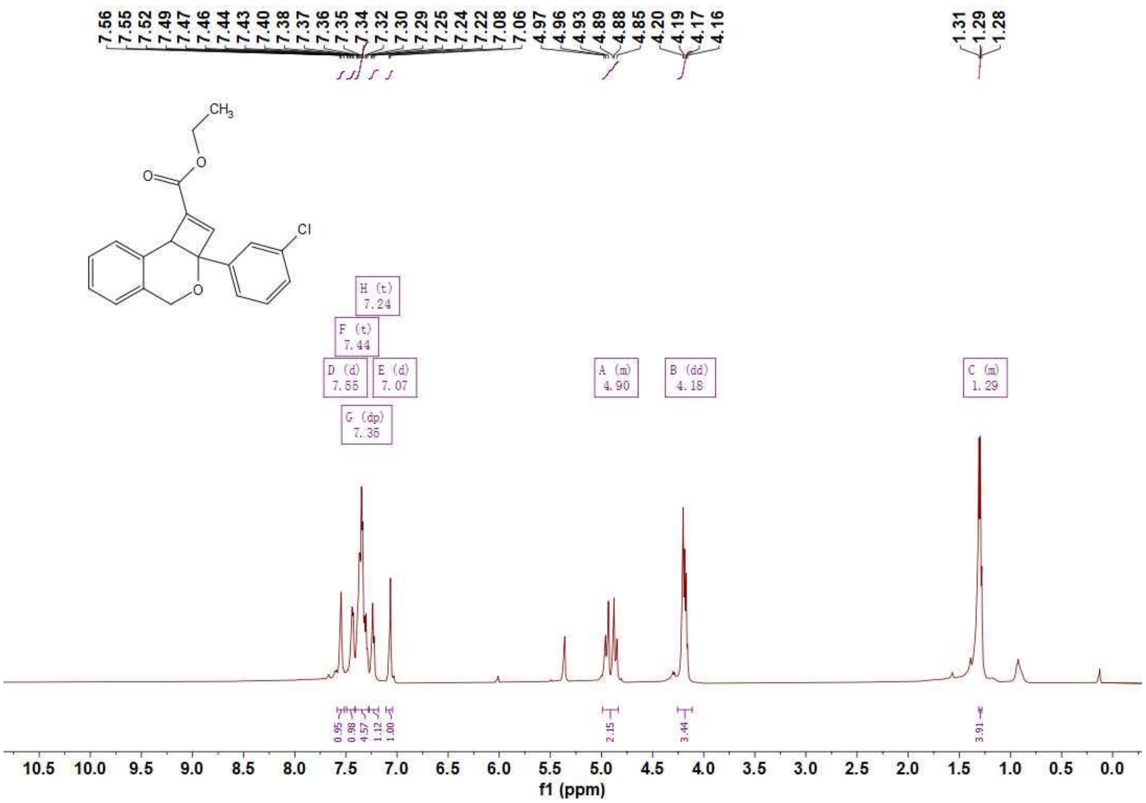


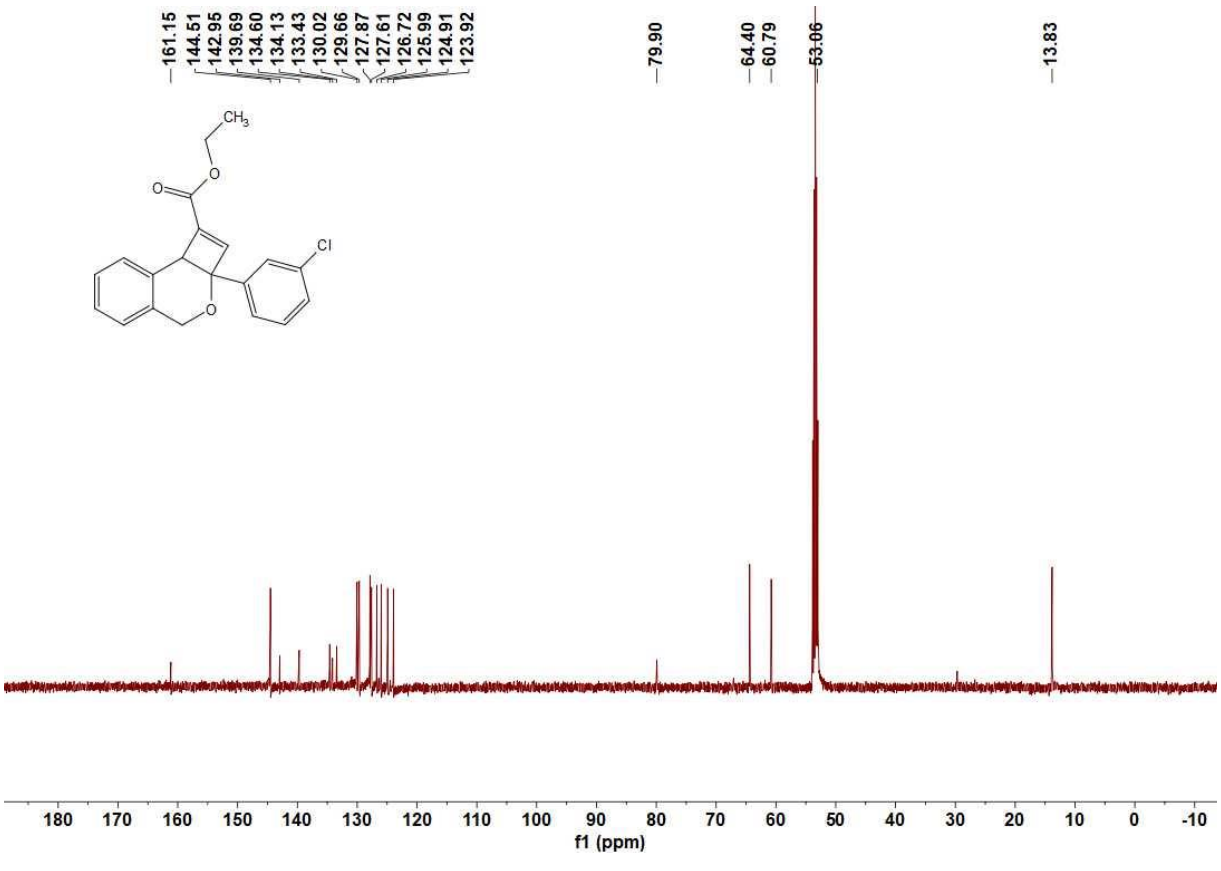

**Ethyl 2a-(4-chlorophenyl)-2a,8b-dihydro-4*H*-cyclobuta[*c*]isochromene-1-carboxylate (10b)**

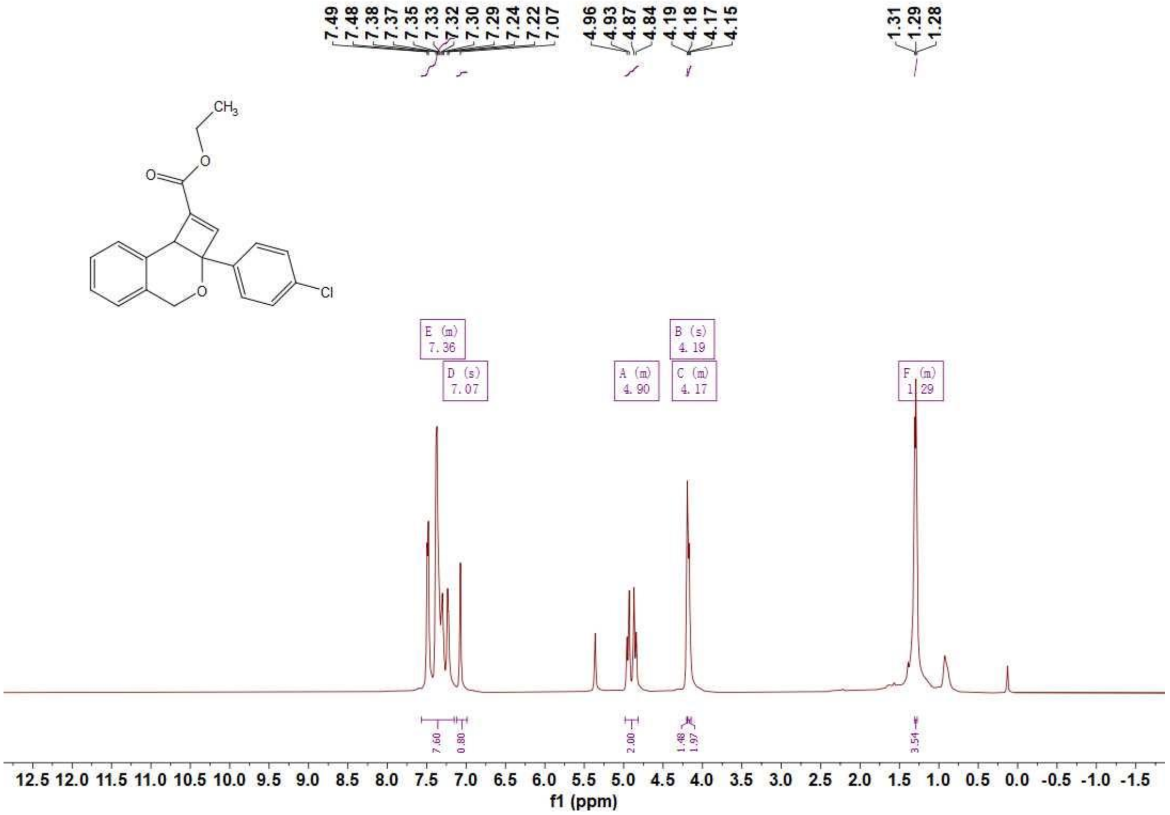


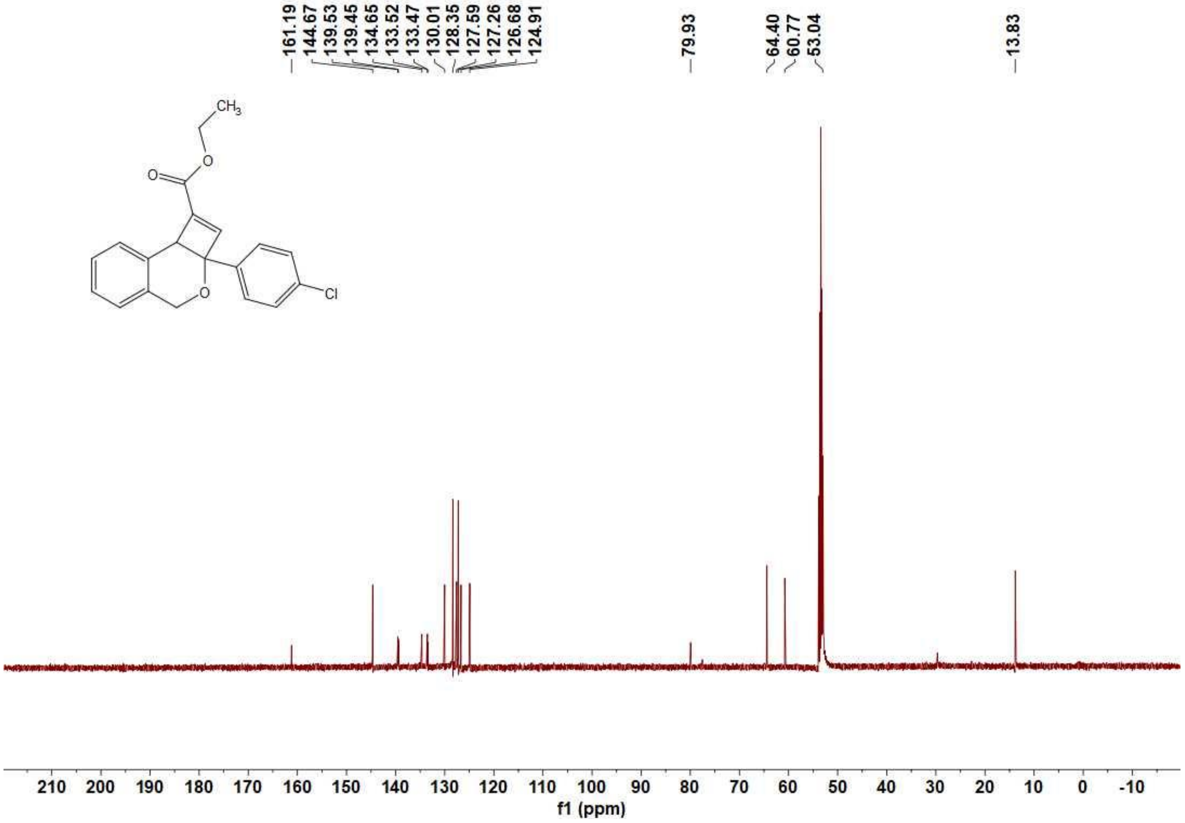

**Ethyl 2a-(4-(trifluoromethoxy)phenyl)-2a,8b-dihydro-4*H*-cyclobuta[*c*]isochromene-1-carboxylate (11b)**

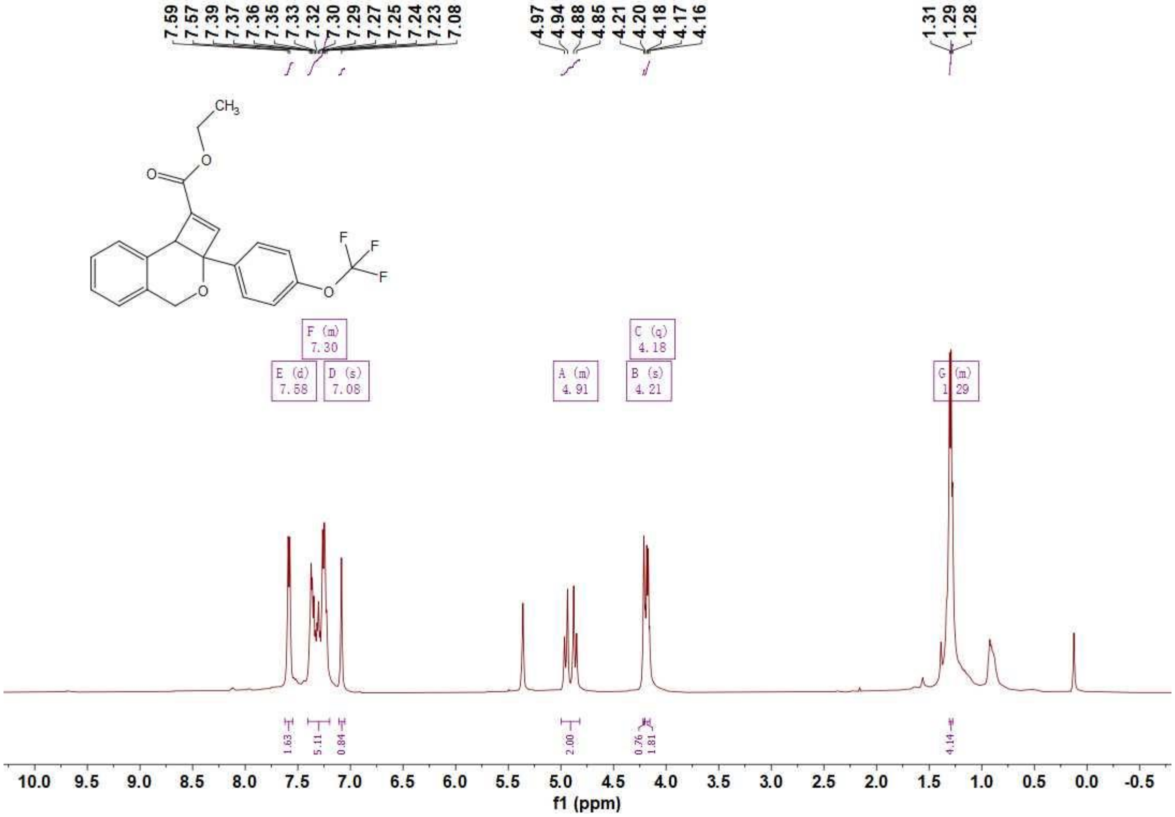


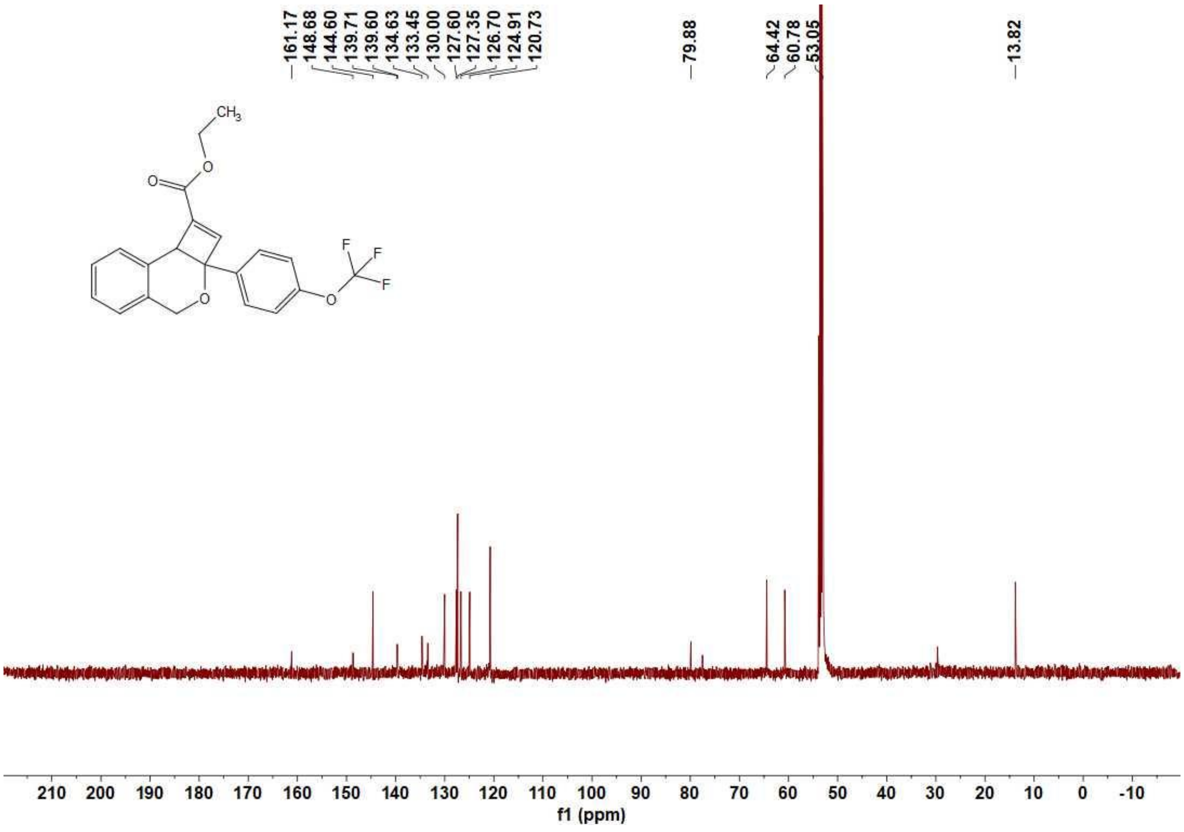

**Ethyl 2a-(3-(trifluoromethyl)phenyl)-2a,8b-dihydro-4*H*-cyclobuta[*c*]isochromene-1-carboxylate (12b)**

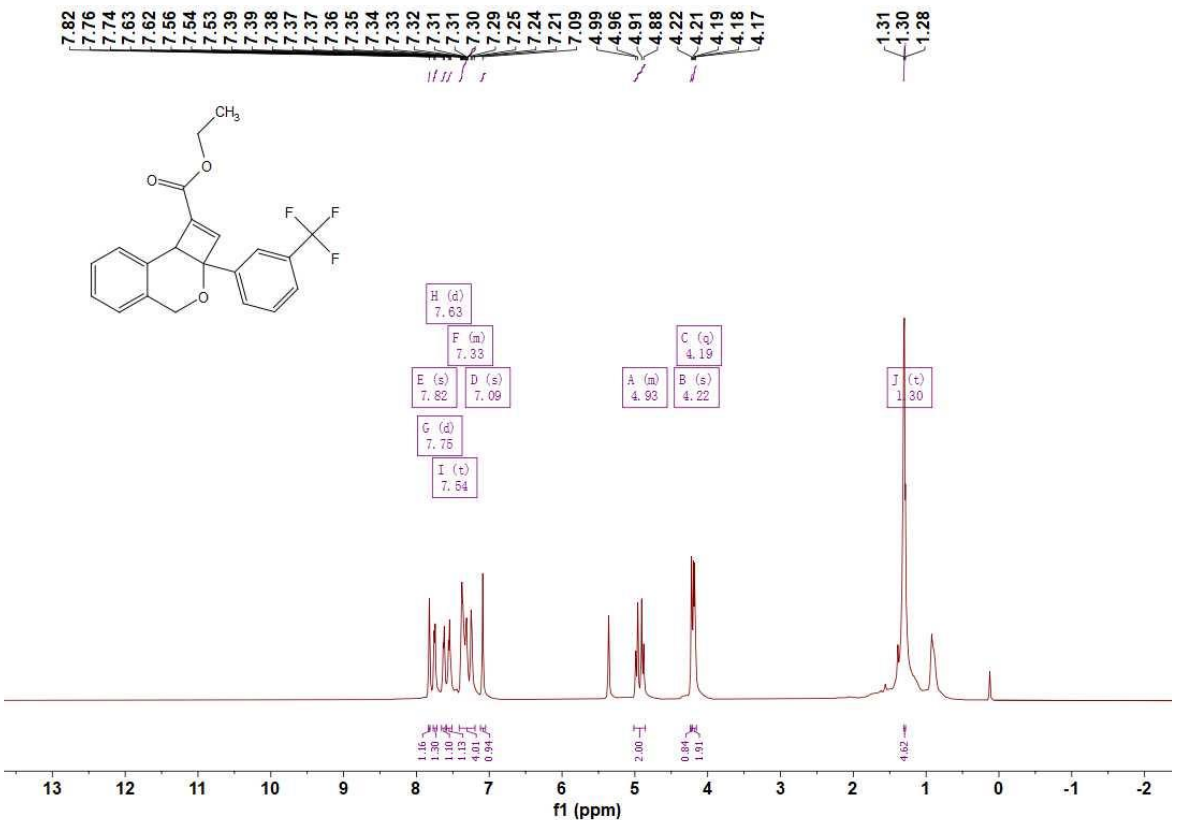


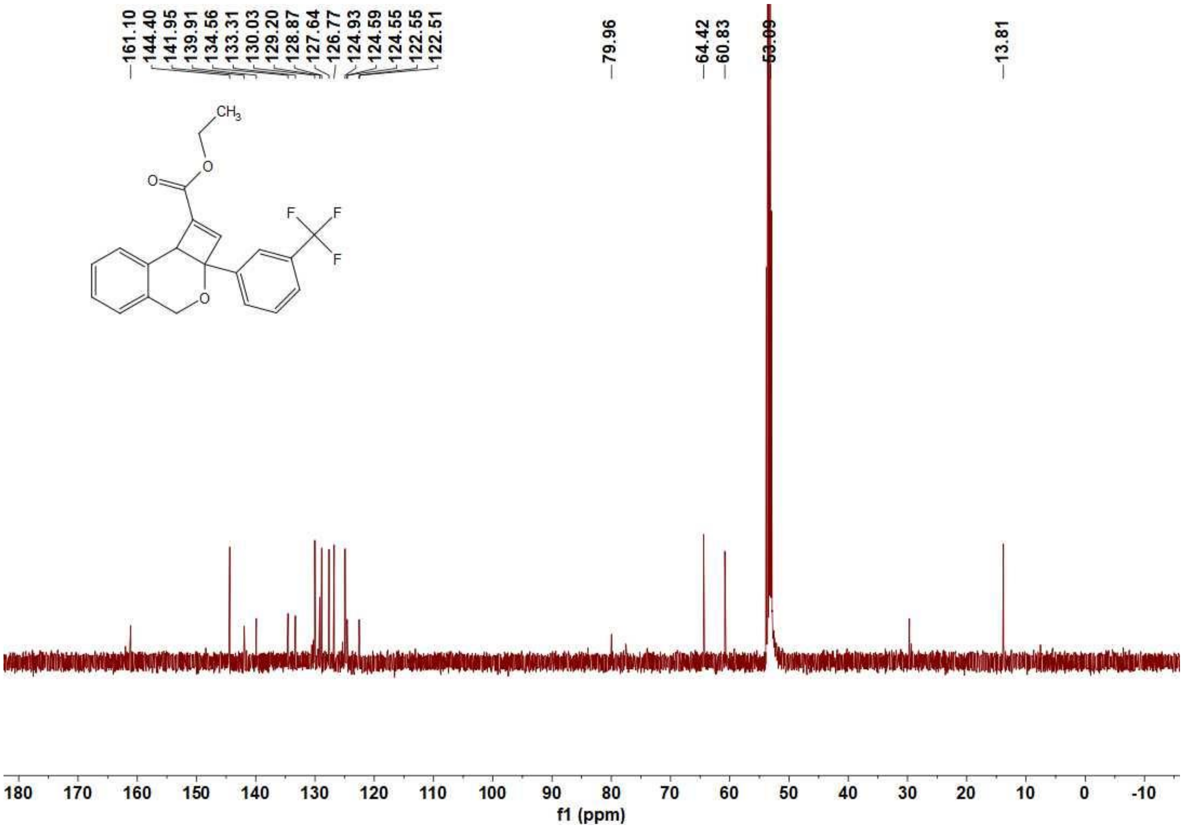

**Ethyl 2a-methyl-2a,8b-dihydro-4*H*-cyclobuta[*c*]isochromene-1-carboxylate (13b)**

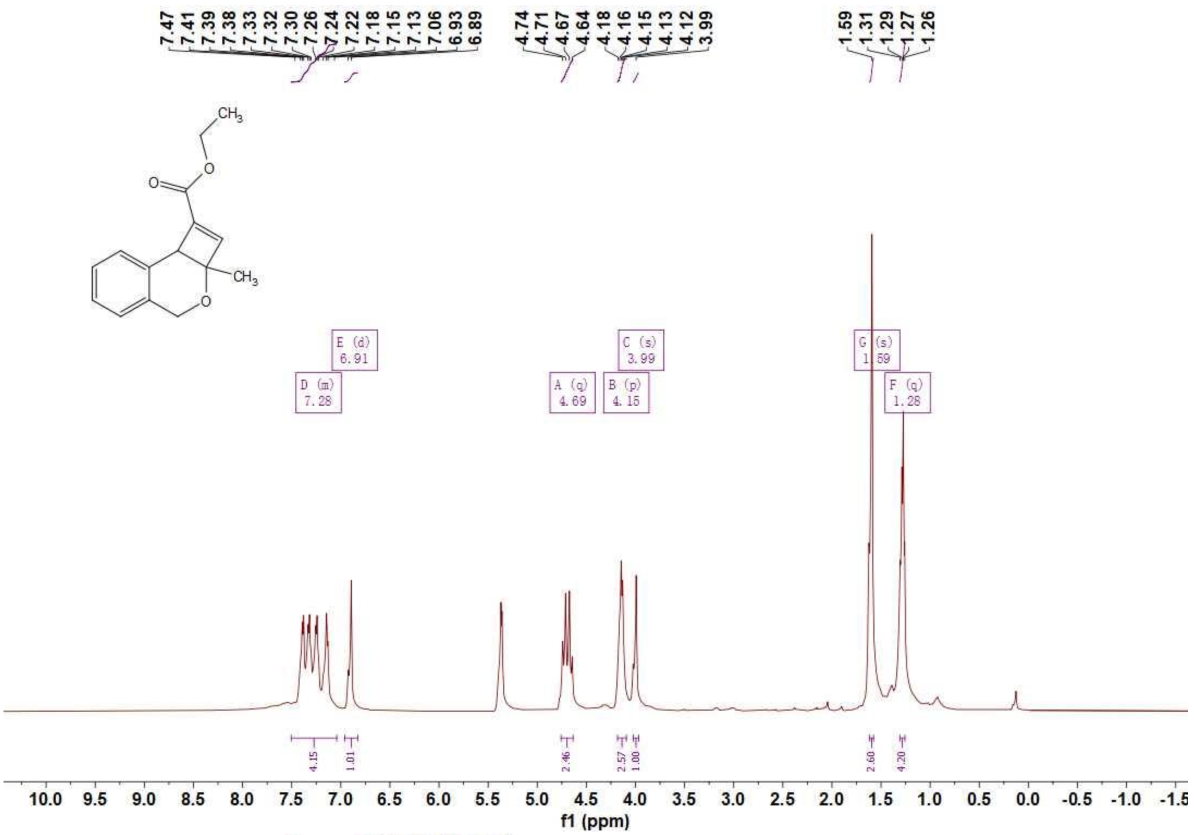


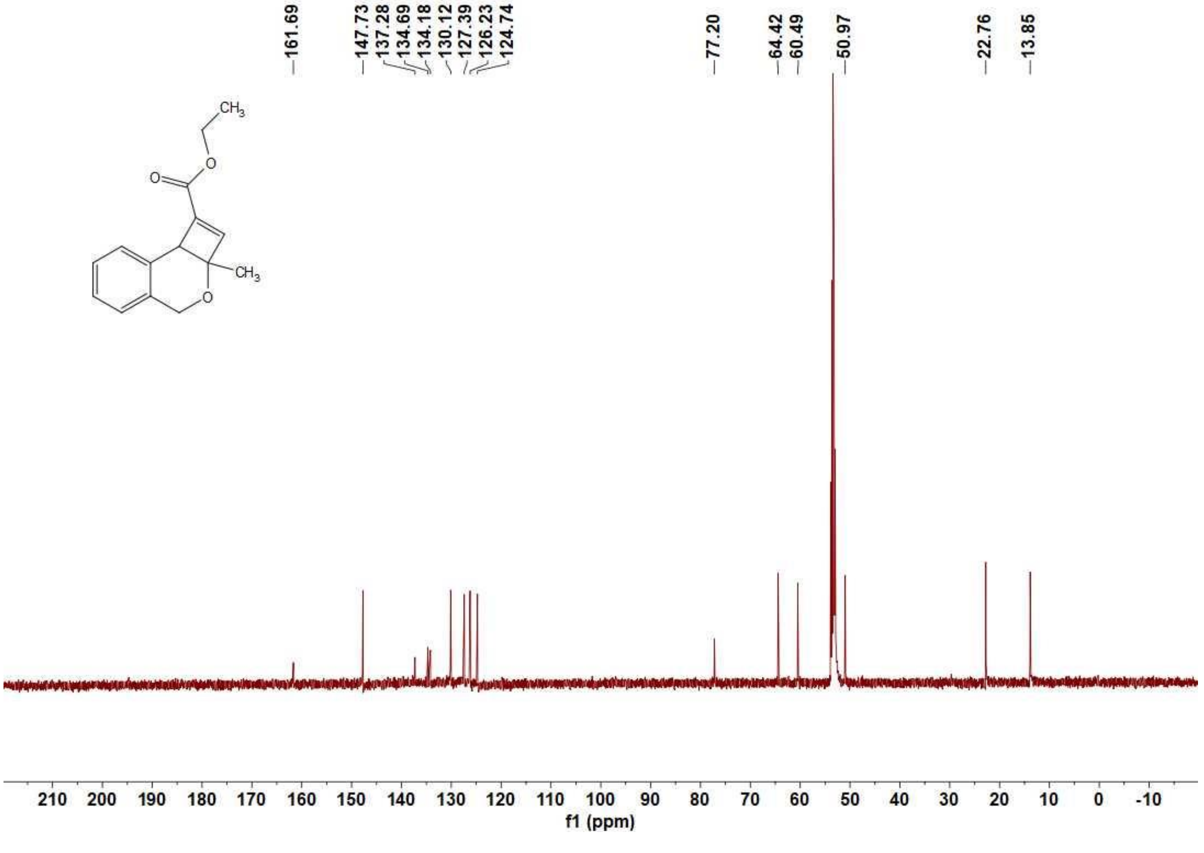

**Ethyl 2a-ethyl-2a,8b-dihydro-4*H*-cyclobuta[*c*]isochromene-1-carboxylate (14b)**

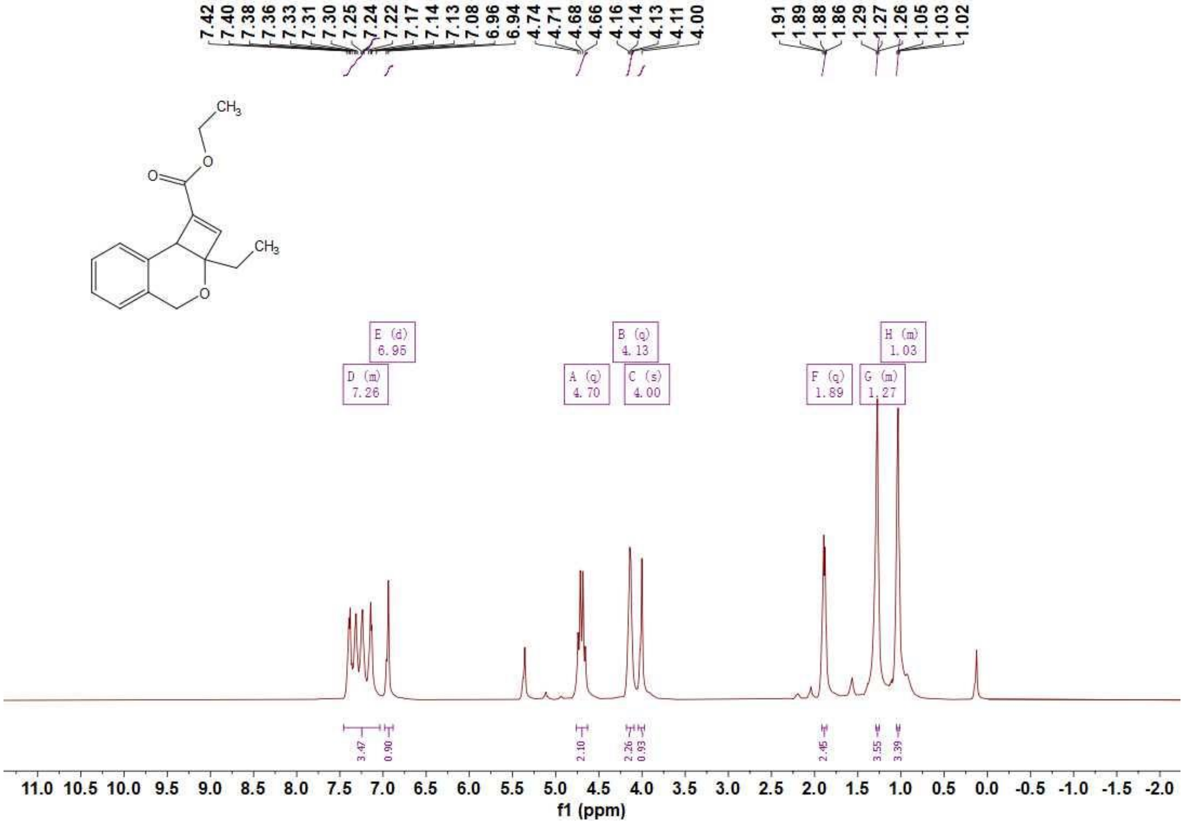


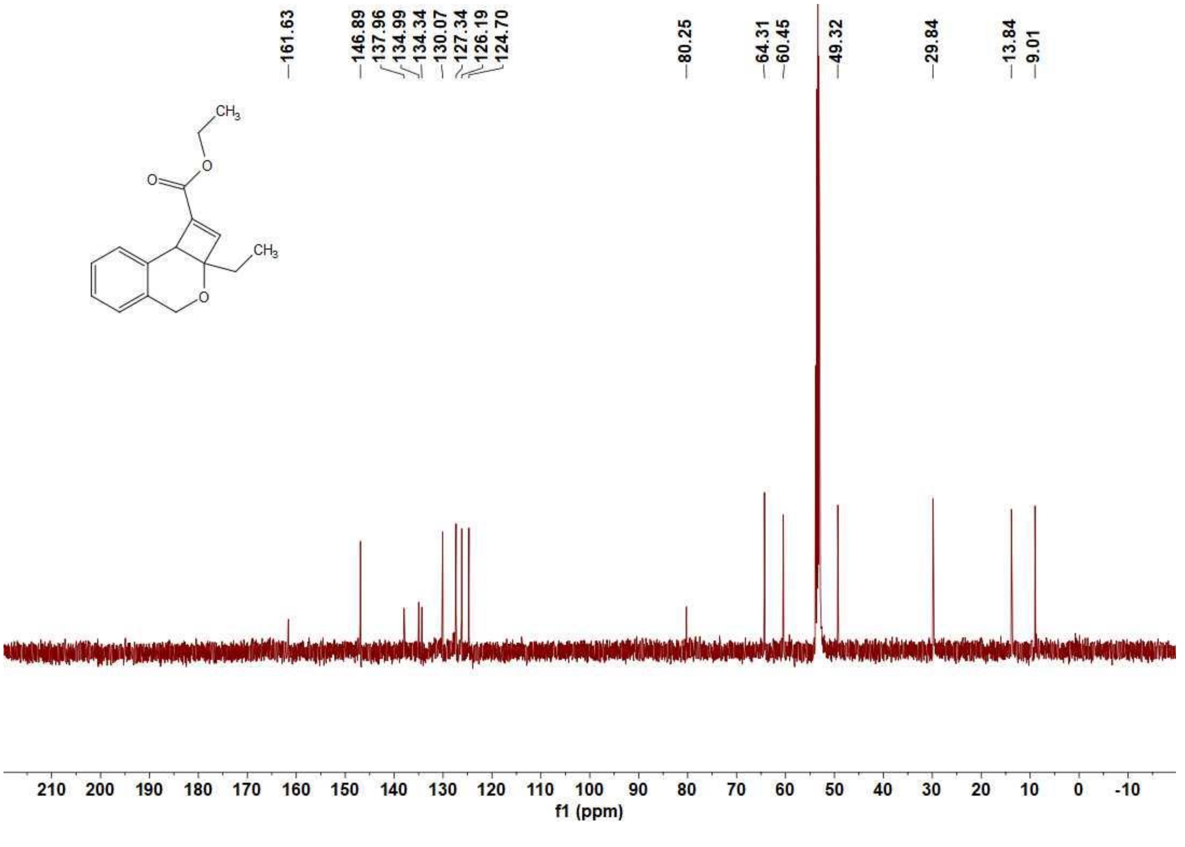

**Ethyl 2a-isopropyl-2a,8b-dihydro-4*H*-cyclobuta[*c*]isochromene-1-carboxylate (15b)**

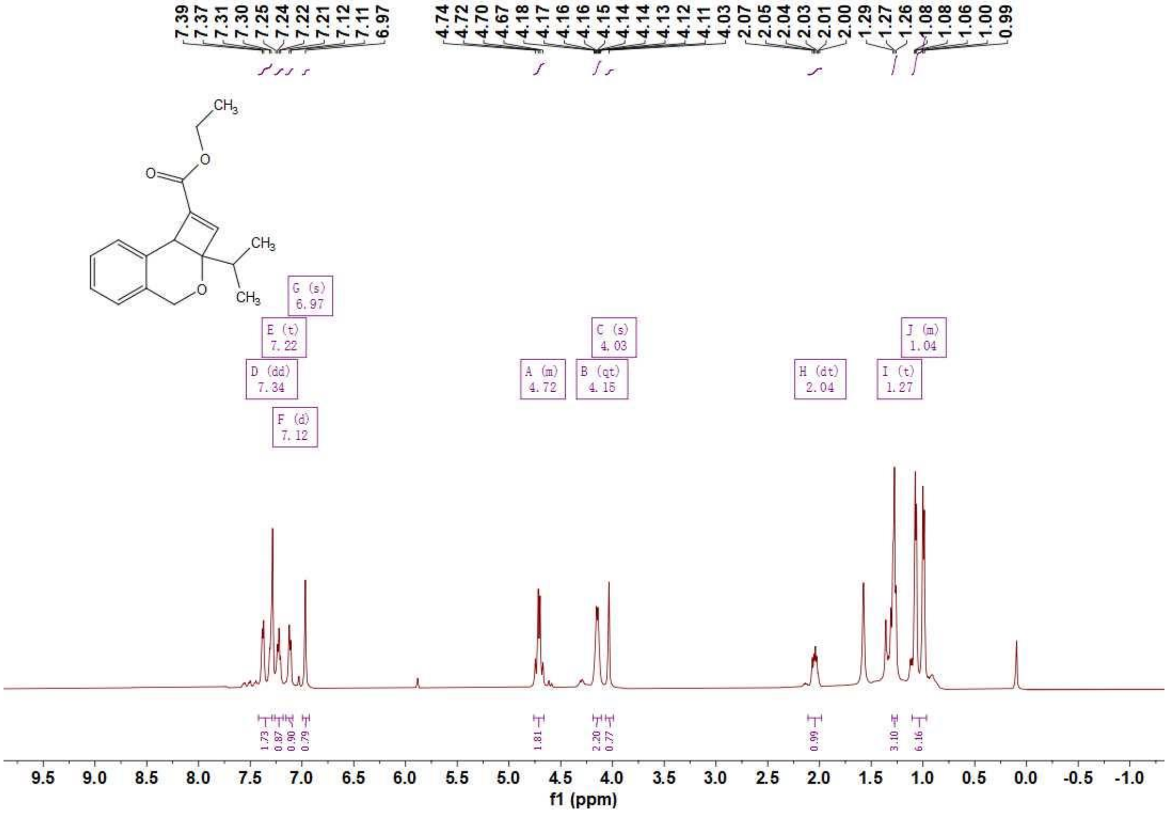


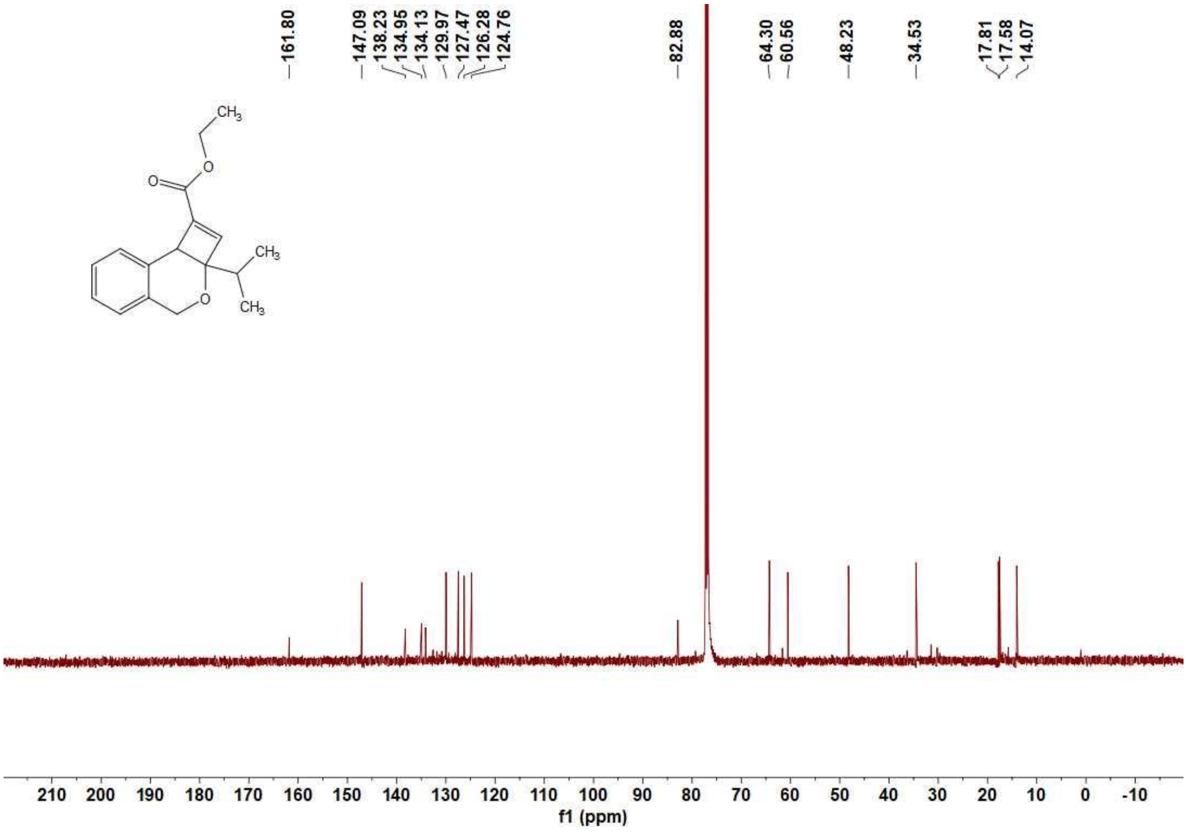

**Ethyl 2a-(tert-butyl)-2a,8b-dihydro-4*H*-cyclobuta[*c*]isochromene-1-carboxylate (16b)**

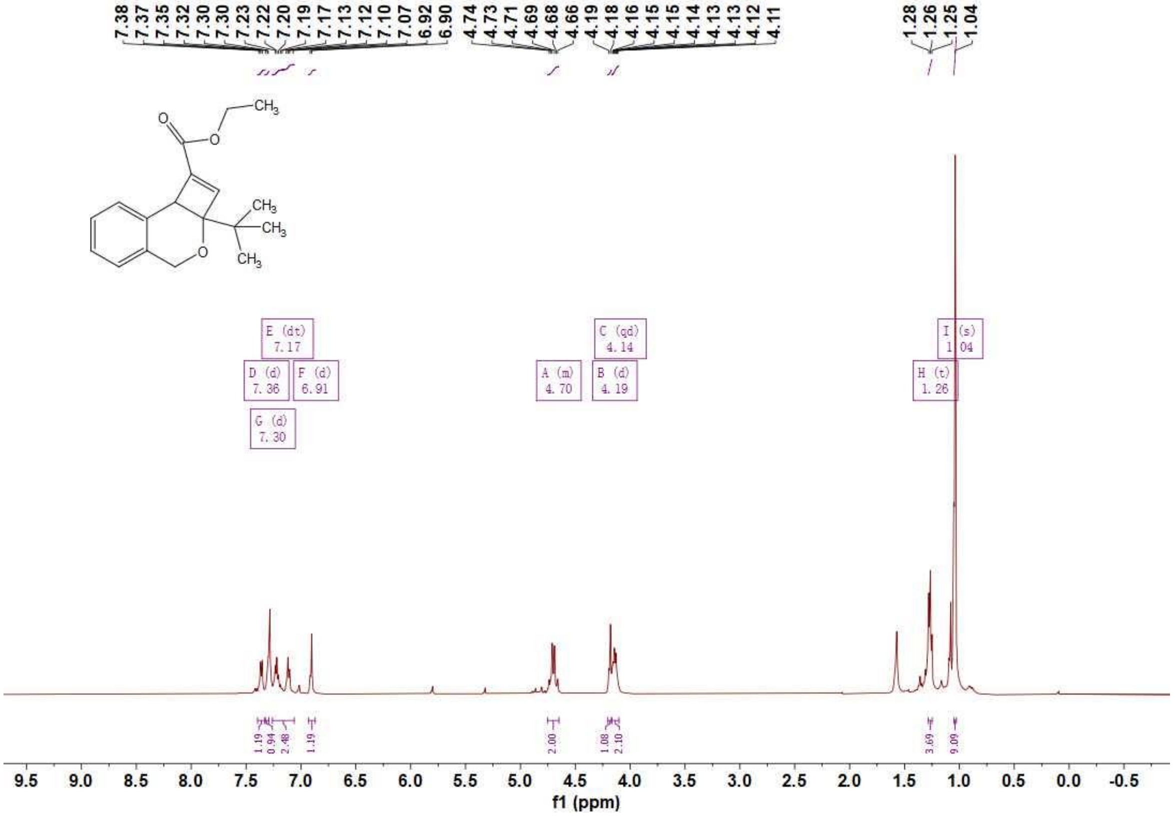


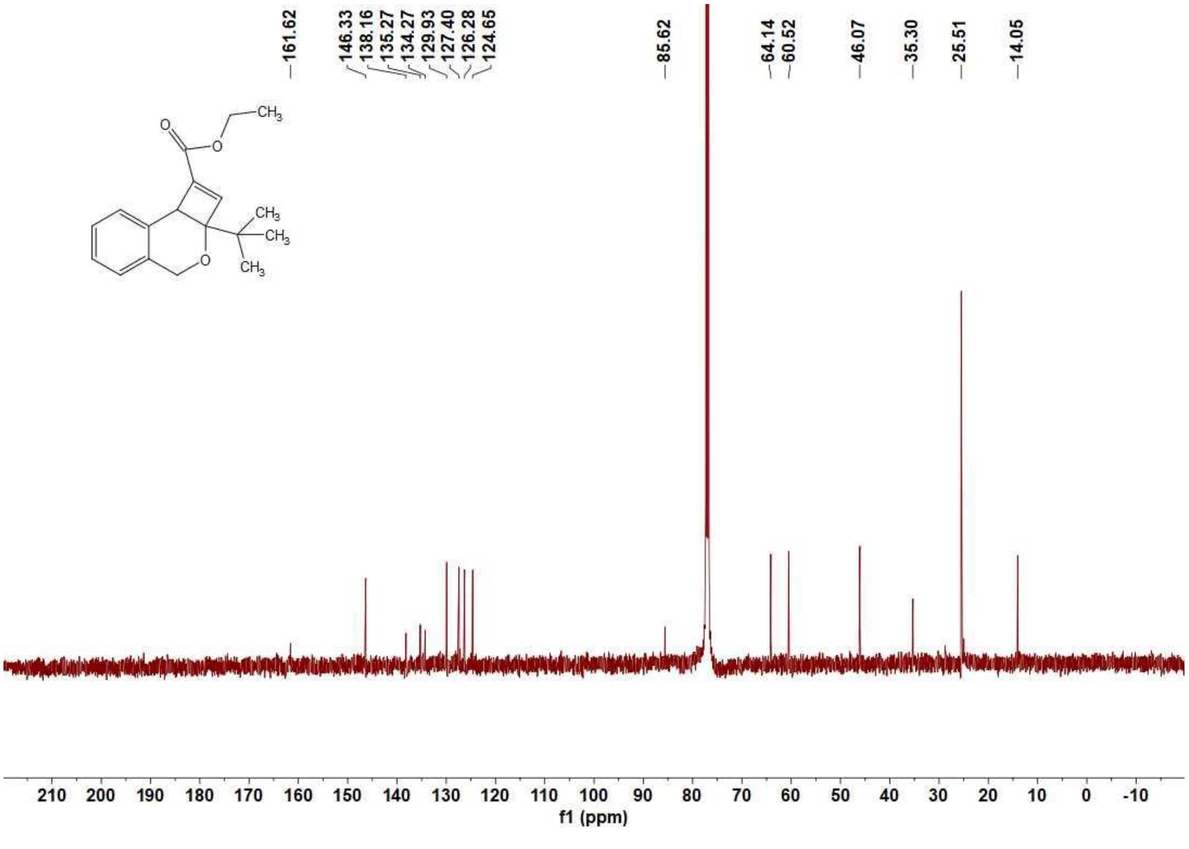

**Ethyl 2a-cyclopropyl-2a,8b-dihydro-4*H*-cyclobuta[*c*]isochromene-1-carboxylate (17b)**

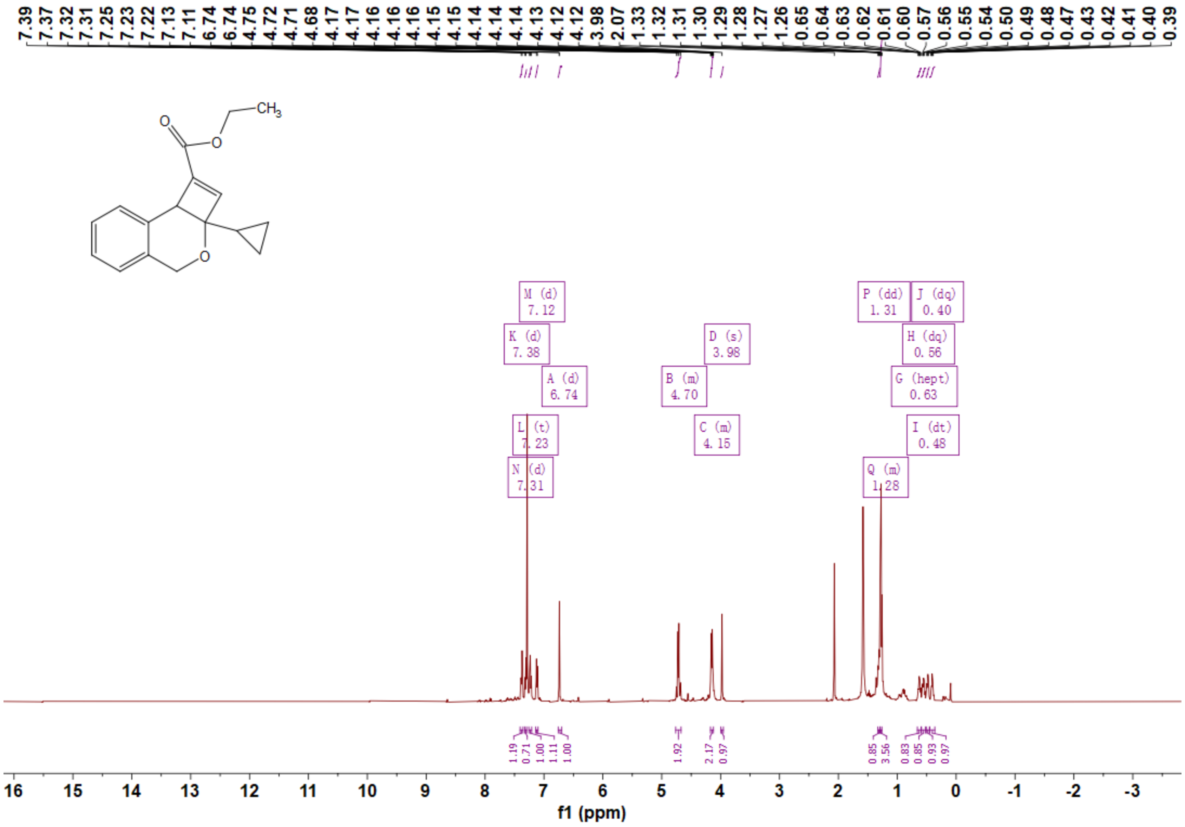

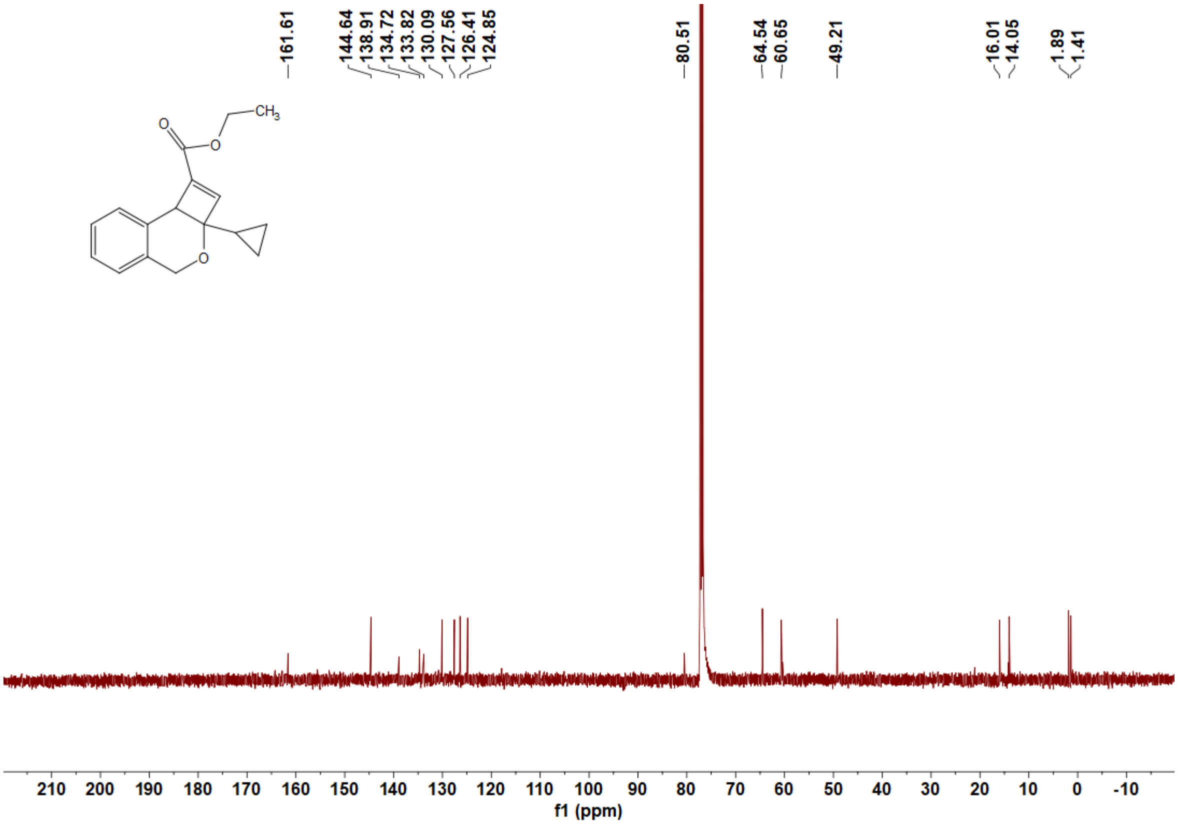

**Ethyl 2a-cyclohexyl-2a,8b-dihydro-4*H*-cyclobuta[*c*]isochromene-1-carboxylate (18b)**

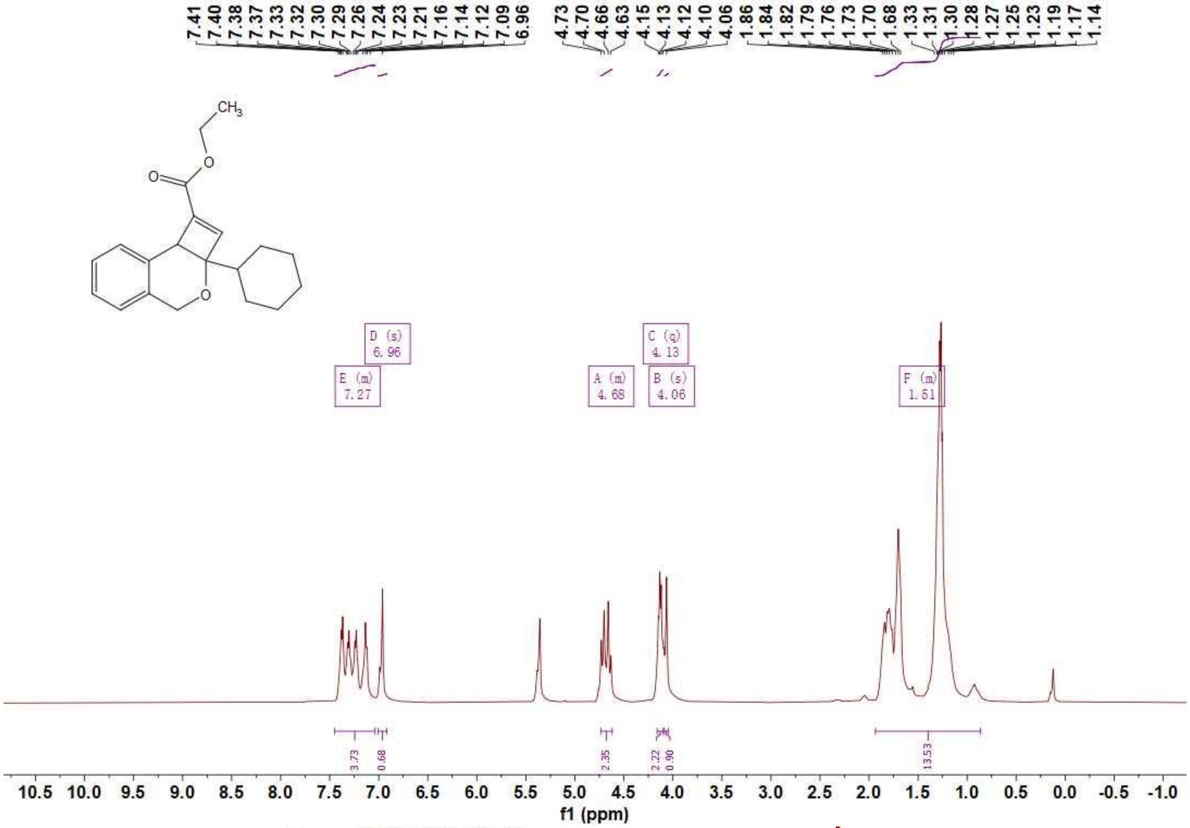


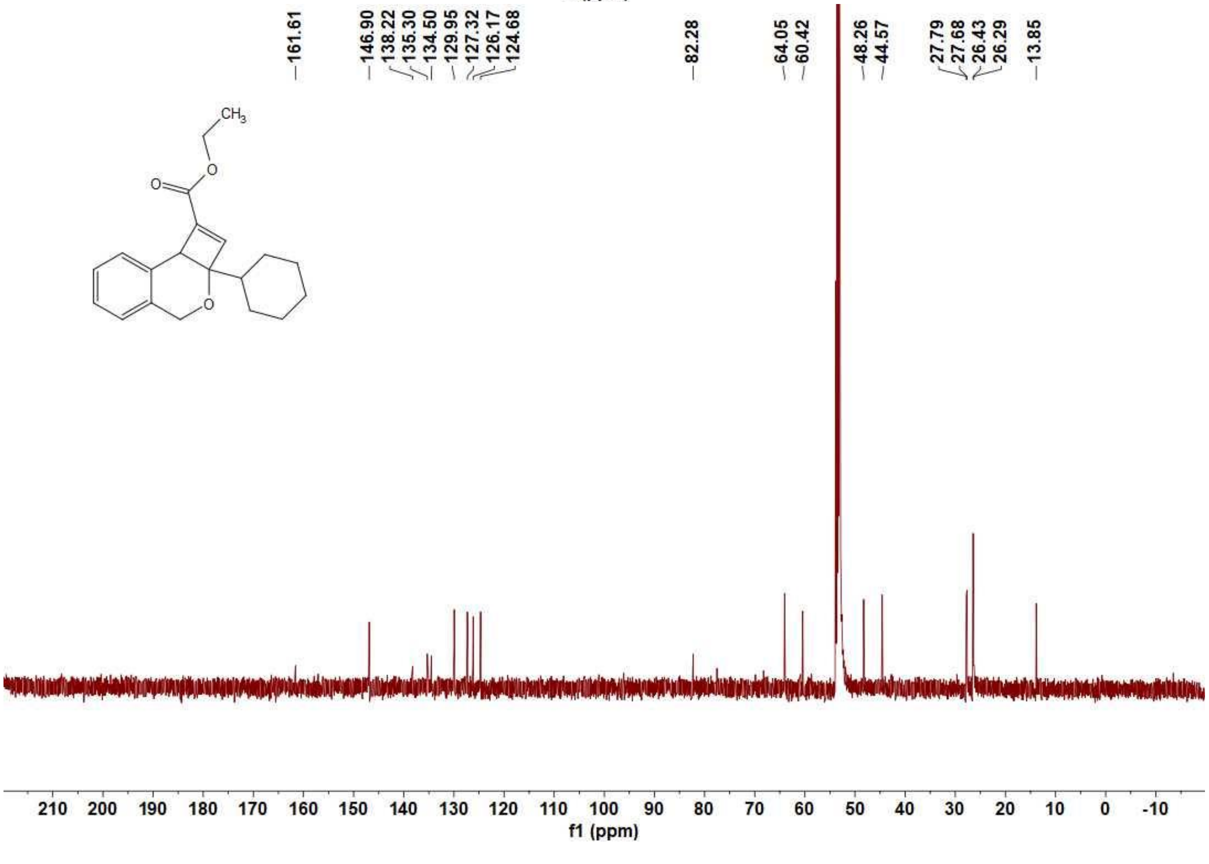

**Ethyl 2a-hexyl-2a,8b-dihydro-4*H*-cyclobuta[*c*]isochromene-1-carboxylate (19b)**

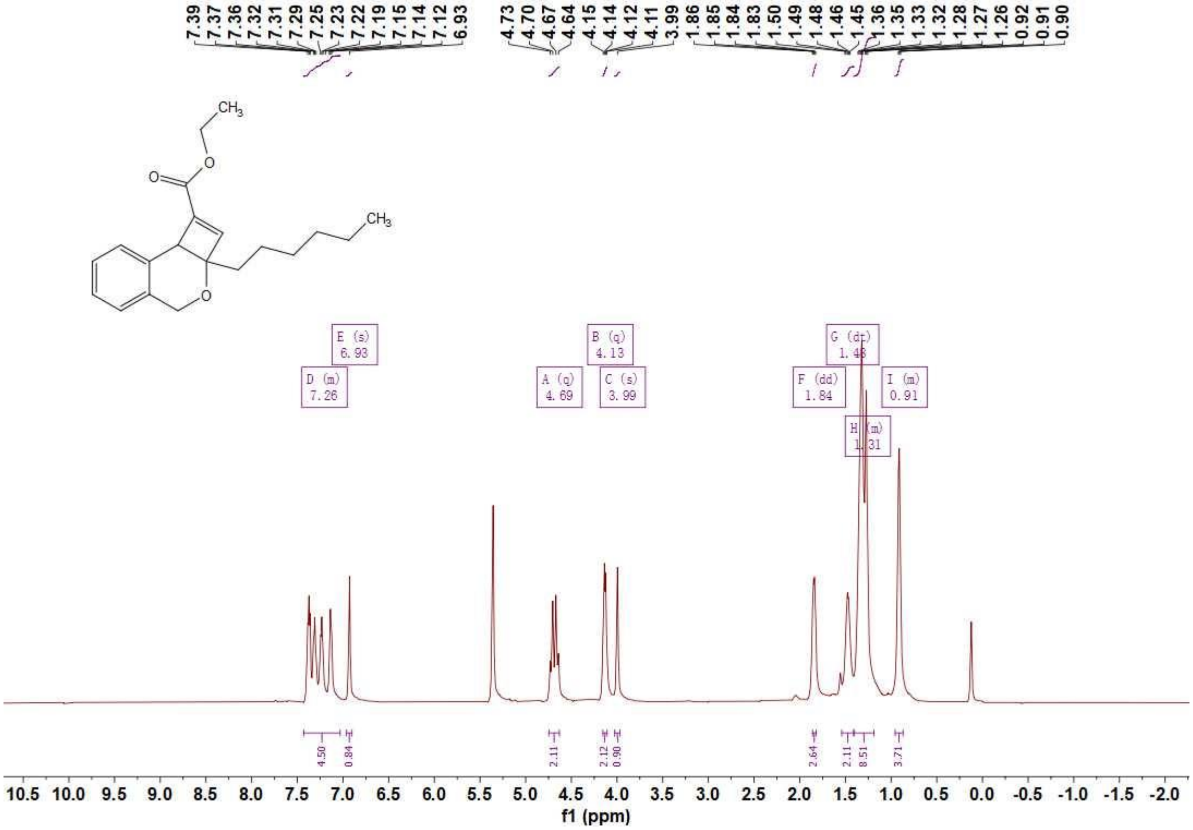

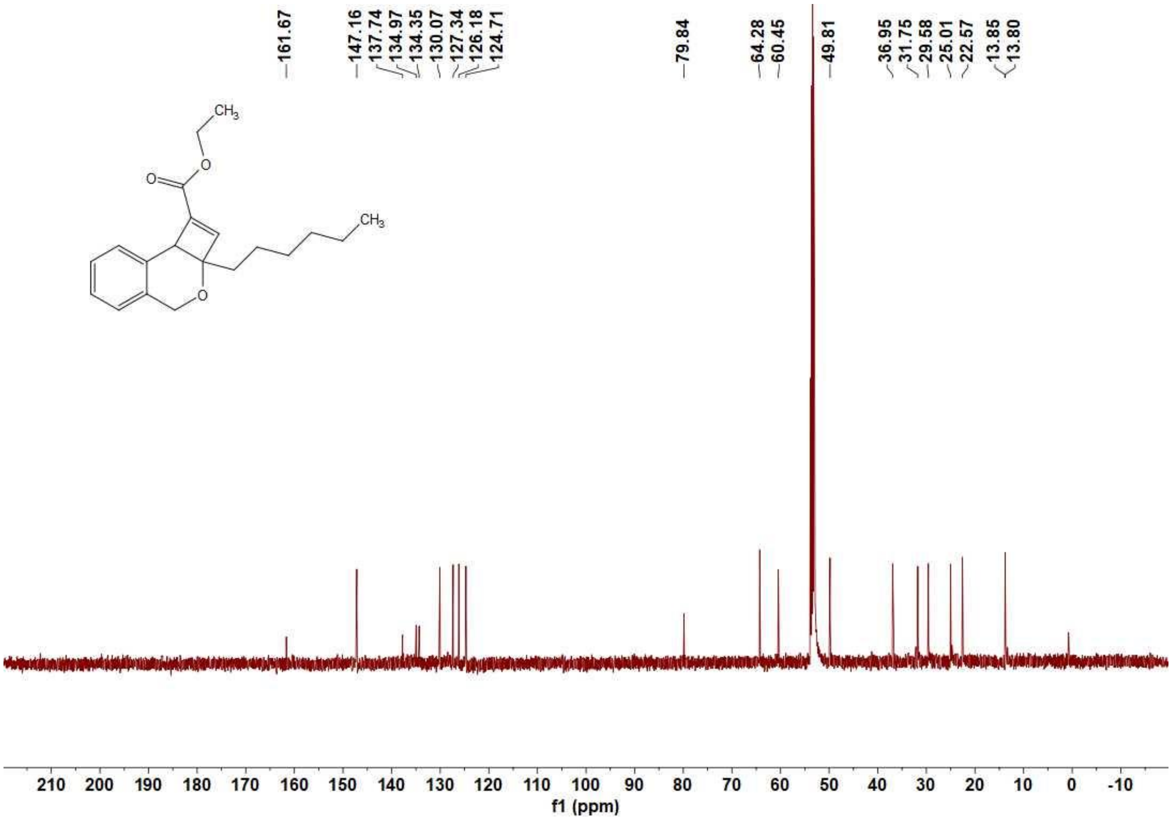

**Propyl-2a-phenyl-2a,8b-dihydro-4*H*-cyclobuta[*c*]isochromene-1-carboxylate (20b)**

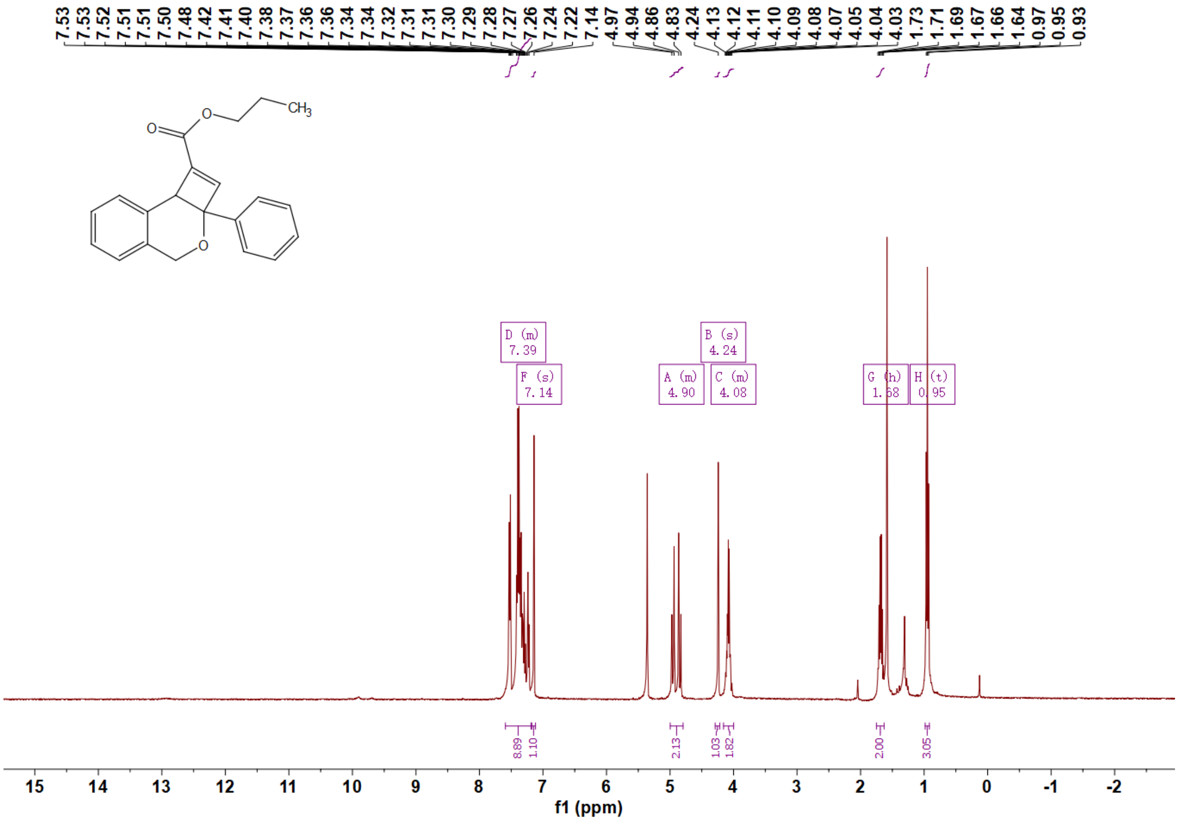

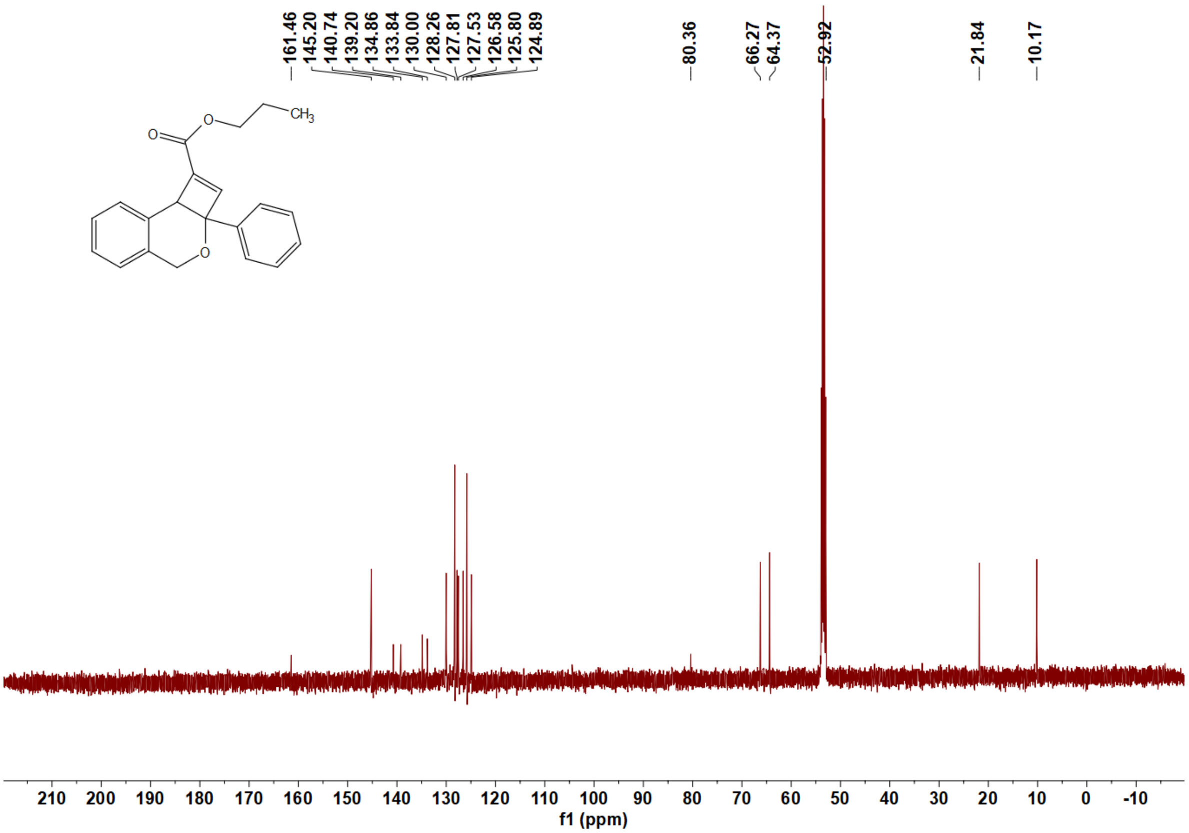

**Tert-butyl 2a-phenyl-2a,8b-dihydro-4*H*-cyclobuta[*c*]isochromene-1-carboxylate (21b)**

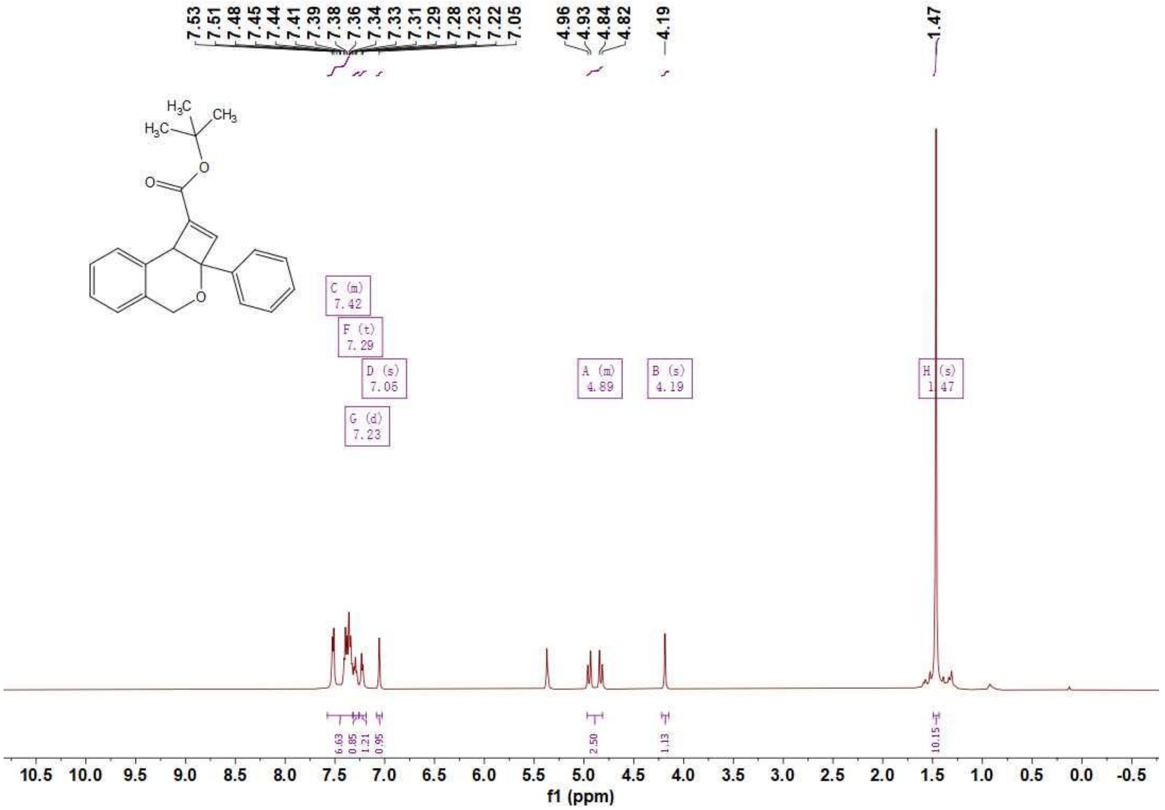


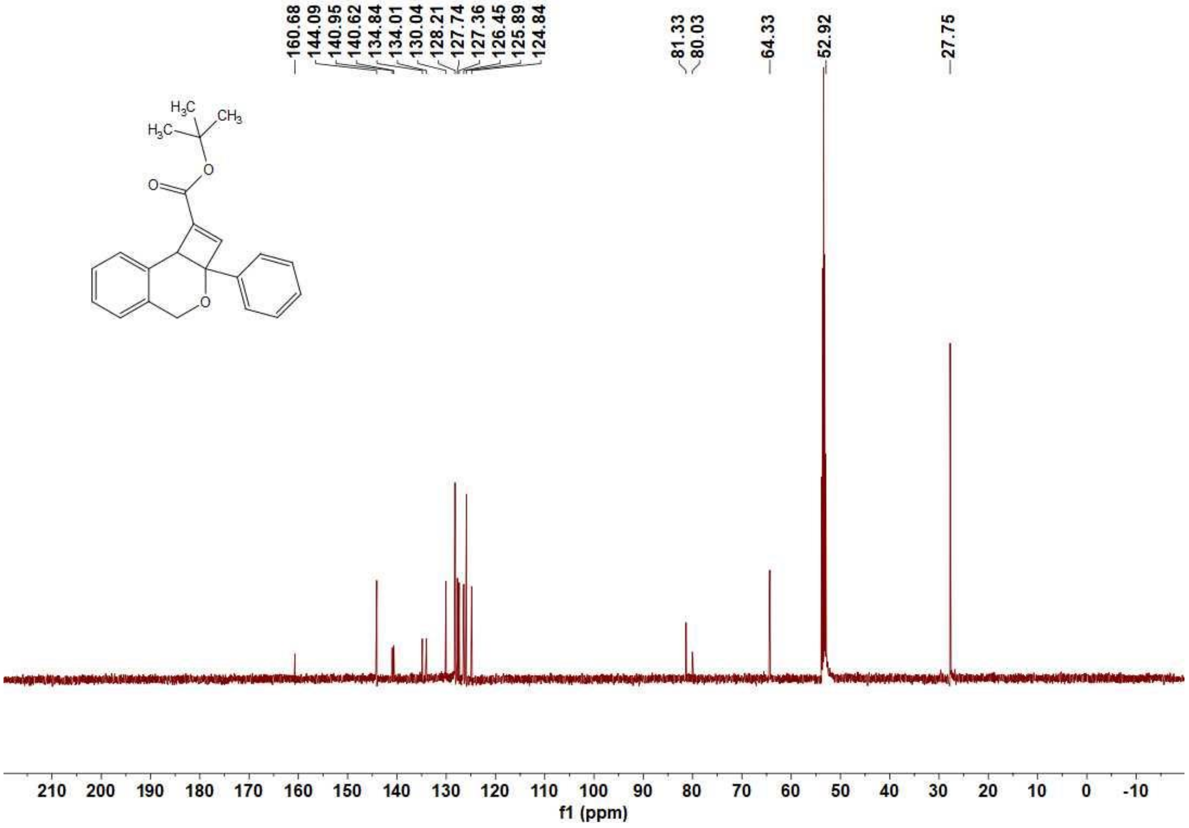

**Ethyl 6-methoxy-2a-methyl-2a,8b-dihydro-4*H*-cyclobuta[*c*]isochromene-1-carboxylate (22b)**

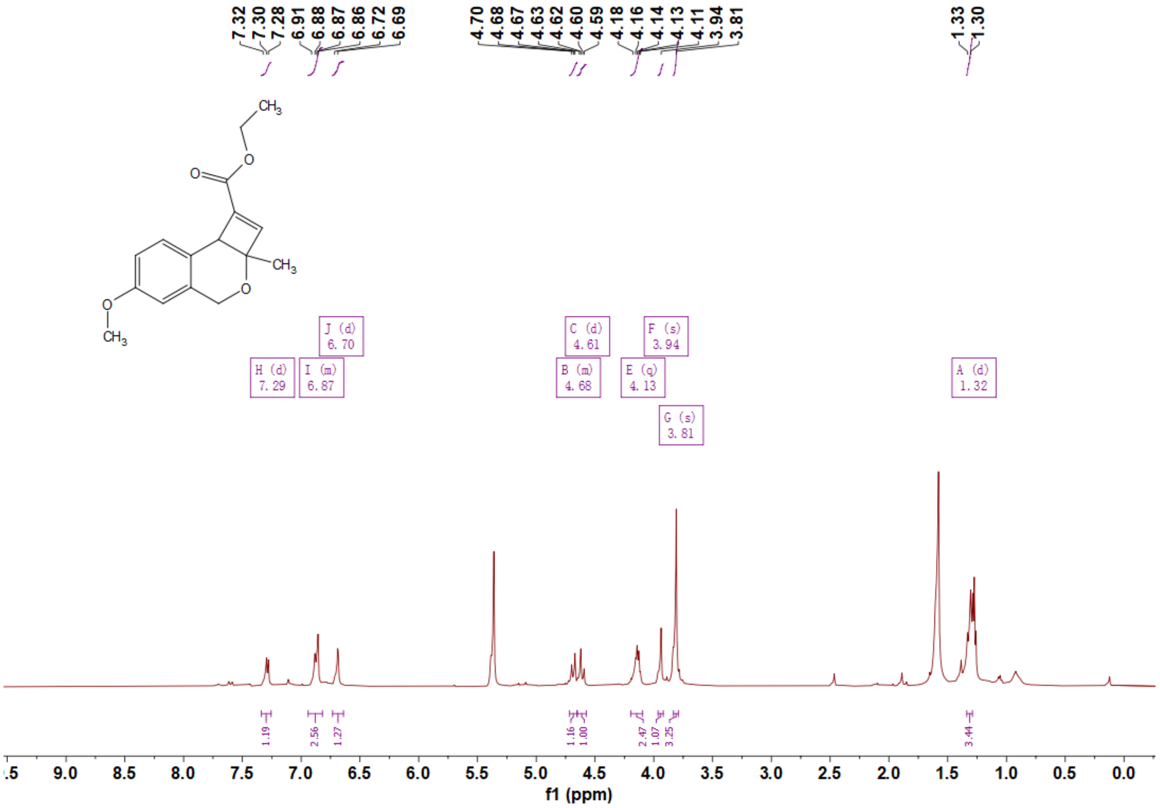

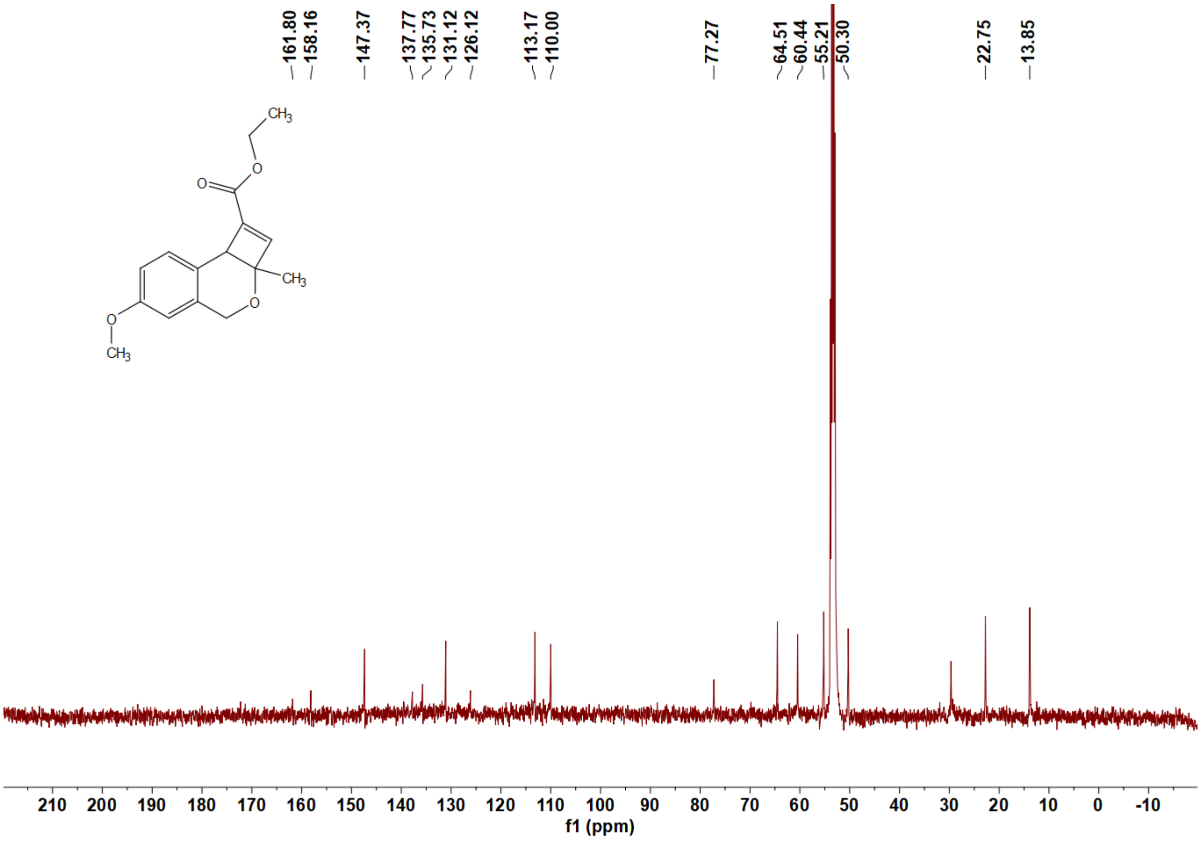

**Ethyl 2a-methyl-6-(trifluoromethyl)-2a,8b-dihydro-4*H*-cyclobuta[*c*]isochromene-1-carboxylate (23b)**

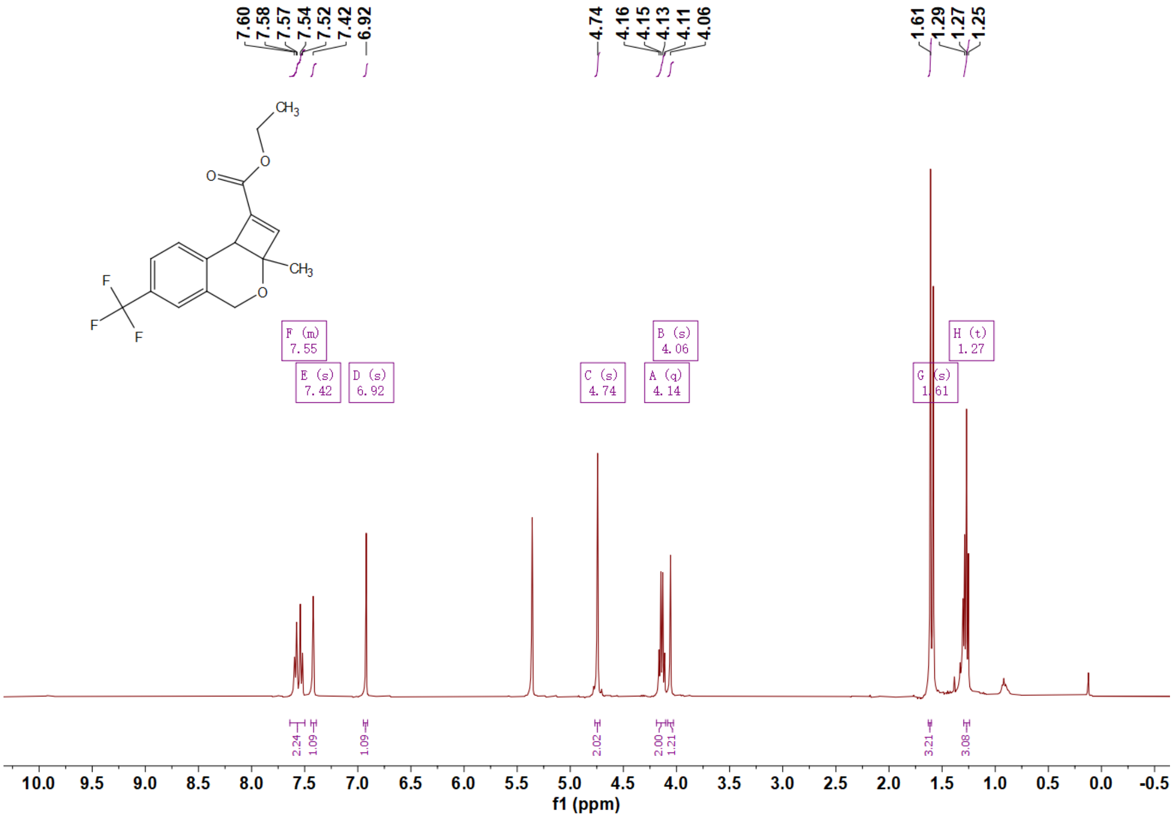

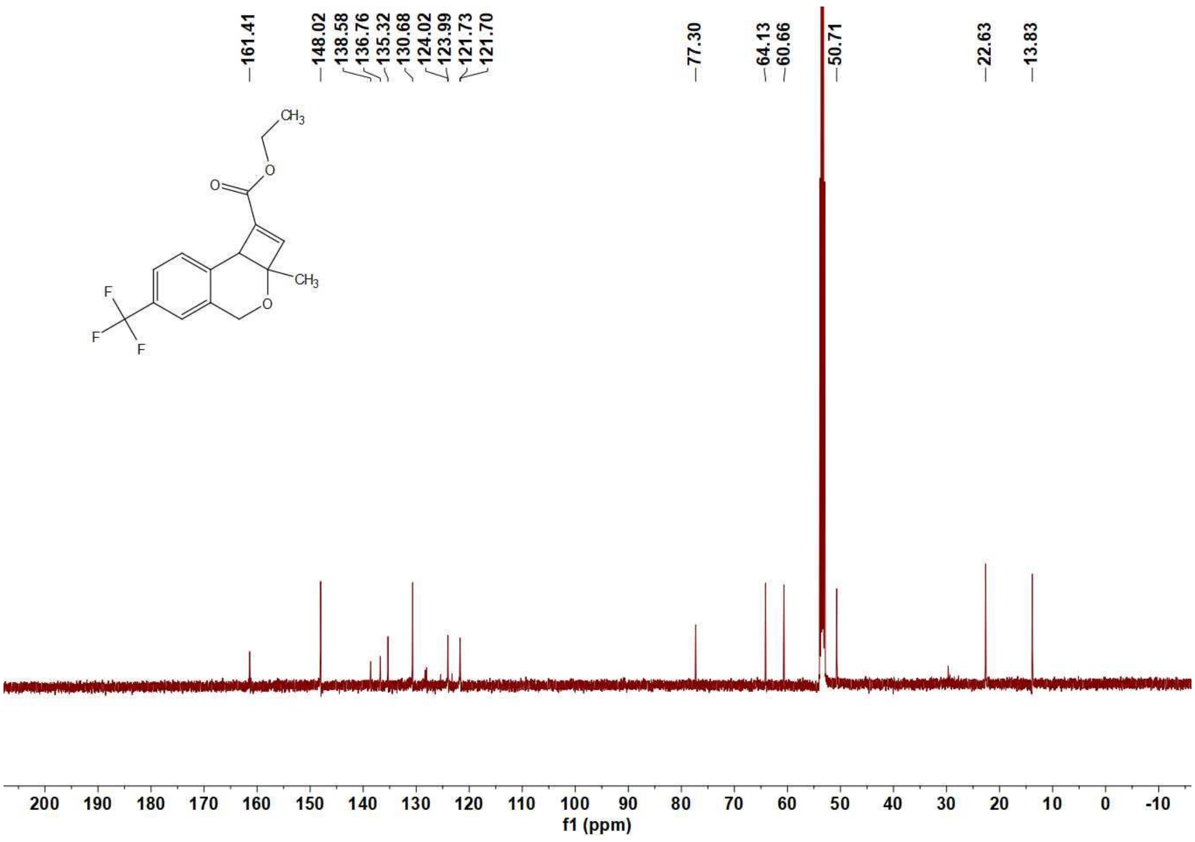

**Ethyl 2a-((3r,5r,7r)-adamantan-1-yl)-2a,8b-dihydro-4*H*-cyclobuta[*c*]isochromene-1-carboxylate (24b)**

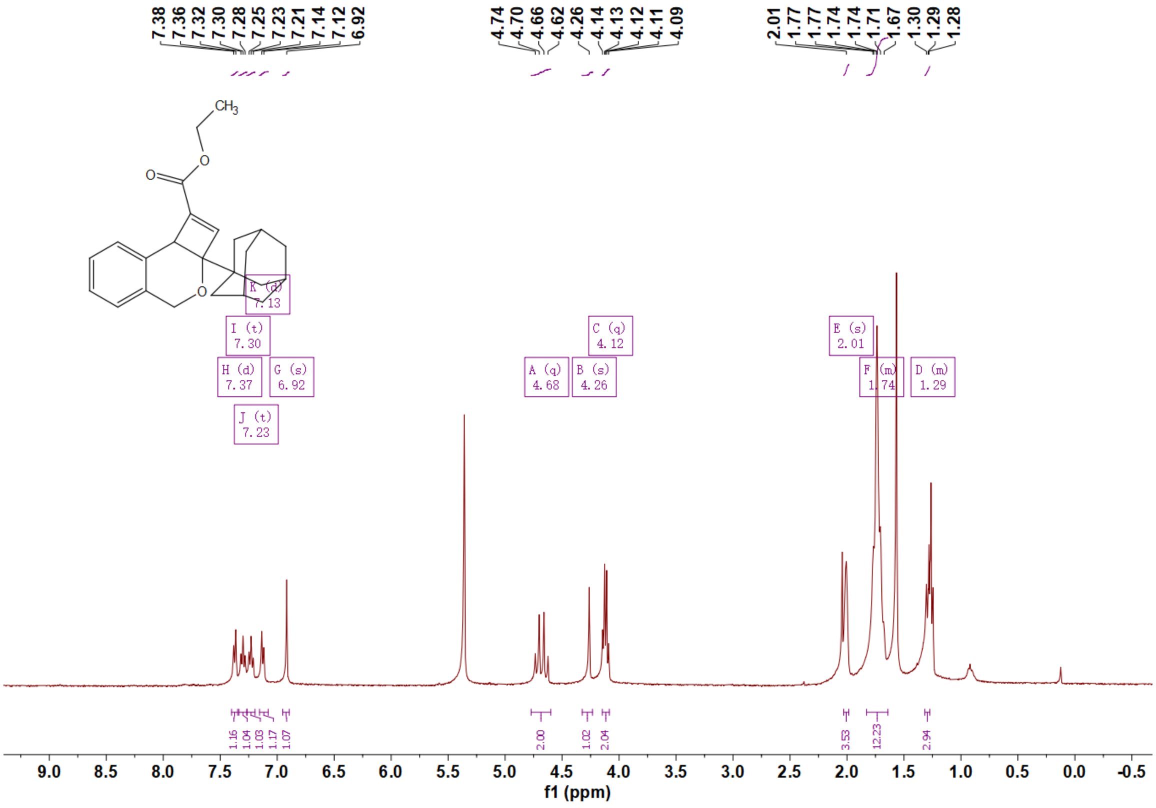


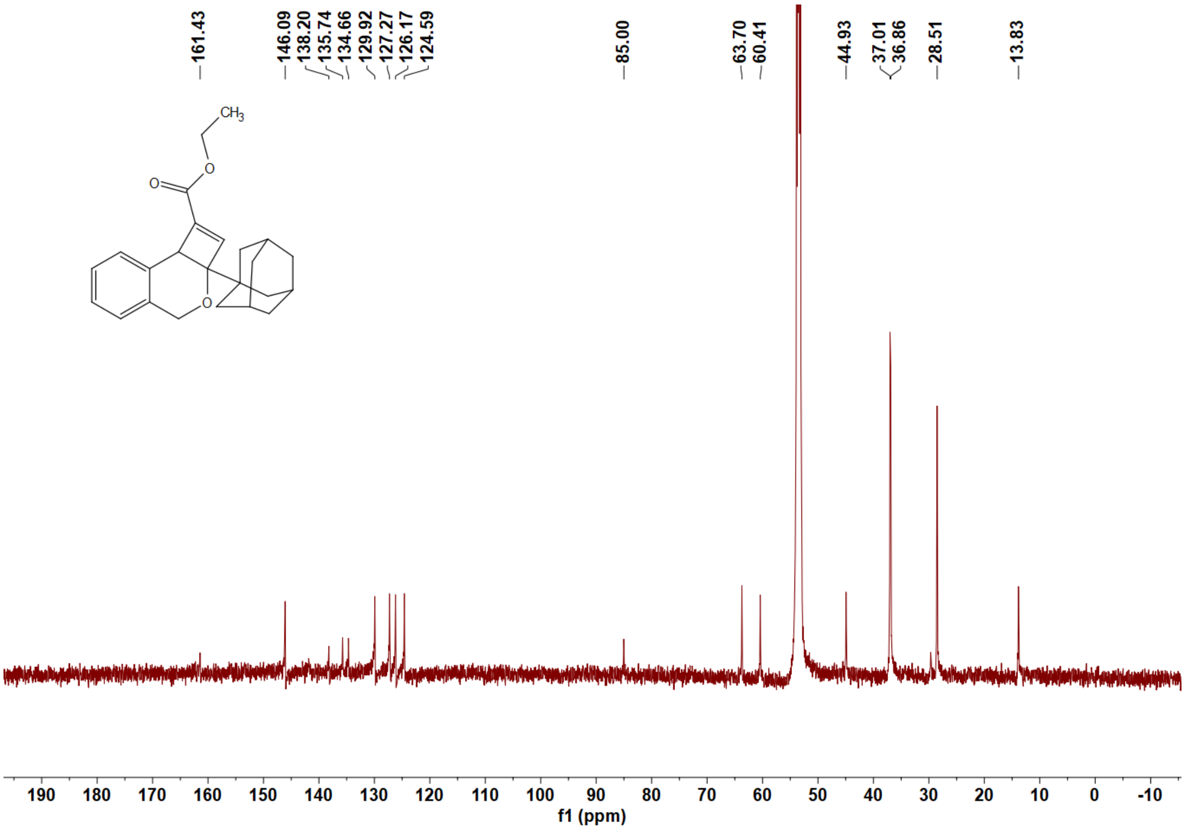

**2-((1S,5R)-6,6-dimethylbicyclo[3.1.1]hept-2-en-2-yl)ethyl 2a-methyl-2a,8b-dihydro-4*H*-cyclobuta[*c*]iso-chromene-1-carboxylate (25b)**

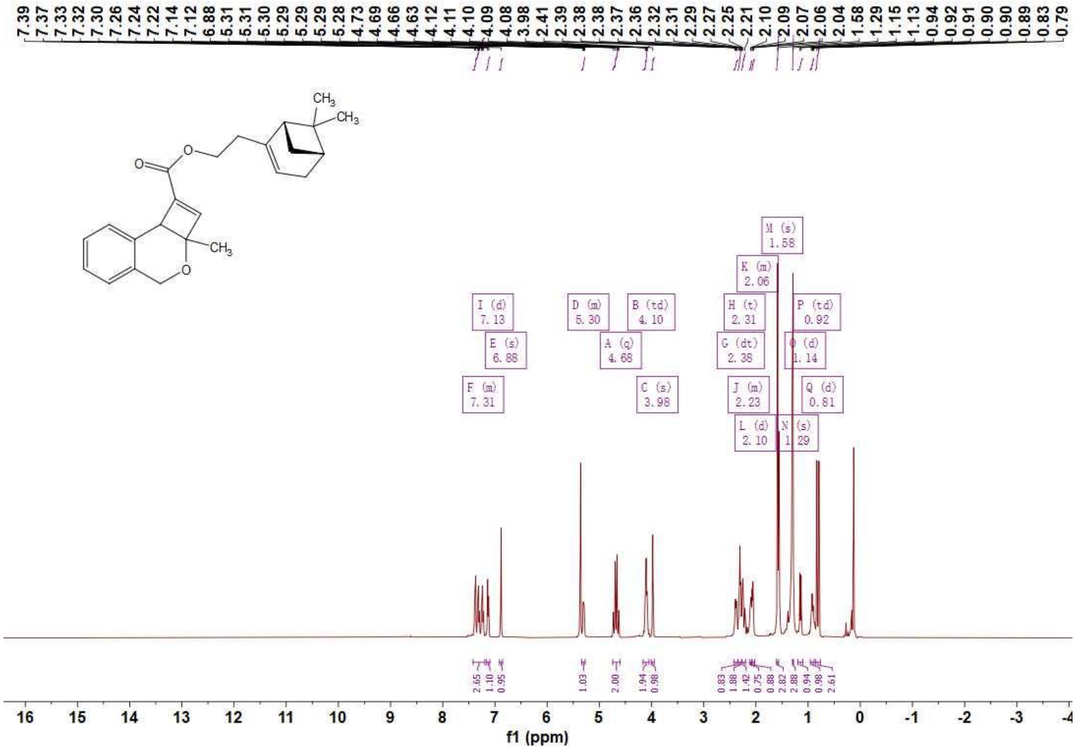


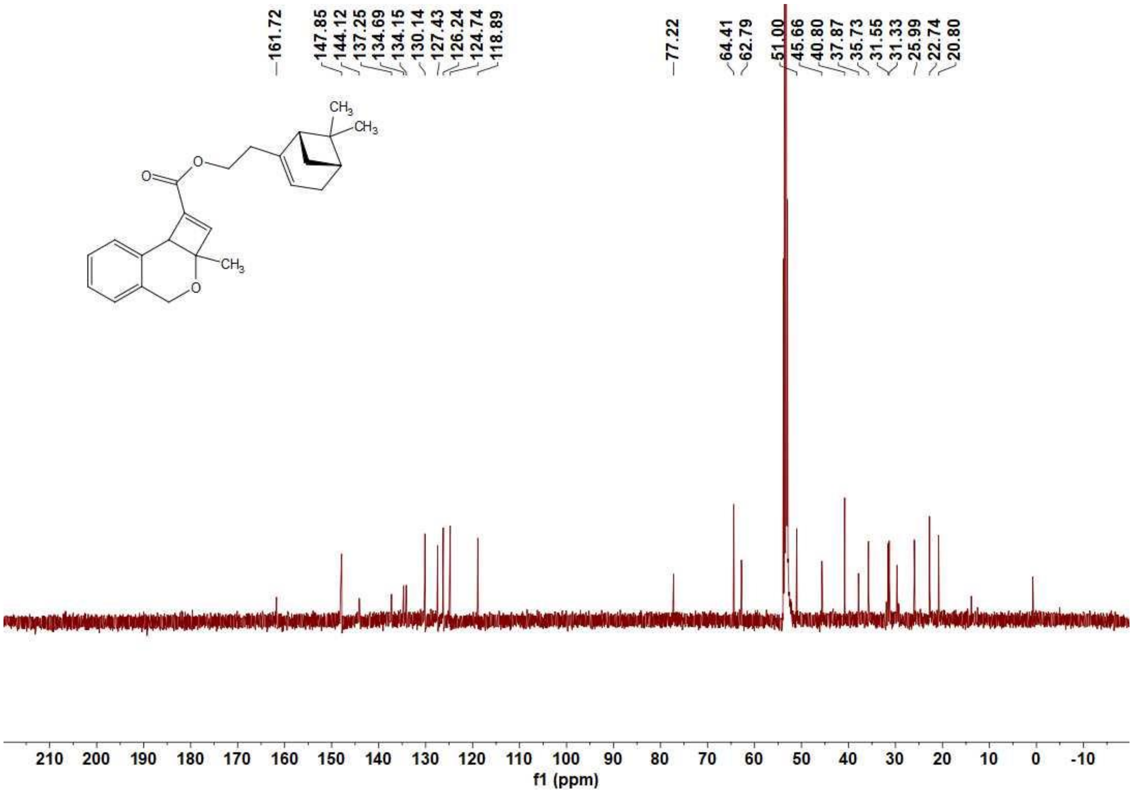

**((R)-4-(prop-1-en-2-yl)cyclohex-1-en-1-yl)methyl-2a-methyl-2a,8b-dihydro-4*H*-cyclobuta[*c*]isochromene-1-carboxylate (26b)**

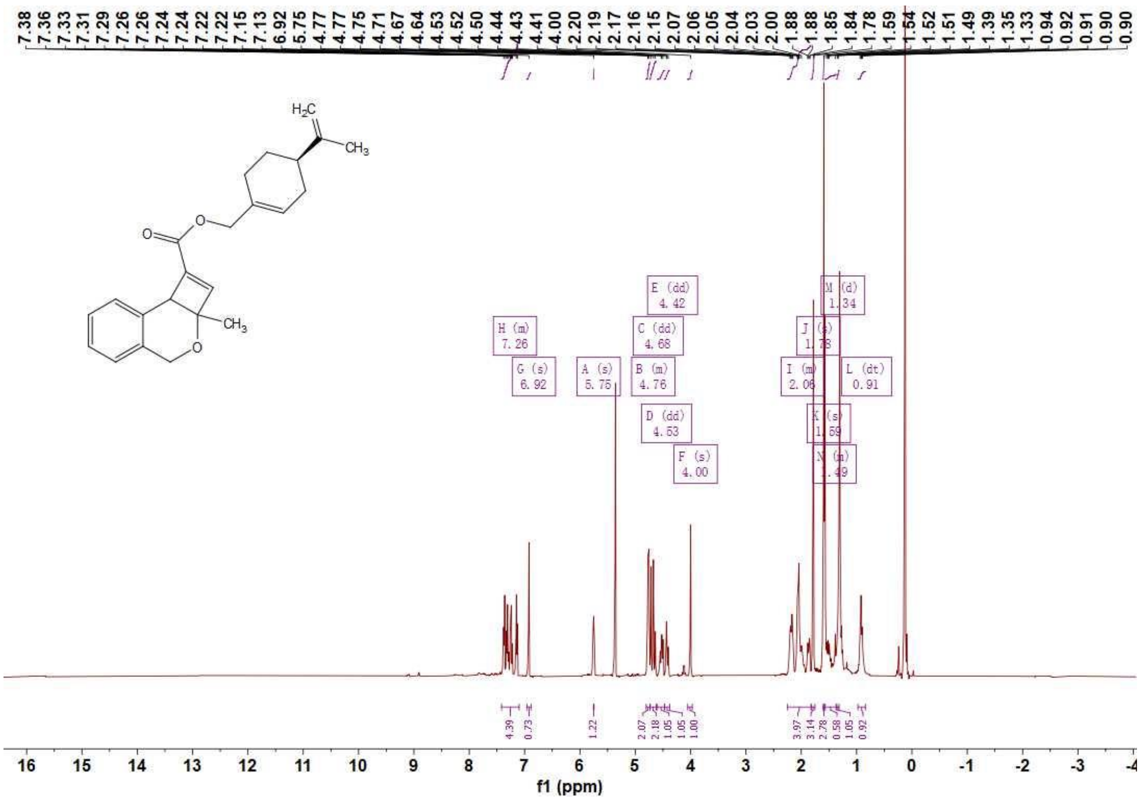


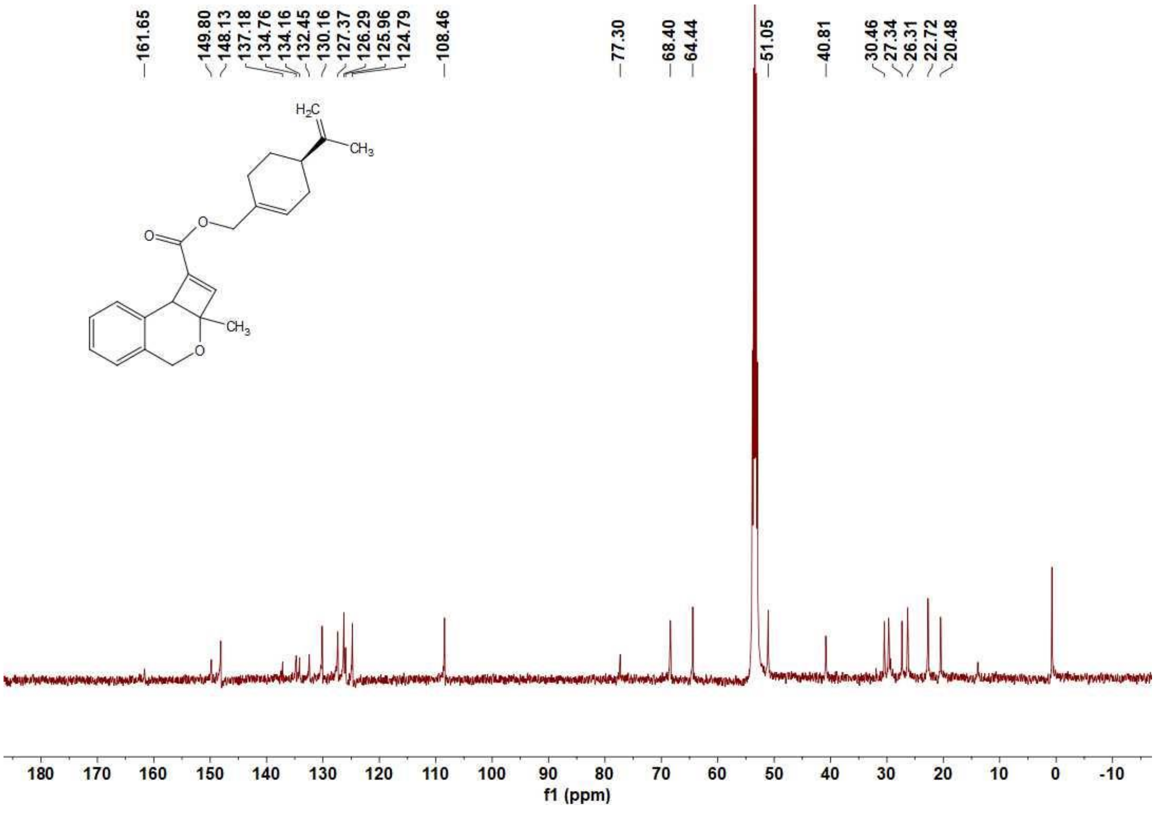

**(1R,4R)-1,7,7-trimethylbicyclo[2.2.1]heptan-2-yl-2a-methyl-2a,8b-dihydro-4*H*-cyclobuta[*c*]iso-chromene-1-carboxylate (27b)**

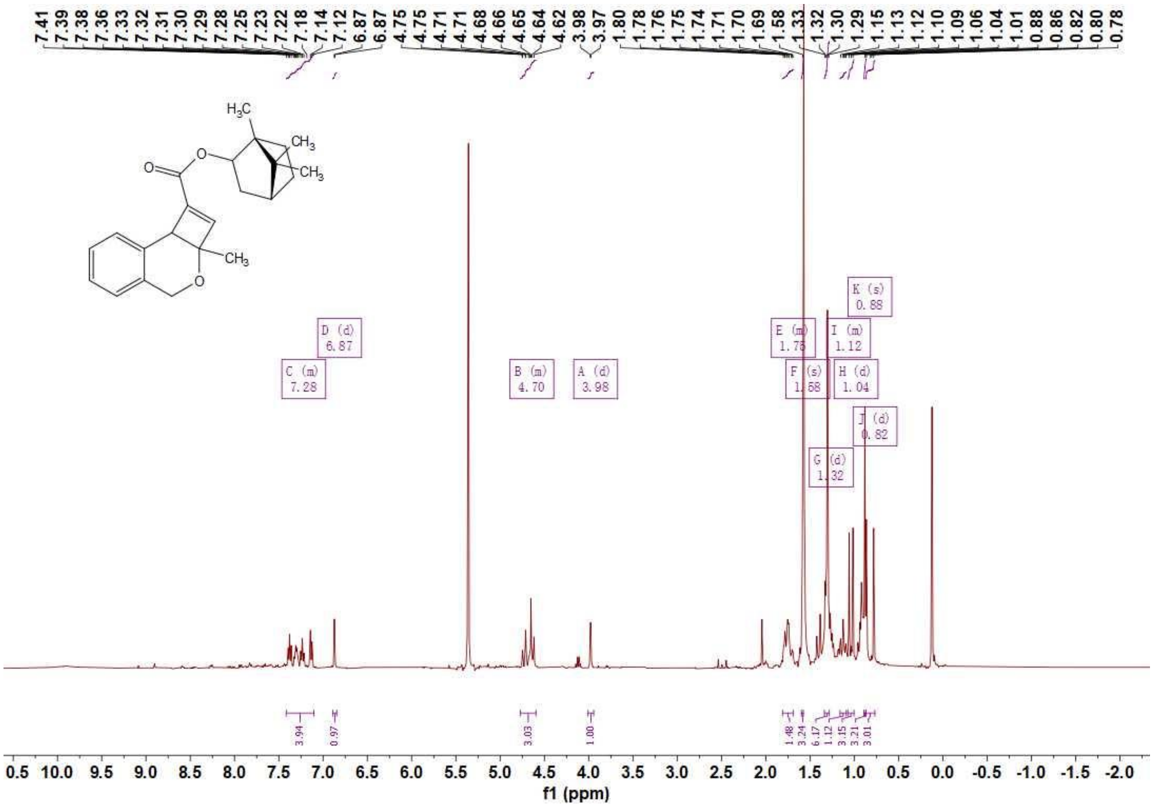


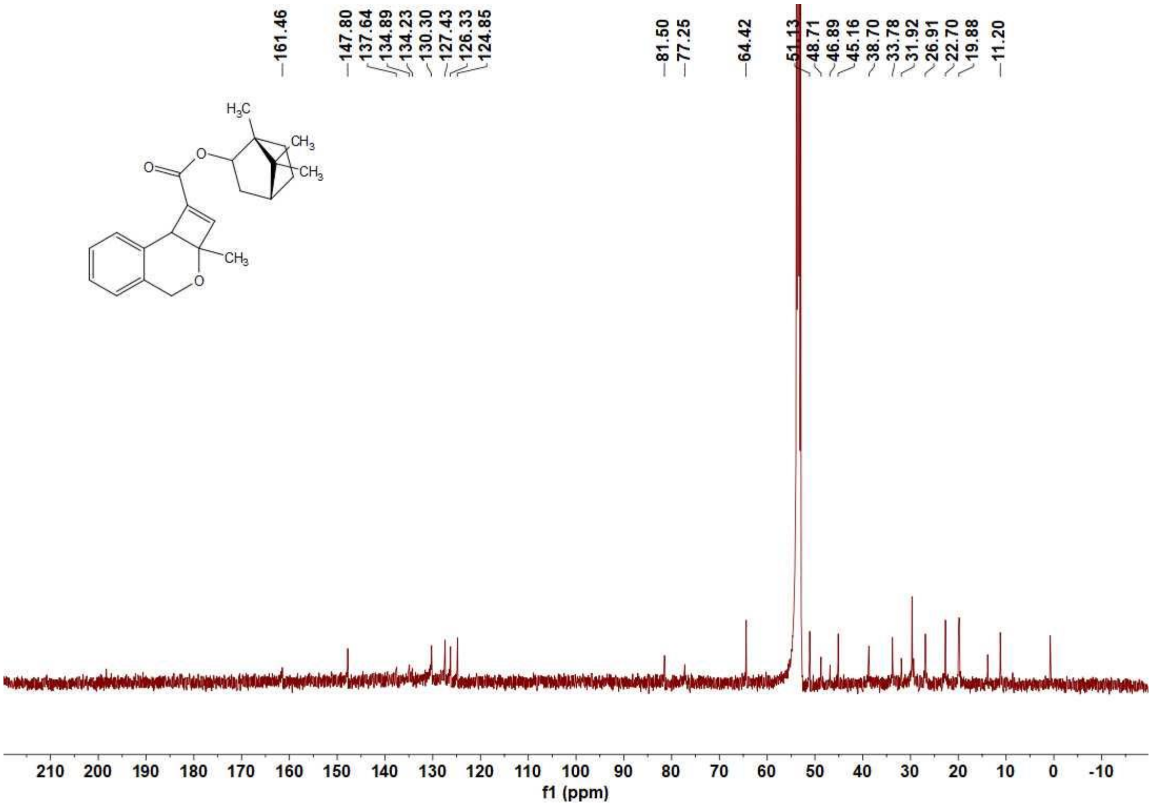

## 9.3. NMR spectra of dihydronaphalenes

### Ethyl 3-benzoyl-3,4-dihydronaphthalene-2-carboxylate (1d)

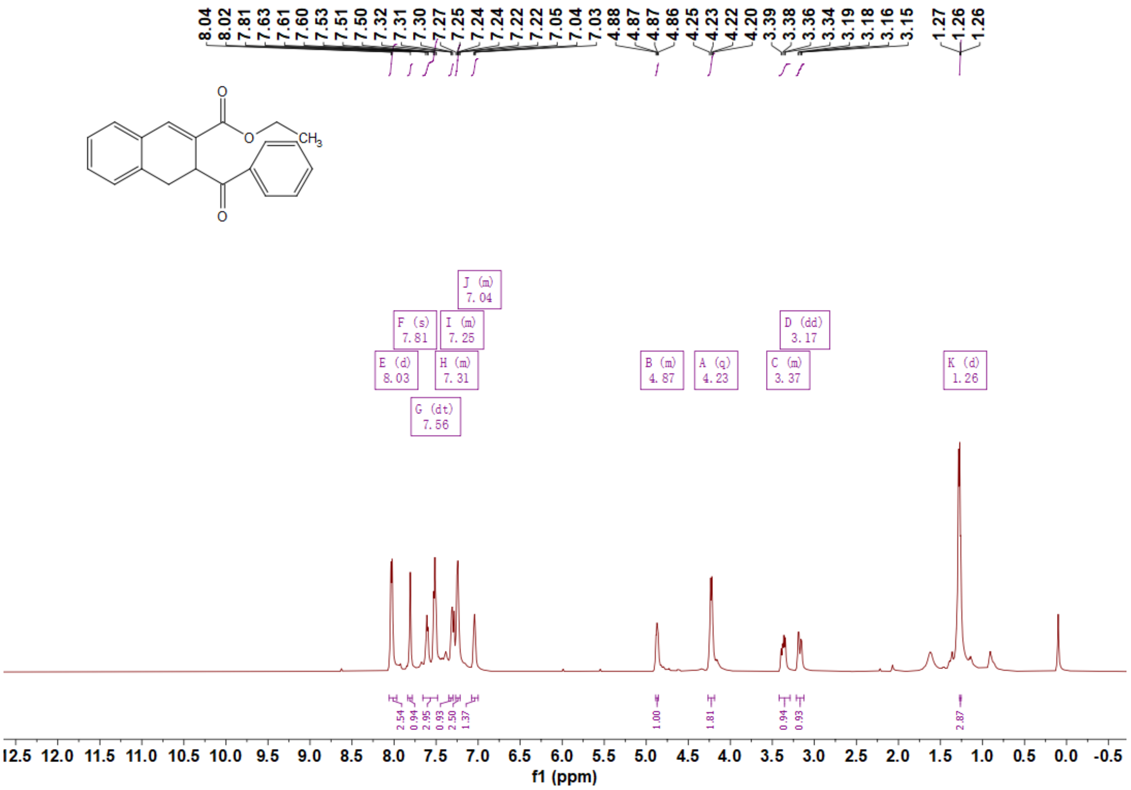

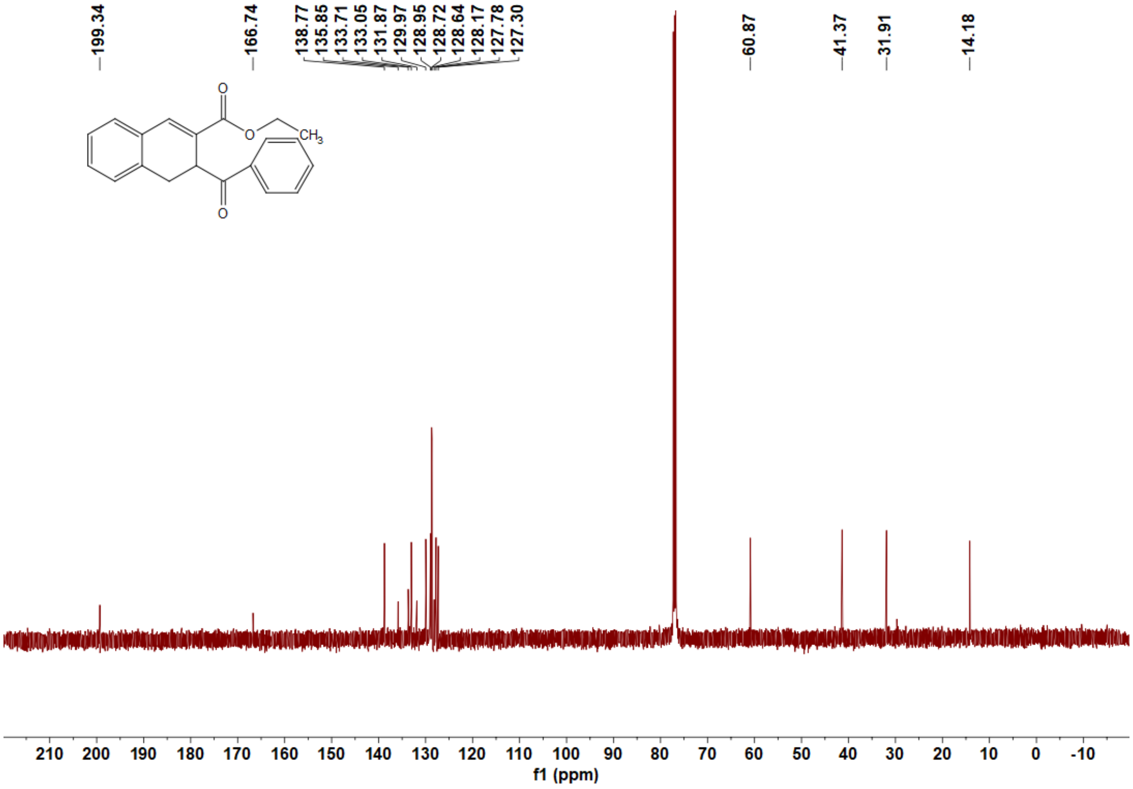

**Ethyl 3-(2,3-dihydrobenzo[*b*][1,4]dioxine-6-carbonyl)-3,4-dihydronaphthalene-2-carboxylate (4d)**

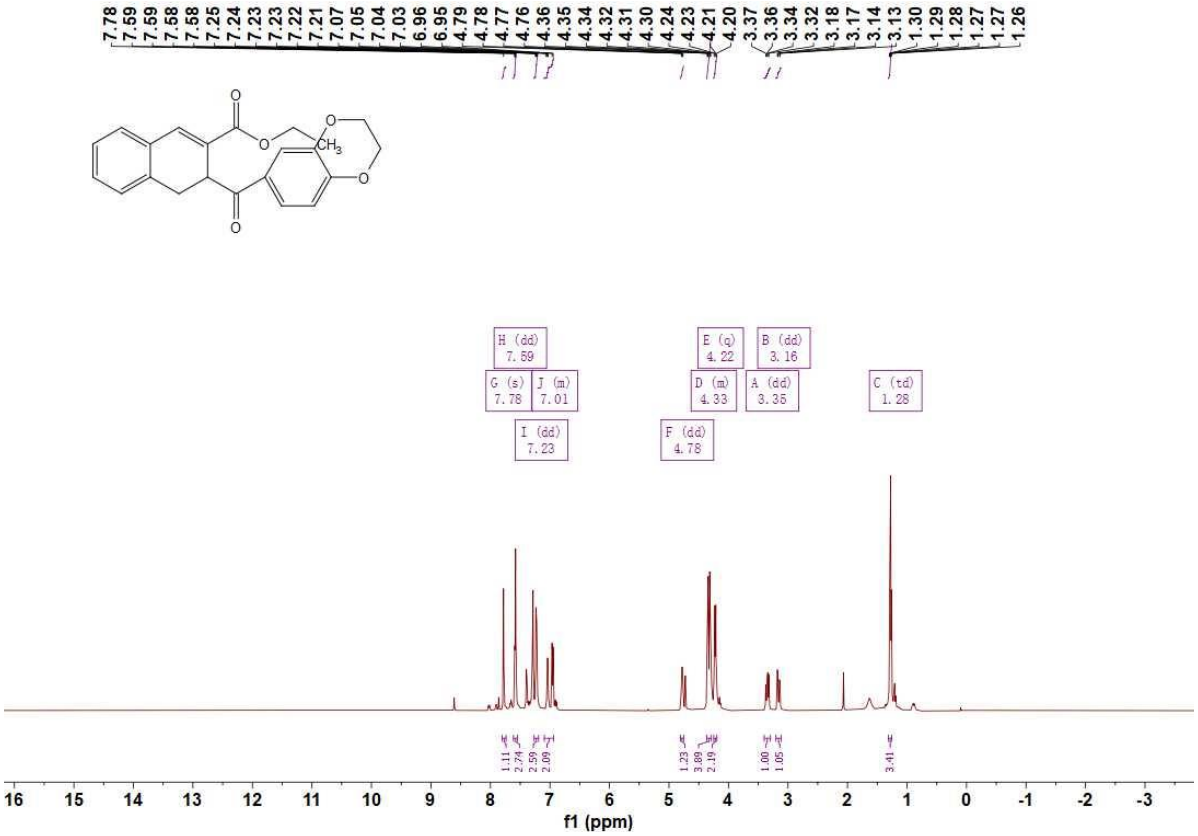

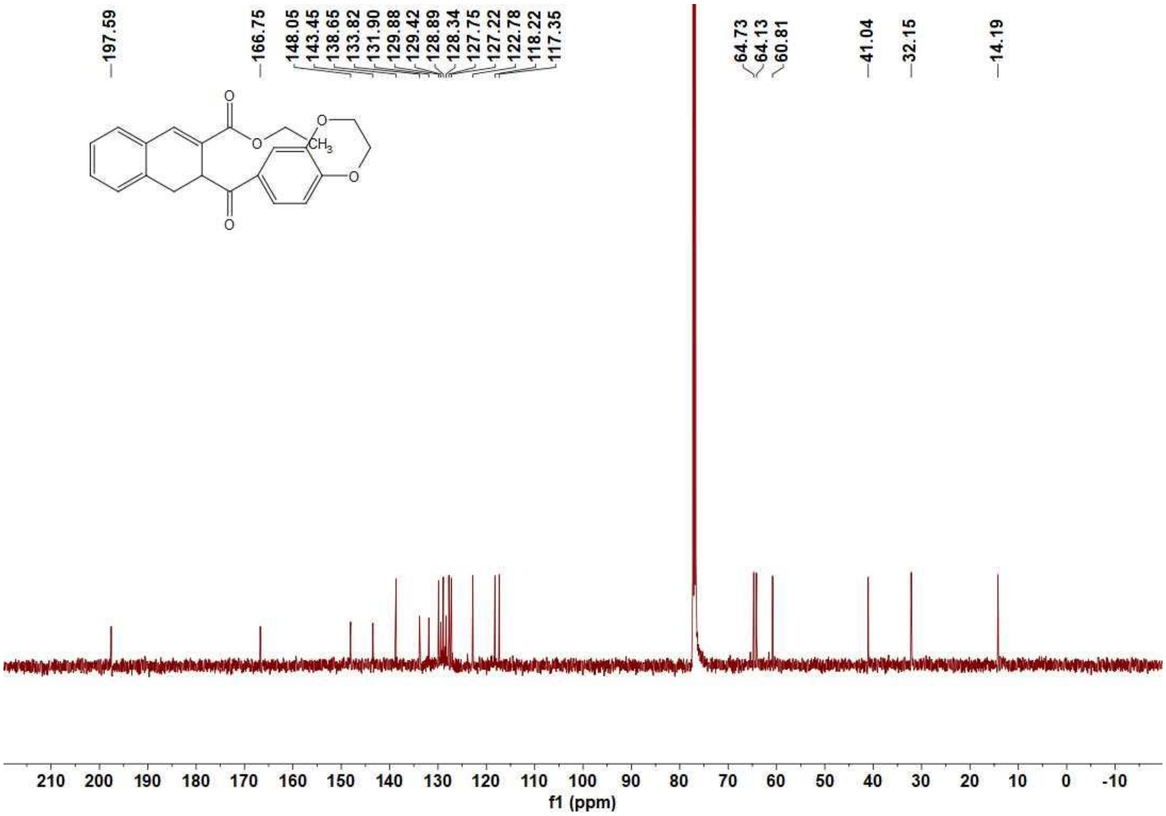

**Ethyl 3-(1-naphthoyl)-3,4-dihydronaphthalene-2-carboxylate (5d)**

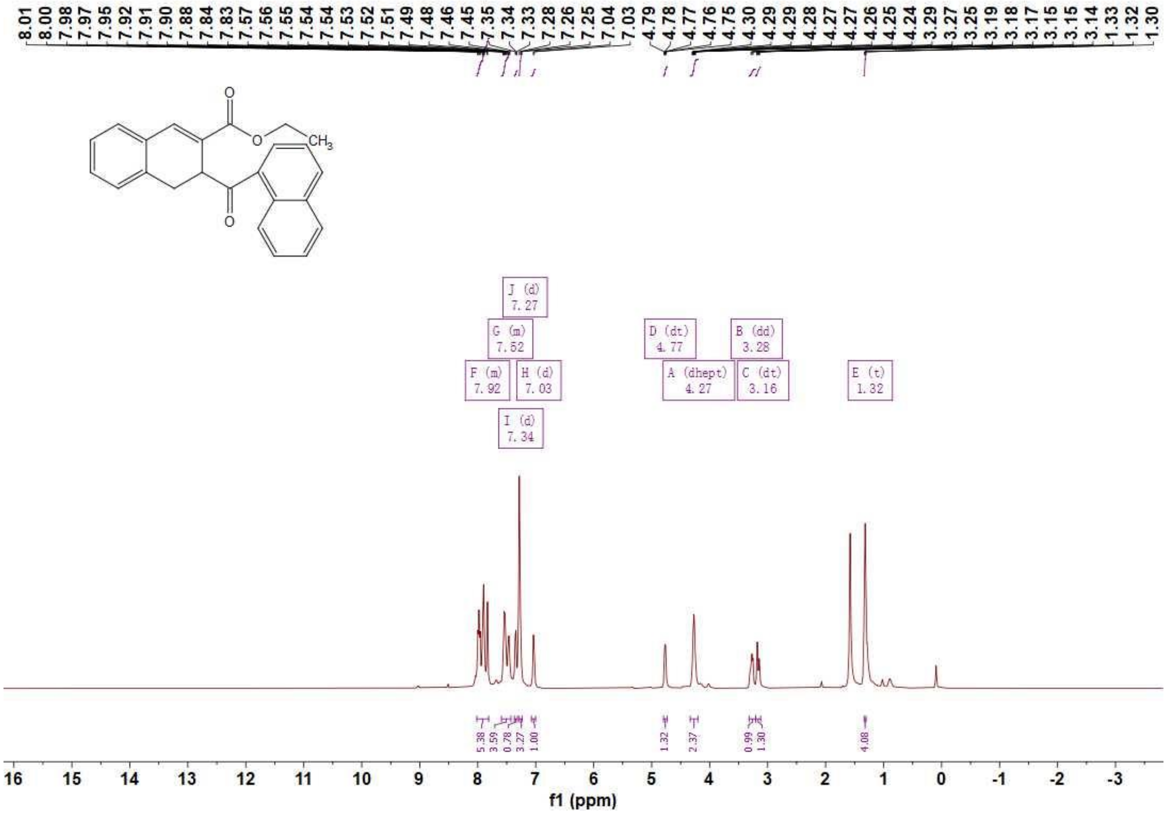


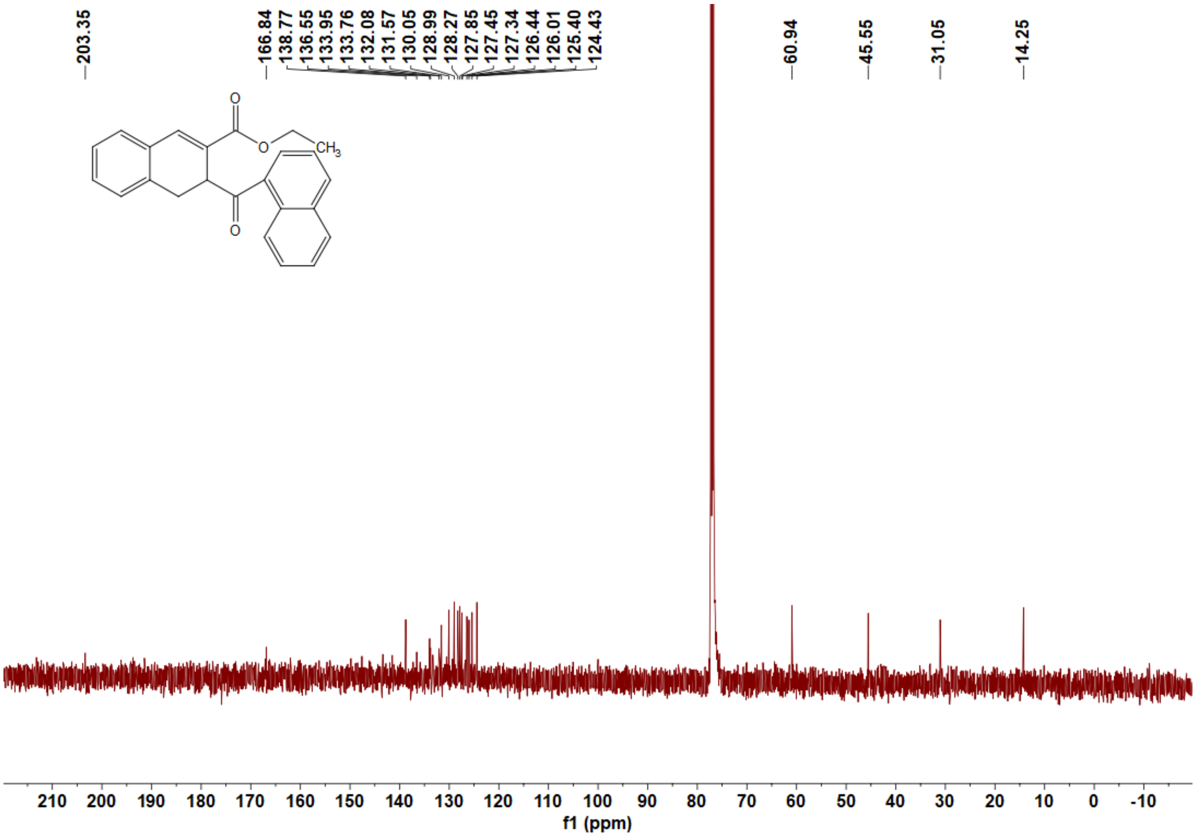

**Ethyl 3-acetyl-3,4-dihydronaphthalene-2-carboxylate (13d)**

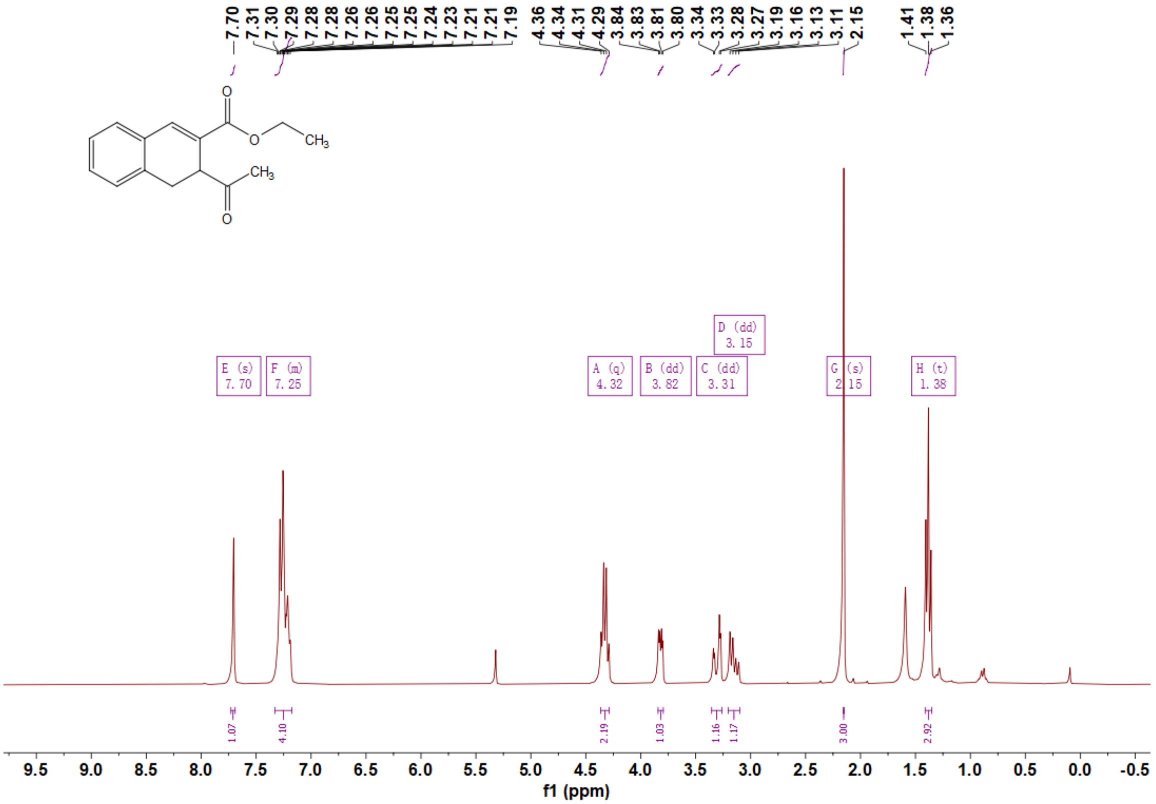


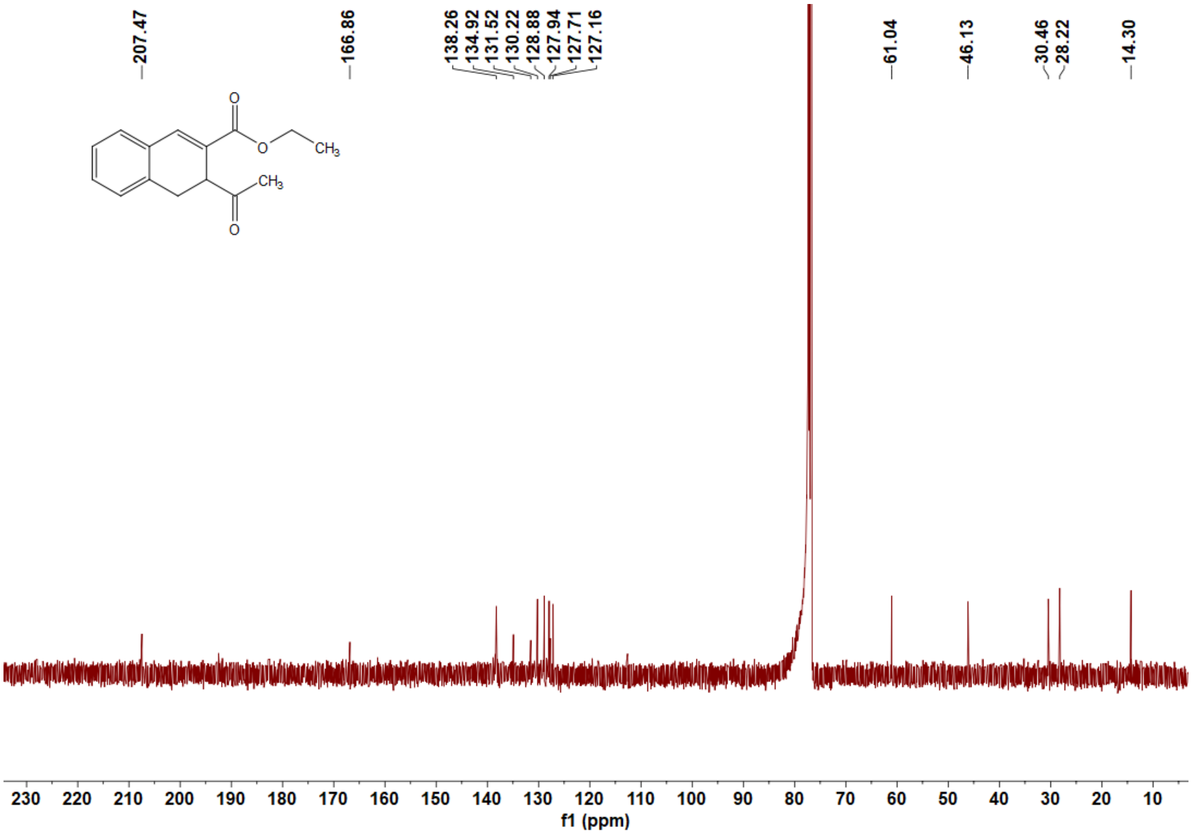

**Ethyl 3-isobutyryl-3,4-dihydronaphthalene-2-carboxylate (15d)**

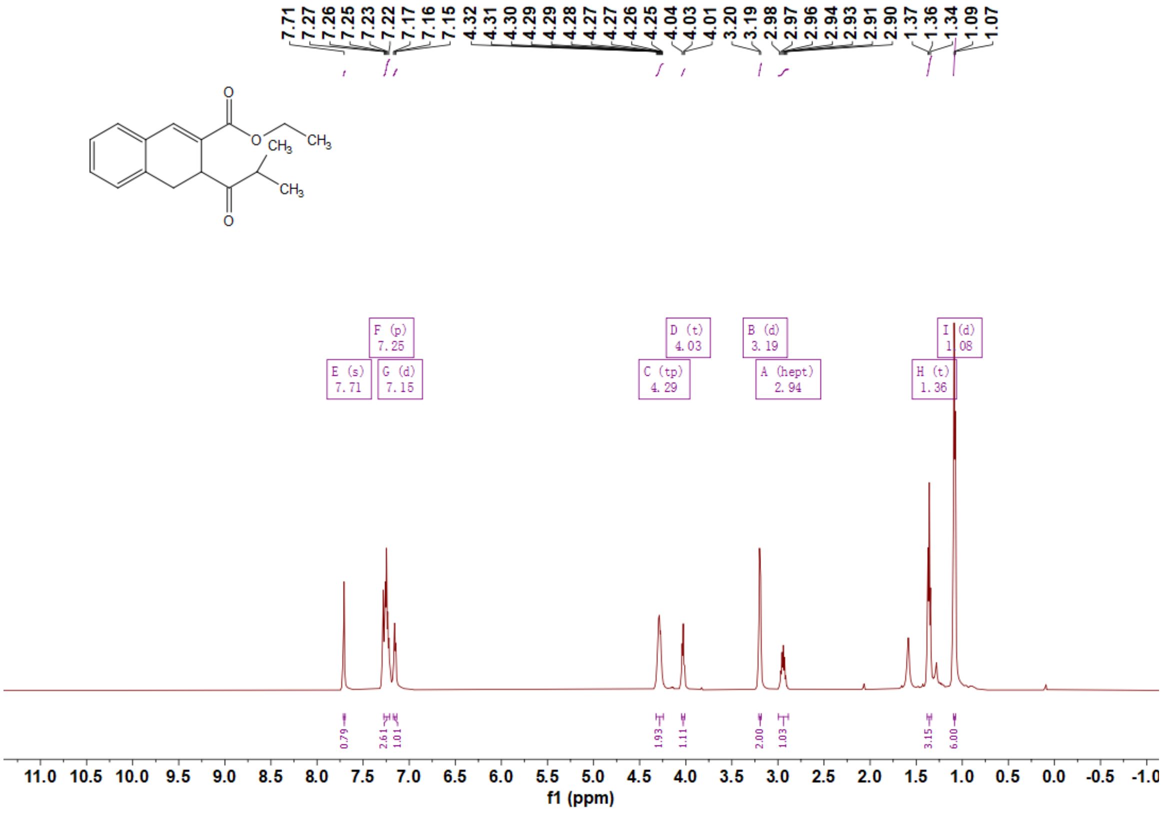


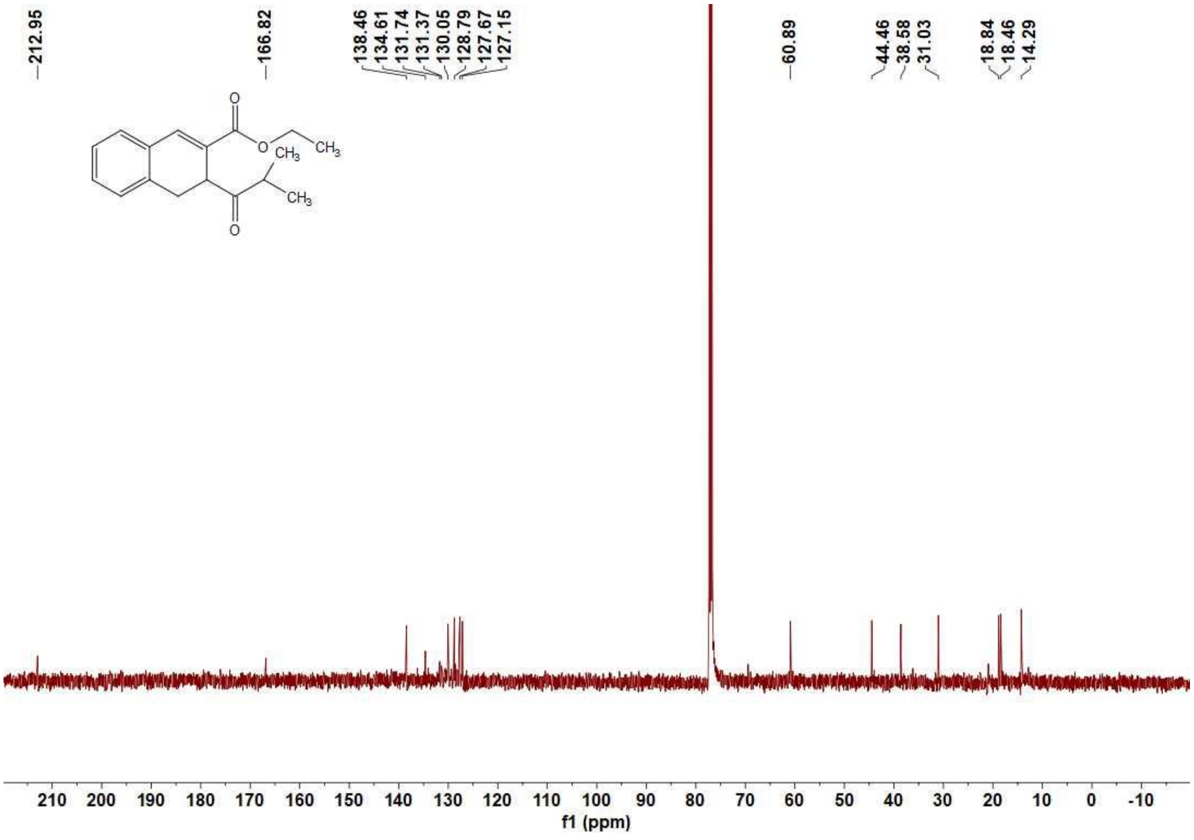

**Ethyl 3-pivaloyl-3,4-dihydronaphthalene-2-carboxylate (16d)**

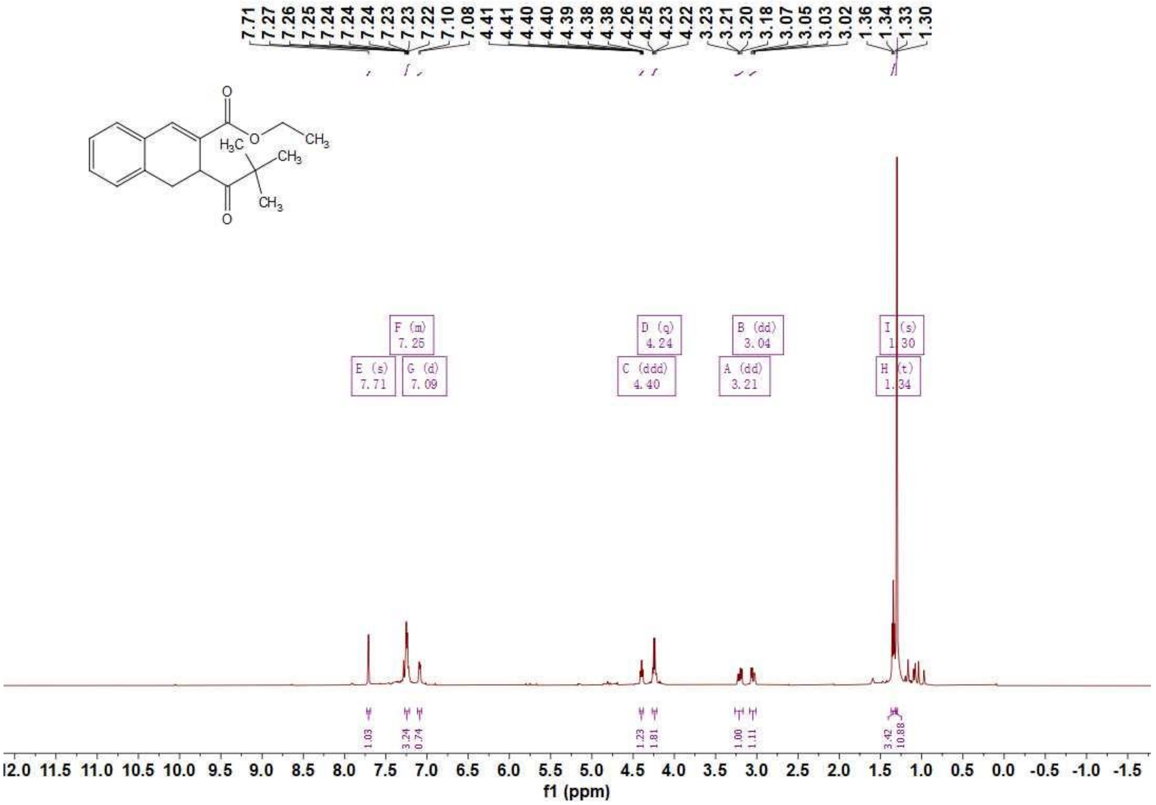


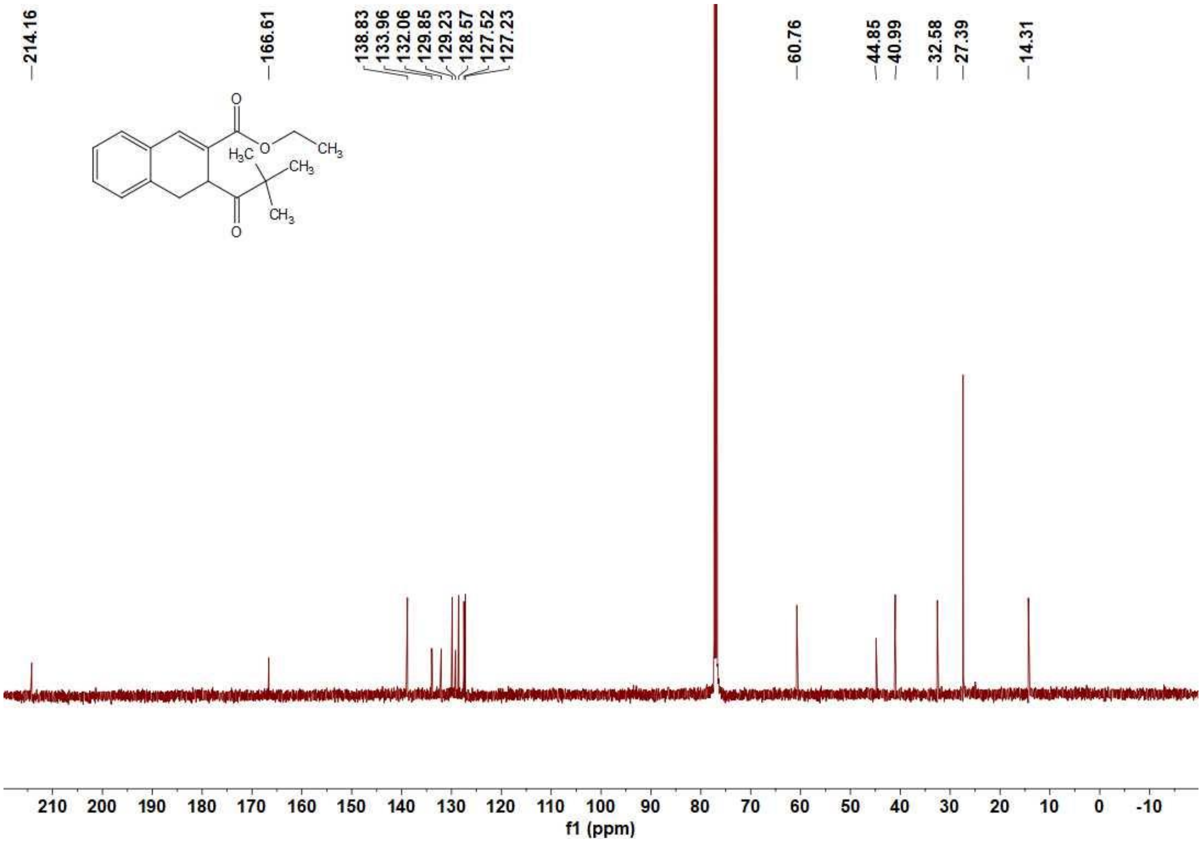

**Ethyl 3-(cyclopropanecarbonyl)-3,4-dihydronaphthalene-2-carboxylate (17d)**

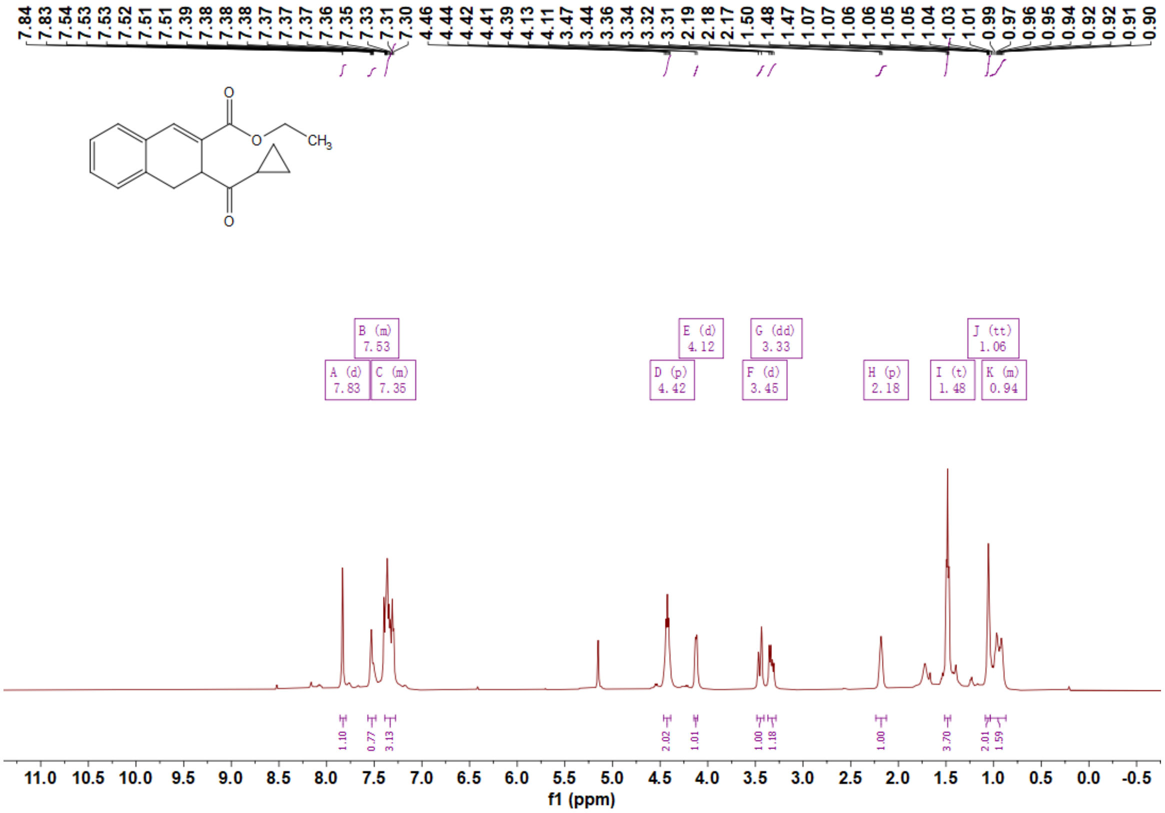


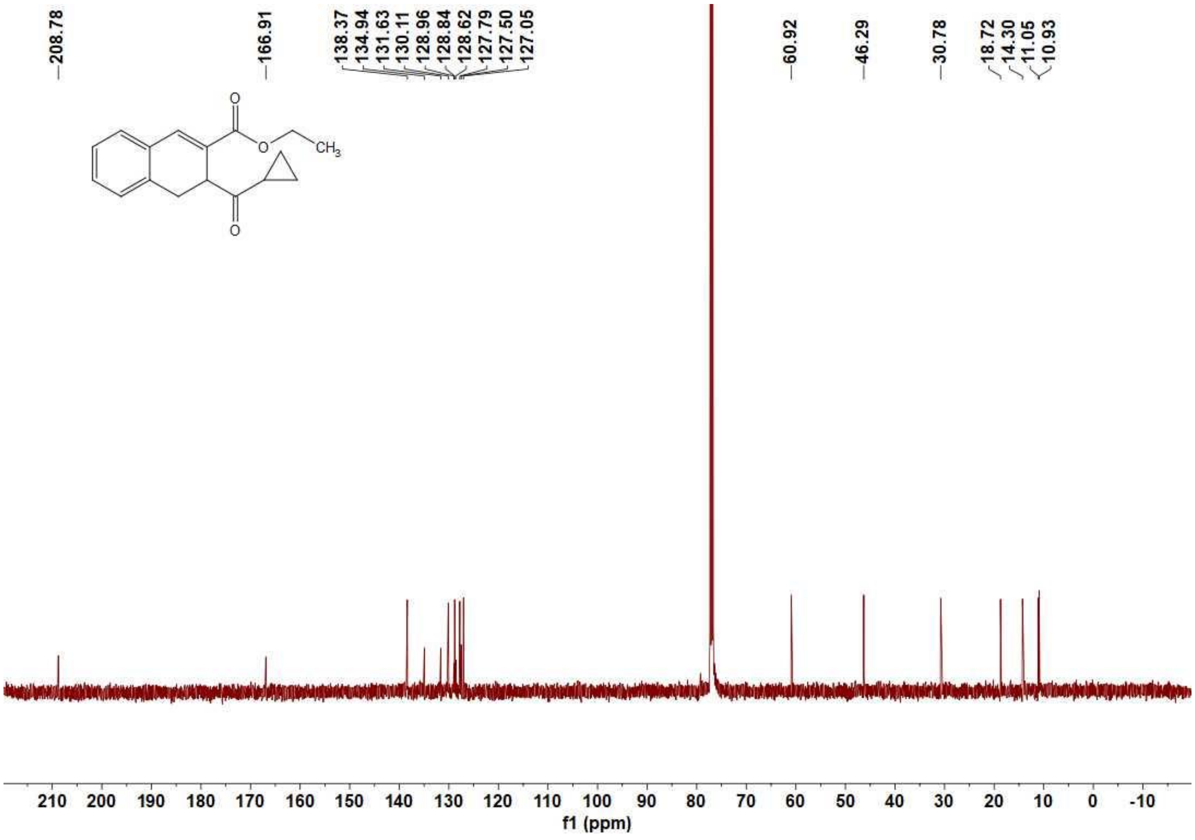

**Ethyl 3-(cyclohexanecarbonyl)-3,4-dihydronaphthalene-2-carboxylate (18d)**

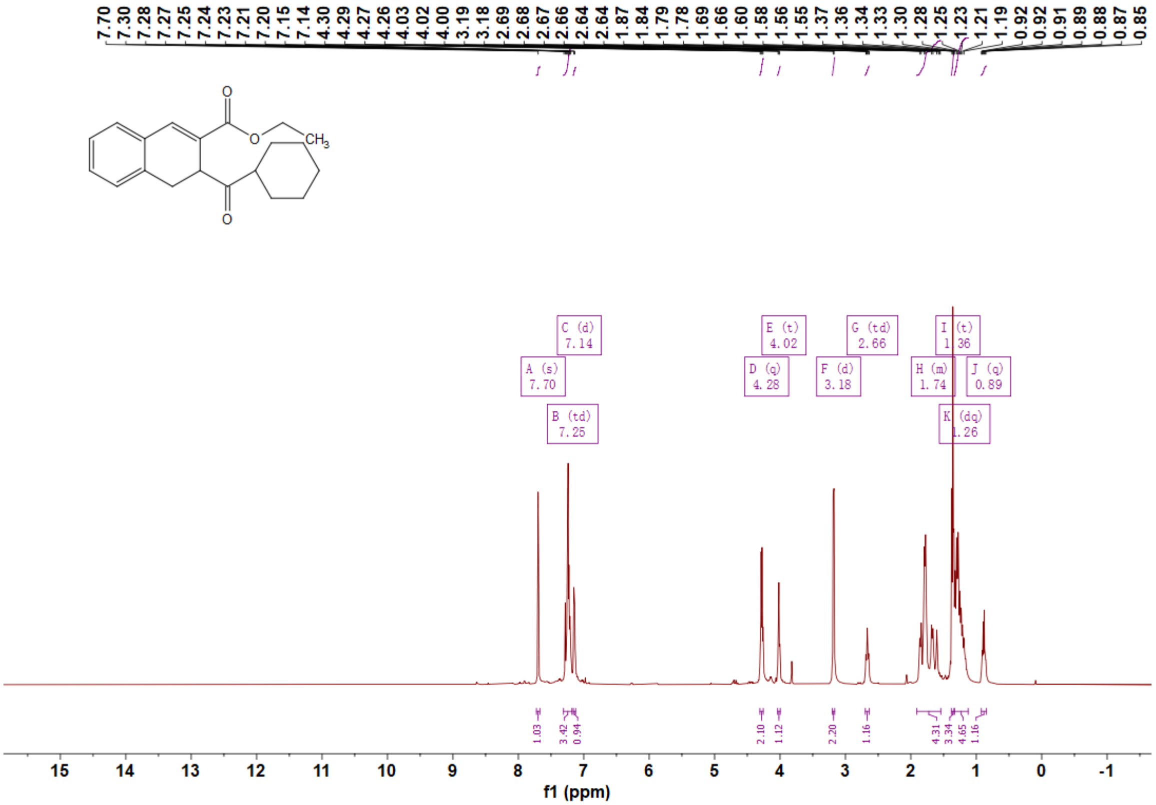

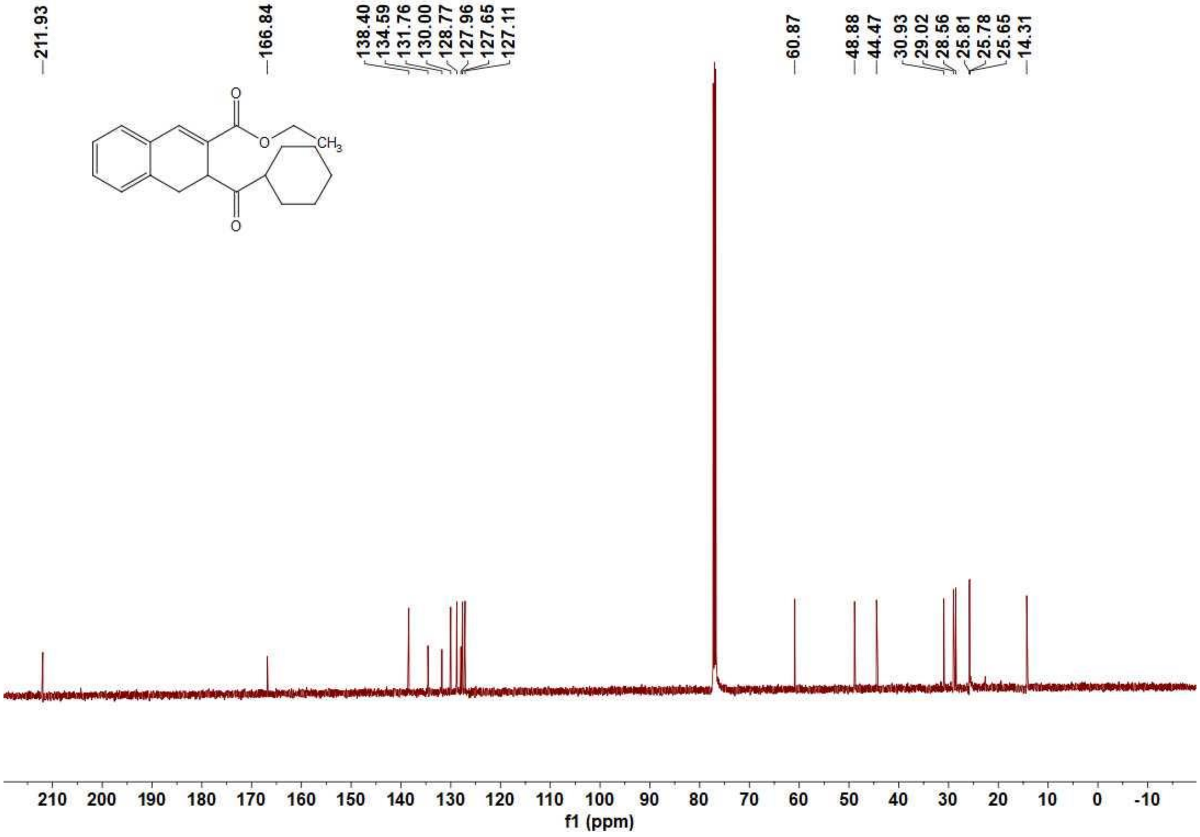

Supplement: Supplementary file 1 — ja2c11310_si_001.pdf [file ja2c11310_si_001.pdf]
